# Oxygenated Cyclohexene Derivatives from the Stem and Root Barks of *Uvaria pandensis*

**DOI:** 10.1021/acs.jnatprod.1c00811

**Published:** 2021-11-22

**Authors:** Gasper Maeda, Pieter J. Gilissen, Anastasia Rudenko, Jelle van der Wal, Catarina Bourgard, Arvind Kumar Gupta, Per Sunnerhagen, Joan J. E. Munissi, Stephen S. Nyandoro, Máté Erdélyi

**Affiliations:** † Chemistry Department, College of Natural and Applied Sciences, University of Dar es Salaam, Dar es Salaam, Tanzania; ‡ Department of Chemistry−BMC, Uppsala University, SE-751 23 Uppsala, Sweden; § Institute for Molecules and Materials, Radboud University, Heyendaalseweg 135, 6525 AJ Nijmegen, The Netherlands; ⊥ Department of Chemistry and Molecular Biology, University of Gothenburg, and Centre for Antibiotic Resistance Research (CARe) at the University of Gothenburg, SE-405 30 Gothenburg, Sweden; ∥ Department of Chemistry−Ångström, Uppsala University, SE-751 20 Uppsala, Sweden

## Abstract

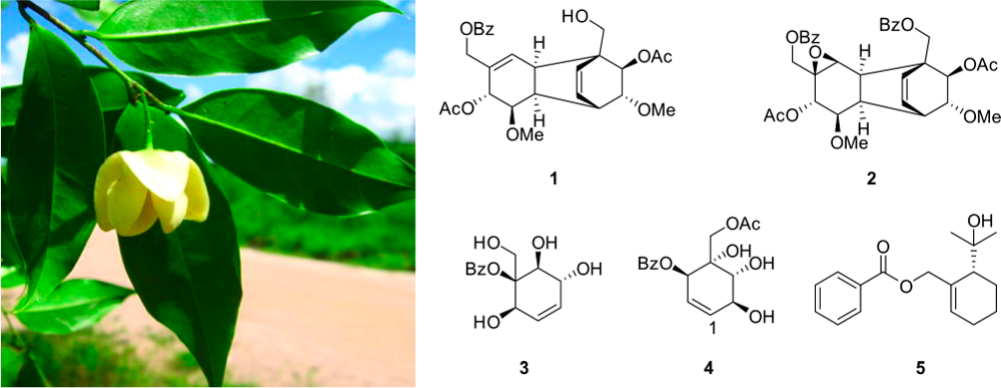

Five new cyclohexene derivatives, dipandensin A and B (**1** and **2**) and pandensenols A–C (**3**–**5**), and 16 known secondary metabolites (**6**–**21**) were isolated from the methanol-soluble extracts of the stem and root barks of *Uvaria pandensis*. The structures were characterized by NMR spectroscopic and mass spectrometric analyses, and that of 6-methoxyzeylenol (**6**) was further confirmed by single-crystal X-ray crystallography, which also established its absolute configuration. The isolated metabolites were evaluated for antibacterial activity against the Gram-positive bacteria *Bacillus subtilis* and *Staphylococcus epidermidis* and the Gram-negative bacteria *Enterococcus raffinosus*, *Escherichia coli*, *Paraburkholderia caledonica*, *Pectobacterium carotovorum*, and *Pseudomonas putida*, as well as for cytotoxicity against the MCF-7 human breast cancer cell line. A mixture of uvaretin (**20**) and isouvaretin (**21**) exhibited significant antibacterial activity against *B. subtilis* (EC_50_ 8.7 μM) and *S. epidermidis* (IC_50_ 7.9 μM). (8′α,9′β-Dihydroxy)-3-farnesylindole (**12**) showed strong inhibitory activity (EC_50_ 9.8 μM) against *B. subtilis*, comparable to the clinical reference ampicillin (EC_50_ 17.9 μM). None of the compounds showed relevant cytotoxicity against the MCF-7 human breast cancer cell line.

The genus *Uvaria* (Annonaceae) comprises about 168 species distributed throughout Africa and Asia, with additional species continuing to be described.^1^ Members of the genus *Uvaria* host a vast number of secondary metabolites with varied chemical structures and biological activities.^2^ The phytochemical constituents of the genus *Uvaria* include flavonoids^3^ and oxygenated cyclohexene derivatives, with the latter being restricted to the genus and its closely related genera within the Uvariae tribe,^4−14^ albeit with very few plant families other than Annonaceae.^15−21^ Thus, oxygenated cyclohexene derivatives have so far been reported from about 20 species of *Uvaria*(4,6−9,14,22−28) and other genera of the Uvariae tribe such as *Monanthotaxis*,^10,11,29^*Ellipeiopsis*,^12,30^*Dasymaschalon*,^31^*Cleistochlamys*,^13,32^ and *Artabotrys*.^33,34^ These compounds exhibit diverse bioactivities including antiproliferative,^15,18,25,35^ antimalarial,^6,9,13^ antibacterial,^32^ and cytotoxic^12,13,31^ activities.

Our recent findings of the bioactive oxygenated cyclohexene derivatives including their chlorinated counterparts from *Cleistochlamys kirkii*(13) and *Monathotaxis trichocarpa*(11) inspired a reinvestigation of *Uvaria pandensis* Verdc., a plant species previously reported to contain similar compounds.^6,36^ The species is used in some parts of Tanzania in traditional medicine to treat stomach disorders and fever.^11^ Reported herein are the isolation and structure determination of five hitherto unreported oxygenated cyclohexene derivatives (**1**–**5**), which were obtained with 16 known compounds (**6**–**21**) from the CH_3_OH extracts of stem and root bark samples. The isolated metabolites were evaluated for antibacterial activity against the Gram-positive bacteria *Bacillus subtilis* and *Staphylococcus epidermidis* and the Gram-negative bacteria *Enterococcus raffinosus*, *Escherichia coli, Paraburkholderia caledonica*, *Pectobacterium carotovorum*, and *Pseudomonas putida*. They were also assessed for cytotoxicity against the MCF-7 human breast cancer cell line.

## Results and Discussion

The separate CH_3_OH extracts of the root and stem barks of *U. pandensis* were subjected to a repeated silica gel chromatographic separation. Further purification on Sephadex LH-20 and by HPLC and recrystallization yielded 21 compounds, of which five (**1**–**5**) were new. The structures of the isolated secondary metabolites were elucidated based on their NMR, IR, and UV spectroscopic and mass spectrometric data. The 16 known compounds, 6-methoxyzeylenol (**6**),^5^ zeylenol (**7**),^35^ cleistenediol C (**8**),^13^ cleistenediol F (**9**),^13^ cherrevenol I (**10**),^9^ 3-methoxybenzylbenzoate (**11**),^11,37^ (8′α,9′β-dihydroxy)-3-farnesylindole (**12**),^36^ (6′,7′-dihydro-8′α,9′β-dihydroxy)-3-farnesylindole (**13**),^36^ zeylenyl-2,6-diacetate (**14**),^35^ benzoic acid 2,3-diacetoxy-1,6-dihydroxycyclohex-4-enylmethyl ester (**15**),^35^ lupeol (**16**),^38^ betulin (**17**),^39^ a mixture of stigmasterol (**18**)^11^ and β-sitosterol (**19**),^11^ and a mixture of uvaretin (**20**)^40^ and isouvaretin (**21**),^40^ were identified by comparison of their spectroscopic data (Supporting Information) to those previously reported in the literature.

**Chart 1:**
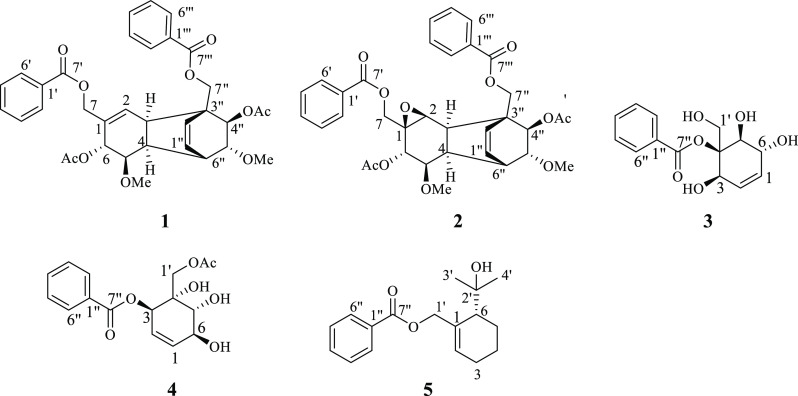


**Figure 1:**
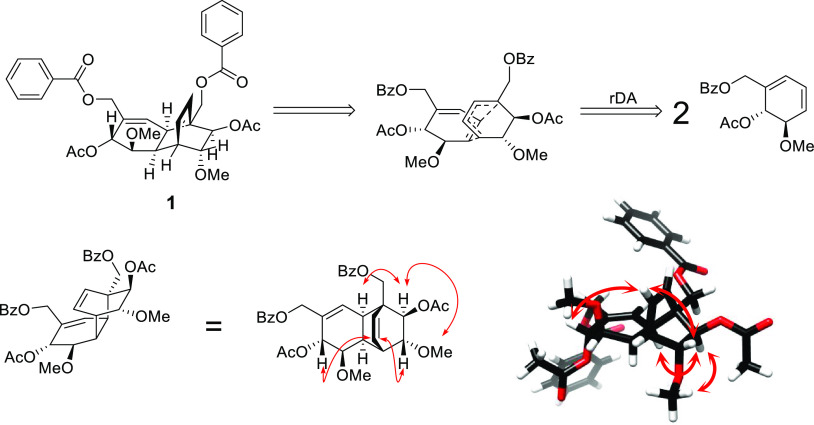
Retro-Diels–Alder analysis and key NOEs (red arrows) of compound **1**.

Compound **1**, [α]^24^_D_ −33 (*c* 0.2, CH_3_OH), was isolated from the CH_3_OH extract of the stem bark of *U. pandensis* as a white solid. The HRESIMS showed a [M + NH_4_]^+^ peak at *m*/*z* 622.2442 (calcd 622.2652 for C_34_H_40_NO_10_), which in combination with the NMR data (Table 1) indicated 17 double-bond equivalents (DBEs). Additionally, the HRMS showed a base peak at *m*/*z* 545.1937 (calcd for [M + H – HOAc]^+^ 545.2175), indicative of the presence of an acetate group. The UV spectrum exhibited absorption maxima at 208, 229, and 274 nm, typical for a conjugated π-system.^10^ The IR spectrum of compound **1** consisted of absorption bands for hydroxy (3520 cm^–1^), ester carbonyl (1740 cm^–1^), and double-bond (1635 cm^–1^) stretches. The ^1^H and ^13^C NMR spectroscopic data (Table 1, Figures S1–S2, Supporting Information) of compound **1** showed signals evocative of dimeric polyoxygenated cyclohexene derivatives resembling those previously reported.^8,15^ Thus, the ^1^H and ^13^C NMR spectra showed signals at δ_H/C_ 7.97 (H-2′/6′)/130.3, 7.50 (H-3′/5′)/129.5, 7.62 (H-4′)/134.1, 131.0 (C-1), 166.7 (C-7′) and 8.04 (H-2‴/6″′)/130.4, 7.50 (H-3″′/5′′′)/129.5, 7.62 (H-4′′′)/134.2, 131.2 (C-1′′′), 166.8 (C-7′′′), corresponding to two benzoyloxy units. In addition, the resonances were observed corresponding to two olefinic units [δ_H/C_ 135.2 (C-1), 6.02 (H-2)/129.5, 6.28 (H-1″)/132.6 and 5.75 (H-2″)/133.0], two oxymethylene groups [δ_H/C_ 4.77/4.62 (H-7a/7b)/65.3, 4.48 (2H, AB_q_, H-7″)/62.3], two acetoxy groups [δ_H/C_ 1.99 (CH_3_COO-6)/21.1 and 171.3; 1.96 (CH_3_COO-4″)/21.1 and 170.4], and two methoxy groups [δ_H/C_ 3.37 (OCH_3_-5)/57.9 and 3.32 (OCH_3_-5″)/57.2]. The COSY and TOCSY spectra (Figures S3 and S6, Supporting Information) showed correlations between H-2 (δ_H_ 6.02) and H-3 (δ_H_ 3.04), H-2 and H-6 (allylic coupling), H-3 and H-4 (δ_H_ 3.09), H-4 and H-5 (δ_H_ 3.48), H-3 and H-6 (long-range-coupling), H-5 and H-6 (δ_H_ 5.41), H-1″ (δ_H_ 6.28) and H-2″ (δ_H_ 5.75), H-1″ and H-6″ (δ_H_ 3.15), H-4″ (δ_H_ 5.09) and H-5″, and H-5″ (δ_H_ 3.10) and H-6″. These correlations indicated two distinct spin systems corresponding to two cyclohexenyl moieties. The linkage of these units and their substitution patterns were established from HMBC analyses (Table 1, Figure S5, Supporting Information). Thus, the HMBC correlations of δ_H_ 4.62/4.77 (H-7a/7b) to C-7′ (δ_C_ 166.7), C-1 (135.2), C-2 (129.5), and C-6 (δ_C_ 70.5) and those of δ_H_ 4.48 (H-7″) to C-7‴ (δ_C_ 166.8), C-2″ (δ_C_ 133.0), C-3″ (δ_C_ 47.6), and C-4″ (δ_C_ 76.5) indicated the two sets of benzoyloxymethylene units to be attached at C-1 and C-3″, respectively. The regiochemistry of the two methoxy and two acetate groups was indicated by the HMBC cross-peaks of CH_3_O-5 (δ_H_ 3.37), H-6 (δ_H_ 5.41), H-4″ (δ_H_ 5.09), and CH_3_O-5′’ (δ_H_ 3.32) to C-5 (δ_C_81.4), OAc-6 (δ_C_ 171.3), OAc-4″ (δ_C_ 170.4), and C-5″ (δ_C_ 87.1), respectively. The HMBC correlations of H-6″ (δ_H_ 3.15) to C-3 (δ_C_ 40.2), C-4 (δ_C_ 31.1), and C-5 (δ_C_ 81.4) as well as those of H-4 (δ_H_ 3.09) to C-1″ (δ_C_ 132.6) and C-6″ (δ_C_ 34.1), and of H-5 (δ_H_ 3.09) to C-6″ (δ_C_ 34.2) suggested the linkage of the two sets of cyclohexenyl moieties. The NMR and MS data supported by retro-biogenetic analysis led to the conclusion that **1** is a dimer of 6-acetoxy-5-methoxy-1,3-dienyl)methylbenzoate moieties and hence the product of the Diels–Alder *endo*-addition (Figure 1). The fact that **1** was isolated as an optically active compound suggests that it is formed in an enzymatic Diels–Alder reaction, as the nonenzymatic alternative would be expected to provide a racemic product.^41^

**Table 1:** NMR Spectroscopic Data (500 MHz, CD_3_CN) for Dipandensin A (**1**)

position	δ_C_, type	δ_H_	(*J* in Hz)	HMBC
1	135.2, C			
2	129.5, CH	6.02	dd (2.9, 2.4)	C-1, C-3, C-4, C-6, C-7, C-3″
3	40.2, CH	3.04	ddd (9.0, 2.9, 2.4)	C-1, C-2, C-4, C-5, C-2″, C-3″, C-4″, C-6″
4	31.1, CH	3.09	dd (9.0, 6.5)	C-2, C-3, C-5, C-6, C-1″, C-3″, C-5″, C-6″
5	81.4, CH	3.48	dd (9.1, 6.5)	C-1, C-3, C-4, C-6, C-1″, OCH_3_-5
CH_3_O-5	57.9, CH_3_	3.37	s	C-5
6	70.5, CH	5.41	ddd (9.1, 2.4, 2.4)	C-1, C-2, C-4, C-5, COO-6
OAc-6	171.3, OC=O			
	21.1, CH_3_	1.99		COO-6
7	65.3, CH_2_	4.77	d (12.4)	C-1, C-2, C-6, C-7′
		4.62	d (12.4)	C-1, C-2, C-6, C-7′
1′	131.0, C			
2′/6′	130.3, CH	7.97	dd (8.3, 1.5)	C-1′, C-3′/5′, C-4′, C-7′
3′/5′	129.5, CH	7.50	dd (8.3, 7.5)	C-1′, C-2′/6′, C-4′
4′	134.1, CH	7.62	dd (7.5, 1.5)	C-2′/6′, C-3′/5′
7′	166.7, OC=O			
1″	132.6, CH	6.28	dd (8.2, 6.8)	C-4, C-2″, C-3″, C-5″, C-6″
2″	133.0, CH	5.75	d (8.2)	C-3″, C-4″, C-6″, C-7″
3″	47.6, C			
4″	76.5, CO	5.09	d (2.9)	C-2″, C-3″, C-5″, C-6″, C-7″, COO-4″
OAc-4″	170.4, OC=O			
	21.1, CH_3_	1.96		COO-4″
5″	87.1, CO	3.10	dd (2.9, 2.9)	C-1″, C-3″, C-4″, OCH_3_-5″, C-6″
CH_3_O-5″	57.2, CH_3_	3.32	s	C-5″
6″	34.1, CH	3.15	dd (6.8, 2.9)	C-3, C-4, C-5, C-1″, C-2″, C-4″, C-5″,
7″	62.3, CH_2_	4.48	AB_q_ (12.0)	C-3, C-2″, C-3″, C-4″, C-7‴
1‴	131.2, C			
2‴/6‴	130.4, CH	8.04	dd (8.4, 1.5)	C-1‴, C-3‴/5‴, C-4‴, C-7‴
3‴/5‴	129.5, CH	7.50	dd (8.4, 7.7)	C-1‴, C-2‴/6‴, C-4″
4‴	134.2, CH	7.62	tt (7.7, 1.5)	C-2‴/6‴, C-3‴/5‴
7‴	166.8, OC=O			

The relative configuration of **1** was established based on scalar coupling constants (Table 1) and NOESY correlations (Figure 1 and Figure S7, Supporting Information). Thus, ^3^*J*_H-4/5_ (6.5 Hz) and ^3^*J*_H-5/6_ (9.1 Hz) indicated the *cis* axial–equatorial and the *trans* diaxial orientation for the corresponding protons, establishing the relative configurations at C-4, C-5, and C-6. Moreover, H-6 exhibited a NOE with H-1″, which exhibited also a weak NOE correlation with H-5″. The NOE correlation of CH_3_O-5″ to H-4″ was used to establish the relative configurations at C-4″, C-5″, and C-6″. The ^3^*J*_H-3/4_ (9.0 Hz) indicated a *cisoid* ring junction for the protons at C-3 and C-4. Such a large *J* value for the bridgehead protons suggested that their dihedral angle is inclined toward an *eclipsed* orientation.^42^ Based on the above spectroscopic data analysis, the new compound **1** (dipandensin A) was characterized as benzoic acid 6α,4″β-diacetoxy-3α-methylbenzoate-5β,5″α-dimethoxytricyclo[6.2.2.0^3,6″^]dodeca-2,2″-dien-1-ylmethyl ester.

**Figure 2:**
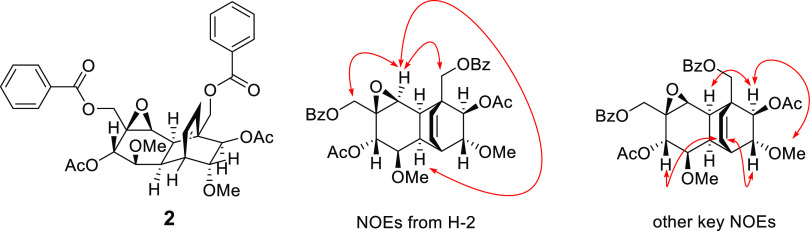
Key NOEs for compound **2**, indicated as red arrows.

Compound **2**, [α]^24^_D_ −40 (*c* 0.2, CH_3_OH), was isolated from the stem bark CH_3_OH extract as a white solid. Its HRESIMS (Figure S16, Supporting Information) spectrum exhibited a molecular ion [M + H]^+^ at *m*/*z* 621.2297 (calcd 621.2336), corresponding to the molecular formula C_34_H_36_O_11_ with 17 DBEs, similar to **1**. The established molecular formula was supported by the NMR data (Table 2). The UV spectrum exhibited absorption maxima at 205 and 230 nm, revealing a conjugated π-system.^10^ The IR spectrum, again similar to that of **1**, showed absorption bands for hydroxy (3508 cm^–1^), ester carbonyl (1730 cm^–1^), and C=C bond (1630 cm^–1^) stretches. The NMR spectroscopic data (Table 2 and Figures S9–S15, Supporting Information) closely resembled those of dipandensin A (**1**), with the only difference being saturation at C-1 and C-2. Thus, **2** exhibited a signal at δ_H_ 3.43 (δ_C_ 61.7) assignable to C-2, compared with δ_H_ 6.02 (δ_C_ 129.5) for the same position in **1**. In addition, the ^13^C NMR data (Table 2, Figure S10, Supporting Information) of **2** consisted of a peak at δ_C_ 60.8 for a tertiary oxygenated carbon, assignable to C-1, instead of δ_C_ 135.2 observed for C-1 in **1**. The carbon resonances at C-1 (60.8) and C-2 (61.7) were in agreement with a 1,2-epoxide derivative of **1**. For a 1,2-diol, the carbons would have been expected to resonate at ∼70 ppm. The oxymethine proton at δ_H_ 3.43 (H-2) showed HMBC cross-peaks to C-1 (δ_C_ 60.8), C-3 (δ_C_ 38.6), C-4 (δ_C_ 32.8), C-6 (δ_C_ 70.3), C-7 (δ_C_ 63.8), and C-3″ (δ_C_ 47.1), supporting its proposed positioning. The relative configurations at C-1 and C-2 were established based on the NOEs of H-2 (δ_H_ 3.43) with H-3 (δ_H_ 2.88), H-4 (δ_H_ 2.91), H-7 (δ_H_ 4.01), and H-7″ (δ_H_ 4.64) (Figure 2 and Figure S9, Supporting Information), indicating these protons to be cofacial. No scalar coupling was measurable between H-2 and H-3, consistent with their dihedral angle being close to 90°. The *J*_H-3/4_ (9.1 Hz) suggested their dihedral angle to be inclined toward an *eclipsed cis* orientation for these protons, whereas the ^3^*J*_H-5/6_ (9.9 Hz) indicated their *transoid* orientation, similar to that in **1**. In compound **1**, an allylic coupling of H-2 with H-6 was observed. This coupling was absent for H-2 and H-6 of the saturated cyclohexanyl ring of compound **2**. The relative configurations of the other parts of **2** were similar to those of **1** as established by analysis of the NOEs (Figure 2 and Figure S9, Supporting Information) and of scalar couplings (Table 2). The HMBC analysis established the linkage of the subunits of **2** with all key cross-peaks being similar to **1** (Tables 1 and 2). Based on the spectroscopic data obtained, this new compound, dipandensin B (**2**), was characterized as benzoic acid 4″β,6α-diacetoxy-1β,2β-epoxy-3″α-methylbenzoate-5β,5″α-dimethoxytricyclo[6.2.2.0^4,5^]dodeca-1″-en-1-ylmethyl ester. Dimeric polyoxygenated cyclohexene derivatives, such as **1** and **2**, are rare natural products with restricted occurrence in only a few plant species. They have been reported previously from *Uvaria cherrevensis*(8) and *Kaempferia rotunda* (Zingiberaceae).^16^

**Table 2:** NMR Spectroscopic Data (500 MHz, CD_3_CN) for Dipandensin B (**2**)

position	δ_C_, type	δ_H_	(*J* in Hz)	HMBC
1	60.8, C			
2	61.7, CH	3.43	br, s	C-1, C-3, C-4, C-6, C-7, C-3″
3	38.6, CH	2.88	d (9.1)	C-1, C-2, C-4, C-5, C-2″, C-3″, C-6″, C-7″
4	32.8, CH	2.91	dd (9.1, 5.7)	C-2, C-3, C-6, C-1″, C-3″, C-5″, C-6″
5	77.8, CH	3.62	dd (9.9, 5.7)	C-1, C-3, C-4, OCH_3_-5, C-6, C-6″
CH_3_O-5	58.1, CH_3_	3.28	S	C-5
6	70.3, CH	5.29	d (9.9)	C-1, C-2, C-4, C-5, C-7, COO-6
OAc-6	171.1, OC=O			
	21.0, CH_3_	2.07	s	COO-6
7	63.8, CH_2_	4.42	d (11.7)	C-1, C-2, C-6, C-7
		4.01	d (11.7)	C-1, C-2, C-6, C-7
1′	130.9, C			
2′/6′	130.5, CH	8.05	m	C-1′, C-3′/5′, C- 4′, C-7′
3′/5′	129.7, CH	7.53	m	C-1′, C-2′/6′, C-4′
4′	134.4, CH	7.65	m	C-2′/6′, C-3′/5′
7′	166.8, C=O			
1″	133.3, CH	6.30	dd (8.1, 7.5)	C-4, C-2″, C-3″, C-5″, C-6″
2″	132.2, CH	5.85	d (8.1)	C-3″, C-4″, C-6″, C-7″
3″	47.1, C			
4″	75.7, CH	5.07	d (2.9)	C-5, C-2″, C-3″, C-5″, C-6″, COO-4″
OAc-4″	170.4, OC=O			
	21.0, CH_3_	1.96	s	COO-4″
5″	87.0, CH	3.02	dd (3.0, 2.9)	C-1″, C-3″, C-4″, OCH_3_-5″, C-6″
CH_3_O-5″	57.0, CH_3_	3.27	s	C-5″
6″	33.7, CH	3.09	dd (7.5, 3.0)	C-3, C-4, C-5, C-1″, C-2″, C-4″, C-5″
7″	61.6, CH_2_	4.64	d (12.2)	C-3, C-2″, C-3″, C-4″, C-7″
		4.52	d (12.2)	C-3, C-2″, C-3″, C-4″, C-7″
1‴	130.7, C			
2‴/6‴	130.4, CH	8.04	m	C-1‴, C-3‴/5‴, C-4‴, C-7‴
3‴/5‴	129.5, CH	7.53	m	C-1‴, C-2‴/6‴, C-4‴
4‴	134.2, CH	7.65	m	C-2‴/6‴, C-3‴/5‴
7‴	166.5, C=O			

**Figure 3:**
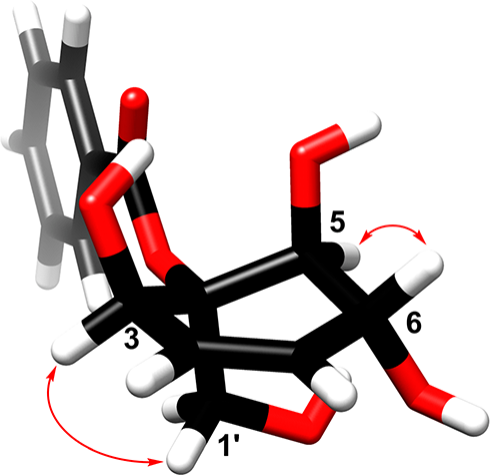
Key NOEs for compound **3**, shown as red arrows.

Compound **3**, [α]^24^_D_ −173 (*c* 0.2, CH_3_OH), was isolated from the CH_3_OH extract of the stem bark of *U. pandensis* as a colorless oil. HRESIMS (Figure S24, Supporting Information) indicated a molecular ion [M + H]^+^ peak at *m*/*z* 281.1025 (calcd 281.1025), which along with the NMR data (Table 3) established the molecular formula C_14_H_16_O_6_, suggesting 7 DBEs. The IR spectrum showed absorptions at 3330 cm^–1^ for hydroxy, 2970 and 2944 cm^–1^ for aliphatic C–H, 1737 cm^–1^ for ester carbonyl, and 1601 cm^–1^ for C=C stretches. The UV spectrum displayed absorptions at 206 and 229 nm, corresponding to conjugated π-system. Unlike compounds **1** and **2**, the NMR data of compound **3** (Table 3, Figures S17–S23 Supporting Information) exhibited signals for only one cyclohexenyl core with benzoyloxy and oxymethylene substituents. The NMR data of **3** consisted of signals corresponding to a tetra-oxygenated cyclohexene skeleton (δ_H/C_ 5.72 (H-1)/132.8, 5.71 (H-2)/128.2, 4.02 (H-3)/71.2, 3.65 (H-5)/74.4, 4.18 (H-6)/71.7; C-4 (76.4)), a benzoyloxy unit (δ_H/C_ 8.02 (H-2″/6″)/130.5, 7.60 (H-4″)/132.8, 7.48 (H-3″/5″)/129.6, C-1 (134.2), C-7″ (169.4)), and an oxymethylene unit (δ_H/C_ 3.82 (H-1′)/66.2). Contrary to **1** and **2** and to most polyoxygenated cyclohexenyl derivatives,^4−19,21−35,43−45^ compound **3** lacked an HMBC cross-peak (Figure S21, Supporting Information) from the oxymethylene protons (CH_2_-1′; δ_H_ 3.82, δ_C_ 66.2) to a carbonyl carbon, indicating that the oxymethylene unit is not connected to the benzoyloxy moiety. Thus, the HMBC correlations of CH_2_-1′ (δ_H_ 3.82) to C-3 (δ_C_ 71.2), C-4 (δ_C_76.4), and C-5 (δ_C_ 74.3) supported the placement of the oxymethylene functionality at C-4. The benzoyloxy carbonyl did not show HMBC correlations to any protons except H-2″/H-6″, indicating that it is attached to the oxycarbon (C-4), making **3** distinctive among polyoxygenated cyclohexene derivatives. The scalar coupling constant ^3^*J*_H-5/6_ of 7.7 Hz suggested H-5 and H-6 to be *trans*-diequatorial. In addition, the weak NOEs observed for H-5/H-6 and H-3/H-1′ (Figure 3 and Figure S23, Supporting Information) revealed the relative configurations at C-3, C4, C-5, and C-6. Based on the above spectroscopic data, pandensenol A (**3**) was characterized as benzoic acid 3β,5β,6α-trihydroxy-4α-hydroxymethylcyclohex-1-enyl ester.

**Table 3:** NMR Spectroscopic Data (500/MHz, CD_3_OD) for Pandensenol A (**3**)

position	δ_C_, type	δ_H_	(*J* in Hz)	HMBC
1	132.8, CH	5.72	dd (10.2, 1.3)	C-2, C-3, C-5, C-6
2	128.2, CH	5.71	dd (10.2, 4.1)	C-1, C-3, C-4, C-6
3	71.2, CH	4.02	d (4.1)	C-1, C-2, C-4, C-5, C-1′
4	76.4, C			
5	74.3, CH	3.65	d (7.7)	C-1, C-3, C-4, C-6, C-1′
6	71.7, CH	4.18	dd (7.7, 1.3)	C-1, C-2, C-4, C-5
1′	66.2, CH_2_	3.82	ABq (11.5)	C-3, C-4, C-5
2′	169.4, C=O			
1″	134.2, C			
2″/6″	130.5, CH	8.02	dd (7.8, 2.1)	C-1″, C-3″/5″, C-4″
3″/5″	129.6, CH	7.48	dd (7.8, 7.7)	C-1″, C-2″/6″, C-4″
4″	132.8, CH	7.60	tt (7.7, 2.1)	C-2″/6″, C-3″/5″

**Figure 4:**
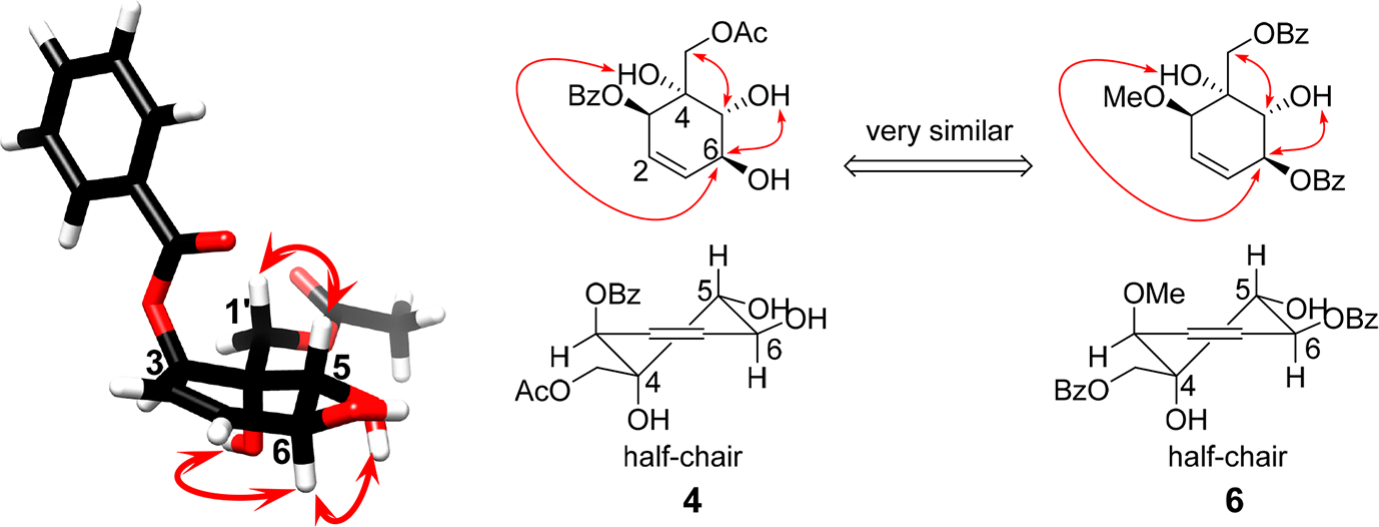
Key NOEs of compounds **4** and **6**, indicated as red arrows.

**Figure 5:**
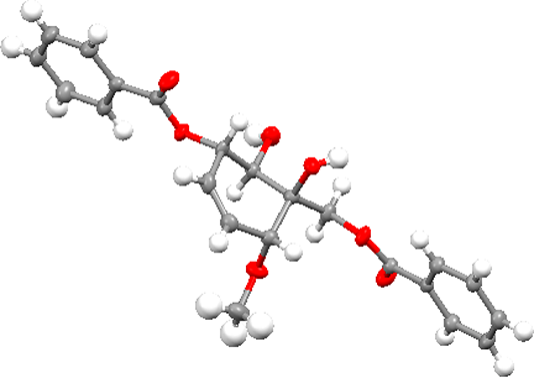
Solid-state structure of 6-methoxyzeylenol (**6**) shown as thermal ellipsoids with 50% probability levels.

Compound **4**, [α]^24^_D_ +85 (*c* 0.3, CH_2_Cl_2_), was isolated from the CH_3_OH extract of the stem bark of *U. pandensis* as a white solid. The HRESIMS (Figure S32, Supporting Information) exhibited a molecular ion [M + H]^+^ peak at *m*/*z* 323.1191 (calcd 323.1131), corresponding to the molecular formula C_16_H_18_O_7_, which indicated eight DBEs, corresponding to a benzene ring, a cyclohexene ring, and two carbonyl groups (NMR data, Table 4). The UV spectrum showed an absorption maximum at 230 nm, indicating a conjugated π-system. The IR spectrum exhibited absorption bands for hydroxy (3318 cm^–1^), aliphatic C–H (2943 and 2832 cm^–1^), C=O (1678 cm^–1^), and C–O (1274 cm^–1^) stretches. The ^1^H NMR spectrum (Table 4 and Figure S25, Supporting Information) displayed signals typical of a polyoxygenated cyclohexene derivative. Thus, the ^1^H NMR spectrum consisted of signals corresponding to protons of a benzoyloxy moiety [δ_H_ 8.00 (H-2″/6″), 7.50 (H-3″/5″), 7.63 (H-4″)], an olefinic moiety [δ_H_ 5.90 (H-1), 5.76 (H-2)], three oxymethines [δ_H_ 5.41 (H-3), 4.18 (H-6), 3.76 (H-5)], an oxymethylene (δ_H_ 4.31), and an acetoxy moiety (δ_H_ 1.87, CH_3_-1‴). The corresponding carbon signals were assigned based on HSQC experiments (Table 4, Figure S28, Supporting Information). The COSY and TOCSY correlations (Figures S27 and S30, Supporting Information) for signals at δ_H_ 3.76 (H-5), 4.18 (H-6), 5.90 (H-1), 5.76 (H-2), and 5.41 (H-3) indicated a continuous spin system, confirming the presence of a cyclohexene core skeleton. The H-2 proton further exhibited allylic coupling with H-6, which in turn coupled with OH-6. Contrary to the previously described polyoxygenated cyclohexene derivatives, the oxymethylene unit (δ_H_ 4.31, δ_C_ 67.2) and the acetoxy protons (δ_H_ 1.87) showed mutual HMBC cross-peaks (Figure S29, Supporting Information) to the C-1‴ (δ_C_ 171.5) carbonyl carbon, which supported the presence of an acetoxymethylene unit. The HMBC cross-peaks of H-3 (δ_H_ 5.41) to C-1 (δ_C_ 135.8), C-4 (δ_C_ 74.2), C-5 (δ_C_ 75.3), C-1′ (δ_C_ 67.2), and C-7″ (δ_C_ 165.9), together with those of H-1′ (δ_H_ 4.31) to C-3 (δ_C_ 71.5), C-4 (δ_C_ 74.2), C-5 (δ_C_ 75.3), and C-1‴ (δ_C_ 171.5), corroborated the placement of the benzoyloxy and acetoxymethylene units at C-3 and C-4, respectively. The relative configurations of H-5 and H-6 were established as being *trans* (^3^*J*_H-5/6_ = 7.6 Hz) in solution. Moreover, the NOE correlations (Figure 4 and Figure S31, Supporting Information) of H-6 (δ_H_ 4.18) to OH-4 (δ_H_ 3.42) and OH-5 (δ_H_ 3.34) and that of H-5 (3.76) to H-1′ (δ_H_ 4.31) facilitated assignment of the relative configuration of compound **4** at C-3, C-4, C-5, and C-6. Coincidently, the key NOEs of compounds **4** and **6** were similar (Figures 4, S31 and S47, Supporting Information). A single-crystal X-ray structure of compound **6** was obtained (Figure 5), which further supported the configurational assignments of both **4** and **6**. Based on the above-mentioned spectroscopic and X-ray crystallographic evidence, this new compound, pandensenol B (**4**), was characterized as benzoic acid 1β-acetoxymethyl-4α,5α,6 β-trihydroxy-cyclohex-1-enyl.

**Table 4:** NMR Spectroscopic Data (600 MHz, CD_3_CN) for Pandensenol B (**4**)

position	δ_C_, type	δ_H_	(*J* in Hz)	HMBC
1	135.8, CH	5.90	dd (10.0, 2.1)	C-2, C-3, C-5, C-6
2	123.3, CH	5.76	ddd (10.0, 4.4, 2.2)	C-1, C-3, C-4, C-6
3	71.5, CH	5.41	d (4.4)	C-1, C-2, C-4, C-5, C-1′, C-7″
4	75.3, C			
5	74.2, CH	3.76	dd (7.6, 5.7)	C-1, C-3, C-4, C-6, C-1′
6	70.7, CH	4.18	dddd (7.6, 6.4, 2.2, 2.1)	C-1, C-2, C-4, C-5
1′	67.2, CH_2_	4.31	ABq (11.7)	C-3, C-4, C-5, C-1‴
1″	130.8, C			
2″/6″	130.3, CH	8.00	dd (8.4, 1.4)	C-1″, C-3″/5″, C-4″
3″/5″	129.6, CH	7.50	dd (8.4, 7.6)	C-1″, C-2″/6″, C-4″
4″	134.3, CH	7.63	tt (7.6, 1.4)	C-2″/6″, C-3″/5″
7″	165.9, OC=O			
OAc-1′	171.5, OC=O			
	20.8, CH_3_	1.87	s	COO-1′
OH-4		3.42	s	C-3, C-4, C-5, C-1′
OH-5		3.34	d (5.7)	C-4, C-5, C-6
OH-6		3.28	d (6.4)	C-1, C-5, C-6

Compound **5**, [α]^24^_D_ +81 (*c* 0.2, CH_3_OH), was isolated from the CH_3_OH extract of the root bark of *U. pandensis* as a white solid. It was assigned the molecular formula C_17_H_22_O_3_ based on HRESIMS ([M + H]^+^*m*/*z* 275.1674, calcd 275.1647; Figure S40, Supporting Information) and NMR data (Table 5) analyses. Its UV spectrum exhibited absorption maxima at 206, 228, and 273 nm corresponding to a conjugated π-system. The IR spectrum displayed absorption bands for hydroxy (3439 cm^–1^), C–H (2968 and 2926 cm^–1^), C=O (1718 cm^–1^), C=C (1601 cm^–1^), and C–O (1271 cm^–1^) stretches. Its NMR spectroscopic data (Table 5, Figures S33 and S34, Supporting Information) were to a large extent comparable to those of the polyoxygenated cyclohexene derivatives described above. The ^1^H and ^13^C NMR spectroscopic data consisted of signals corresponding to benzoyloxy and oxymethylene units, similar to those observed for compounds **1**–**4**. Furthermore, the ^1^H NMR data (Table 5) and multiplicity-edited HSQC data (Figure S36, Supporting Information) showed signals for an olefinic proton at δ_H/C_ 5.84 (H-2)/125.9, methylene protons at δ_H/C_ 2.00 (H-3)/26.4, two sets of diastereotopic protons at δ_H/C_ 2.18/1.90 (H-4a/H-4b)/26.8 and δ_H/C_ 1.98/1.32 (H-5a/H-5b)/23.6, a methine proton at δ_H/C_ 1.58 (H-6)/45.0, and two methyl group protons at δ_H/C_ 1.20 (H-3′)/27.1 and 1.21 (H-4′)/27.6. The COSY and TOCSY (Figures S35 and S38, Supporting Information) coupling patterns of these signals suggested the presence of a cyclohexene core structure. Thus, COSY correlations were observed for H-2 and H-3, H-4b (*W*-coupling), and H-1′ (allylic coupling). The H-3 proton further coupled to H-4a/b, which in turn coupled to H-5a/b, while H-6 coupled to H-5a/b and to H-4b (*W*-coupling).

**Table 5:** NMR Spectroscopic Data (500 MHz, CDCl_3_) for Pandensenol C (**5**)

position	δ_C_, type	δ_H_	(*J* in Hz)	HMBC
1	130.5, C			
2	125.9, CH	5.84	m	C-1, C-3, C-4, C-6, C-1′
3	26.4, CH_2_	2.20	m	C-1, C-2, C-4, C-5
4	26.8, CH_2_	1.90	m	C-2, C-3, C-5, C-6
		2.18	m	
5	23.6, CH_2_	1.98	ddd (12.5, 5.3, 2.3)	C-1, C-3, C-4, C-6, C-2′
		1.32	ddd (13.9, 12.5, 5.3)	C-1, C-3, C-4, C-6, C-2′
6	45.0, CH	1.58	dddd (13.9, 11.5, 4.9, 2.5)	C-1, C-4, C-5, C-2′, C-3′, 4′
1′	68.9, CH_2_	4.72	m	C-1, C-2, C-6, C-7″
2′	72.7, C–O			
3′	27.1, CH_3_	1.20	s	C-2′, C-4′
4′	27.6, CH_3_	1.21	s	C-2′, C-3′
1″	133.1, C			
2″/6″	129.7, CH	8.05	dd (8.2, 1.4)	C-3″/5″, C-4″, C-7″
3″/5″	128.5, CH	7.44	dd (8.2, 7.4)	C-2″/6″, C-4″, C-1″
4″	133.0, CH	7.56	tt (7.4, 1.4)	C-2″/6″, C-3″/5″
7″	166.6, C=O			

The cyclohexene core structure was confirmed by the ^3^*J* HMBC cross-peaks of H-2 (δ_H_ 5.84) to C-4 (δ_C_ 26.6) and C-6 (δ_C_ 45.0), H-3 (δ_H_ 2.00) to C-1 (δ_C_130.5) and C-5 (δ_C_ 23.6), and H-6 (δ_H_1.58) to C-2 (δ_C_ 125.9) and C-4 (δ_C_ 26.6) (Table 5 and Figure S37, Supporting Information). In contrast to compounds **1**–**4**, structure **5** lacks oxygenation within the cyclohexene skeleton. Instead, its ^1^H NMR spectrum exhibited signals at δ_H_ 1.21 (3H, s, H-4′) and 1.20 (3H, s, H-3′), which were assigned to two methyl groups substituted at the carbinol carbon C-2′ (δ_C_ 72.7). This indicated the presence of an oxygenated isopropyl unit, which is different from the cyclohexene derivatives so far reported in the literature,^4−14^ and was corroborated by the HMBC cross-peaks of H-6 (δ_H_ 1.58) to C-2′ (δ_C_ 72.7) and those of the methyl groups (δ_H_ 1.21 and 1.20) to C-6 (δ_C_ 45.0) and C-2′ (δ_C_ 72.7). The position of the benzoyloxymethylene unit was established at C-1 (δ_C_ 130.5), based on the cross-peaks of the oxymethylene protons H-1′ (δ_H_ 4.72) to C-6 (δ_C_ 45.0), C-1 (δ_C_ 130.5), and C-2 (δ_C_ 125.9). This was supported further by the HMBC cross-peaks of H-2 (δ_H_ 5.84) and H-6 (δ_H_ 1.58) to C-1′ (δ_C_ 68.9). The HMBC correlations of the oxymethylene protons (δ_H_ 4.72) and those of H-2″/6″ (δ_H_ 8.05) to C-7″ (δ_C_ 166.6) were consistent with the attachment of the methylene unit to the benzoyl unit. Based on the above spectroscopic evidence, the new compound pandensenol C (**5**) was characterized as benzoic acid 1-(2′-hydroxy-2′-methylethyl)cyclohex-1-enyl methyl ester.

As similar secondary metabolites were reported to exhibit antibacterial and cytotoxic activities,^12,31,32^ the isolated compounds were evaluated for their activity against the Gram-positive bacteria *Bacillus subtilis* and *Staphylococcus epidermidis* and the Gram-negative bacteria *Enterococcus raffinosus*, *Escherichia coli*, *Paraburkholderia caledonica*, *Pectobacterium carotovorum*, and *Pseudomonas putida* as well as for cytotoxicity against MCF-7 human breast cancer cells. The mixture of uvaretin and isouvaretin (**20** and **21**) obtained exhibited activity against *B. subtilis* (EC_50_ 8.7 μM) and *S. epidermidis* (IC_50_ 7.9 μM), but showed only moderate activity against *E. coli* (EC_50_ 1130.8 μM) and *P. carotovorum* (EC_50_ 263.1 μM). Compound **13** also showed modest activity against *B. subtilis*, EC_50_ 1154.1 μM, whereas compound **12** gave strong inhibition with an EC_50_ of 9.8 μM (Figure S148, Supporting Information) and was comparable to the known ampicillin antibiotic clinical reference value (EC_50_ 17.9 μM, Figure S151, Supporting Information). All other compounds showed no or low activity against the tested bacterial strains (MIC > 725 μM) and no relevant cytotoxicity (EC_50_ > 10 μM) (Figure S150, Supporting Information).

In conclusion, 21 natural products including the five new cyclohexene derivatives **1**–**5** were isolated and characterized from separate CH_3_OH extracts of the stem and root barks of *U. pandensis*. This is the first report of all but compounds **12**(36) and **13**(36) from this plant. Polyoxygenated cyclohexenes and *C*-benzylated chalcones have restricted occurrence in plants. They are known to be produced by the members of the Uvariae tribe of the family Annonaceae. Hence, their occurrence in *U. pandensis* is of chemotaxonomic importance, confirming the placement of this plant in the Uvariae taxon. Some of the isolated compounds showed activity against Gram-positive bacteria, along with low to moderate cytotoxicity.

## Experimental Section

### General Experimental Procedures

Optical rotations were determined using a 341LC OROT polarimeter (589 nm, 20 °C), whereas UV measurements were done using a 264 UV–vis spectrophotometer. A MIR 450FT-IR spectrometer was used to record the IR spectra. NMR spectra were acquired on either a Bruker Avance III HD 600/500 or 400 NMR MHz spectrometer and were analyzed with the MestReNova (v10.0.0) software. Structural assignments were based on ^1^H NMR, ^13^C NMR, COSY, TOCSY, NOESY, HSQC, and HMBC spectra. LC-MS (ESI) spectra were acquired with a PerkinElmer PE SCIEX API 150 EX instrument equipped with a Turbolon spray ion source and a Gemini 5 mm RP-C_18_ 110 Å column, using a gradient of H_2_O–CH_3_CN (80:20 to 20:80) in the presence of 0.2% HCO_2_H and a separation time of 8 min. HRESIMS were obtained with a Q-TOF-LC/MS spectrometer with a lock mass ESI source (Stenhagen Analysis Lab AB, Gothenburg, Sweden), using a 2.1 × 30 mm 1.7 μm RP-C_18_ column and an elution gradient of H_2_O–CH_3_CN (5:95 to 95:5, with 0.2% HCO_2_H). Analytical TLC was performed on aluminum plates precoated with silica gel 60 F254 (Merck). After development with an appropriate solvent system, the plates were evaluated under UV light (254 and 366 nm) and then sprayed with 4-anisaldehyde reagent, prepared by mixing 3.5 mL of 4-anisaldehyde with 2.5 mL of concentrated H_2_SO_4_, 4 mL of glacial HOAc, and 90 mL of CH_3_OH. The plates were then heated for the identification of UV-negative compounds and assessment of the color change of the UV-positive spots. Column chromatography was carried out using silica gel 60 (230–400 mesh), and gel filtration was performed over Sephadex LH-20 (Pharmacia) suspended in CH_2_Cl_2_–CH_3_OH (1:1). Preparative HPLC was performed on a Waters 600E system using Chromulan software (Pikron Ltd.) and an RP-C_8_ Kromasil column (250 mm × 25 mm) with a H_2_O–CH_3_OH gradient (70:30 to 100:0) for 20–40 min at a flow rate of 7 mL/min.

### Plant Material

The root (230.0 g) and stem (983.7 g) barks of *U. pandensis* were collected separately from the Mkwagulo and Mtakayo clans’ sacred forest graveyards in Fukayosi Village, Bagamoyo District, in Pwani Region, at GPS location 6°24′51.918″ S 38°40′19.308″ E altitude 78.80 m. The taxonomic identification of the plant species was performed by Mr. F. M. Mbago in the field and confirmed at the Herbarium, Botany Department of the University of Dar es Salaam, where a voucher specimen (FMM-3807) was deposited.

### Extraction and Isolation

The stem and root barks of *U. pandensis* were air-dried for 2 weeks and then powdered to obtain 230.0 and 983.7 g samples, respectively. The ground materials were then soaked in CH_3_OH for 48 h twice for each of the plant parts. The filtrates were concentrated in vacuo on a rotary evaporator at 40 °C to obtain 45 g of root bark and 23.0 g of stem bark crude extracts.

Gravity column chromatography of the root bark crude extract (45 g) was performed by adsorbing the extract on silica gel followed by gradient elution with solvent systems ranging from 5% EtOAc–isohexane to 10% EtOAc–CH_3_OH. Altogether 215 fractions of ca. 250 mL each were collected, then combined to obtain 33 fractions based on TLC analysis. Prior to combining, fraction 165 obtained with 50% EtOAc–isohexane elution precipitated and was further purified using isohexane to give zeylenol (**7**, 9.2 mg). Fraction 79, obtained with 5% EtOAc–isohexane, precipitated from CH_3_OH to give 17.6 mg of a mixture of stigmasterol (**18**) and β-sitosterol (**19**). Fraction 20 (117–131) obtained with 50% EtOAc–isohexane was subjected to passage over a Sephadex column (1:1 CH_3_OH–CH_2_Cl_2_) to afford 18 fractions, from which subsequent subfractions 1 (5–7), 3 (9–12), and 4 (15–16) gave cherrevenol I (**10**, 12.4 mg), pandensenol C (**5**, 6.7 mg), and (8′α,9′β-dihydroxy)-3-farnesylindole (**12**, 12.1 mg), respectively. Fraction 21 (132–133) obtained with 50% ethyl acetate–isohexane precipitated from EtOAc–isohexane and crystallized on standing in EtOAc to give 6-methoxyzeylenol (**6**, 18.5 mg). The soluble portion of fraction 21 (132–133) was subjected to preparative HPLC utilizing a H_2_O–CH_3_OH eluent system, affording 3-methoxybenzylbenzoate (**11**, 5.2 mg) and (6′,7′-dihydro-8′α,9′α-dihydroxy)-3-farnesylindole (**13**, 8.7 mg), at 9.45 and 12.46 min retention times, respectively. Fraction 25 (155–167) obtained with 50% EtOAc–isohexane was subjected to Sephadex column separation utilizing CH_3_OH–CH_2_Cl_2_ (1:1) as eluent system to afford seven fractions (ca. 2 mL each). Subfraction 2 contained pandensenol A (**3**, 9.2 mg), while the rest of the subfractions contained complex mixtures of inseparable compounds. Fraction 25 (142–145) was subjected to passage over a Sephadex column eluting with CH_3_OH–CH_2_Cl_2_ (1:1) to afford 12 subfractions of ca. 2 mL each. Subfractions 5–8 were then subjected to HPLC utilizing a H_2_O–CH_3_OH eluent system to afford cleistenediol C (5.5 mg, **8**) and cleistenediol F (3.6 mg, **9**) with retention times of 9.88 and 13.43 min, respectively.

The stem bark crude extract (23.0 g) was adsorbed on silica gel and subjected to gravity column chromatography employing gradient elution ranging from 5% EtOAc–isohexane to 10% CH_3_OH–EtOAc, to afford 356 fractions (ca. 200 mL each). Based on TLC analysis, the fractions were combined to obtain 69 fractions, of which fraction 44 (206–208), eluted with 30–50% EtOAc–isohexane, was purified further using reversed-phase HPLC, which afforded a mixture (10.3 mg) of uvaretin (**20**) and isouvaretin (**21**) at a retention time of 13.53 min. Fraction 54 (274–278), obtained with 50–70% EtOAc–isohexane, precipitated in CH_3_OH to give benzoic acid 2,3-diacetoxy-1,6-dihydroxycyclohex-4-enyl methyl ester (**15**, 6.4 mg). Fraction 59 (291–294) precipitated in EtOAc to furnish 11.5 mg of lupeol (**16**). Fraction 47 (246–253), obtained with 50–70% EtOAc–isohexane, was subjected to gel filtration using a Sephadex column (1:1 CH_3_OH–CH_2_Cl_2_) to give 30 subfractions that on TLC analysis were combined to obtain a further nine subfractions. Subfraction 4 (19–20) was subjected to reversed-phase HPLC for further purification, from which at a retention time of 14.29 min zeylenyl-2,6-diacetate (**14**, 5.2 mg) was collected. Combined fraction 66 (319–327), obtained with 50–70% EtOAc–isohexane, was subjected to Sephadex column (1:1 CH_3_OH–CH_2_Cl_2_) purification and then reversed-phase HPLC utilizing CH_3_OH–H_2_O to give pandensenol A (**3**, 3.6 mg) at a retention time of 11.30 min. Fraction 69 (351–356), obtained with 50–70% EtOAc–isohexane, was subjected to separation over a Sephadex column (1:1 CH_3_OH–CH_2_Cl_2_), whereby 35 subfractions of ca. 1 mL each were collected, which were then combined to obtain 13 subfractions upon TLC analysis. Subfraction 4 precipitated in CH_2_Cl_2_ to give betulin (**17**, 3.4 mg). Subfraction 12 was subjected to reversed-phase HPLC, affording pandensenol B (**4**, 4.2 mg), dipandensin A (**1**, 4.2 mg), and dipandensin B (**2**, 3.4 mg) at retention times of 9.48, 11.32, and 14.56 min, respectively. The remaining subfractions resulted into either reisolation of the compounds or inseparable mixtures.

#### Dipandensin A (**1**)

White solid; [α]^24^_D_ −33 (*c* 0.2, CH_3_OH); UV (CH_3_OH) λ_max_ (log ε) 273 (3.40), 230 (2.37), 205 (3.24) nm; IR ν_max_ 3520, 2973, 2940, 2915, 2824, 2325, 2120, 1740, 1722, 1635, 1471, 1453, 1369, 1315, 1270, 1230, 1216 cm^–1^; ^1^H and ^13^C NMR data, see Table 1; HRESIMS *m*/*z* 622.2652 [M + NH_4_]^+^ (calcd for C_34_H_40_NO_10_, 622.2442).

#### Dipandensin B (**2**)

White solid; [α]^24^_D_ −40 (*c* 0.2, CH_3_OH); UV (CH_3_OH) λ_max_ (log ε) 274 (3.43), 230 (4.27), 205 (4.24) nm; IR ν_max_ 3508, 2949, 2947, 2833, 2262, 1386, 1199, 1026, 833 cm^–1^; ^1^H and ^13^C NMR data, see Table 2; HRESIMS *m*/*z* 621.2297 [M + H]^+^ (calcd for C_34_H_37_O_11_, 621.2336).

#### Pandensenol A (**3**)

Colorless oil; [α]^24^_D_ −173 (*c* 0.2, CH_3_OH); UV (CH_3_OH) λ_max_ (log ε) 229 (2.33), 206 (3.44) nm; [α]^24^_D_ −173 (*c* 0.2, CH_3_OH); IR ν_max_ 3330, 2970, 2944, 1737, 1634, 1601, 1561, 1413, 1316, 1229, 1217, 1032 cm^–1^; ^1^H and ^13^C NMR data, see Table 3; HRESIMS *m*/*z* 281.1025 [M + H]^+^ (calcd for C_14_H_17_O_6_, 281.1025).

#### Pandensenol B (**4**)

White solid; [α]^24^_D_ +85 (*c* 0.3, CH_2_Cl_2_); UV (CH_3_OH) λ_max_ (log ε) 230 (2.48) nm; IR ν_max_ 3318, 2943, 2832, 1678, 1451, 1402, 1274, 1112, 1023 cm^–1^; ^1^H and ^13^C NMR data, see Table 4; HRESIMS *m*/*z* 323.1191 [M + H]^+^ (calcd for C_16_H_19_O_7_ 323.1131).

#### Pandensenol C (**5**)

White solid; [α]^24^_D_ +81 (*c* 0.2, CH_3_OH); UV (CH_3_OH) λ_max_ (log ε) 273 (2.04), 228 (3.01), 206 (3.37) nm; IR ν_max_ 3439, 2968, 2926, 1718, 1601, 1584, 1451, 1367, 1314, 1271, 1176, 11069, 1111, 1026, 916, 809, 687, 711 cm^–1^; ^1^H and ^13^C NMR data, see Table 5; HRESIMS *m*/*z* 275.1674 [M + H]^+^ (calcd for C_17_H_23_O_3,_ 275.1647).

### X-ray Diffraction Analysis of 6-Methoxyzeylenol (**6**)

The solid state structure of **6** was determined from single crystals of **6**, obtained by crystallization from EtOAc. Data were collected on a Bruker D8 APEX-II equipped with a CCD camera using Mo Kα radiation (λ = 0.710 73 Å). Crystals were mounted on a fiber loop and fixated using Fomblin oil. Data reduction was performed with SAINT,^46^ and absorption corrections for the area detector were performed using SADABS.^47^ The structure was solved in the orthorhombic space group *P*2_1_2_1_2_1_ by direct methods and refined by least-squares methods on *F*^2^ using the SHELX and the OLEX2 software suits.^48−50^ The data were collected at 150(2) K. Non-hydrogen atoms were refined anisotropically. Hydrogen atoms were constrained in geometrical positions relative to their parent atoms. A Flack parameter of 0.2(8) precluded determination of the absolute structure based on anomalous dispersion.^51,52^ Further details of structure solutions and refinements can be found in the Supporting Information. The X-ray structure data of **6** (CCDC 2105244) have been deposited with the Cambridge Crystallographic Data Centre. Copies of the data can be obtained, free of charge, on application to the Director, CCDC, 12 Union Road, Cambridge CB2 1EZ, UK (fax: + 44-(0)1223-336033 or e-mail: deposit@ccdc.cam.ac.uk).

### Antibacterial Assays

The antibacterial activity of the isolated compounds was determined against two Gram-positive bacteria, *Bacillus subtilis* (NBRC/ATCC #111470) and *Staphylococcus epidermidis* (ATCC #35984) and five Gram-negative bacteria, *Escherichia coli* MG1655 (CGSC #6300), *Paraburkholderia caledonica* (NBRC/ATCC #102488), *Pseudomonas putida* (NBRC/ATCC #100650), *Pectobacterium carotovorum* (NBRC/ATCC #3380), and *Enterococcus raffinosus* (NBRC/ATCC #100492). The bacteria were cultured as previously described by Mueller and Hinton, and Doyle. Initially, the compounds were dissolved at 10 mg/mL in 100% DMSO, then further diluted 30× in H_2_O and stored at −20 °C. For in vitro determination of antibacterial activity, a culture of bacterial cells was grown to OD 600_nm_ = 0.5. The culture was diluted 10× with prewarmed medium, and the compounds to be tested were added to the culture medium for a final concentration of 30 μg/mL, each at 100 μL in a 96-well microtiter plate, then incubated at 37 °C without agitation for 18 h. To measure cell viability, the resazurin-based assay was used, as described previously. To each well was added 12 μL of 10× alamarBlue solution (resazurin solution, ThermoFisher), and the plate incubated at 37 °C for 1 h. Next, the fluorescence was measured using a POLARstar Omega microplate-reader from BMG Labtech with the excitation filter set to 544 nm and the emission filter at 590 nm. Cells exposed to an equivalent concentration of DMSO were used as a negative control. Bleed-through of fluorescence from resorufin between wells in the microtiter plate fluorescence reader was measured and found to be <1% between adjacent wells. To check for quenching of fluorescence by any of the investigated compounds, grown bacterial cultures were mixed after 1 h of incubation with resazurin and the compound of interest at the highest concentration to be assayed, and the measured fluorescence was compared with samples without compound added. All tests of compound activity were performed in three independent replicates. Those compounds where a reduction of fluorescence by at least 50% relative to the solvent control was observed in any of the bacterial species tested were followed up by additional tests for more accurate determination of the degree of antibacterial activity in terms of minimum inhibitory concentration (MIC). EC_50_ values, from three independent replicate experiments, using 2-fold dilution intervals were also calculated.

### Cytotoxicity Assay

The cytotoxicity levels of the isolated compounds were evaluated against human MCF-7 cells grown in Dulbecco’s modified Eagle medium supplemented with 10% fetal calf serum and kept in exponential growth as previously reported. Before the assay, cells were reseeded into 96-well microtiter plates at a density allowing continued exponential growth and allowed to settle for 24 h. The isolated compounds were added from a stock solution in DMSO, for a final concentration of 0.3% v/v of the solvent in the culture medium. After 24 h of incubation in the presence of the compound, cell viability was assayed using PrestoBlue Cell Viability Reagent (ThermoFisher) according to the manufacturer’s instructions. A Polar Star Omega plate reader (BMG Lab Tech) was used to measure resorufin fluorescence at 544 nm excitation/590 nm emissions. Survival was expressed as percentage of the solvent-only control. EC_50_ values for each compound were calculated, from three independent replicate experiments, using 2-fold dilution intervals.

## Supporting Information Available

NMR, MS, ORD, UV, and IR data for the isolated compounds (PDF)
X-ray crystallographic data of compound **6** (CIF)

## Supplementary Material

np1c00811_si_001.pdf

np1c00811_si_002.cif

## Notes

The original NMR FIDs for all compounds are freely available on Zenodo as DOI: 10.5281/zenodo.5275307.
