# Supplementary material for: Oxygenated Cyclohexene Derivatives from the Stem and Root Barks of Uvaria pandensis
Source: J Nat Prod. 2021 Nov 22;84(12):3080–9. doi: 10.1021/acs.jnatprod.1c00811 (PMC8713284; doi:10.1021/acs.jnatprod.1c00811)

SUPPORTING INFORMATION

Oxygenated Cyclohexene Derivatives from the Stem and Root Barks of *Uvaria pandensis*

Gasper Maeda, Pieter J. Gilissen, Anastasia Rudenko, Jelle van der Wal, Catarina Bourgard, Arvind Kumar Gupta, Per Sunnerhagen, Joan J. E. Munissi, Stephen S. Nyandoro, Máté Erdélyi

Table of contents

1. The structures of known compounds **6-21** S3
2. Spectroscopic data for compound **1** S4
3. Spectroscopic data for compound **2** S8
4. Spectroscopic data for compound **3** S12
5. Spectroscopic data for compound **4** S15
6. Spectroscopic data for compound **5** S20
7. Spectroscopic data for compound **6** S24
8. Spectroscopic data for compound **7** S28
9. Spectroscopic data for compound **8** S31
10. Spectroscopic data for compound **9** S35
11. Spectroscopic data for compound **10** S39
12. Spectroscopic data for compound **11** S43
13. Spectroscopic data for compound **12** S47
14. Spectroscopic data for compound **13** S51
15. Spectroscopic data for compound **14** S55
16. Spectroscopic data for compound **15** S59
17. Spectroscopic data for compound **16** S63
18. Spectroscopic data for compound **17** S66
19. Spectroscopic data for a mixture of compounds **18** and **19** S70
19. Spectroscopic data for a mixture of compounds **20** and **21** S73
20. Spectroscopic and physical data of known compounds (**6-21**) S77
21. X-ray crystallography of compound **6** S79
22. Antibacterial and cytotoxic activities S80

1. The structures of known compounds 6-21

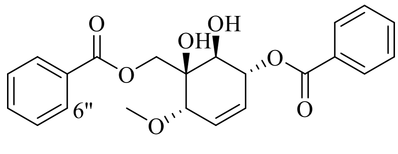

6

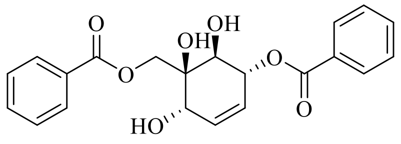

7

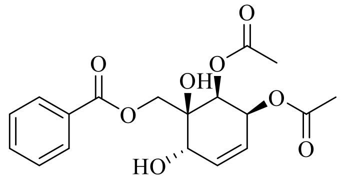

8

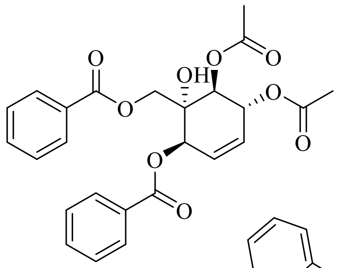

9

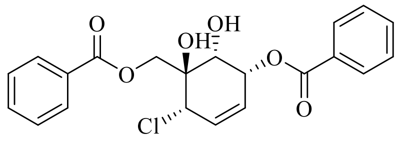

10

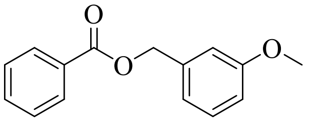

11

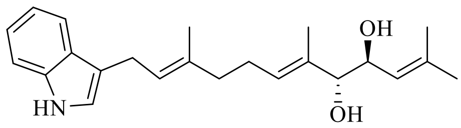

12

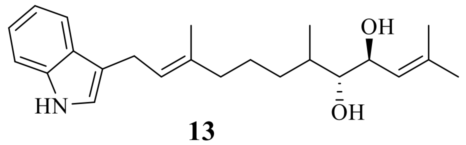

13

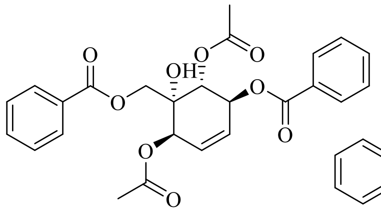

14

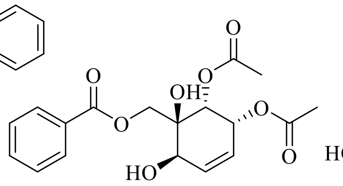

15

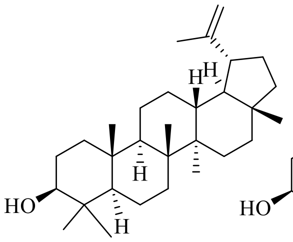

16

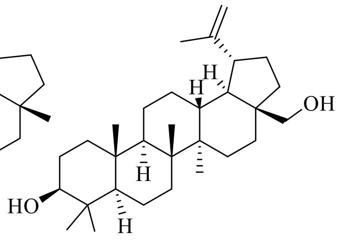

17

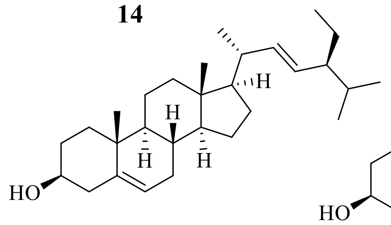

18

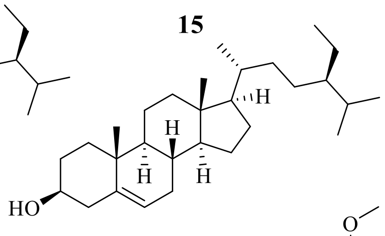

19

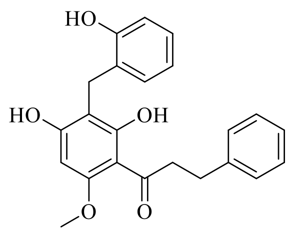

20

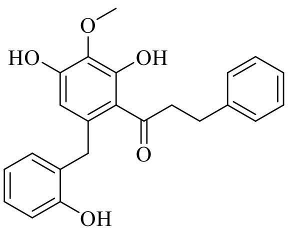

21

The original NMR FIDs for all compounds are freely available on Zenodo as DOI:10.5281/zenodo.5275307.

2. Spectroscopic data for compound 1

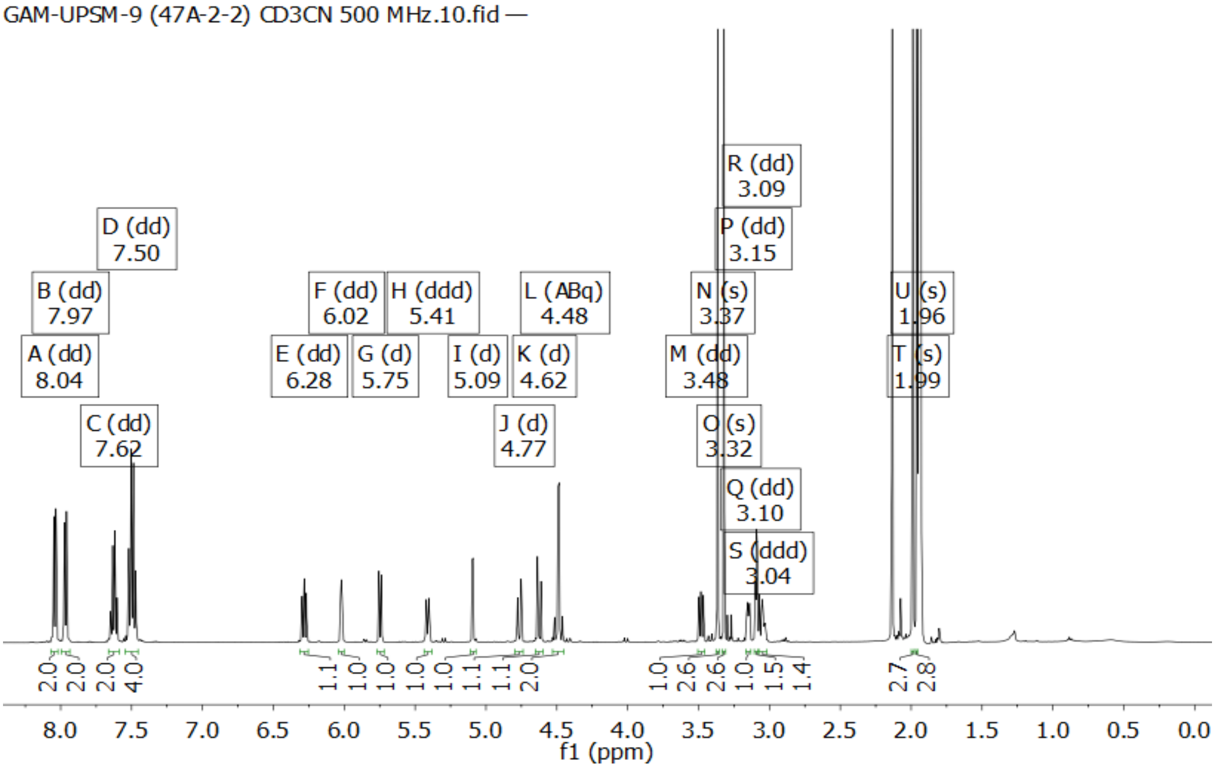

Figure S1. The ^{1}H NMR spectrum of compound **1** (500 MHz, CD_3CN)

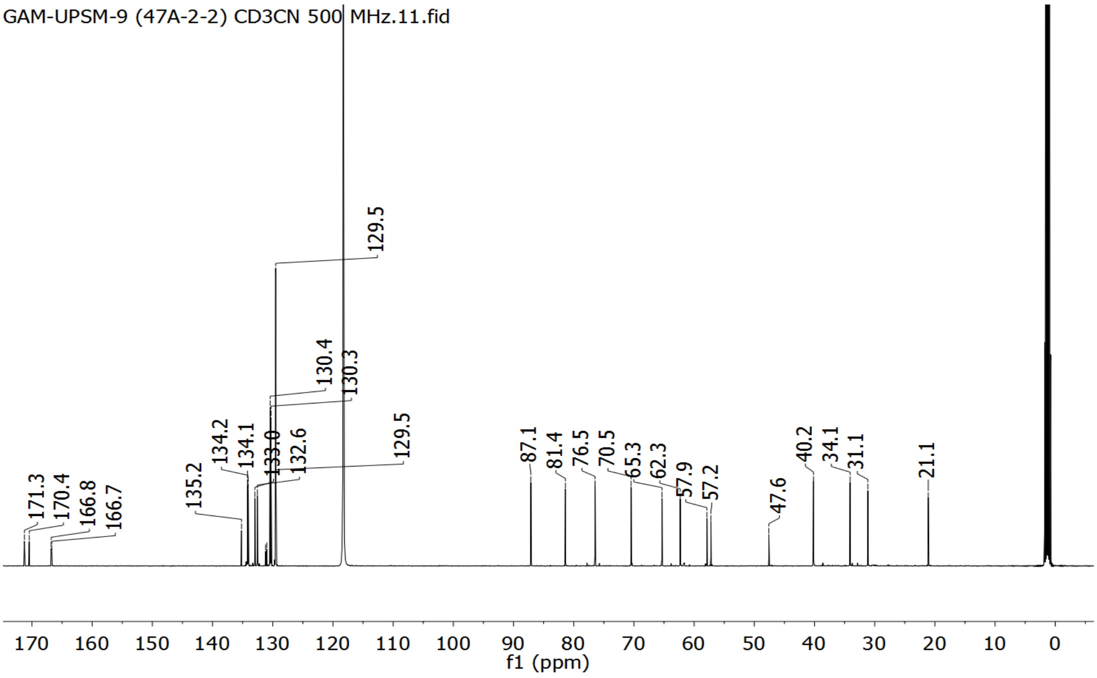

Figure S2. The ^{13}C NMR Spectrum of compound **1** (125 MHz, CD_3CN)

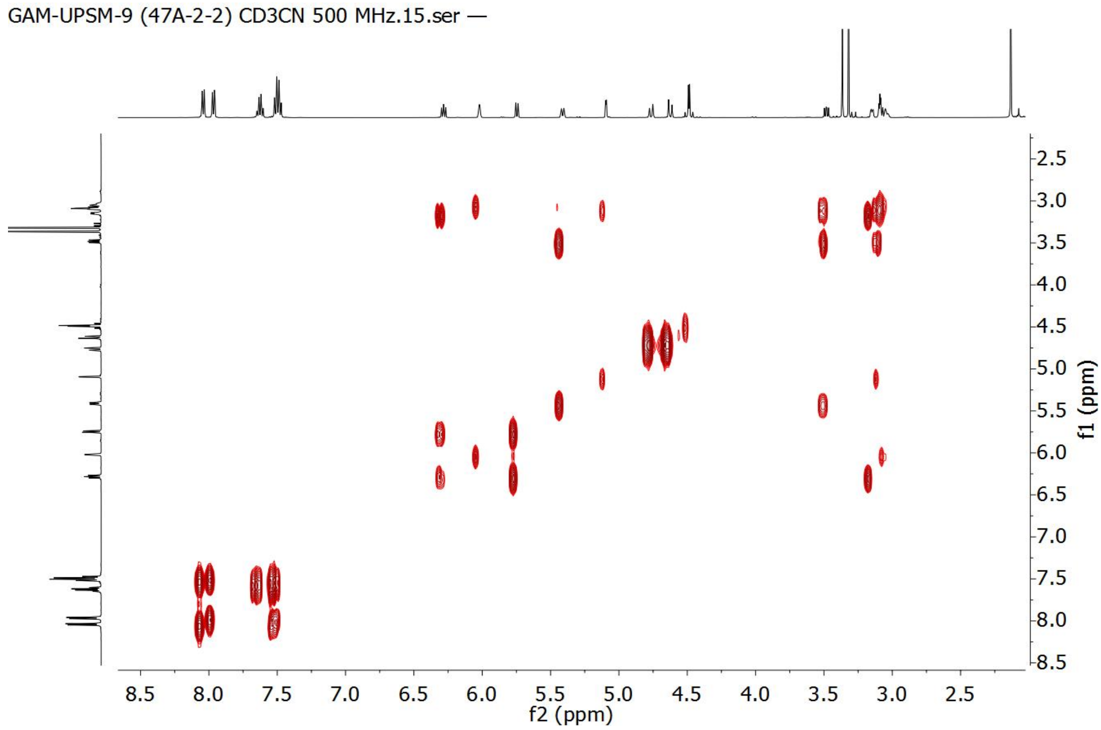

Figure S3. The COSY spectrum of compound **1** (500 MHz, CD_3CN)

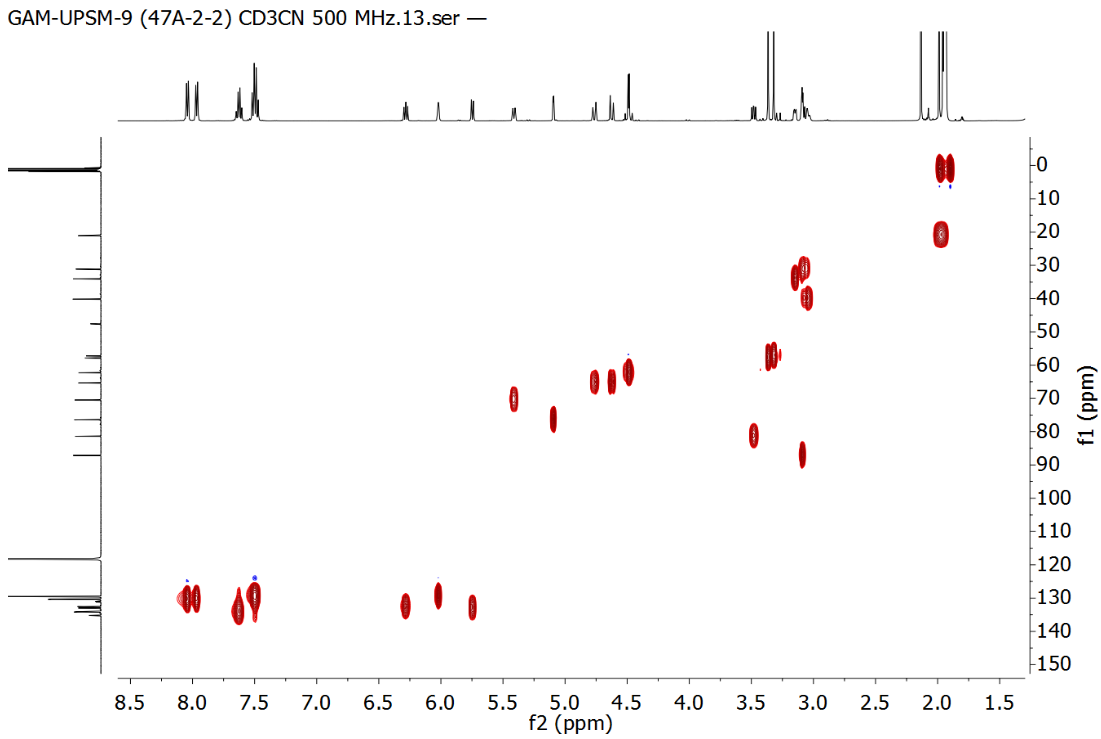

Figure S4. The HSQC spectrum of compound **1** (500/125 MHz, CD_3CN)

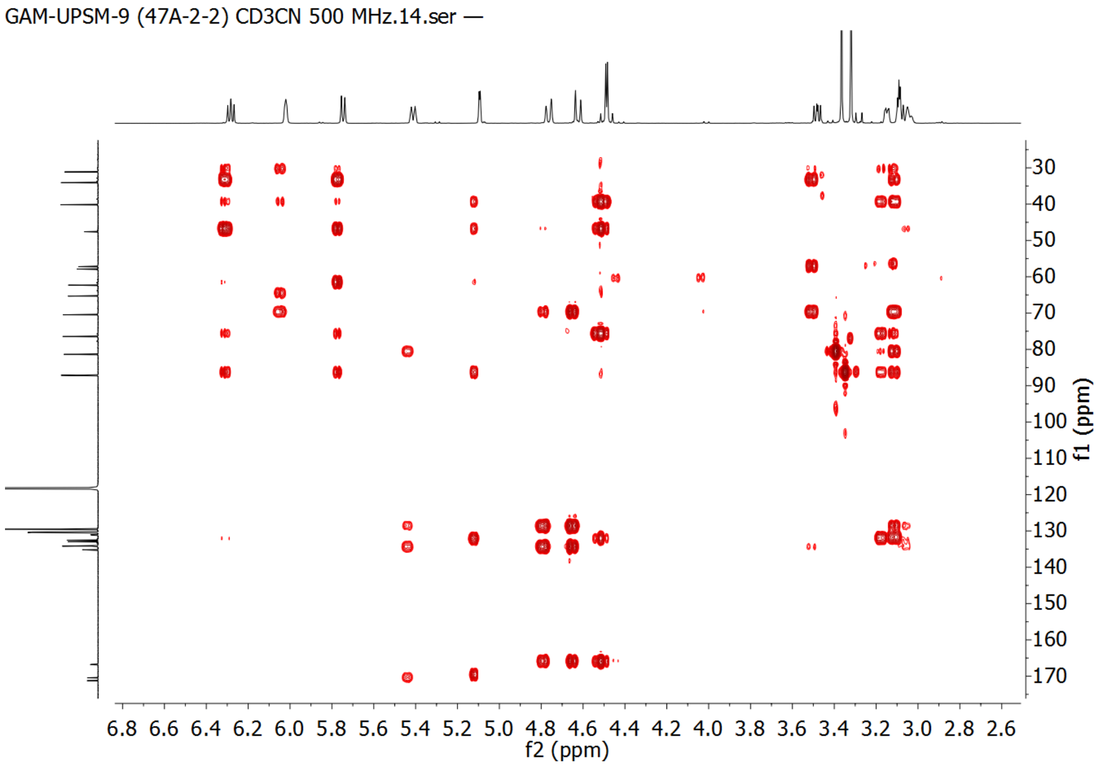

Figure S5. The HMBC spectrum of compound **1** (500/125 MHz, CD_3CN)

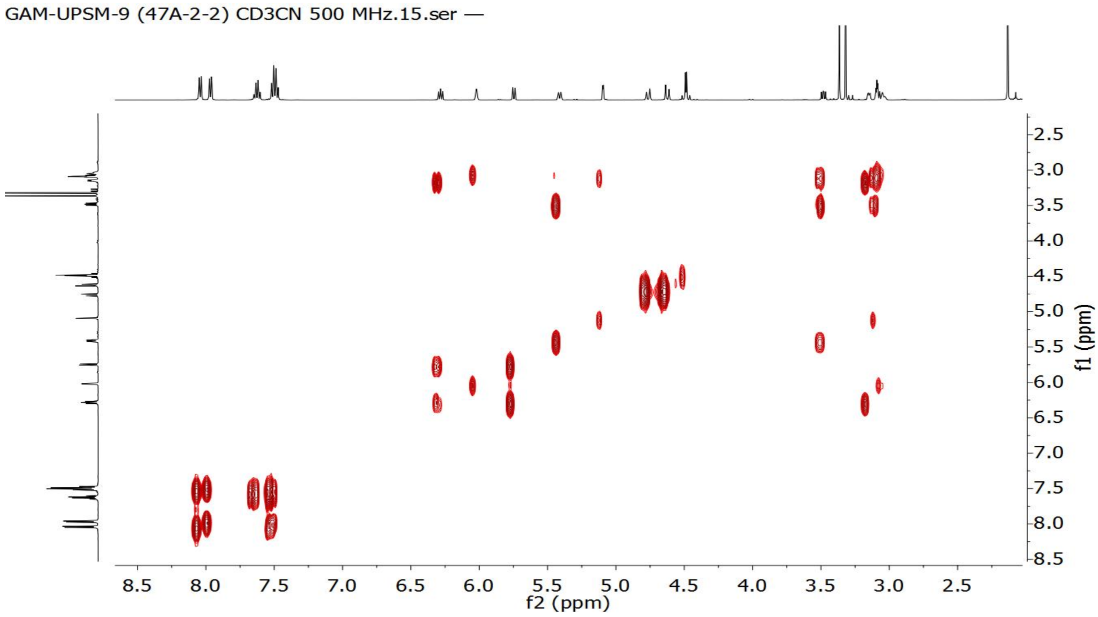

Figure S6. The TOCSY spectrum of compound **1** (500 MHz, CD_3CN)

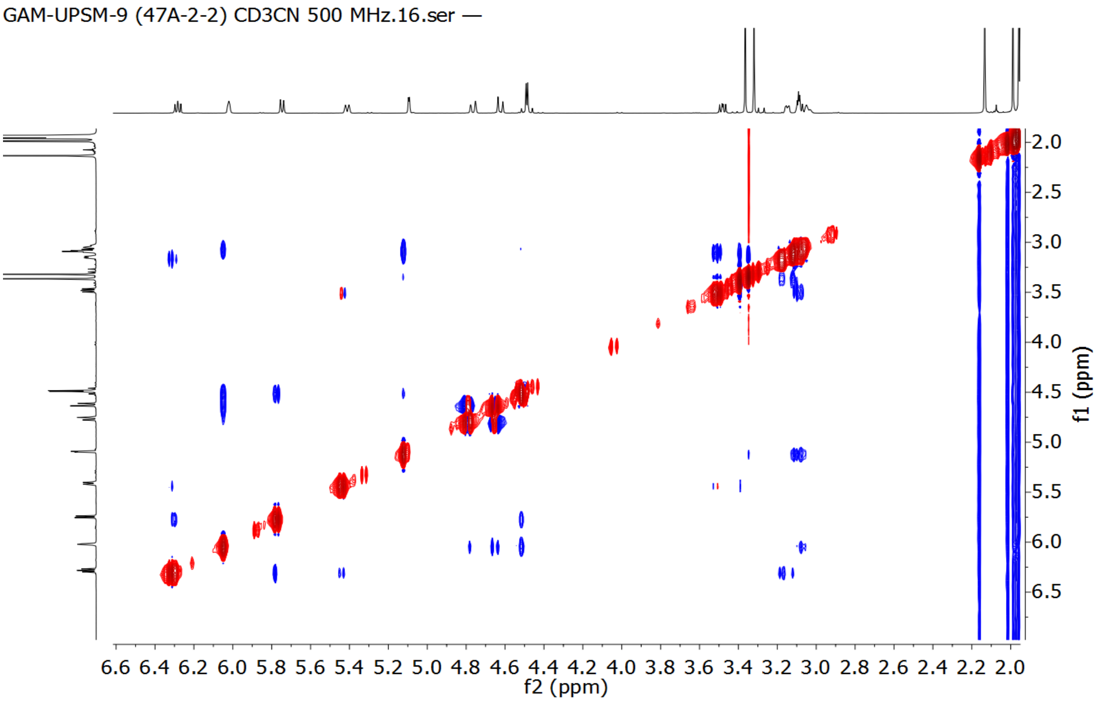

Figure S7. The NOESY spectrum of compound **1** (500 MHz, CD_3CN)

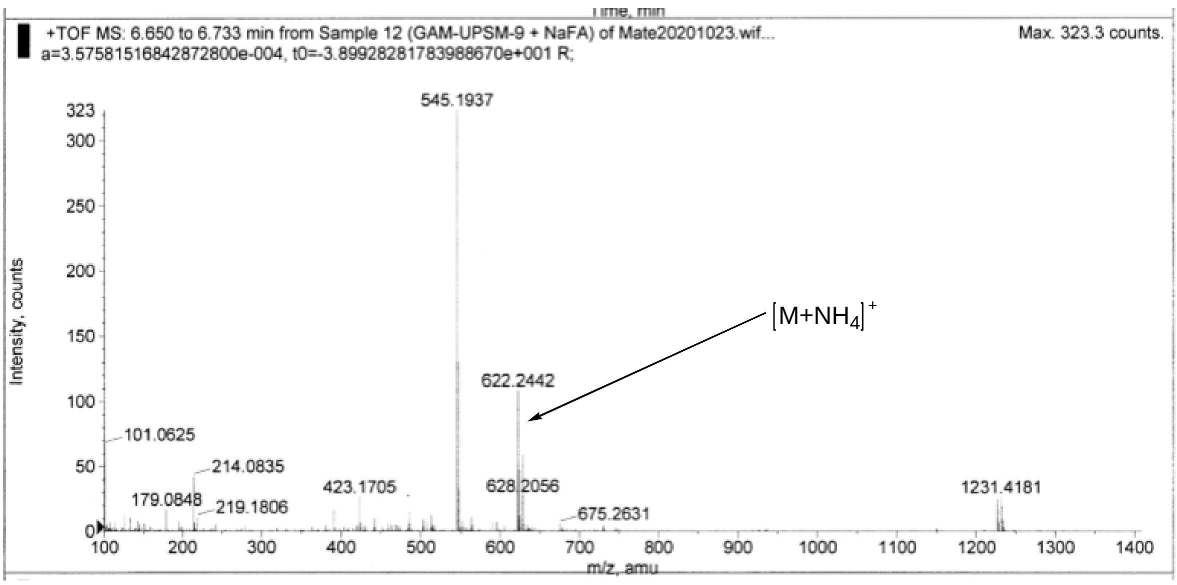

Figure S8. HRESIMS spectrum of compound **1**

3. Spectroscopic data for compound 2

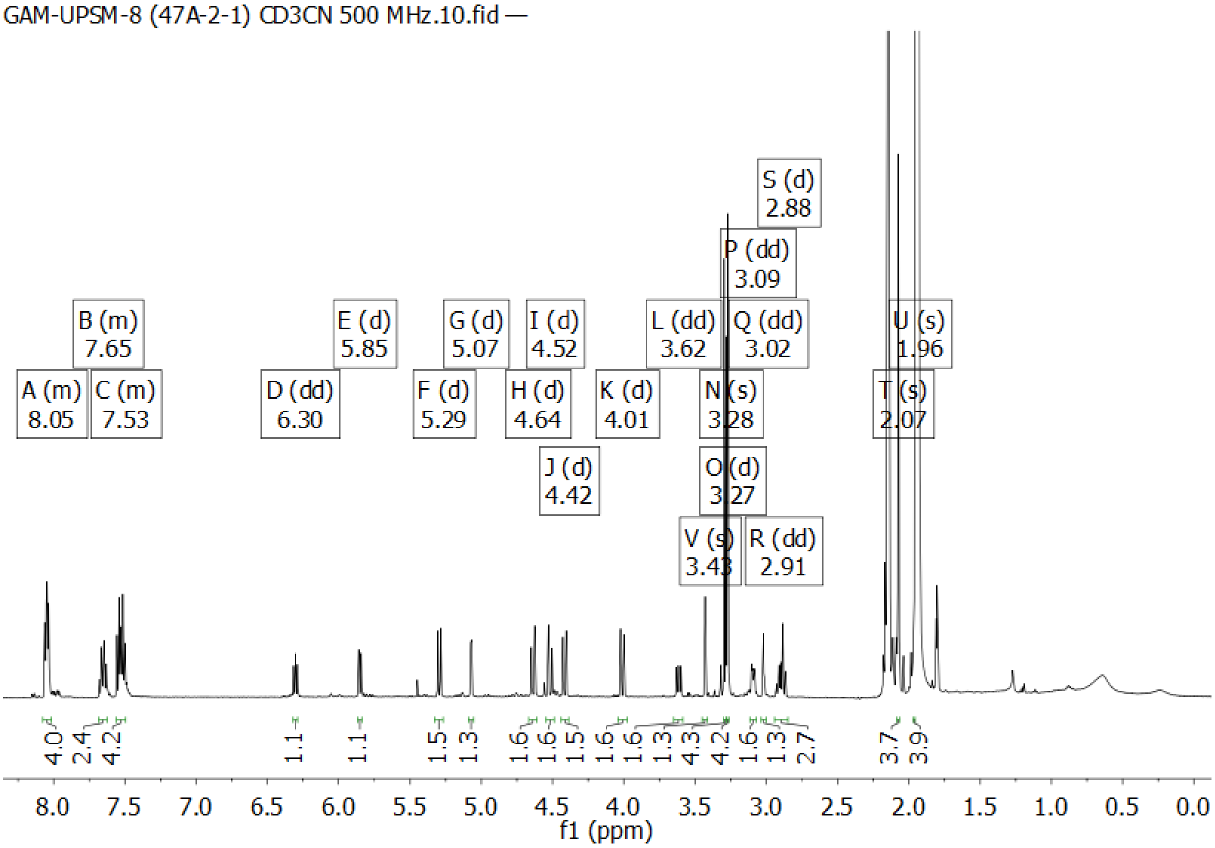

Figure S9. The ^{1}H NMR spectrum of compound **2** (500 MHz, CD_3CN)

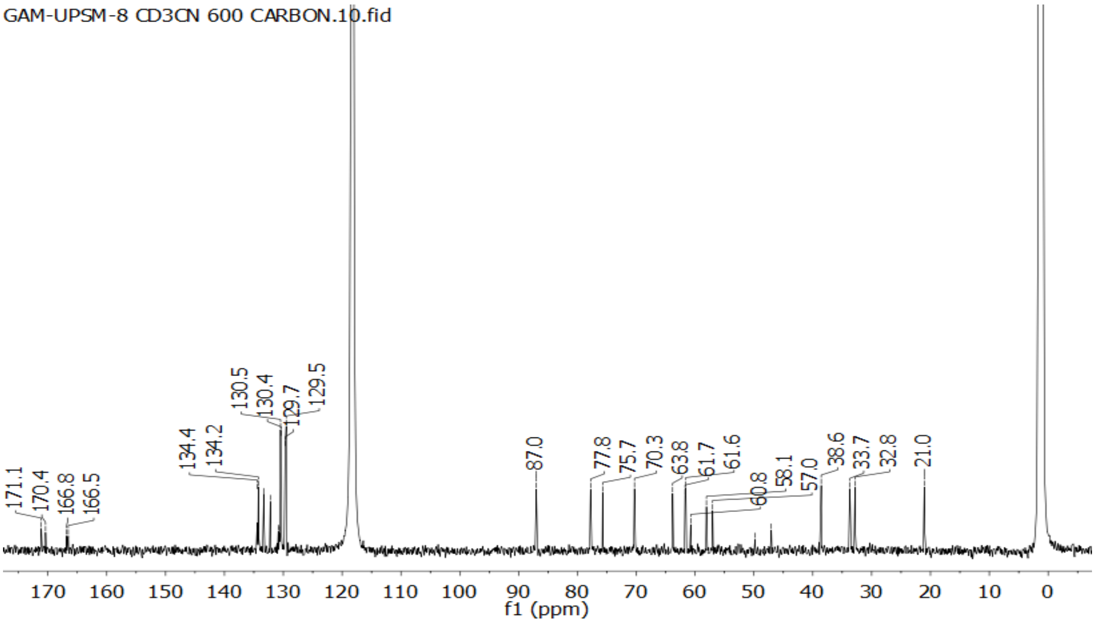

Figure S10. The ^{13}C NMR spectrum of compound **2** (125 MHz, CD_3CN)

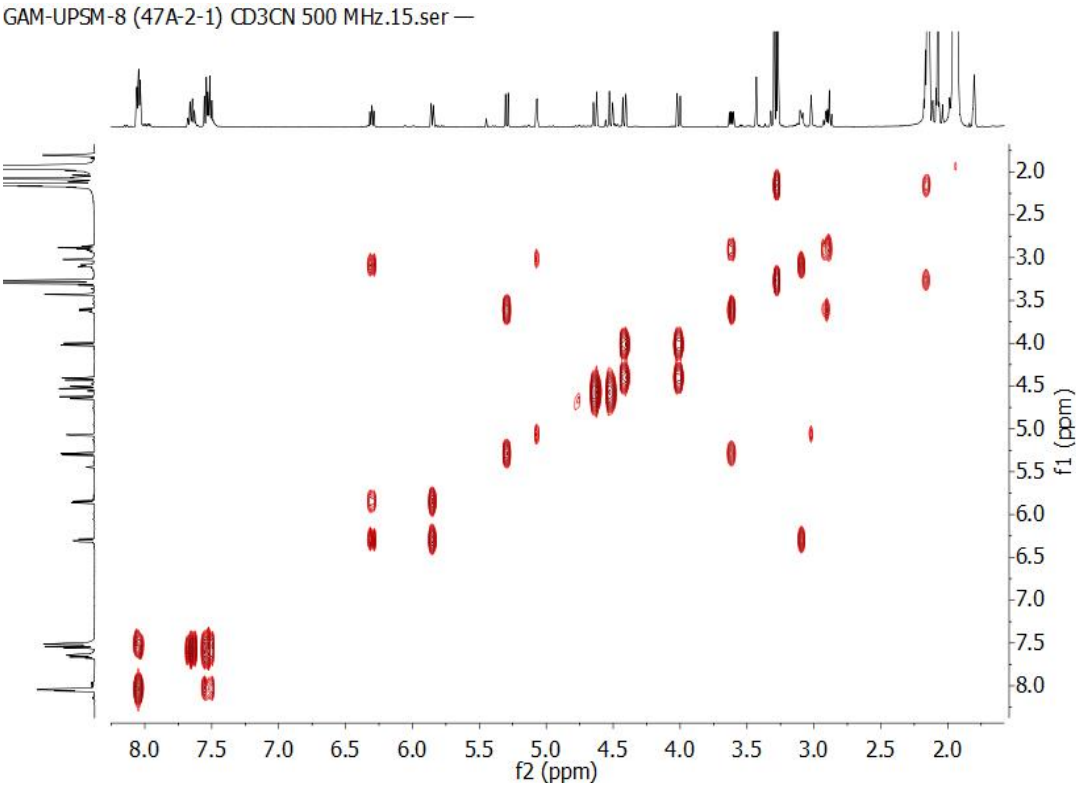

Figure S11. The COSY spectrum of compound **2** (500 MHz, CD_3CN)

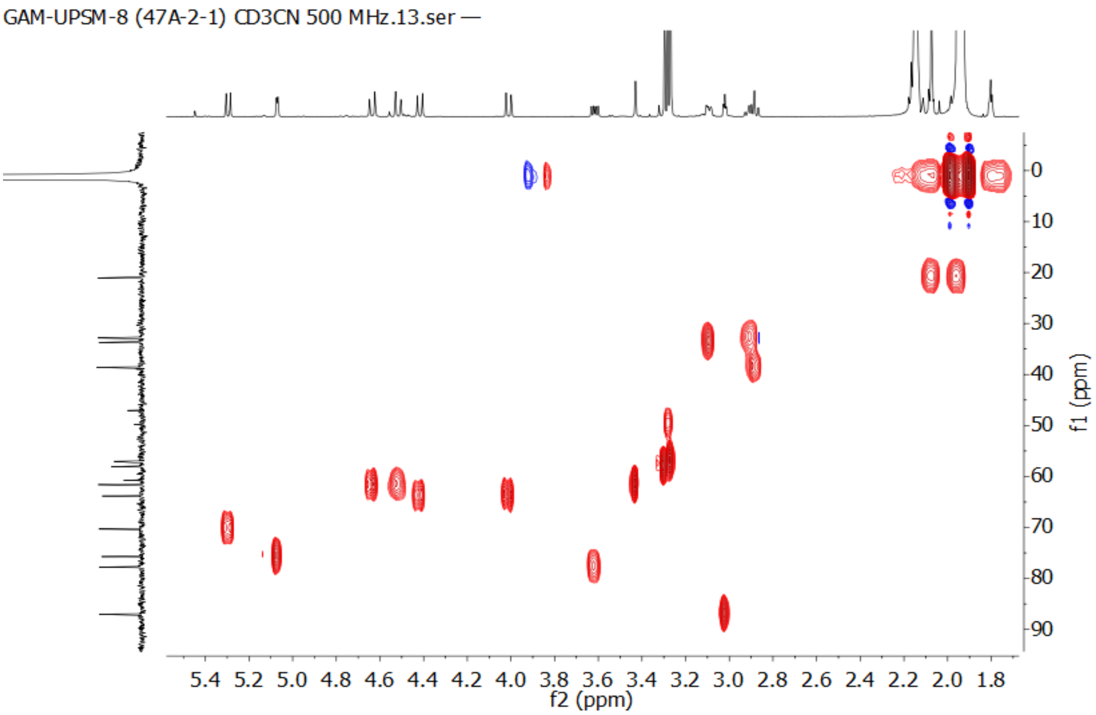

Figure S12. The HSQC spectrum of compound **2** (500/125 MHz, CD_3CN)

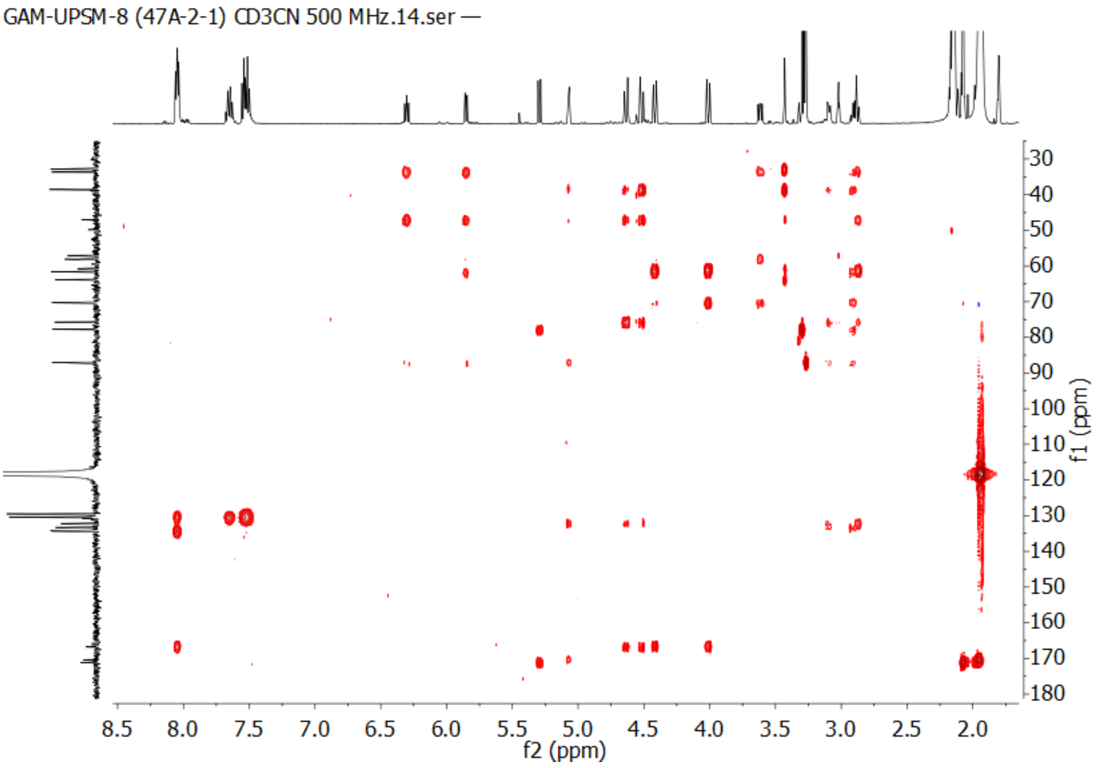

Figure S13. The HMBC spectrum of compound **2** (500/125 MHz, CD_3CN)

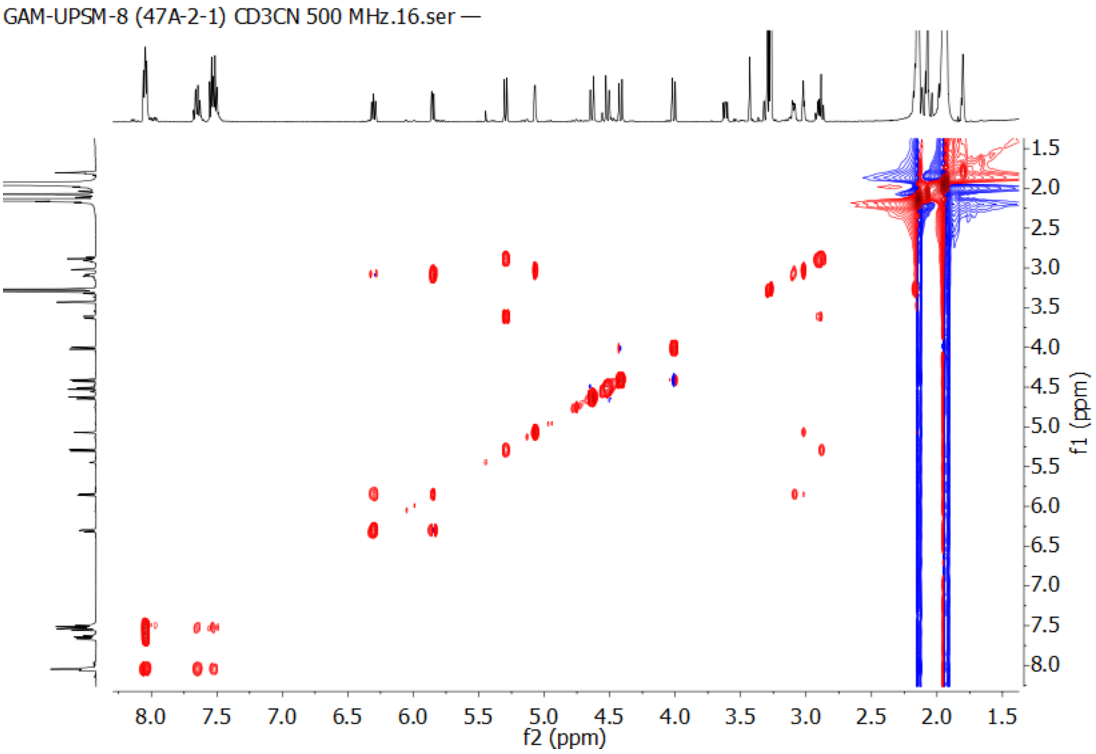

Figure S14. The TOCSY spectrum of compound **2** (500 MHz, CD_3CN)

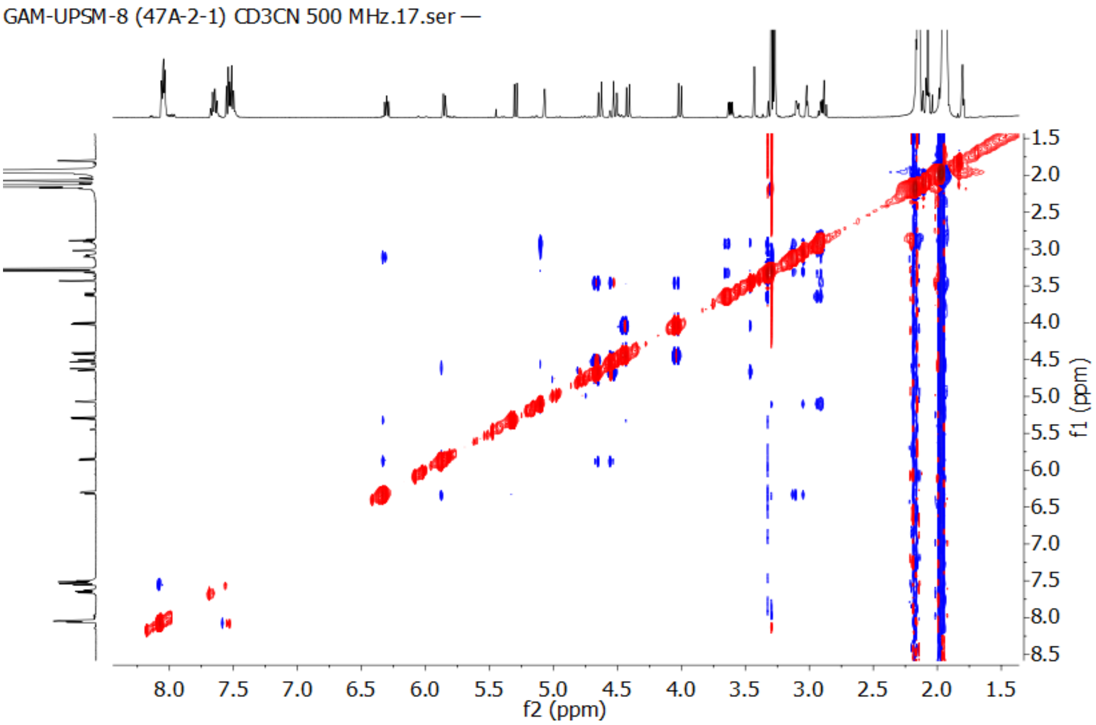

Figure S15. The NOESY spectrum of compound **2** (500 MHz, CD_3CN)

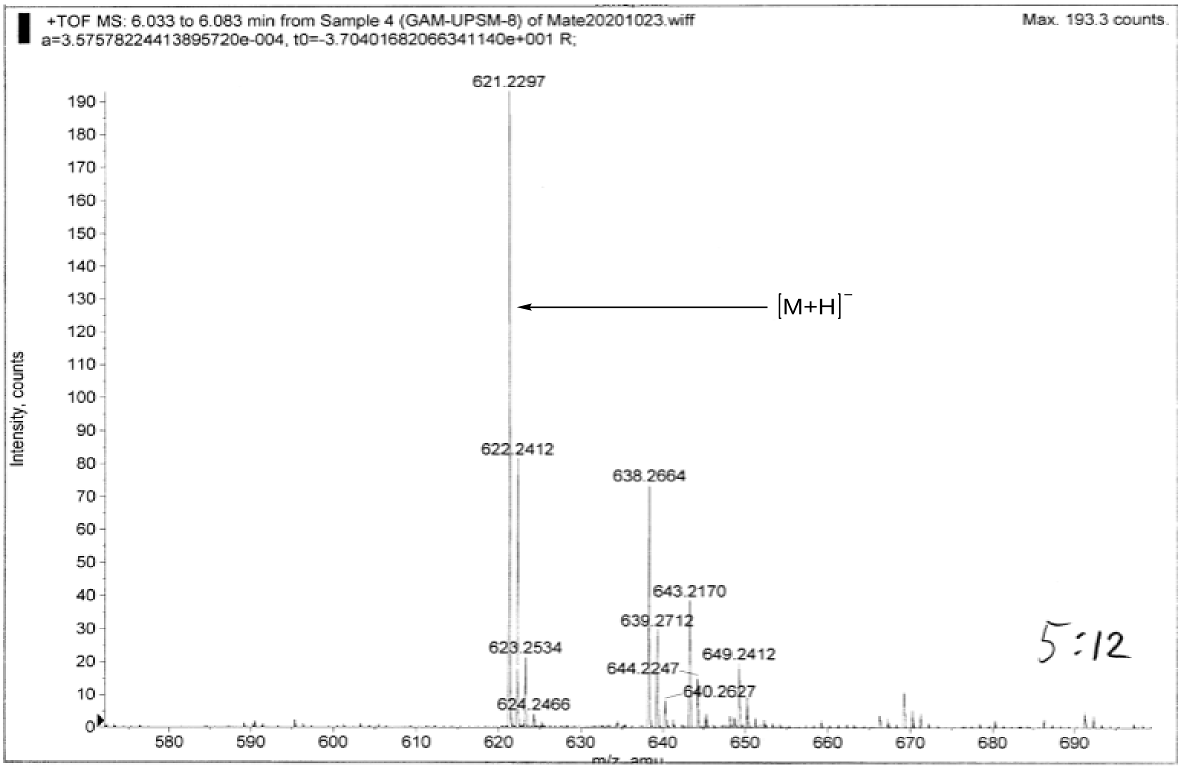

Figure S16: HRESIMS spectrum of compound **2**

4. Spectroscopic data for compound 3

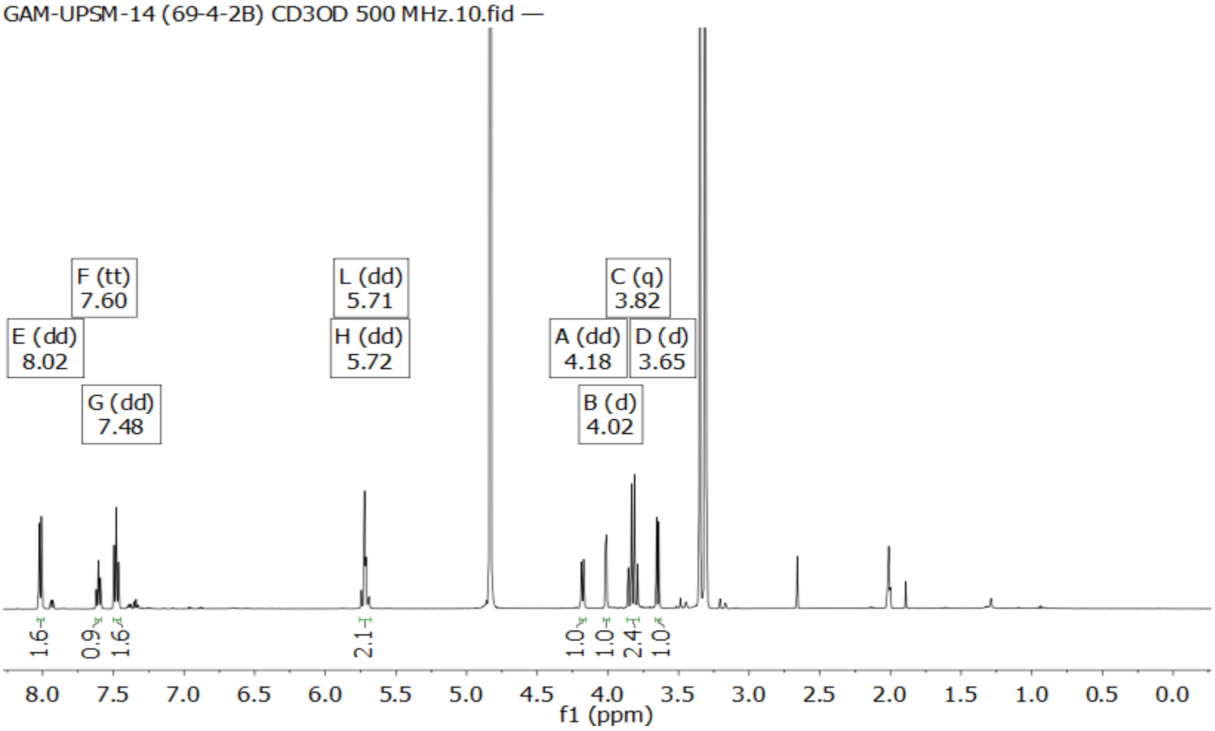

Figure S17. The ^{1}H NMR spectrum of compound **3** (500 MHz, CD_3OD)

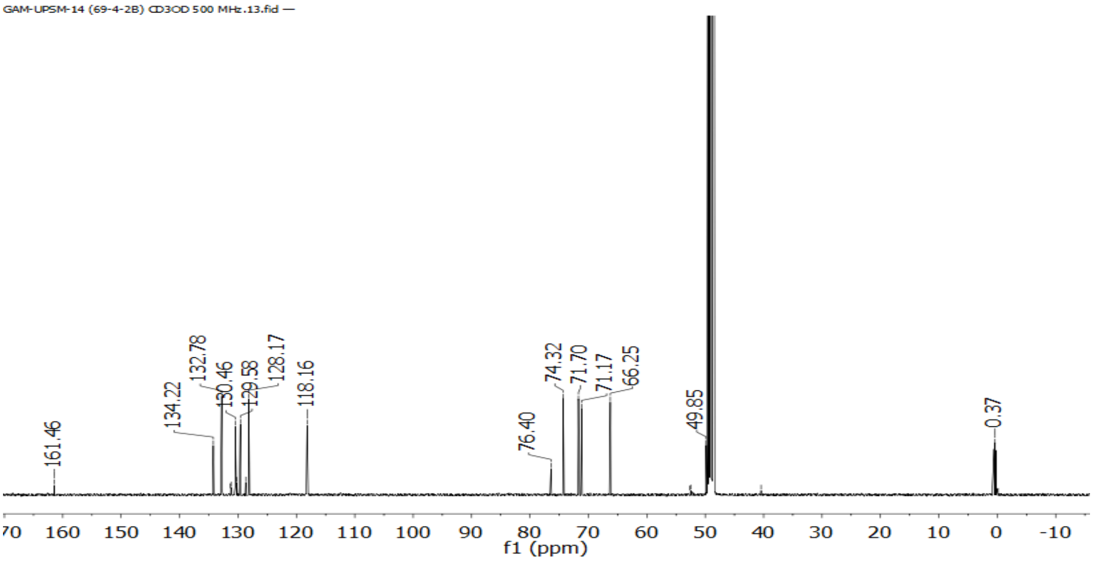

Figure S18. The ^{13}C NMR spectrum of compound **3** (125 MHz, CD_3OD)

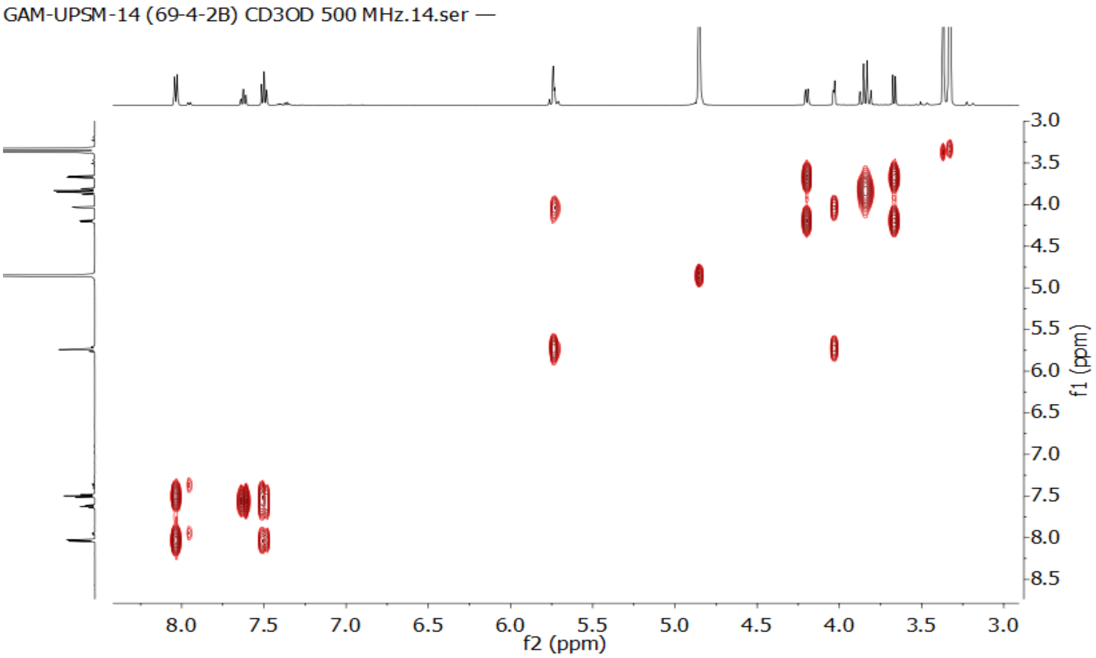

Figure S19. The COSY spectrum of compound **3** (500 MHz, CD_3OD)

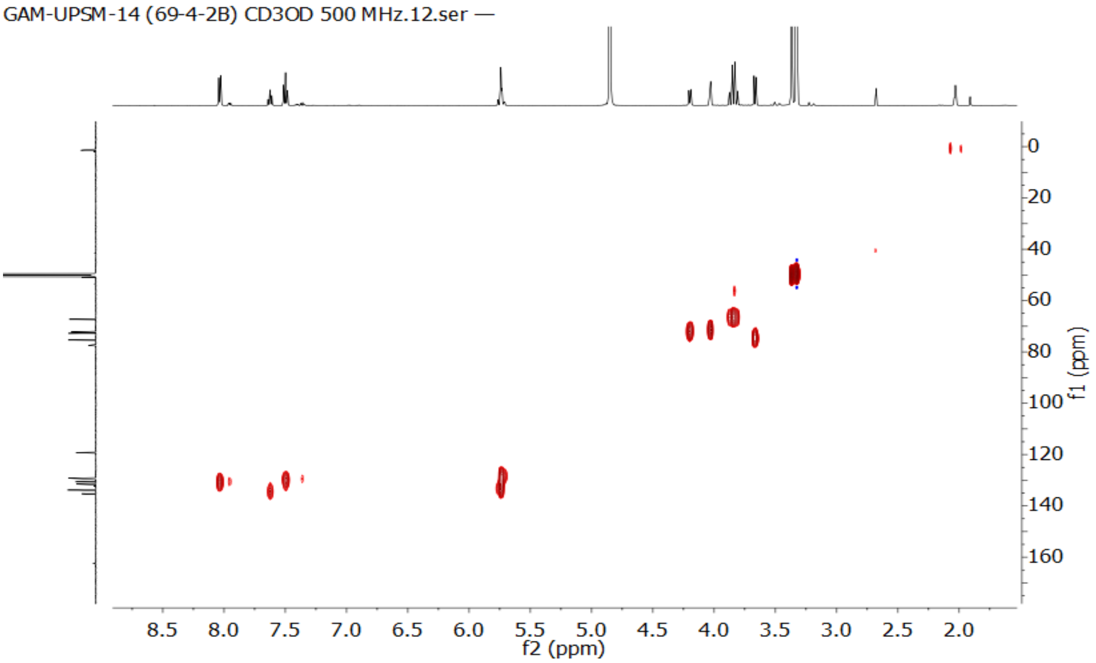

Figure S20. The HSQC spectrum of compound **3** (500/125 MHz, CD_3OD)

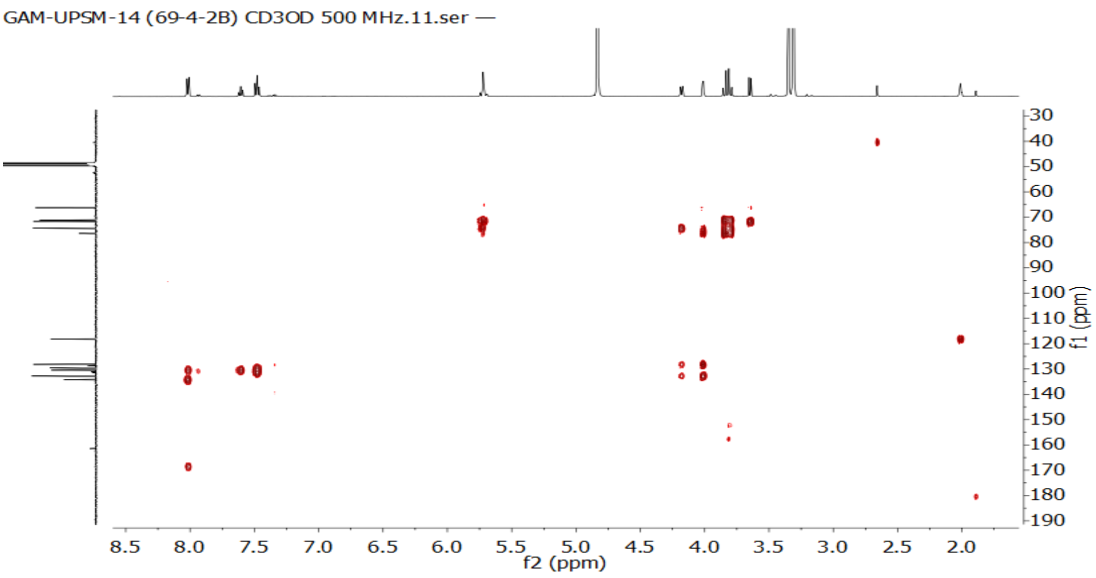

Figure S21. The HMBC spectrum of compound **3** (500/125 MHz, CD_3OD)

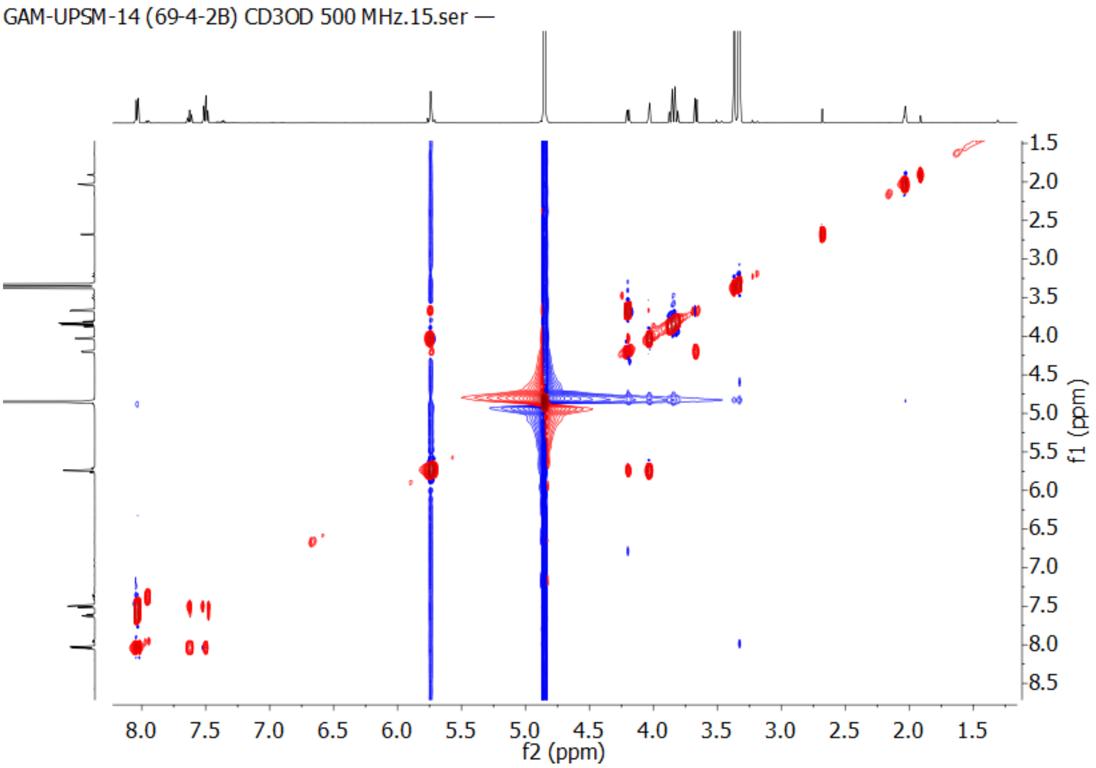

Figure S22. The TOCSY spectrum of compound **3** (500 MHz, CD_3OD)

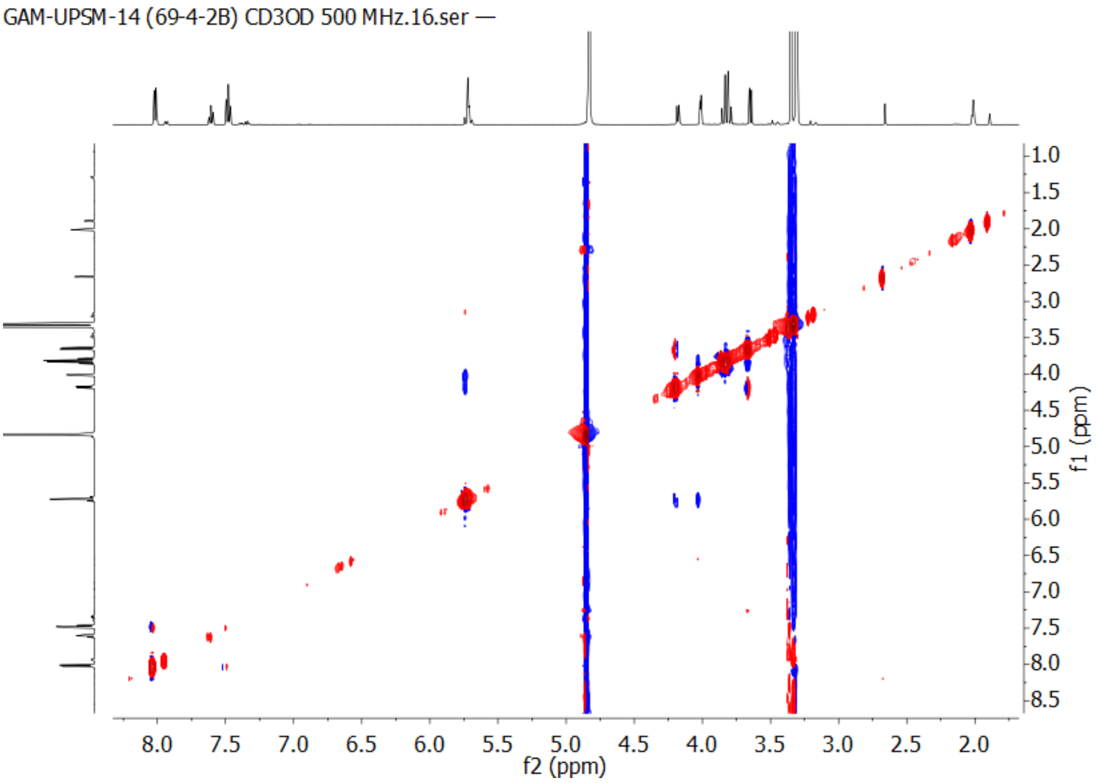

Figure S23. The NOESY spectrum of compound **3** (500 MHz, CD_3OD)

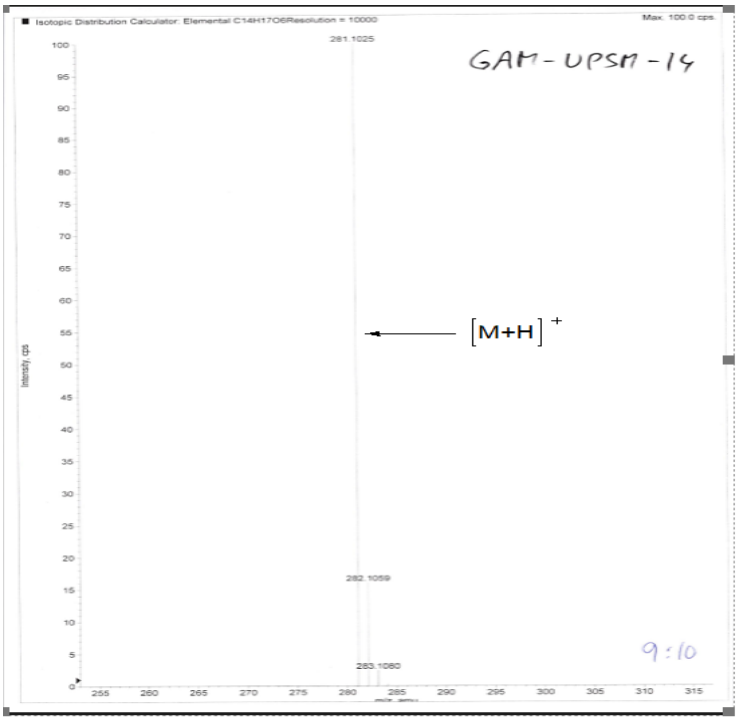

Figure S24. HRESIMS spectrum of compound **3**

5. Spectroscopic data for compound 4

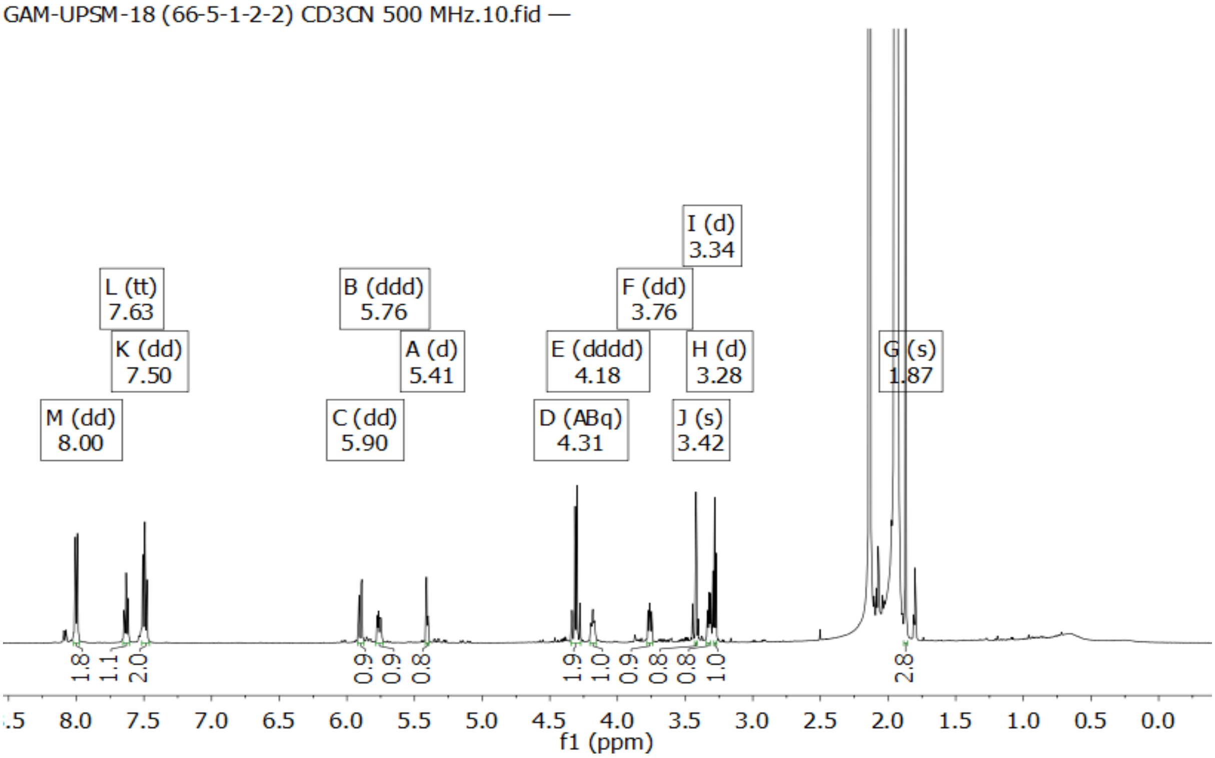

Figure S25. The ^{1}H NMR spectrum of compound **4** (500 MHz, CD_3CN)

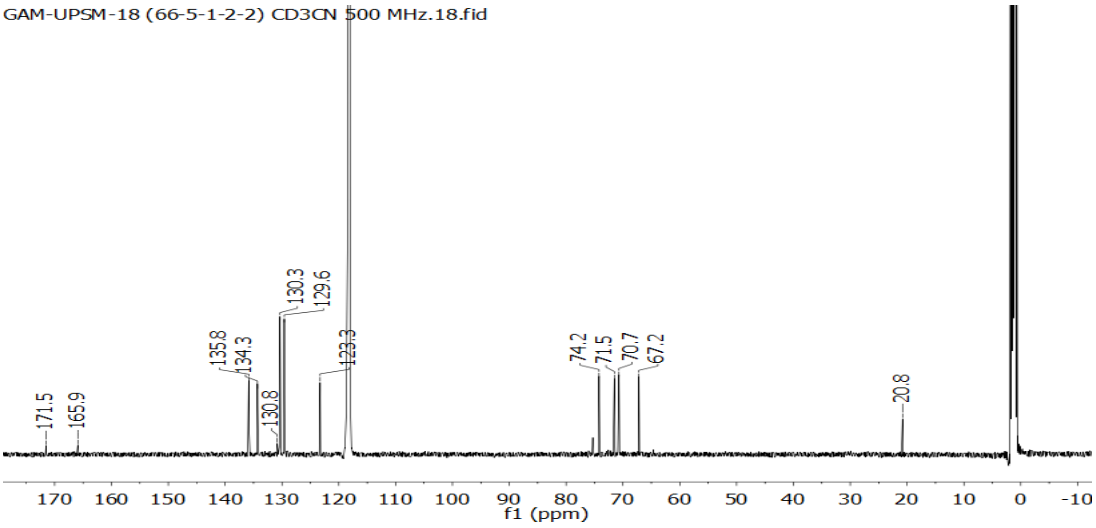

Figure S26. The ^{13}C NMR spectrum of compound **4** (125 MHz, CD_3CN)

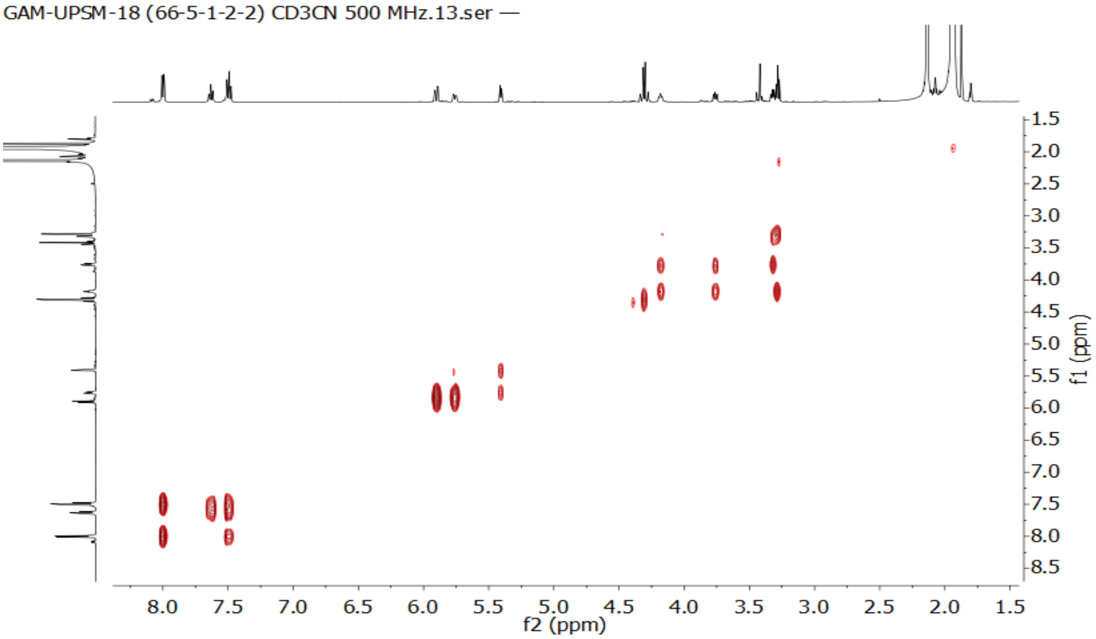

Figure S27. The COSY spectrum of compound **4** (500 MHz, CD_3CN)

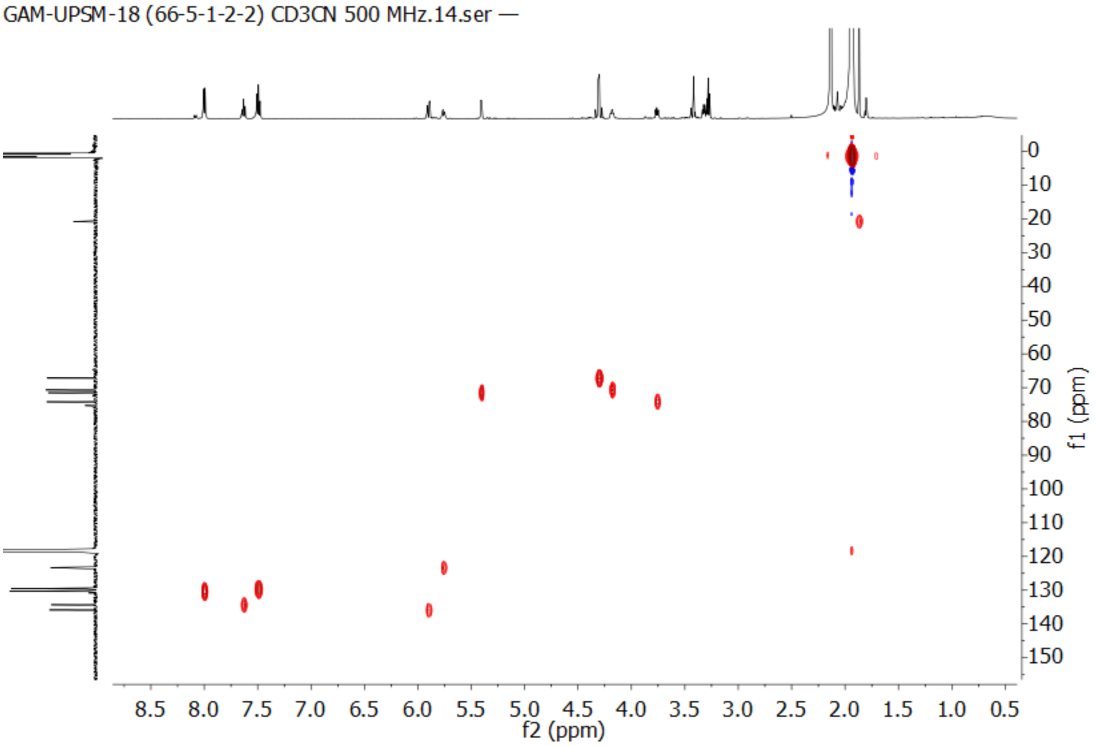

Figure S28. The HSQC spectrum of compound **4** (500/125 MHz, CD_3CN)

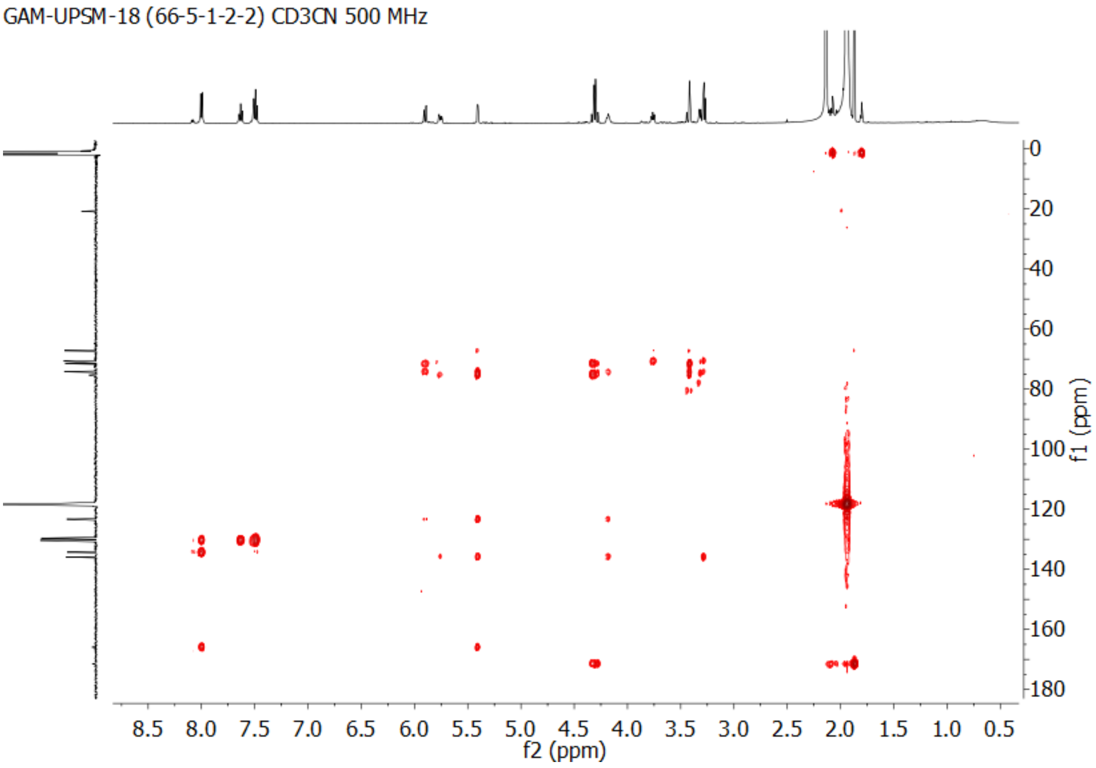

Figure S29. The HMBC spectrum of compound **4** (500/125 MHz, CD_3CN)

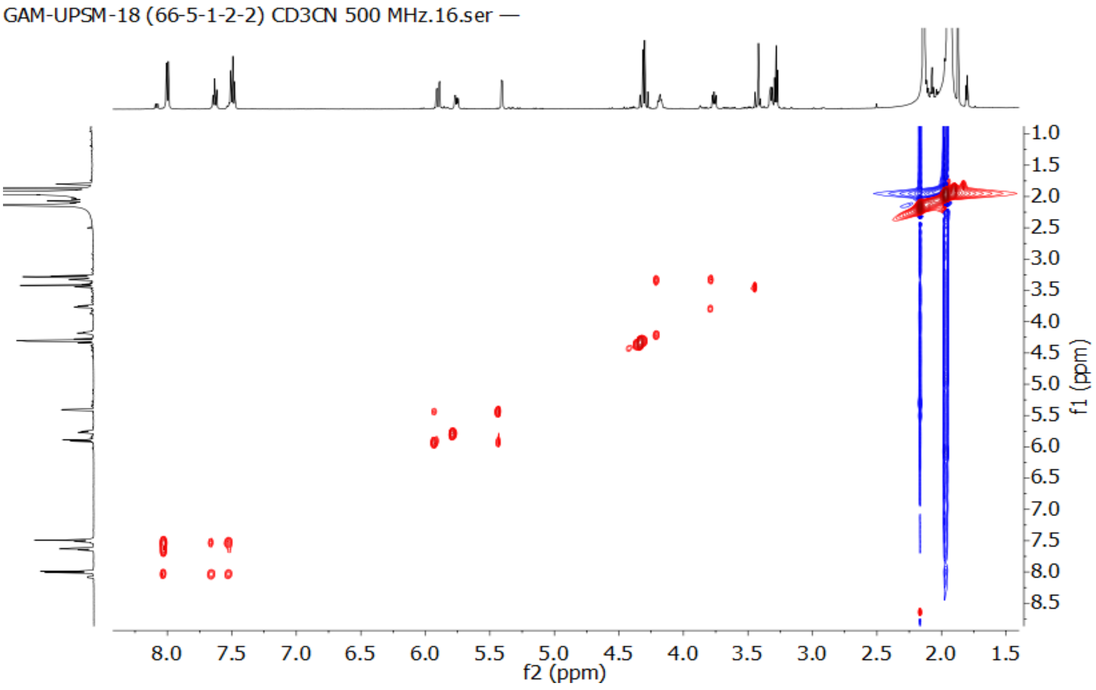

Figure S30. The TOCSY spectrum of compound **4** (500 MHz, CD_3CN)

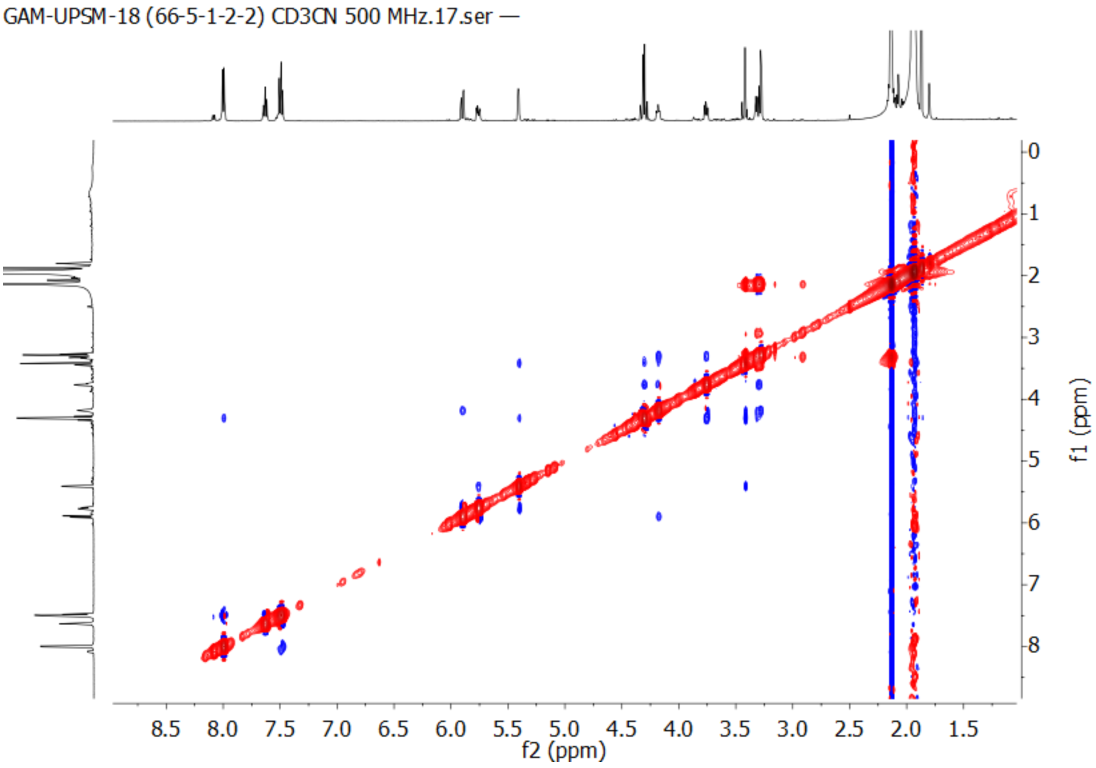

Figure S31. The NOESY spectrum of compound **4** (500 MHz, CD_3CN)

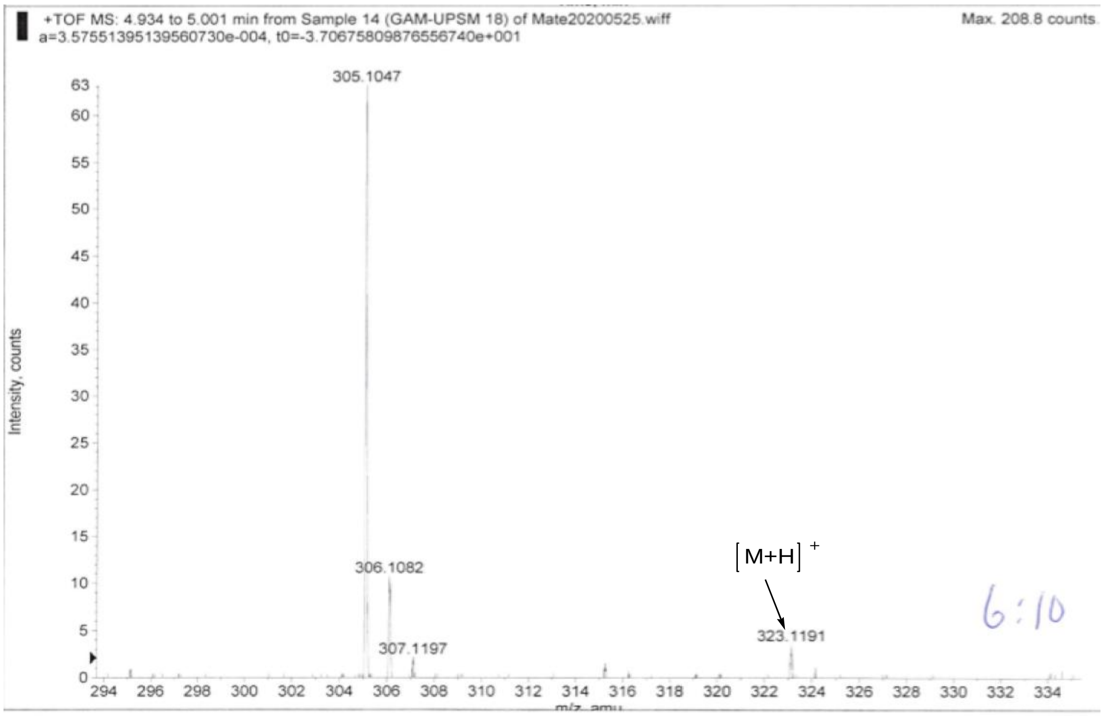

Figure S32. The HRISIMS spectrum of compound **4**

6. Spectroscopic data for compound 5

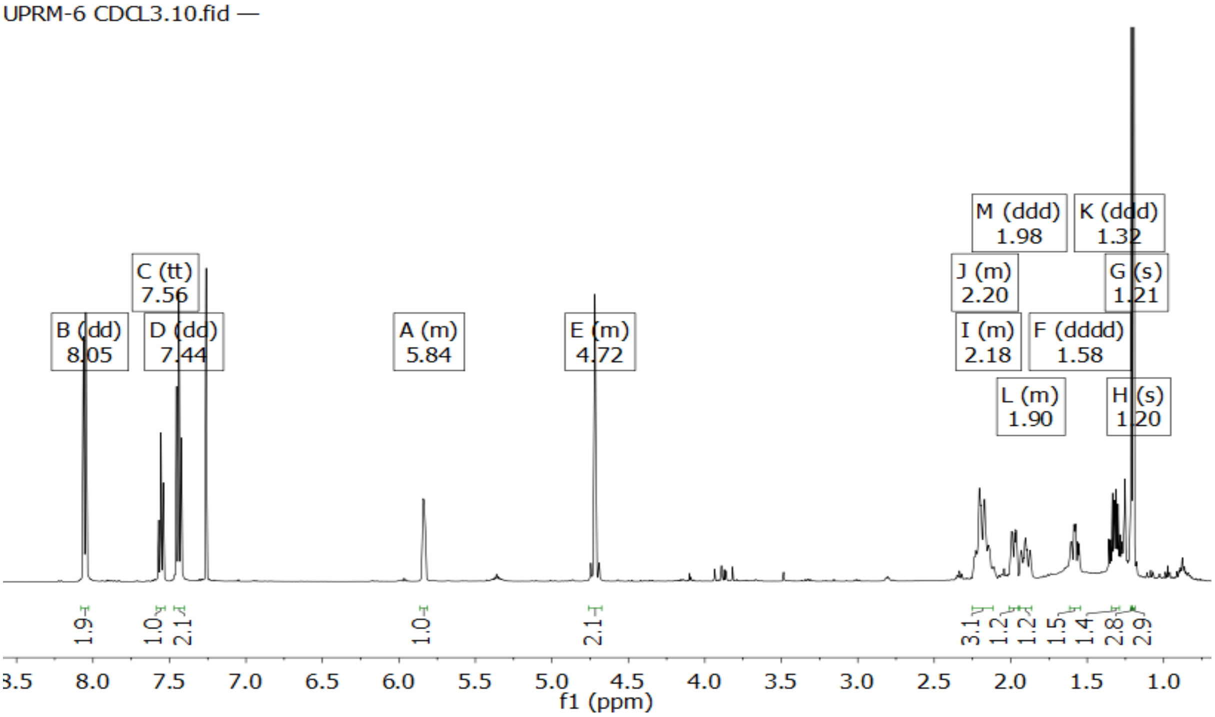

Figure S33. The ^{1}H NMR spectrum of compound **5** (500 MHz, $CDCl_3$)

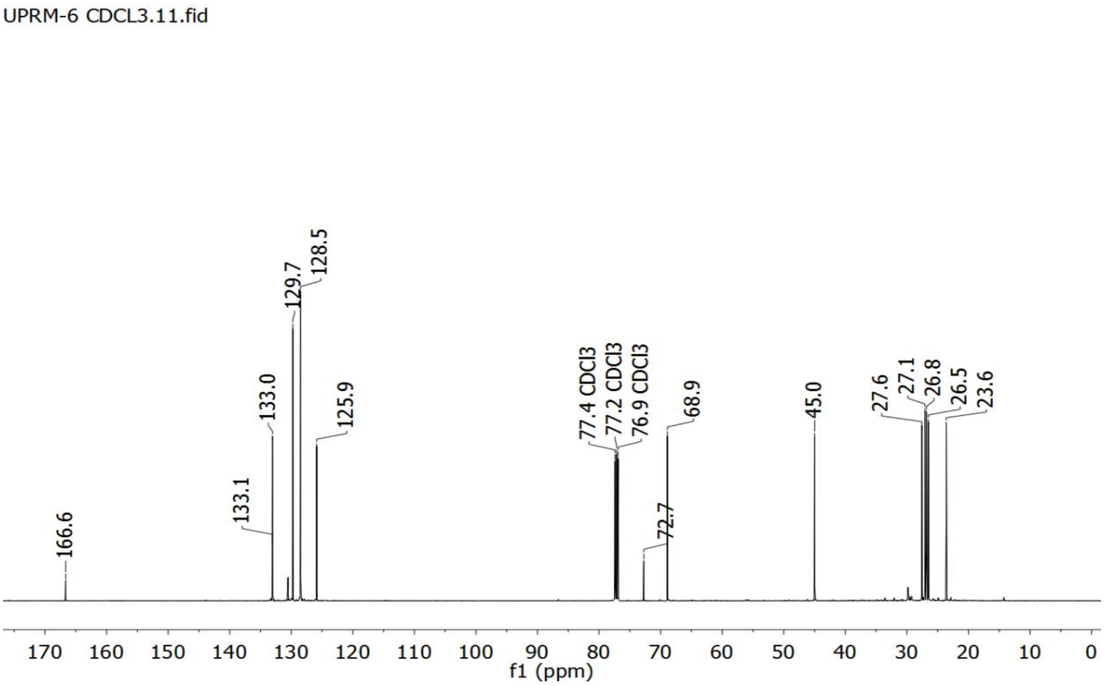

Figure S34. The ^{13}C NMR spectrum of compound **5** (125 MHz, $CDCl_3$)

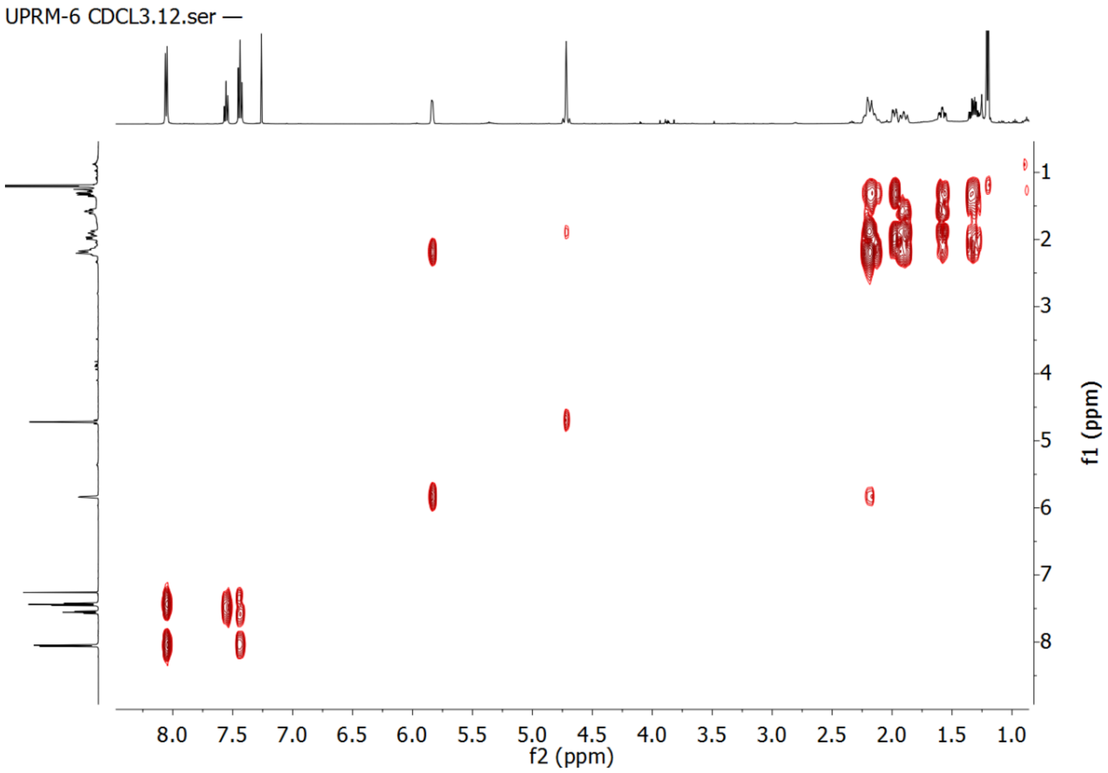

Figure S35. The COSY spectrum of compound **5** (500 MHz, $CDCl_3$)

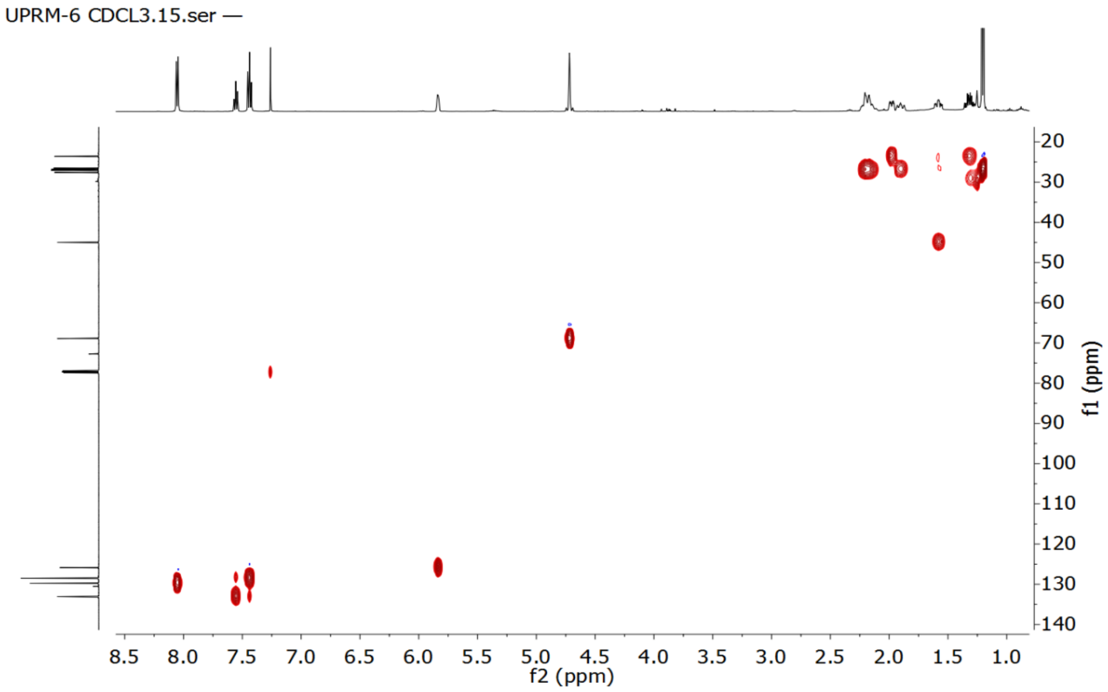

Figure S36. The HSQC spectrum of compound **5** (500/125 MHz, $CDCl_3$)

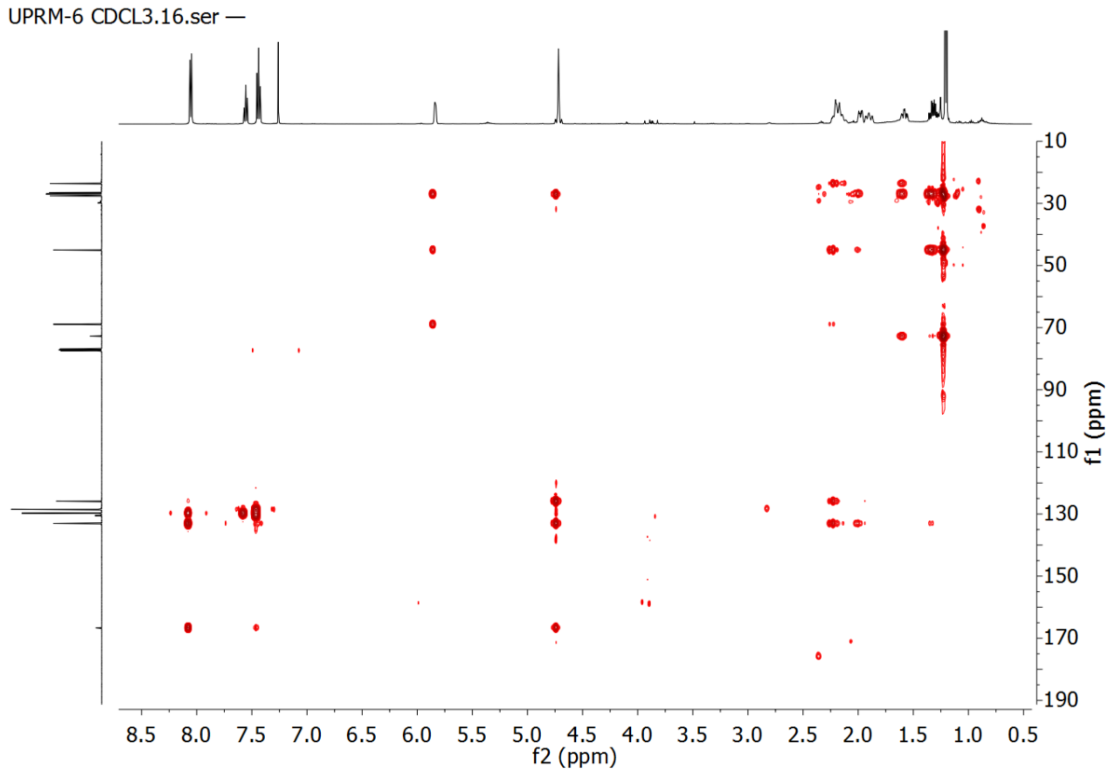

Figure S37. The HMBC spectrum of compound **5** (500/125 MHz, $CDCl_3$)

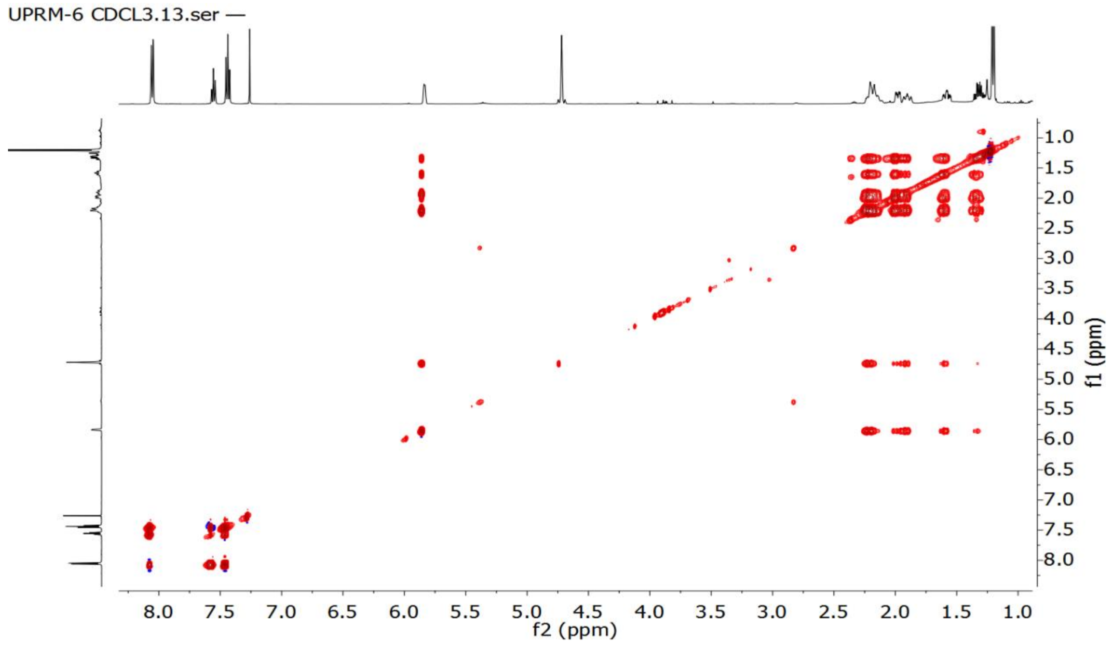

Figure S38. The TOCSY spectrum of compound **5** (500 MHz, $CDCl_3$)

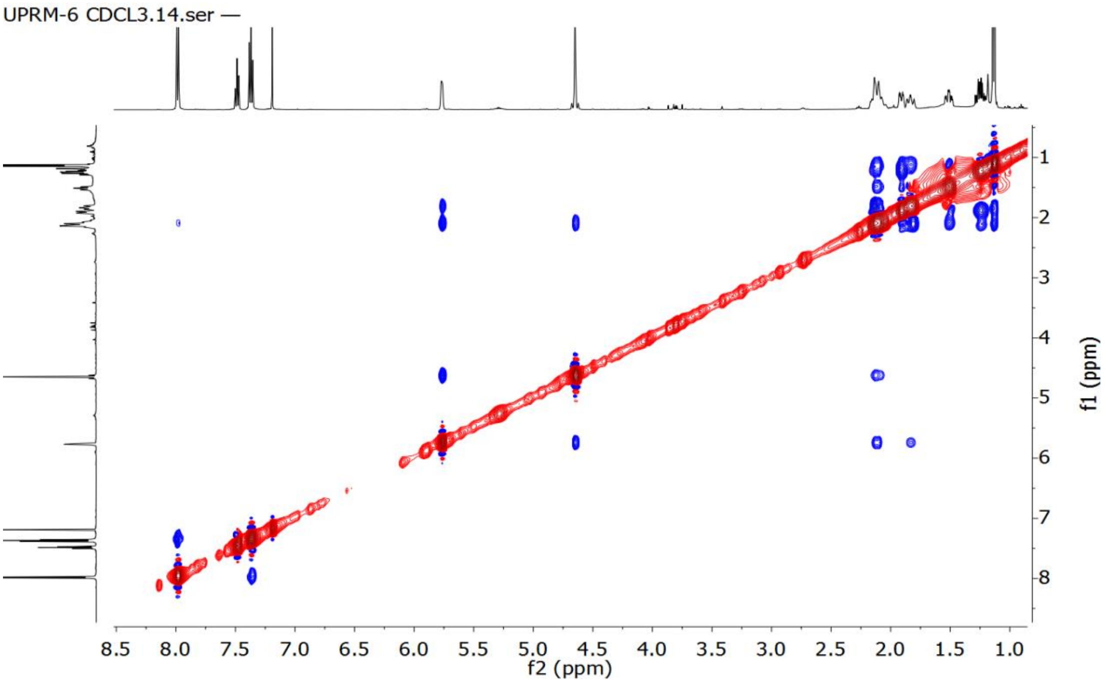

Figure S39. The NOESY spectrum of compound **5** (500 MHz, $CDCl_3$)

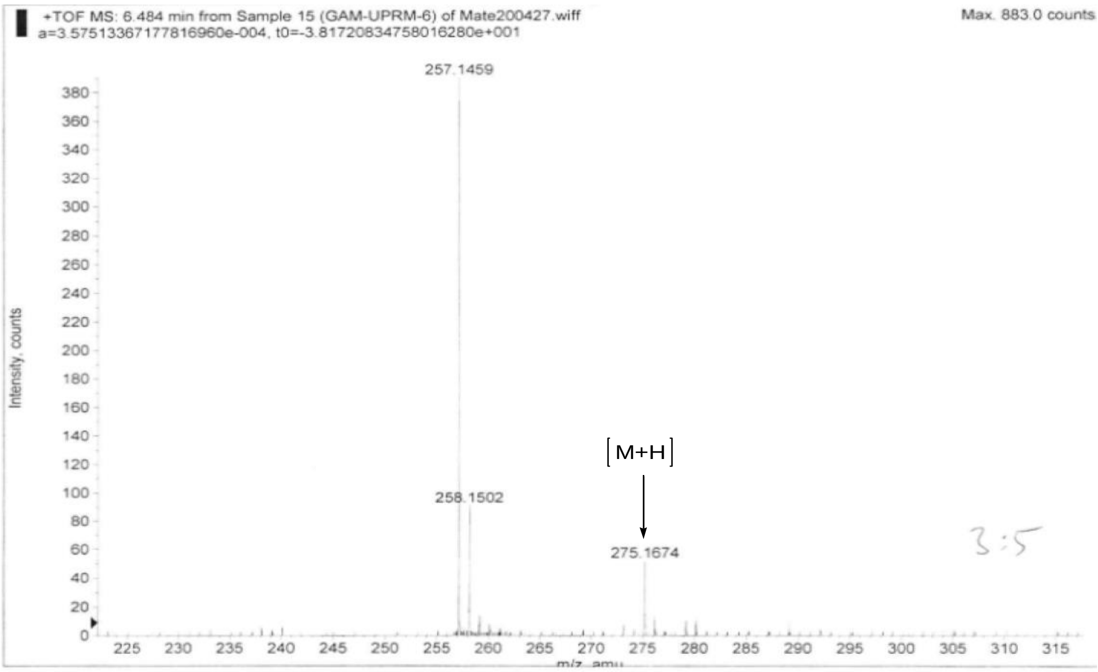

Figure S40. The HRISIMS spectrum of compound **5**

7. Spectroscopic data for compound 6

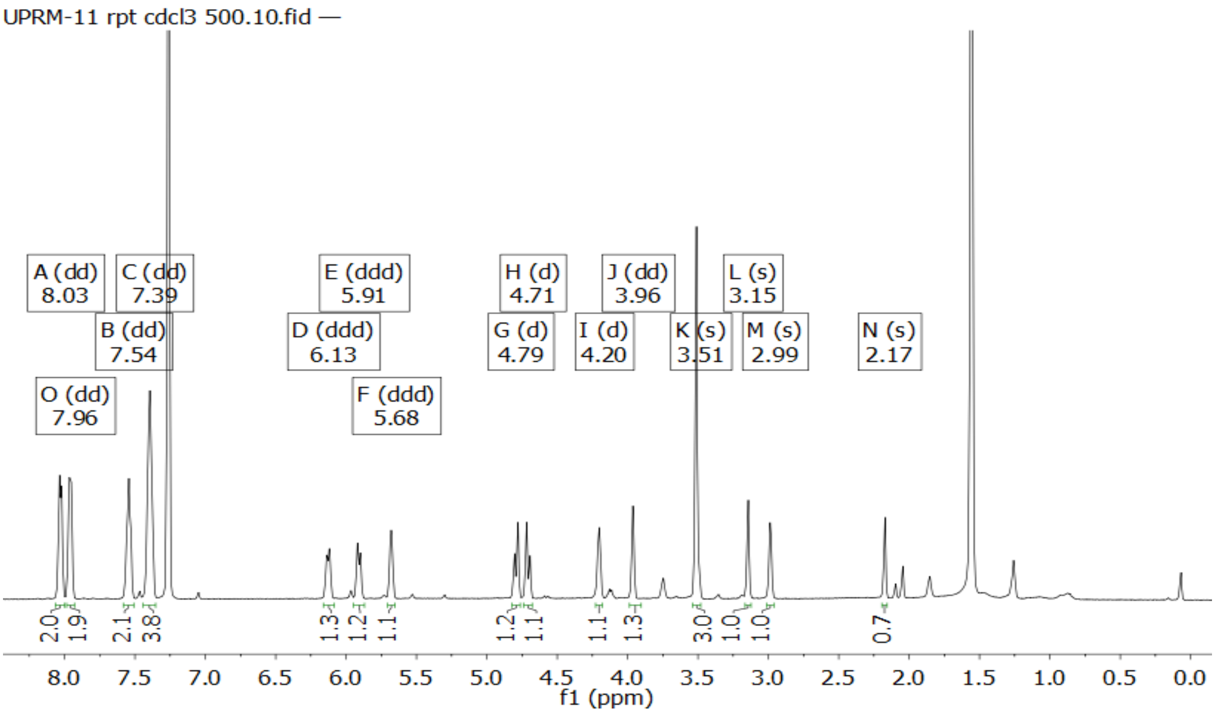

Figure S41. The ^{1}H NMR spectrum of compound **6** (500 MHz, $CDCl_3$)

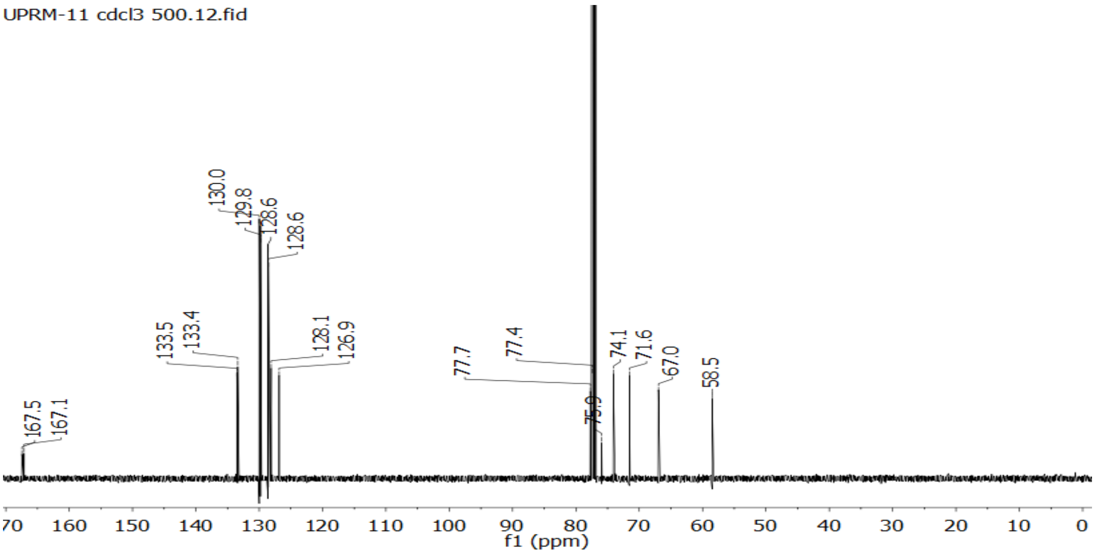

Figure S42. The ^{13}C NMR spectrum of compound **6** (125 MHz, $CDCl_3$)

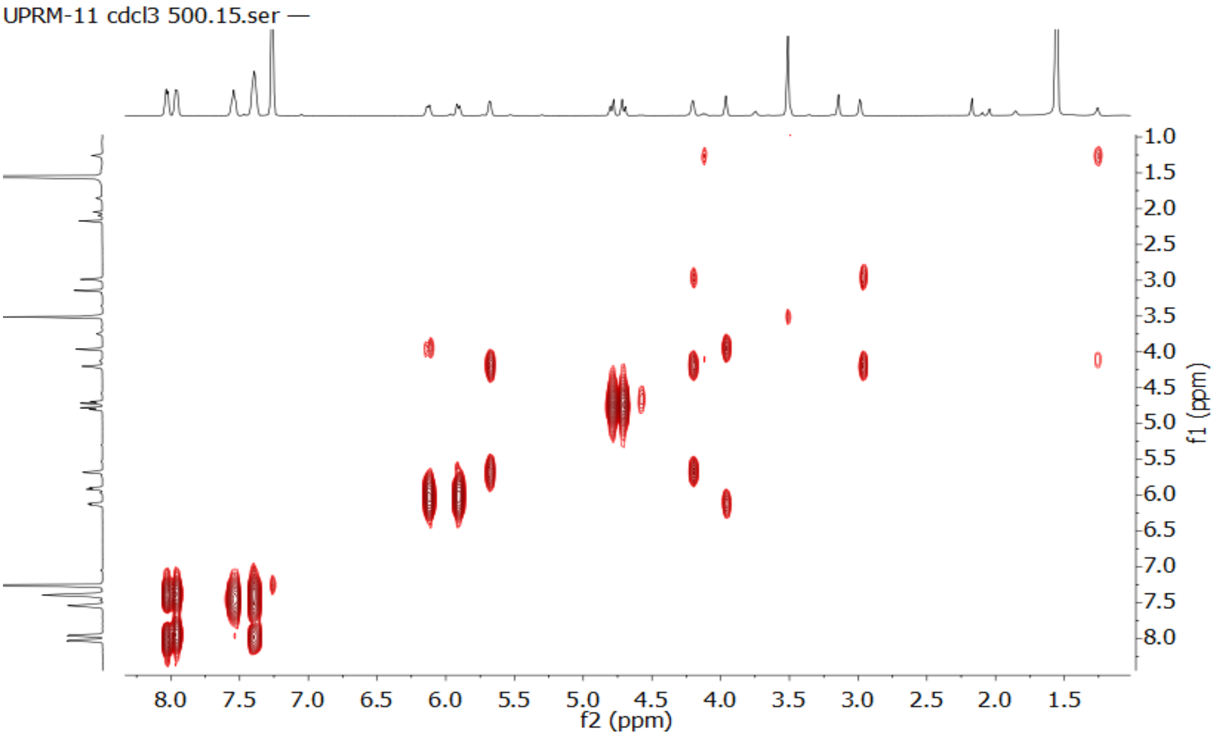

Figure S43. The COSY spectrum of compound **6** (500 MHz, $CDCl_3$)

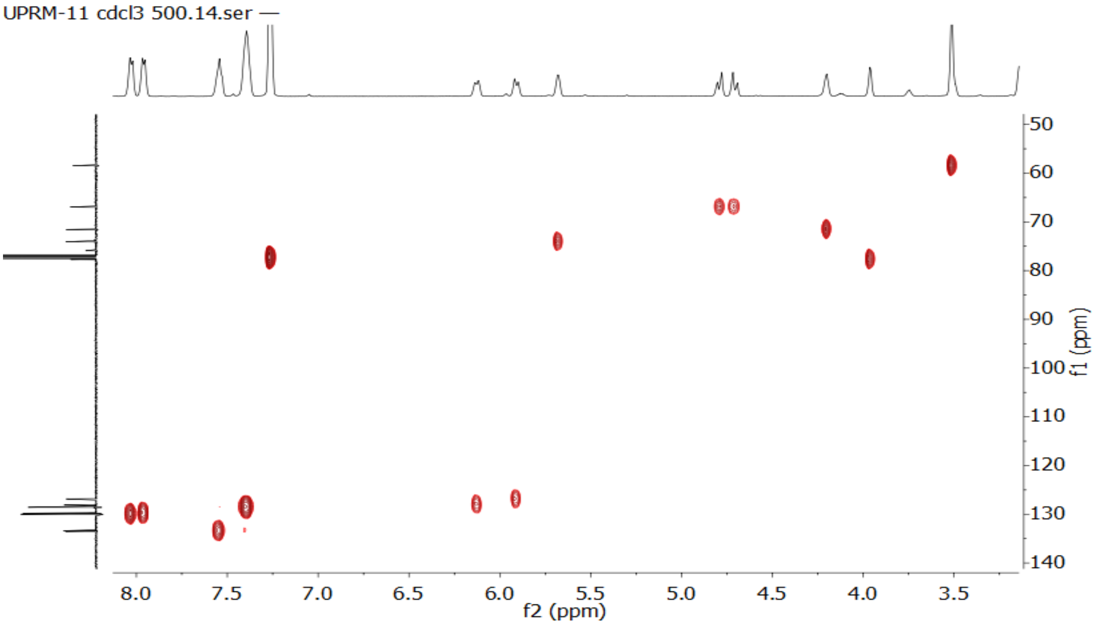

Figure S44. The HSQC spectrum of compound 6 (500/125 MHz, $CDCl_3$)

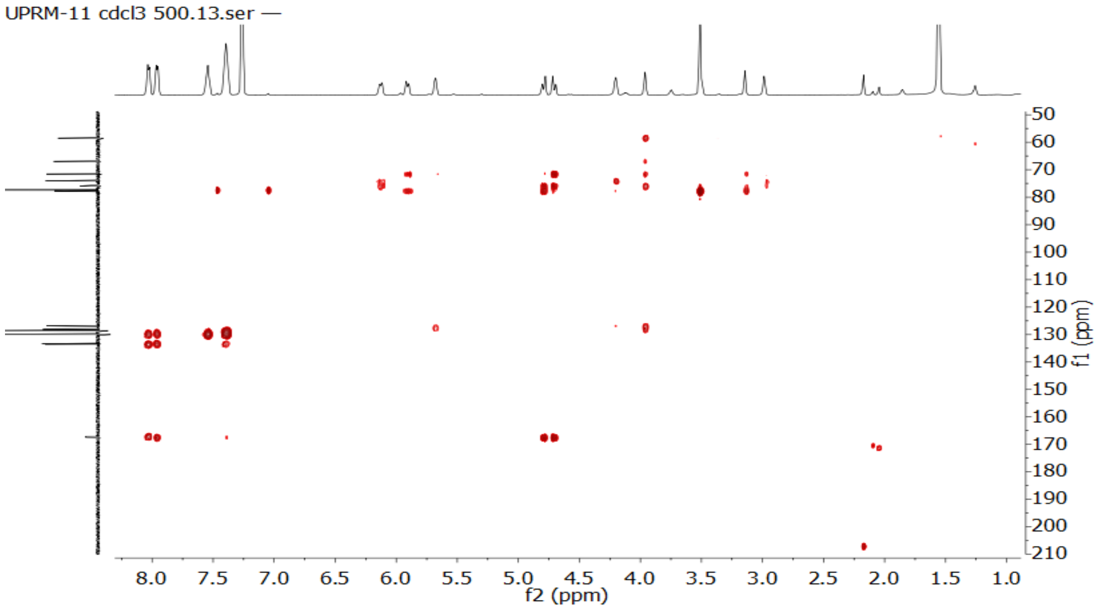

Figure S45. The HMBC spectrum of compound **6** (500/125 MHz, $CDCl_3$)

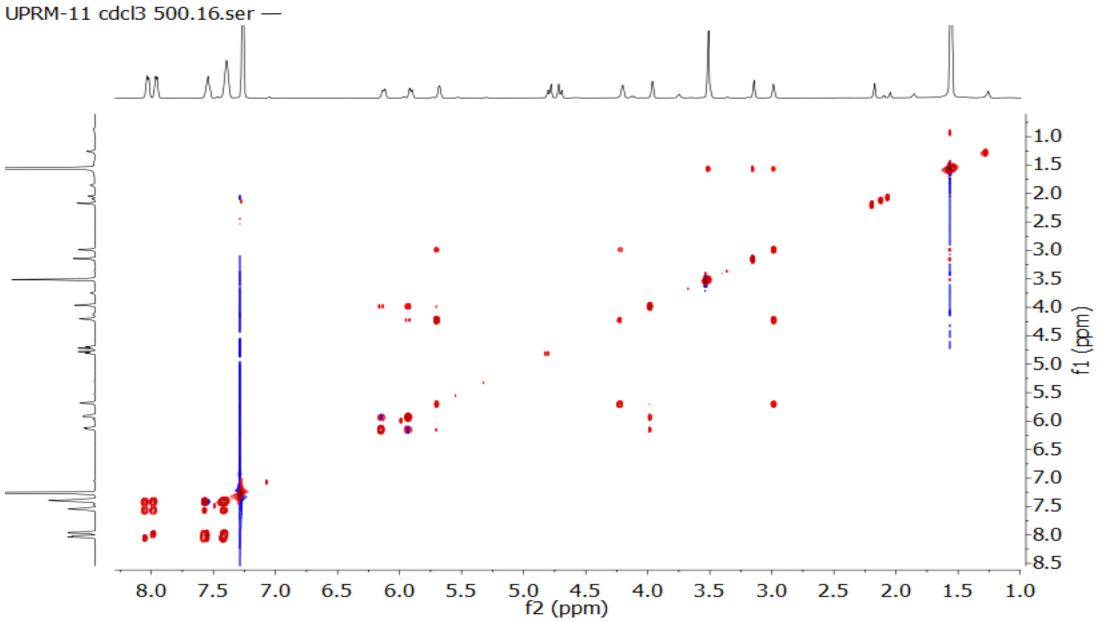

Figure S46. The TOCSY spectrum of compound 6 (500 MHz, $CDCl_3$)

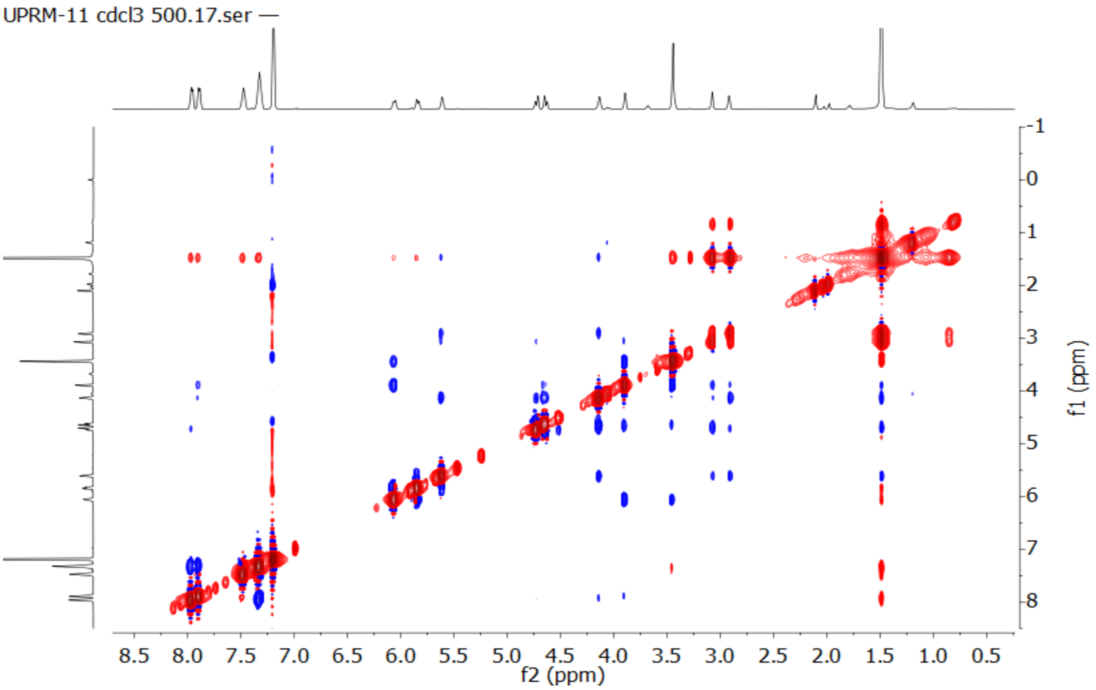

Figure S47. The NOESY spectrum of compound **6** (500 MHz, CDCl3)

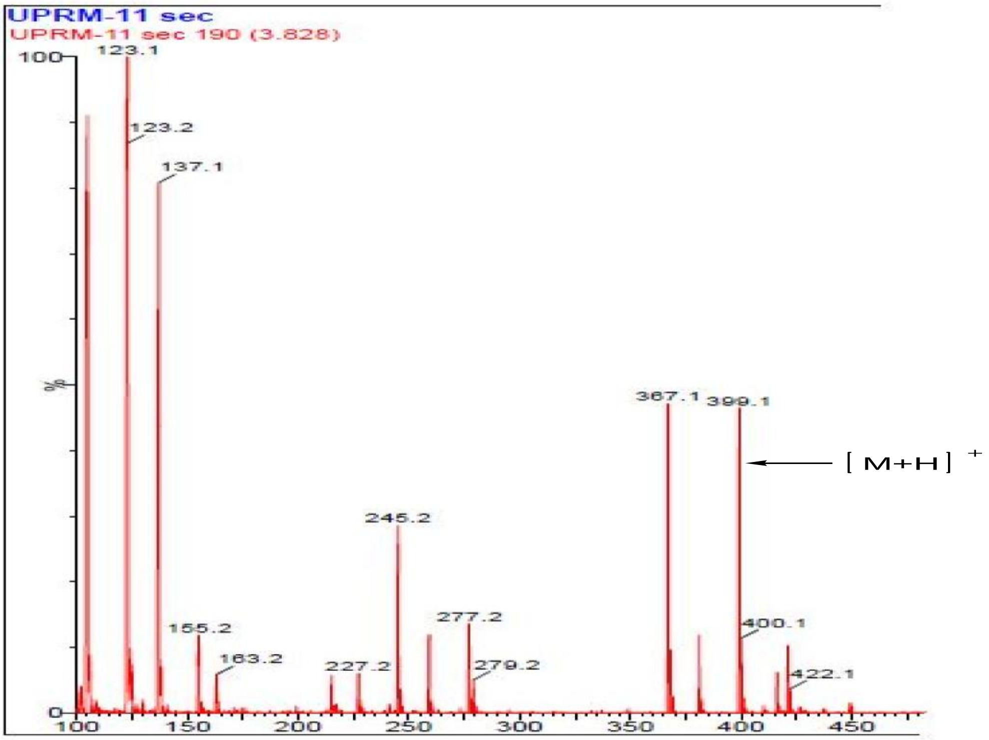

Figure S48: The LC-MS of compound **6**

8. Spectroscopic data for compound 7

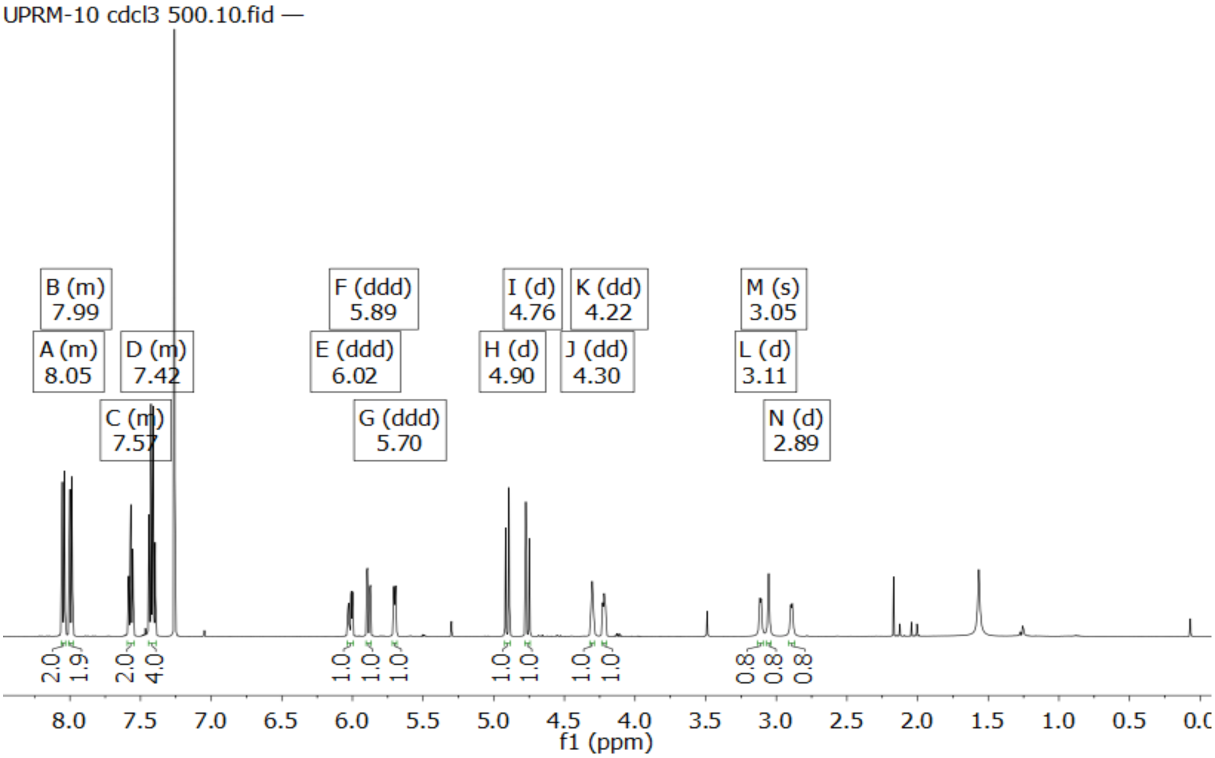

Figure S49. The ^{1}H NMR Spectrum of compound **7** (500 MHz, $CDCl_3$)

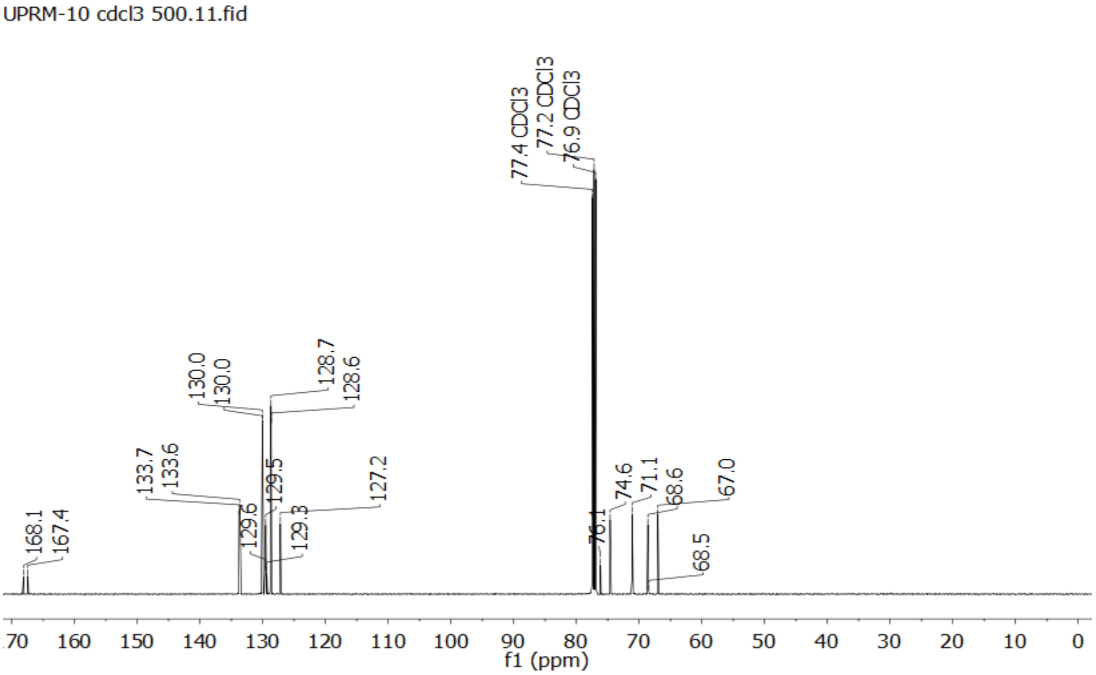

Figure S50. The ^{13}C NMR spectrum of compound **7** (125 MHz, $CDCl_3$)

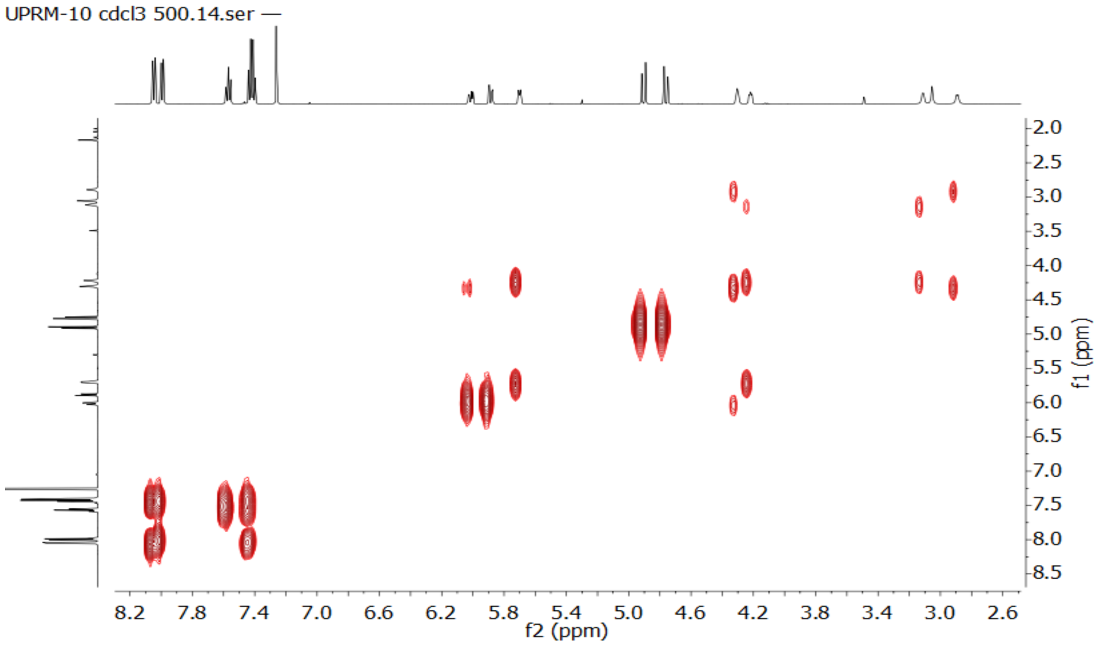

Figure S51. The COSY spectrum of compound **7** (500 MHz, $CDCl_3$)

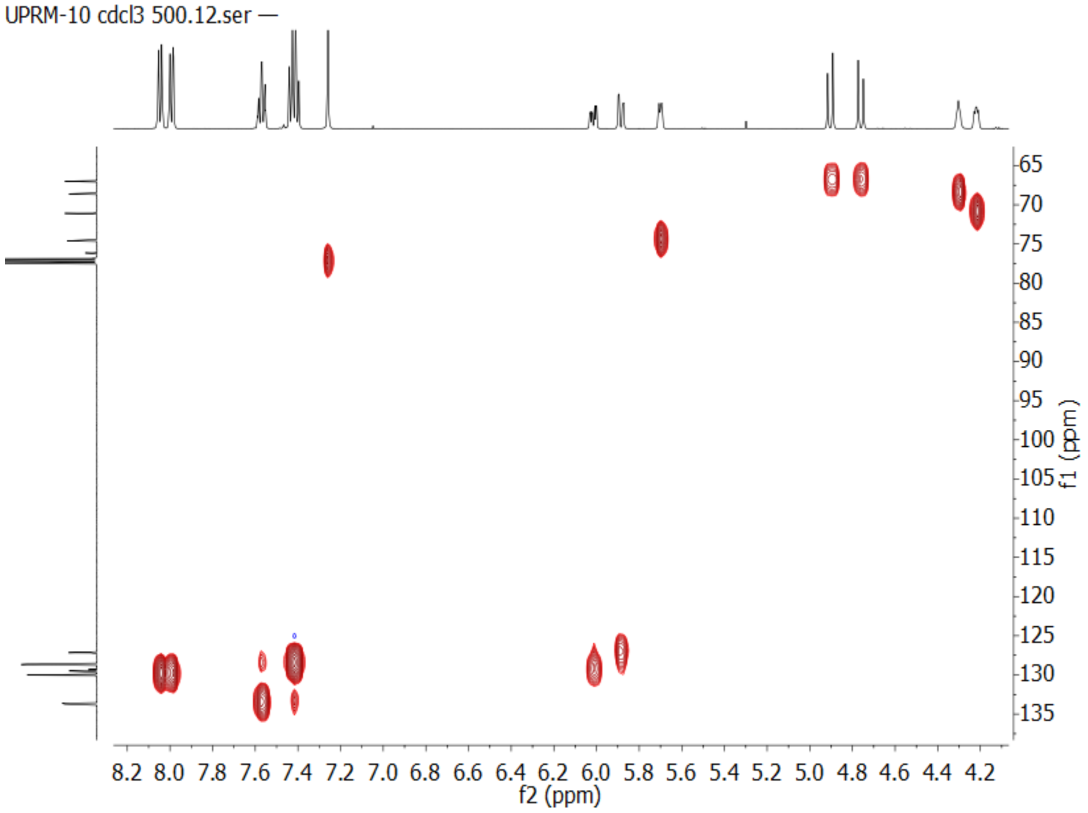

Figure S52. The HSQC spectrum of compound **7** (500/125 MHz, $CDCl_3$)

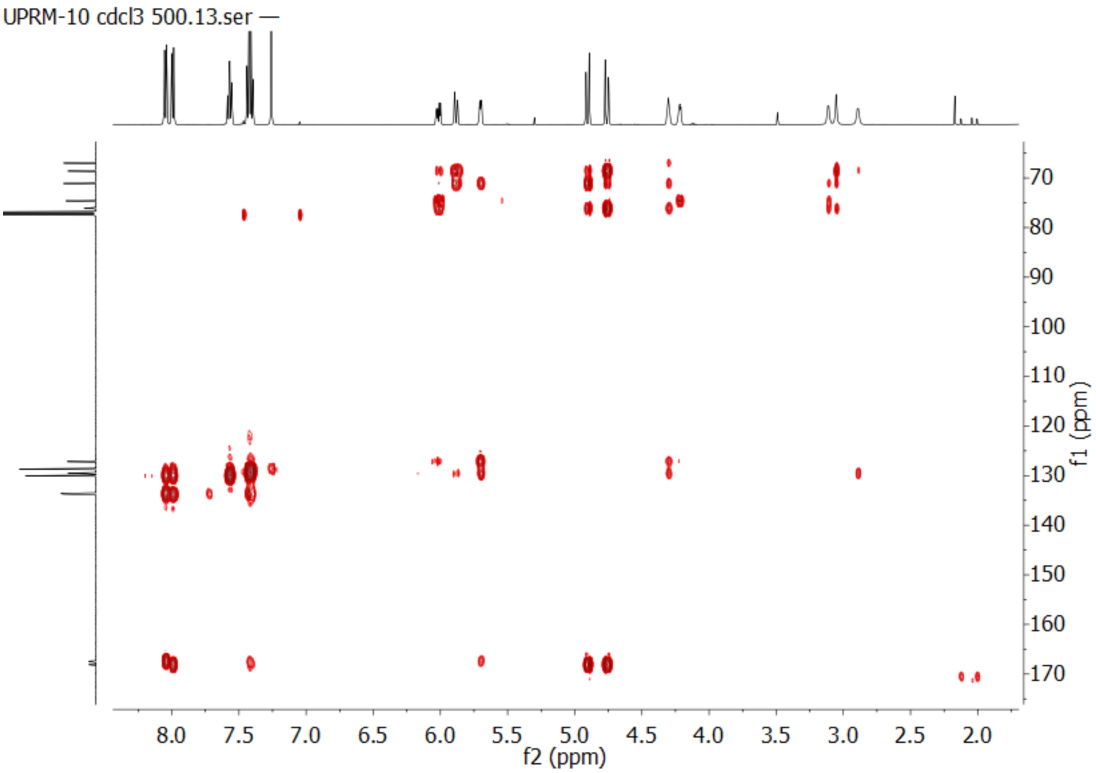

Figure S53. The HMBC spectrum of compound **7** (500/125 MHz, $CDCl_3$)

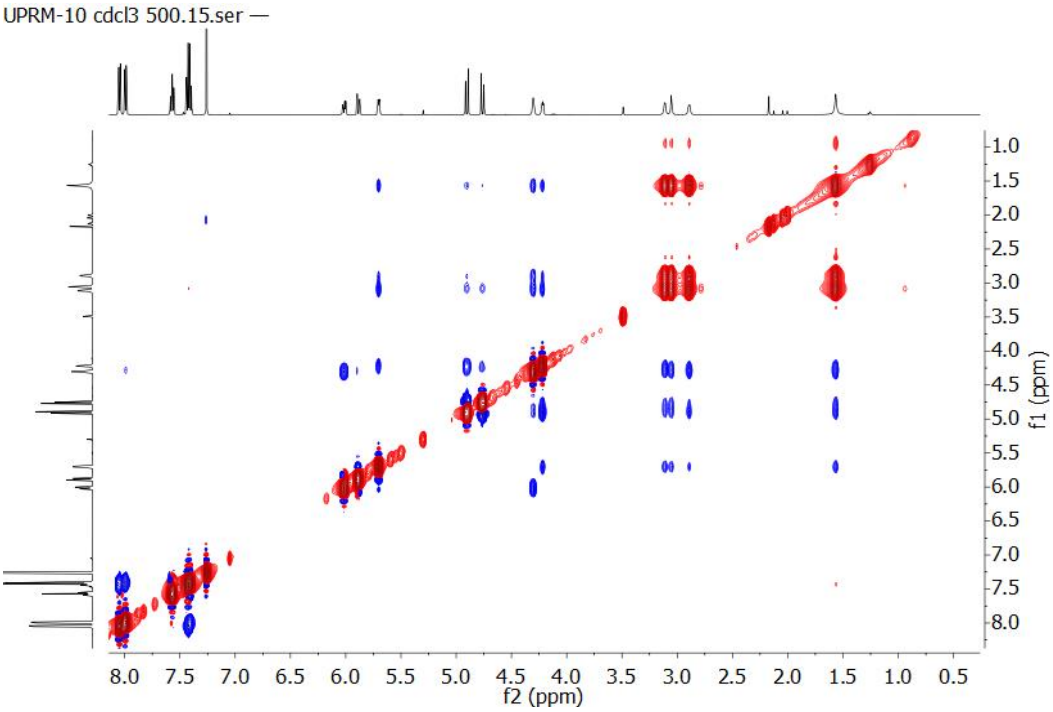

Figure S54. The NOESY spectrum of compound **7** (500 MHz, $CDCl_3$)

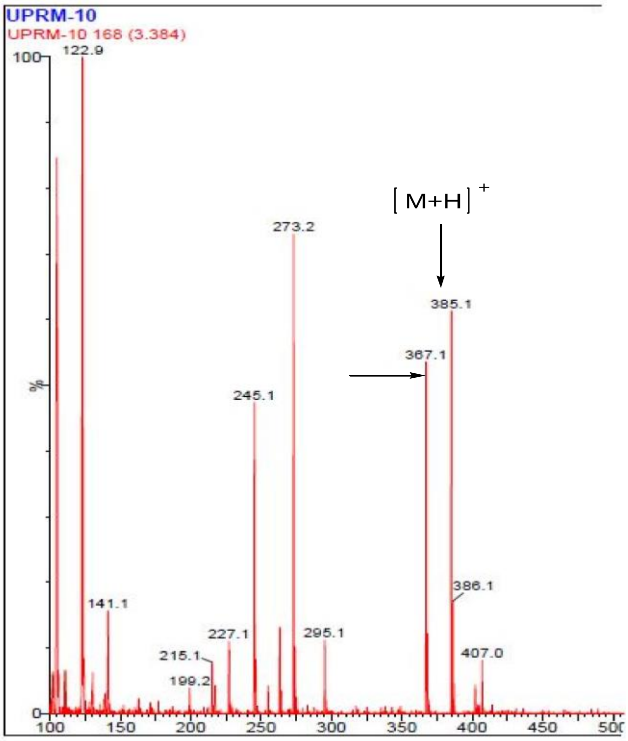

Figure S55. The LC-MS of compound **7**

9. Spectroscopic data for compound 8

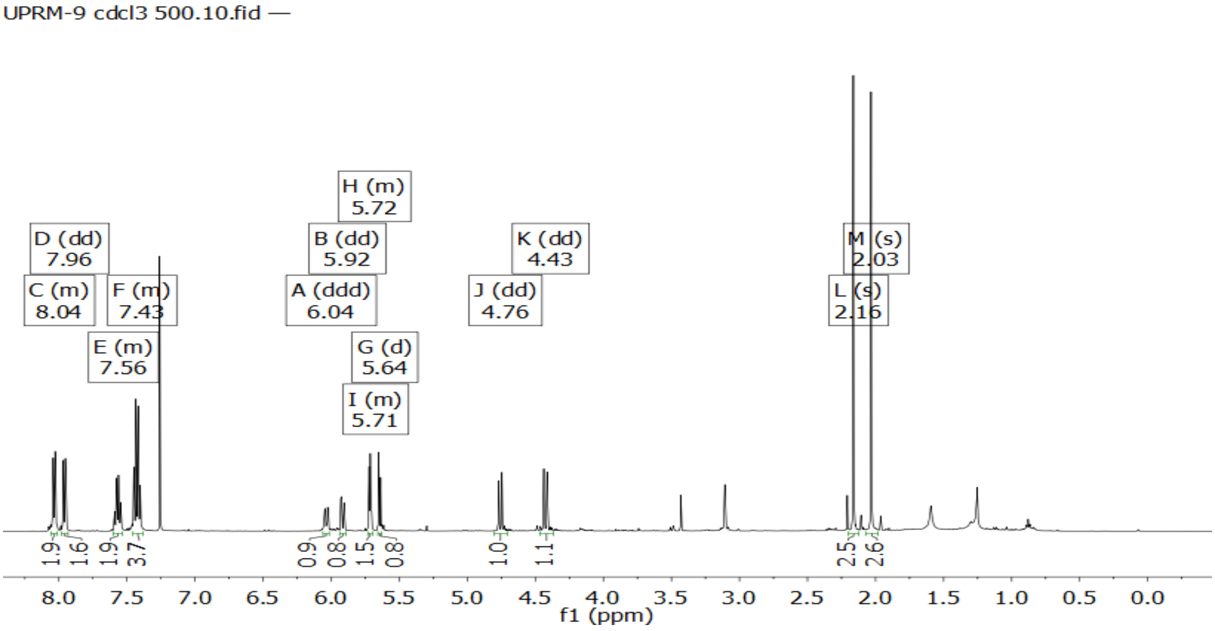

Figure S56. The ^{1}H NMR spectrum of compound **8** (600 MHz, $CDCl_3$)

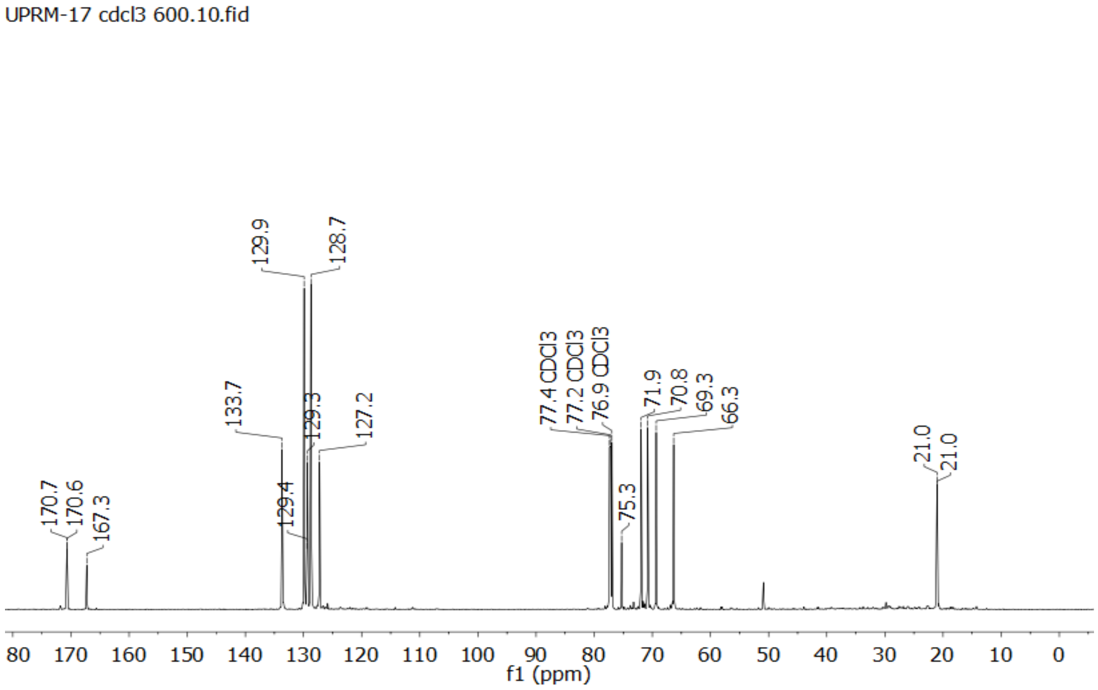

Figure S57. The ^{13}C NMR spectrum of compound **8** (150 MHz, $CDCl_3$)

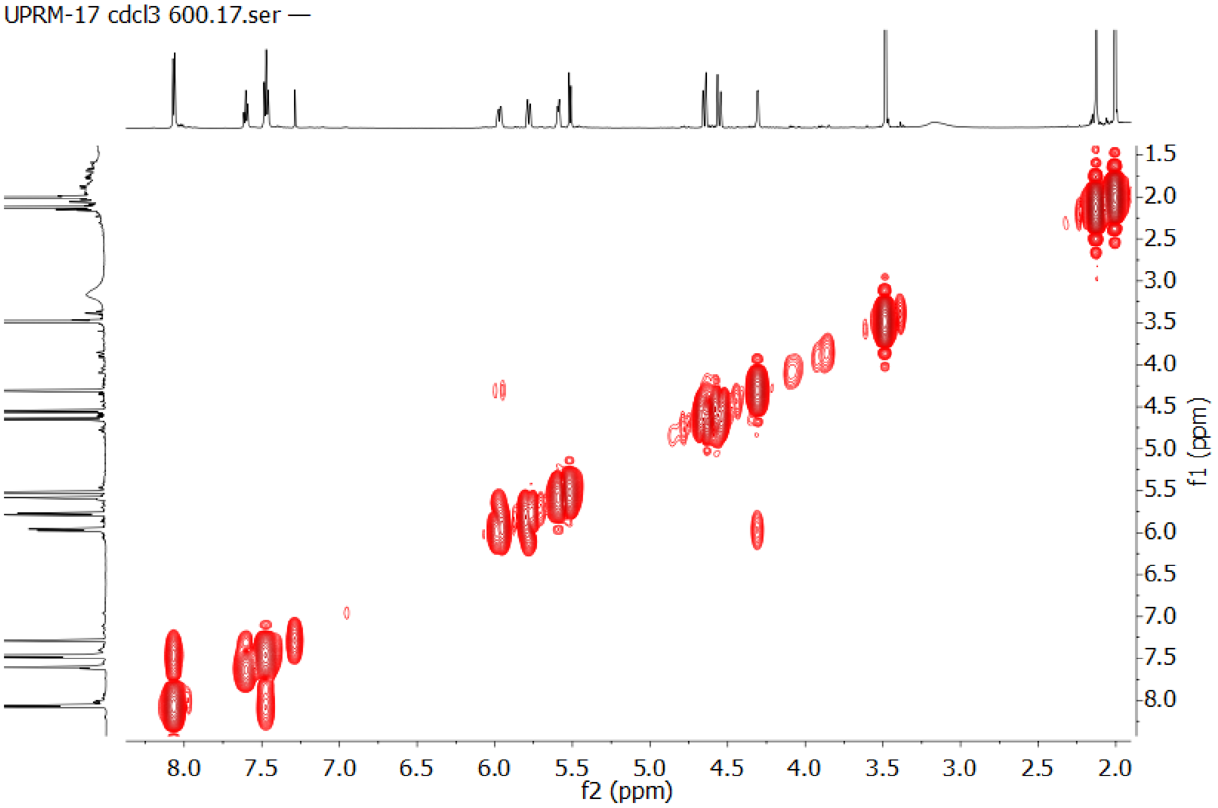

Figure S58. The COSY spectrum of compound **8** (600 MHz, $CDCl_3$)

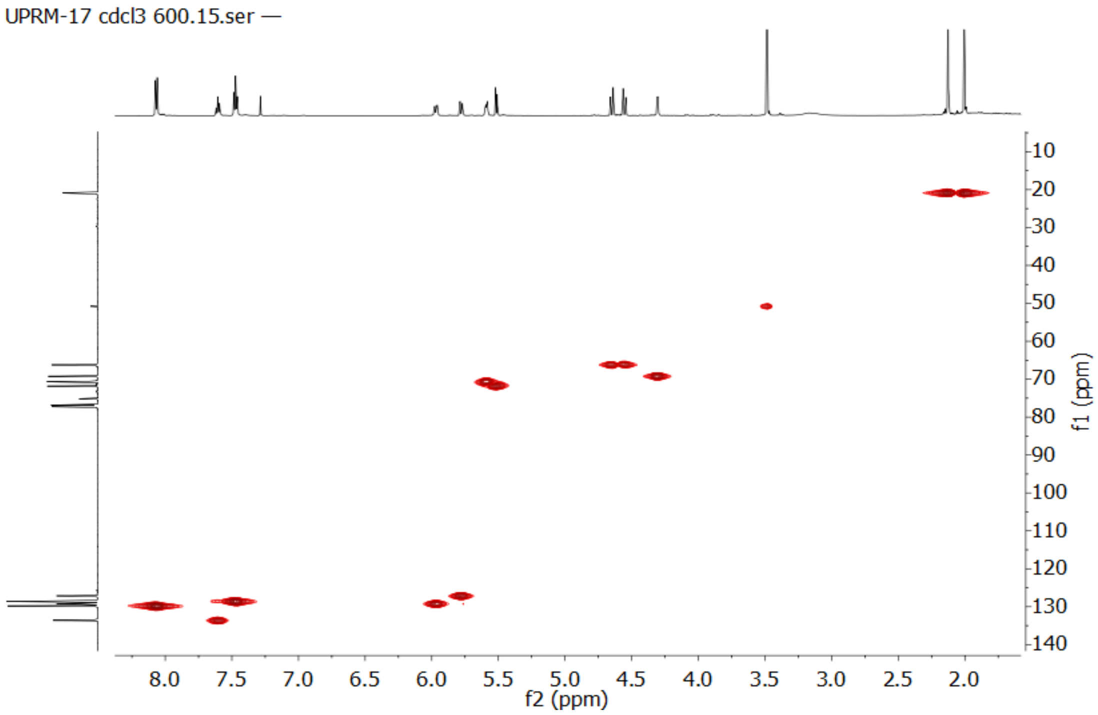

Figure S59. The HSQC spectrum of compound **8** (600/150 MHz, $CDCl_3$)

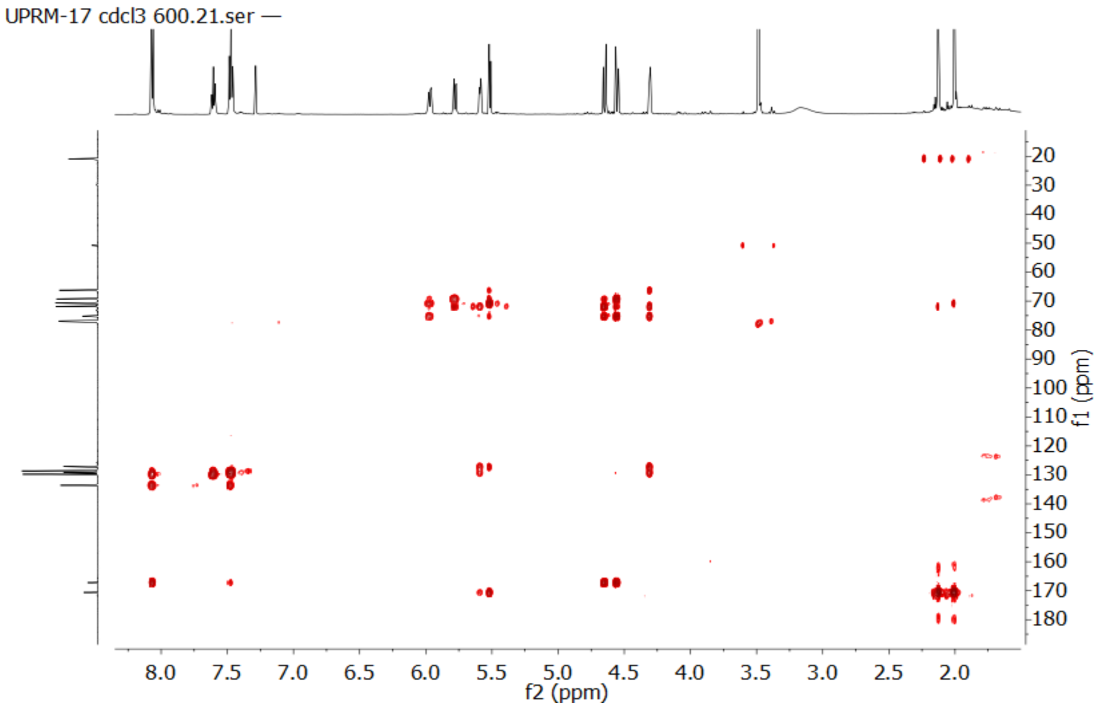

Figure S60. The HMBC spectrum of compound **8** (600/150 MHz, $CDCl_3$)

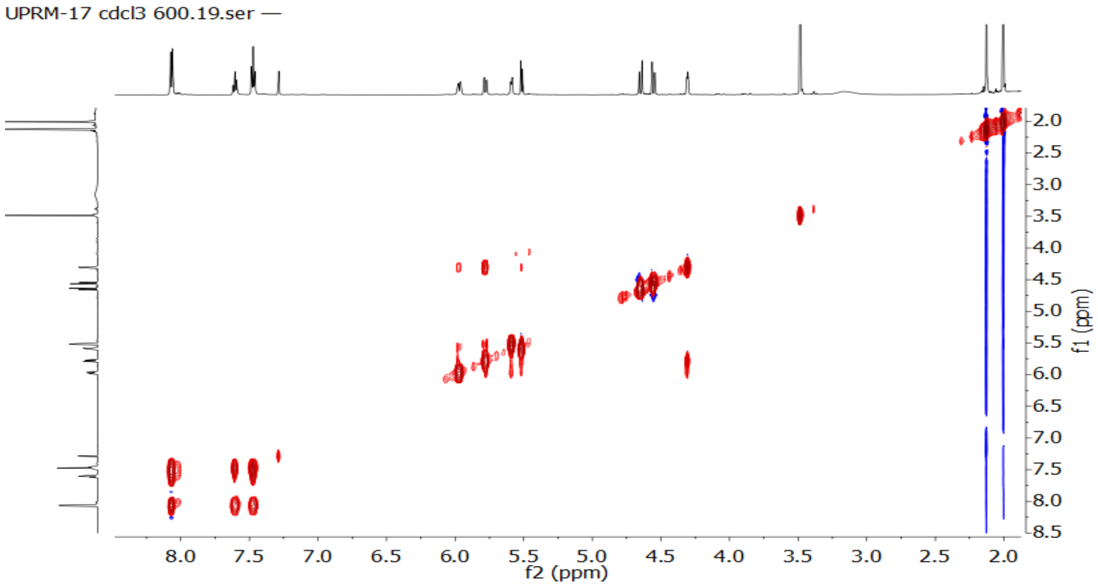

Figure S61. TOCSY spectrum of compound **8** (600 MHz, $CDCl_3$)

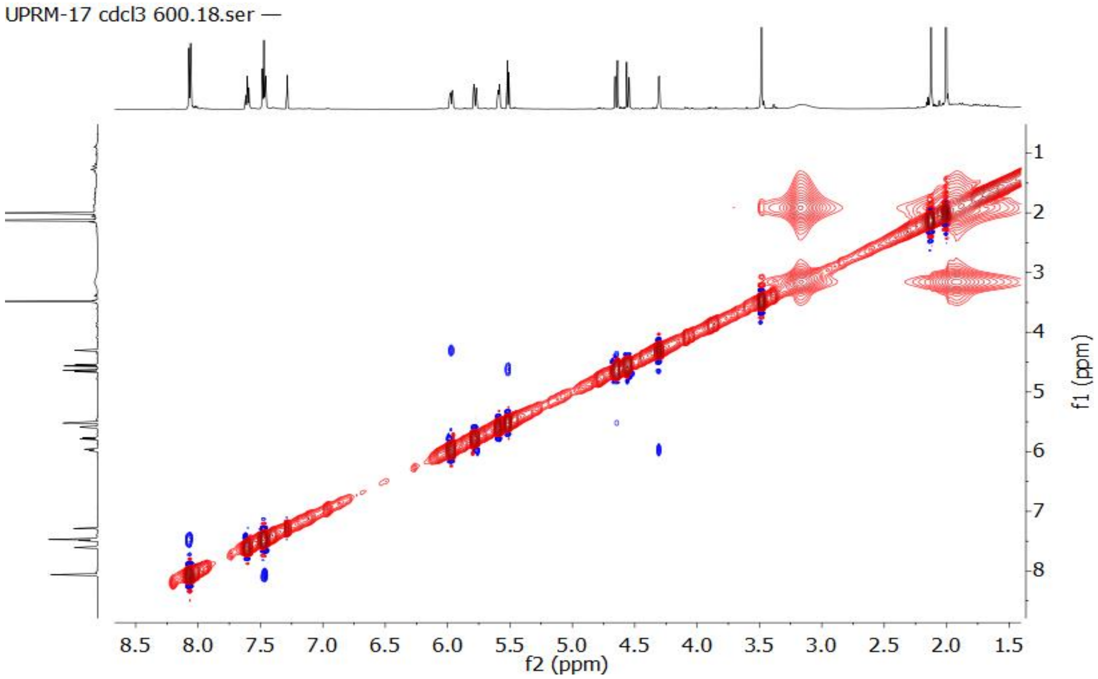

Figure S62. The NOESY spectrum of compound **8** (600 MHz, $CDCl_3$)

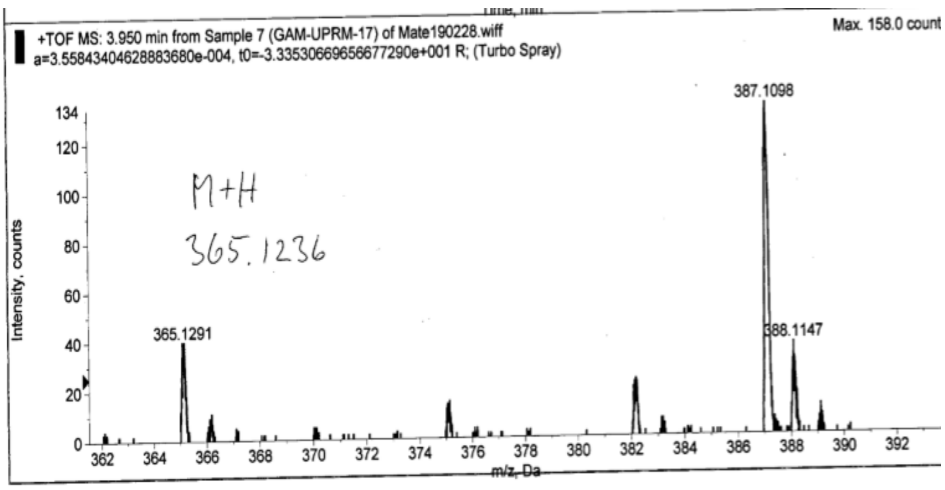

Figure S63. HREIMS spectrum of compound **8**

10. Spectroscopic data for compound 9

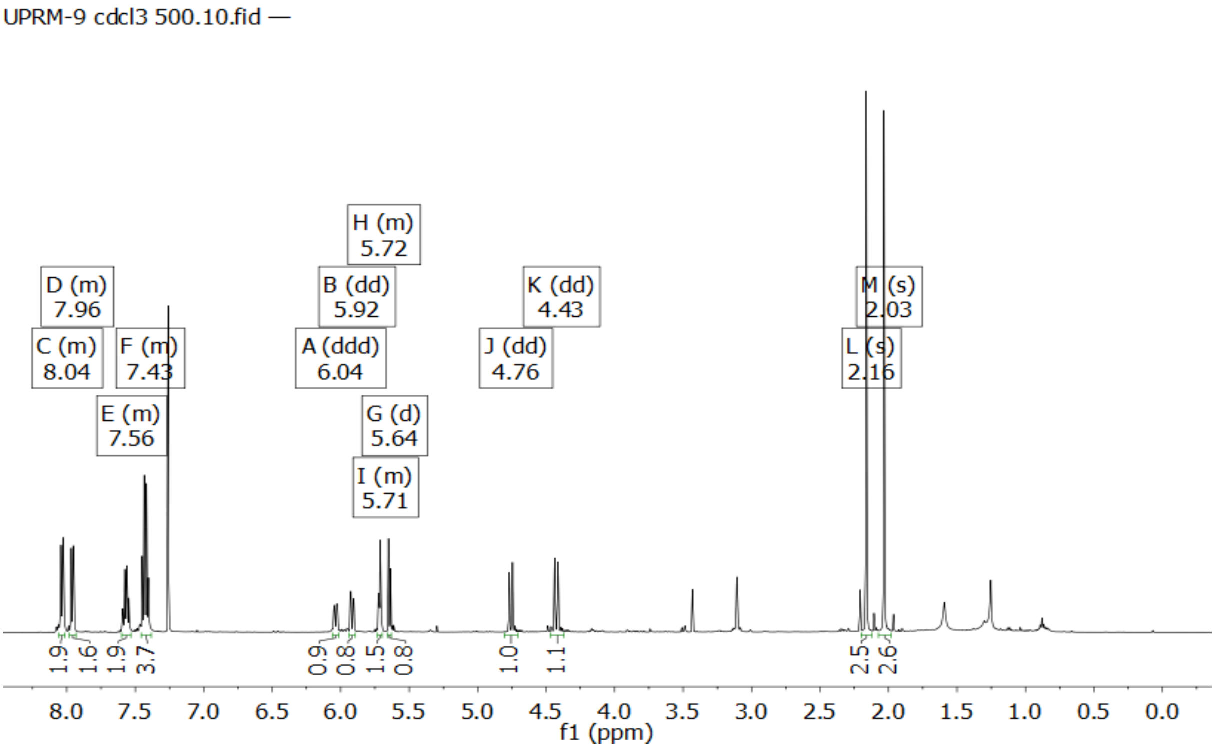

Figure S64. The ^{1}H NMR spectrum of compound **9** (500 MHz, $CDCl_3$)

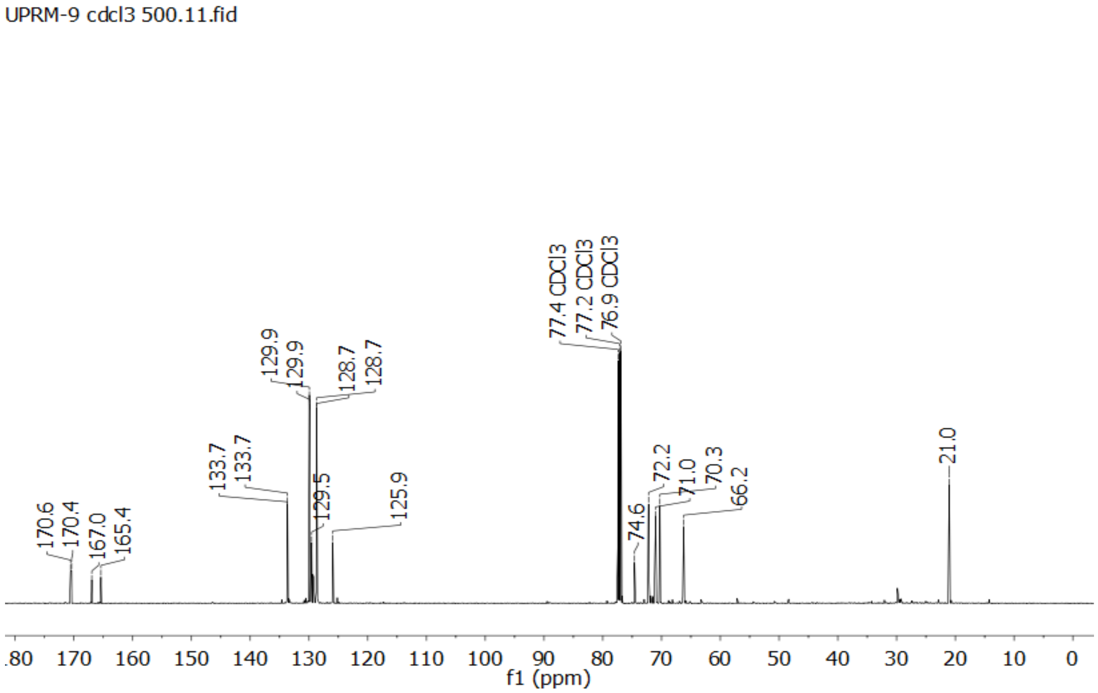

Figure S65. The ^{13}C NMR spectrum of compound **9** (125 MHz, $CDCl_3$)

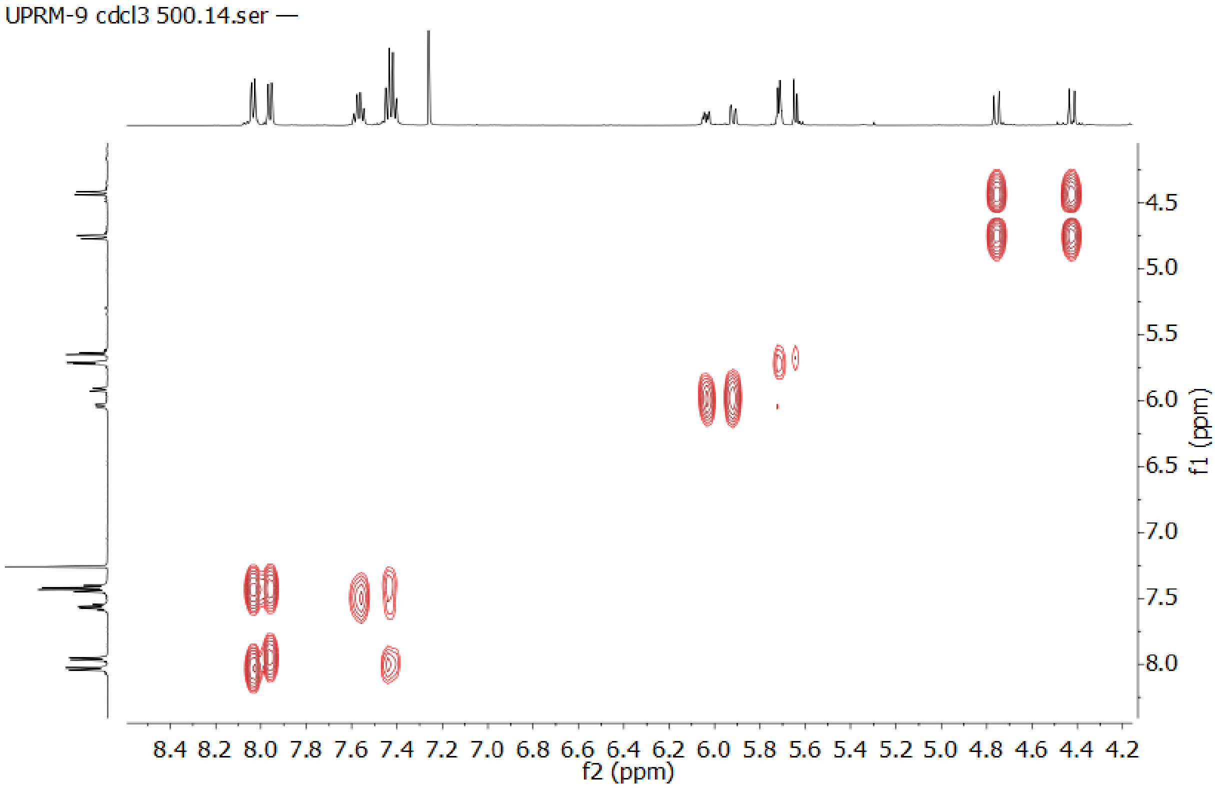

Figure S66. The COSY spectrum of compound **9** (500 MHz, $CDCl_3$)

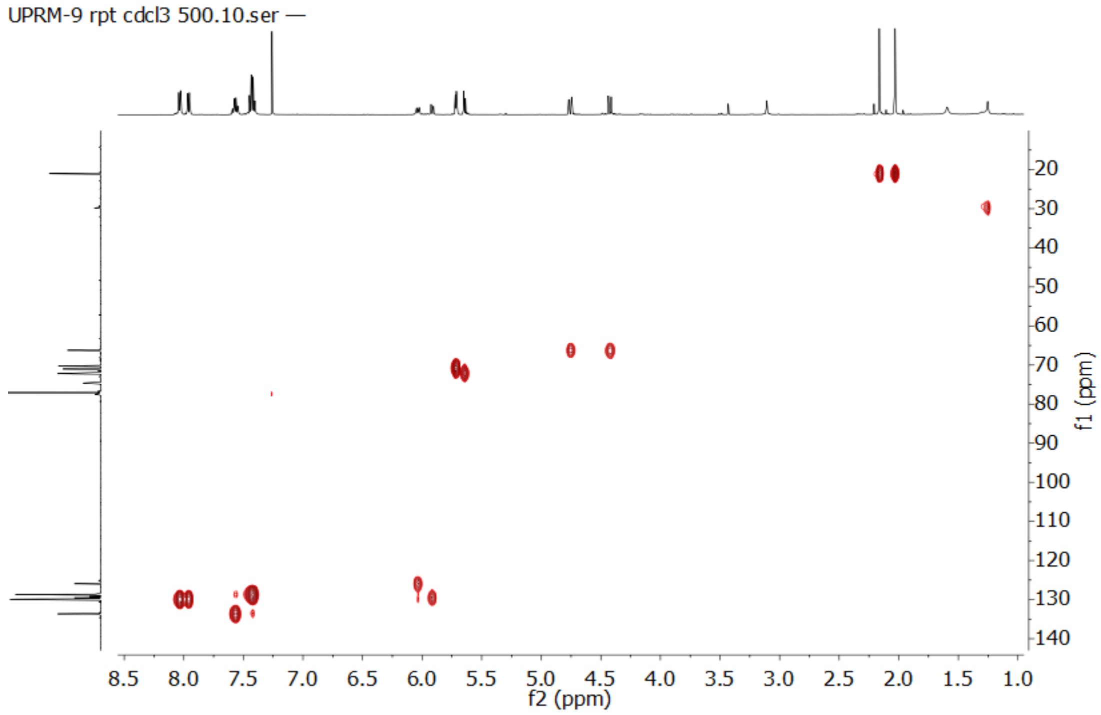

Figure S67. The HSQC spectrum of compound **9** (500/125 MHz, $CDCl_3$)

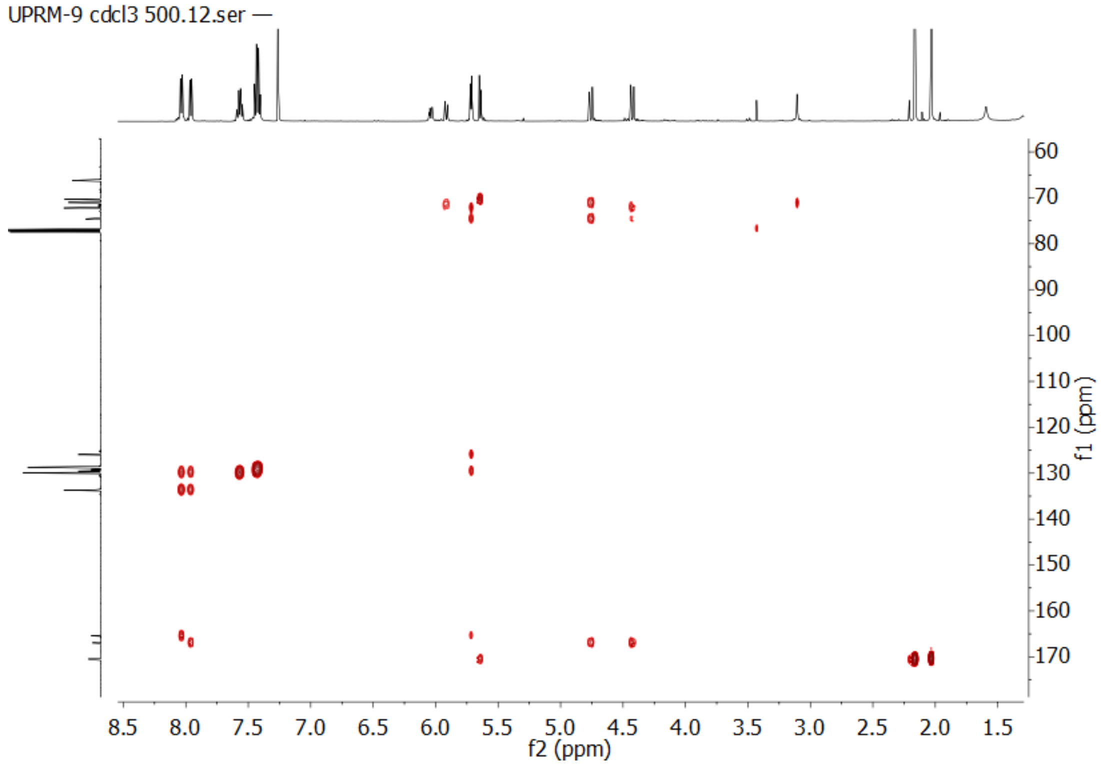

Figure S68. The HMBC spectrum of compound **9** (500/125 MHz, $CDCl_3$)

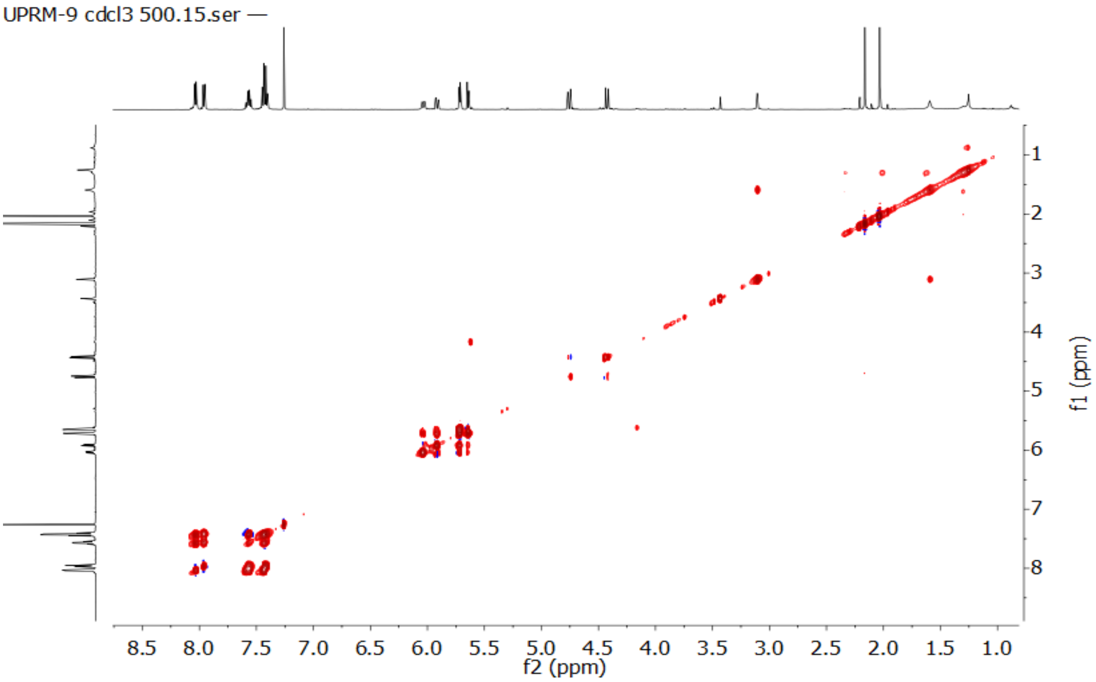

Figure S69. The TOCSY spectrum of compound **9** (500 MHz, $CDCl_3$)

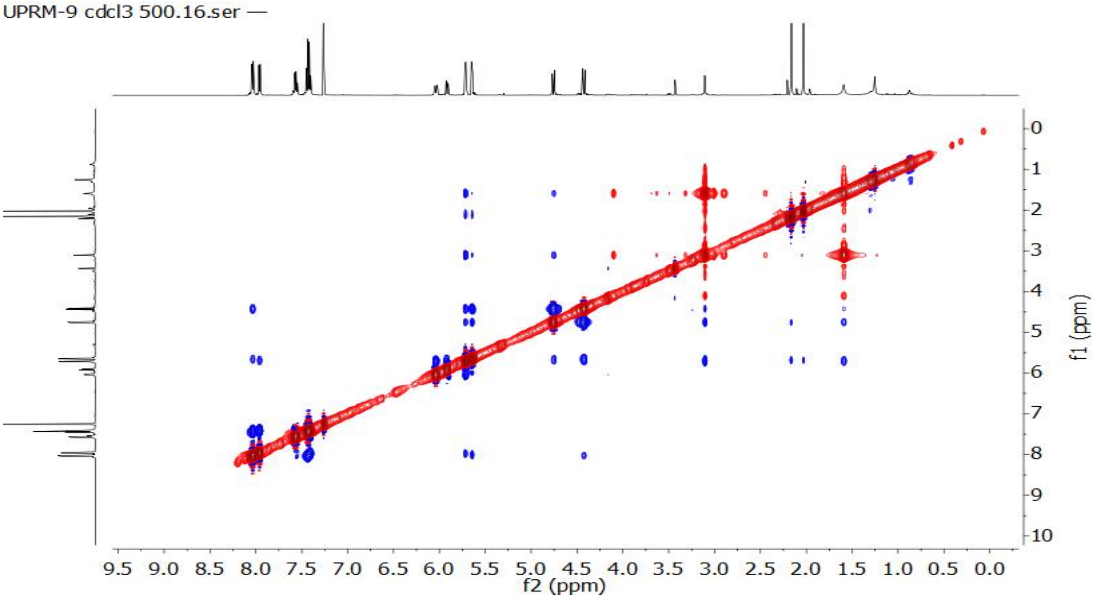

Figure S70. The NOESY spectrum of compound **9** (500 MHz, $CDCl_3$)

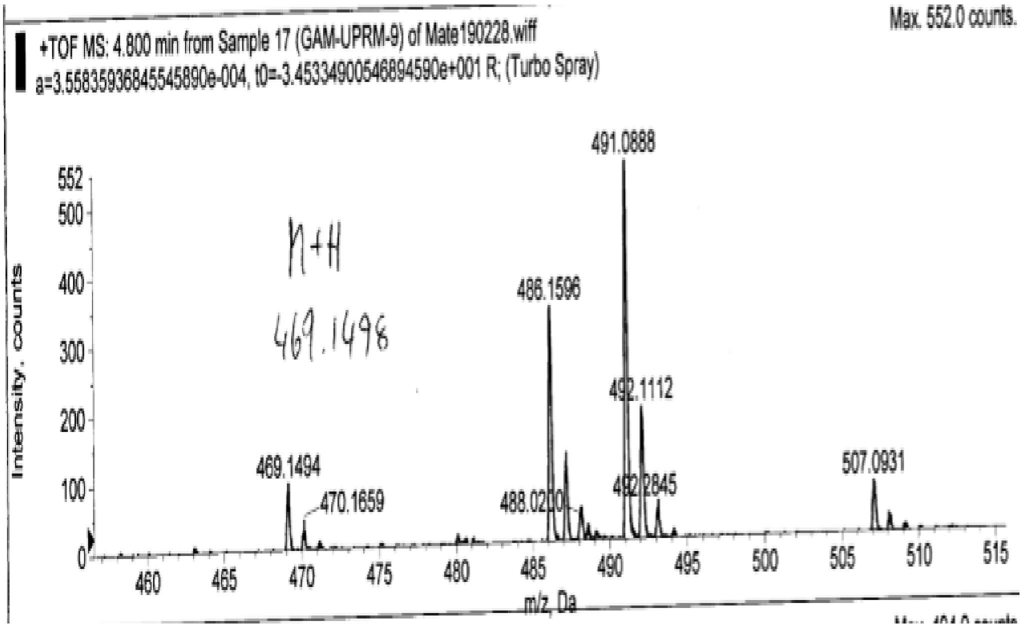

Figure S71. HREIMS spectrum of compound **9**

11. Spectroscopic data for compound 10

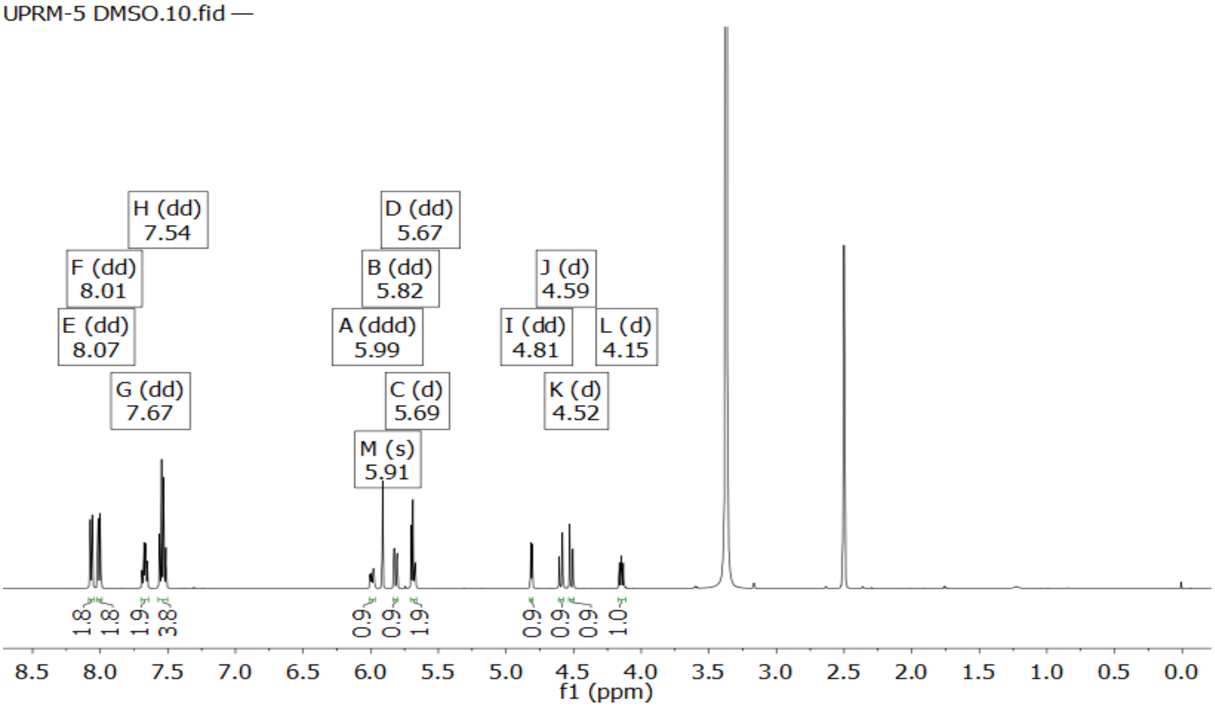

Figure S72. The ^{1}H NMR spectrum of compound **10** (500 MHz, DMSO-d_6)

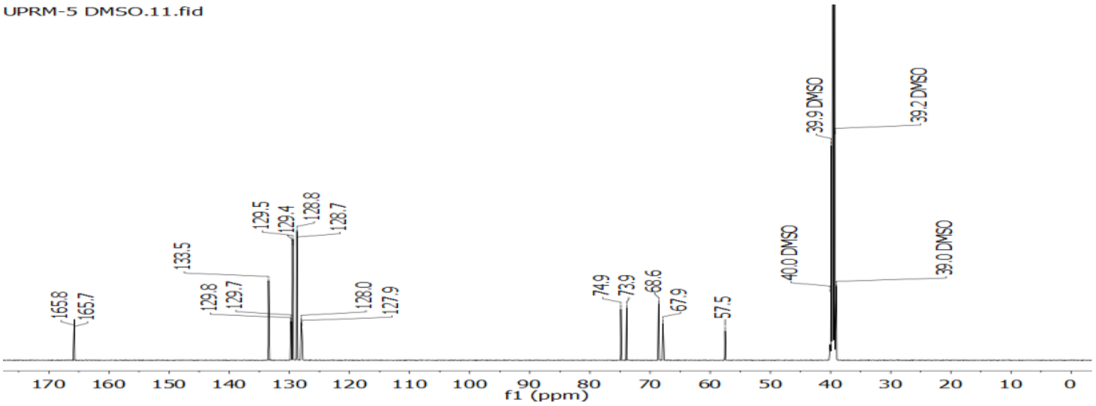

Figure S73. The ^{13}C NMR spectrum of compound **10** (125 MHz, DMSO-d_6)

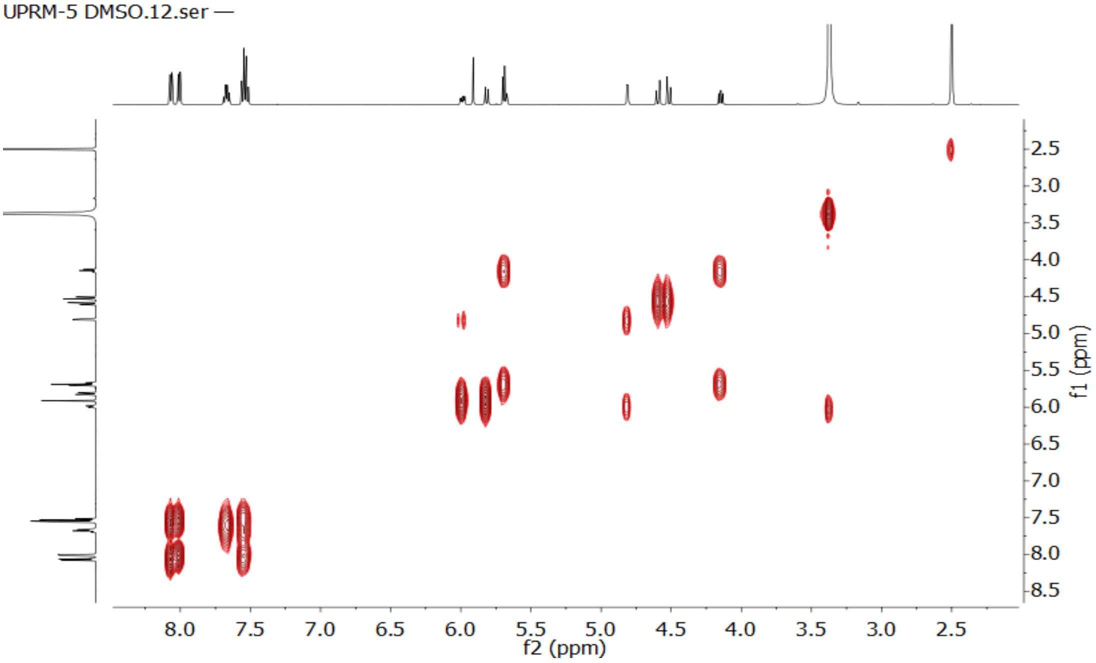

Figure S74. The COSY spectrum of compound **10** (500 MHz, DMSO-d_6)

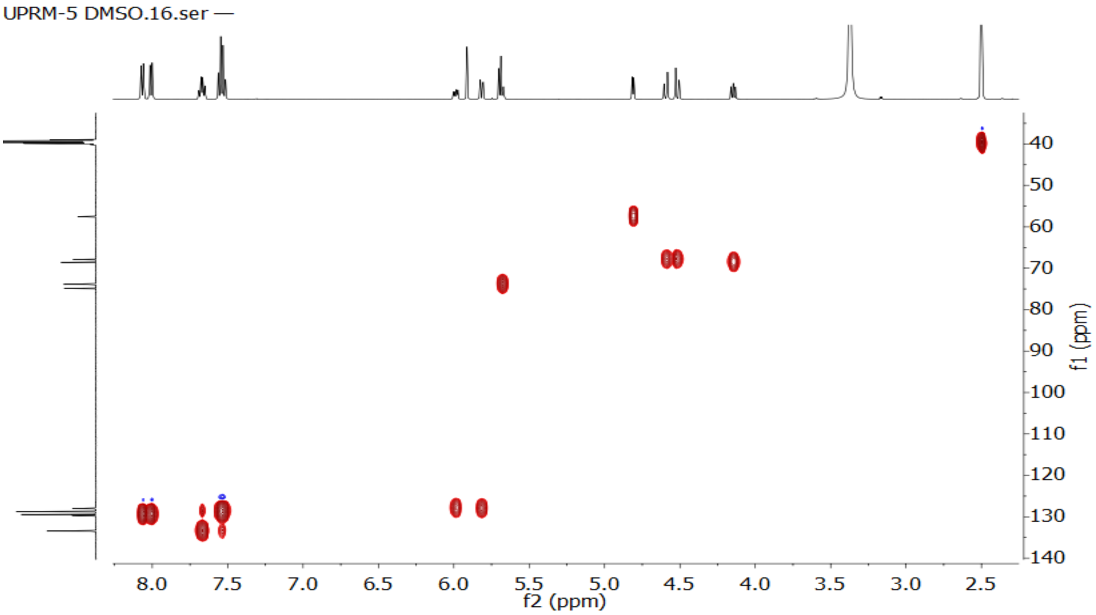

Figure S75. The HSQC spectrum of compound **10** (500/125 MHz, DMSO-d_6)

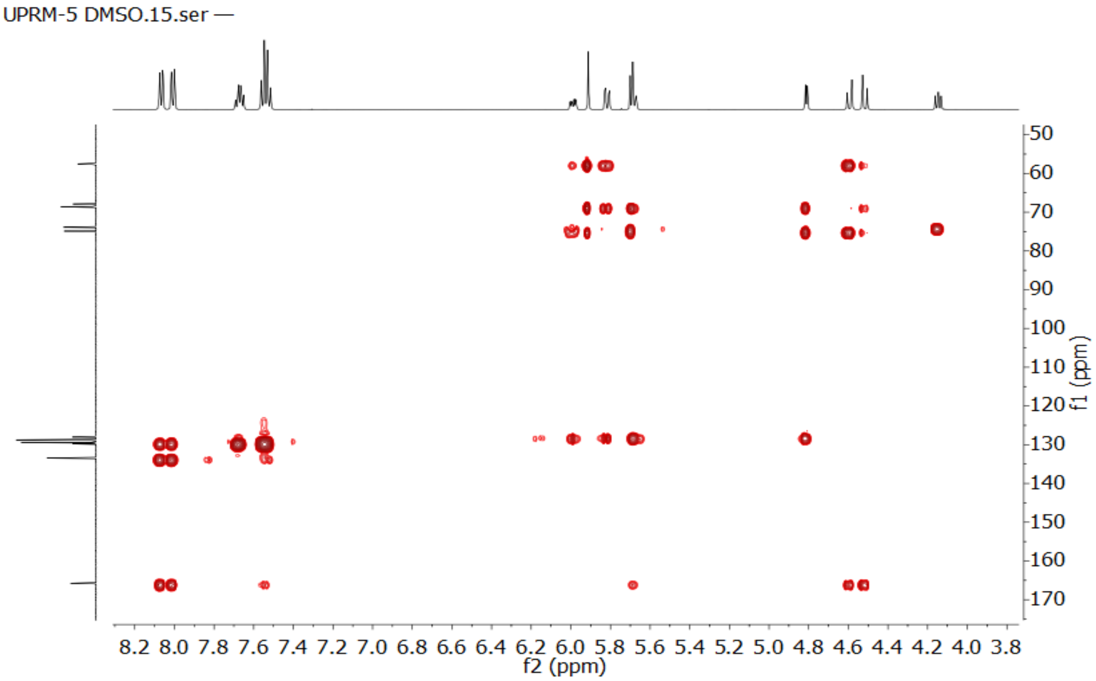

Figure S76. The HMBC spectrum of compound **10** (500/125 MHz, DMSO-d_6)

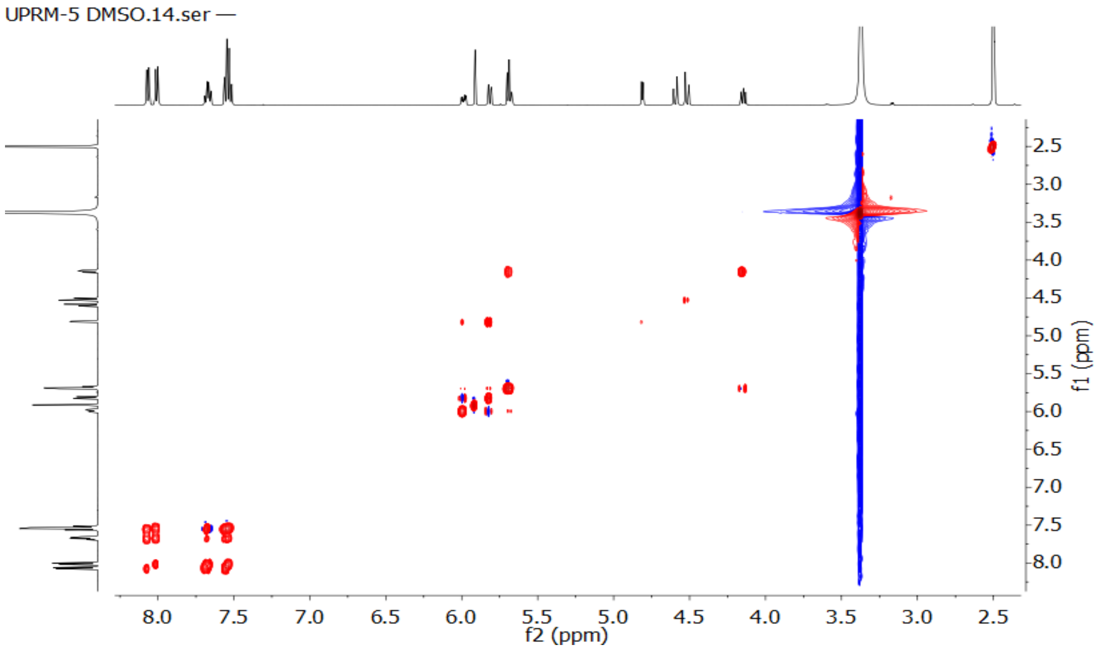

Figure S77. The TOCSY spectrum of compound **10** (500 MHz, DMSO-d$_6$)

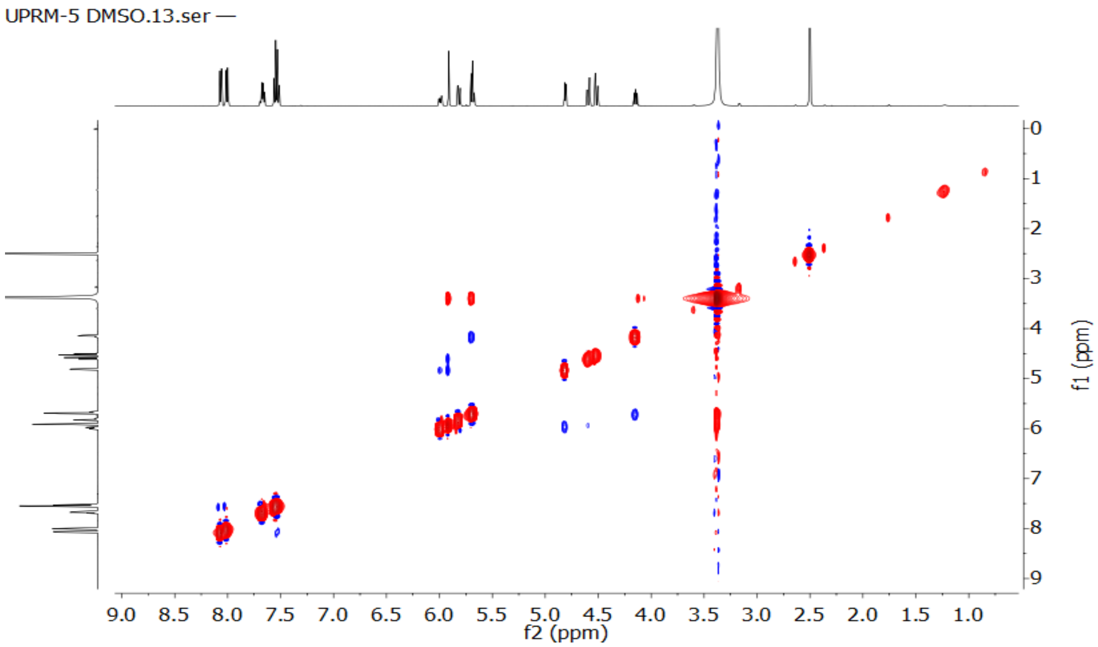

Figure S78: The NOESY spectrum of compound **10** (500 MHz, DMSO-d$_6$)

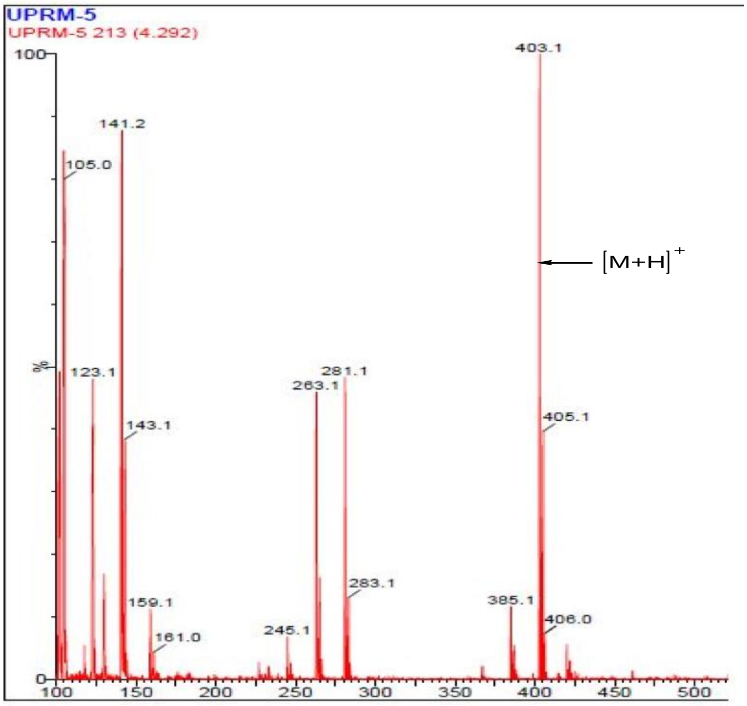

Figure S79. The LC-MS of compound **10**

12. Spectroscopic data for compound 11

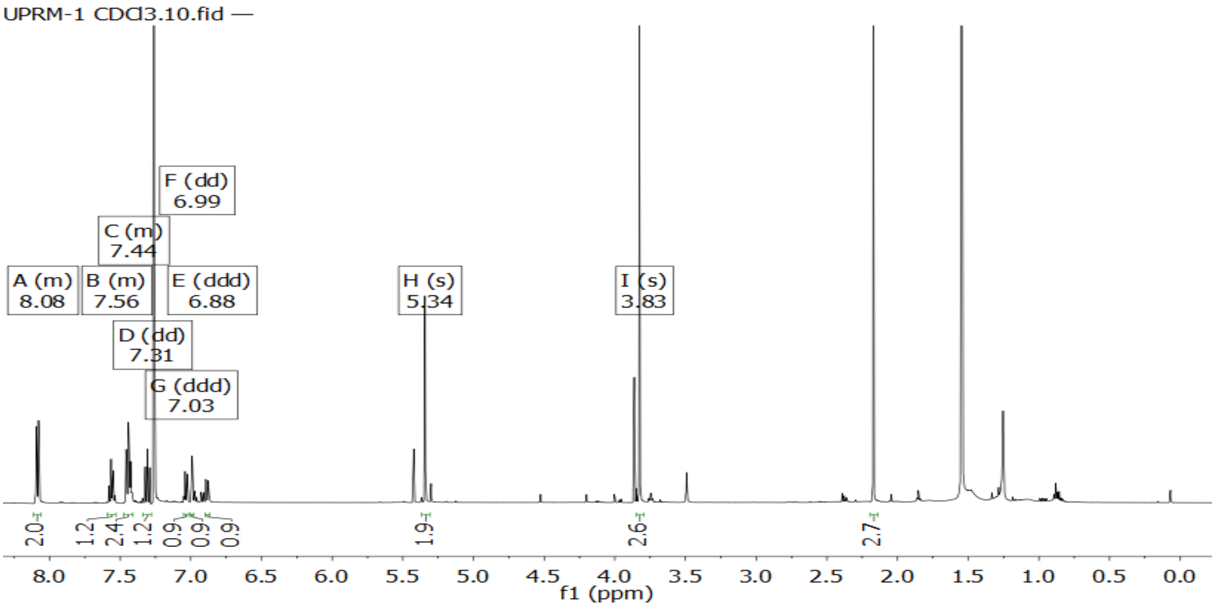

Figure S80. The ^{1}H NMR spectrum of compound **11** (500 MHz, $CDCl_3$)

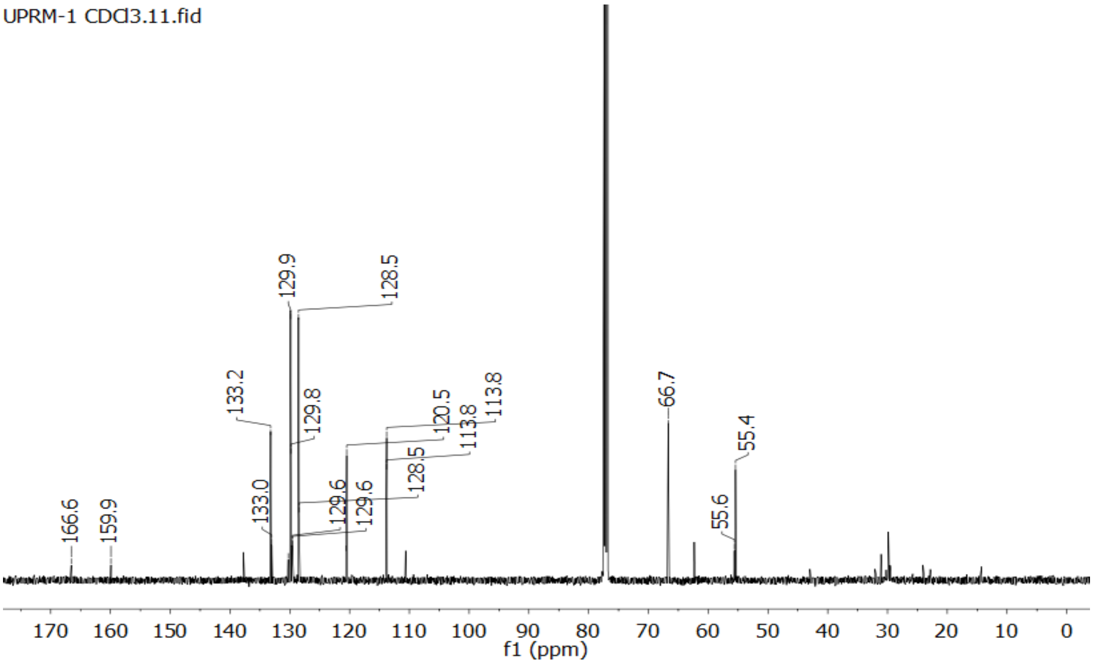

Figure S81. The ^{13}C NMR spectrum of compound **11** (125 MHz, $CDCl_3$)

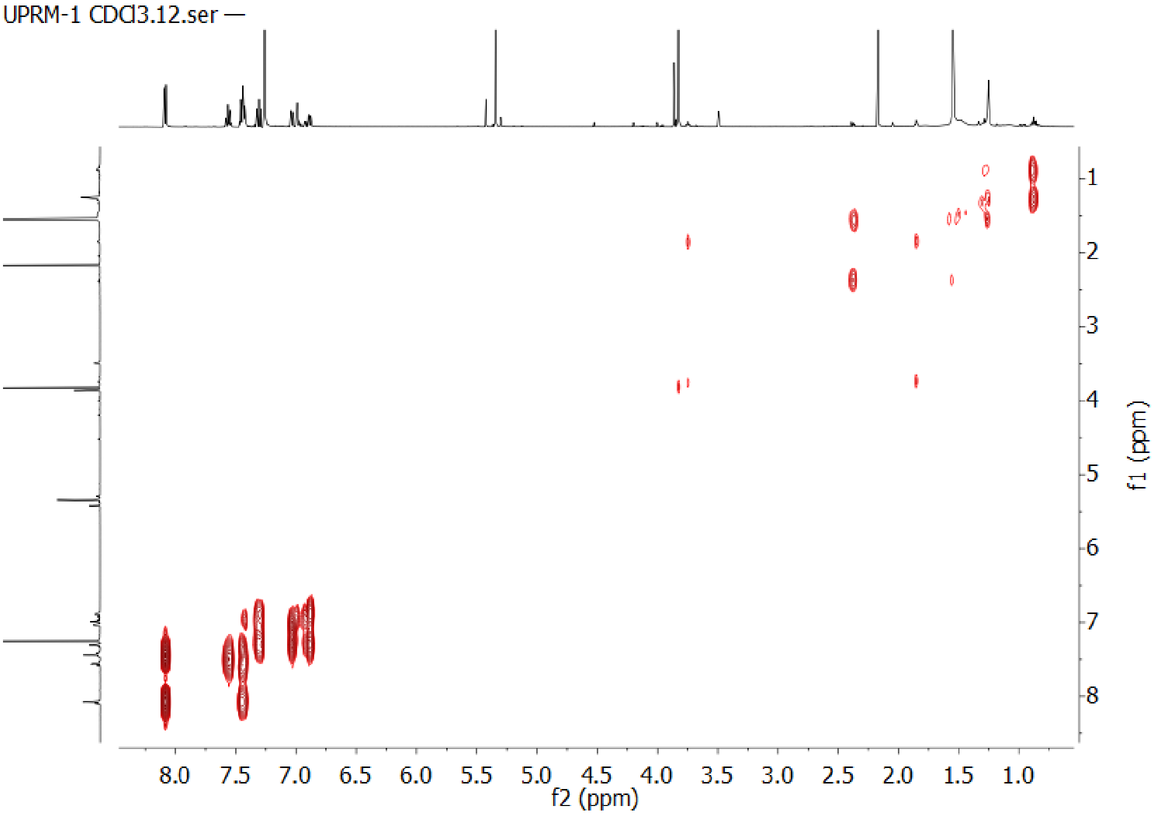

Figure S82. The COSY spectrum of compound **11** (500 MHz, $CDCl_3$)

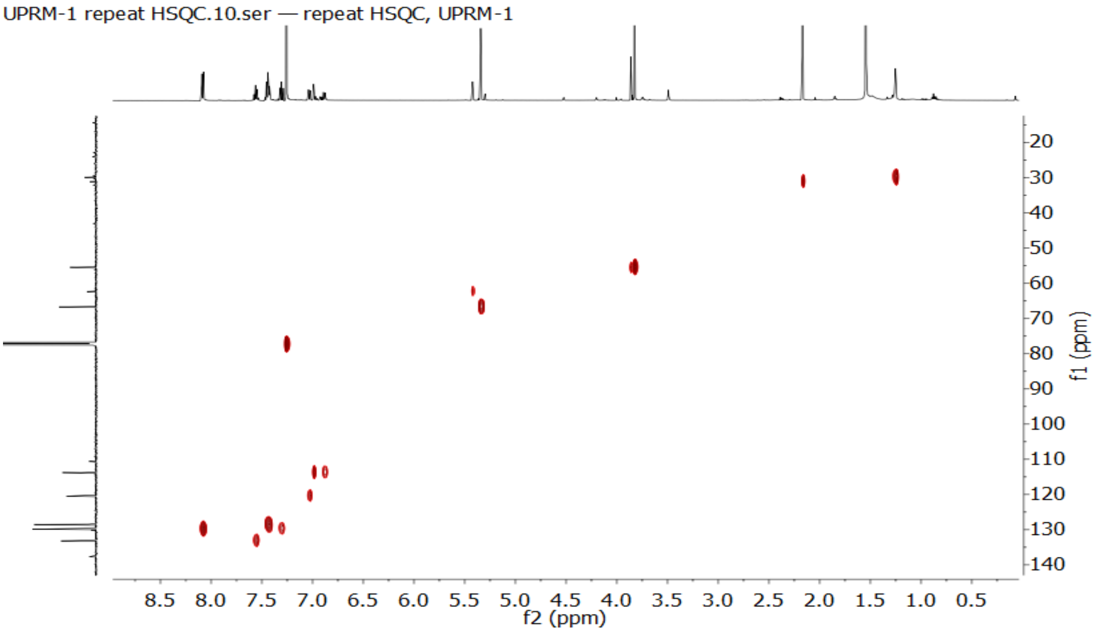

Figure S83. The HSQC spectrum of compound **11** (500/125 MHz, $CDCl_3$)

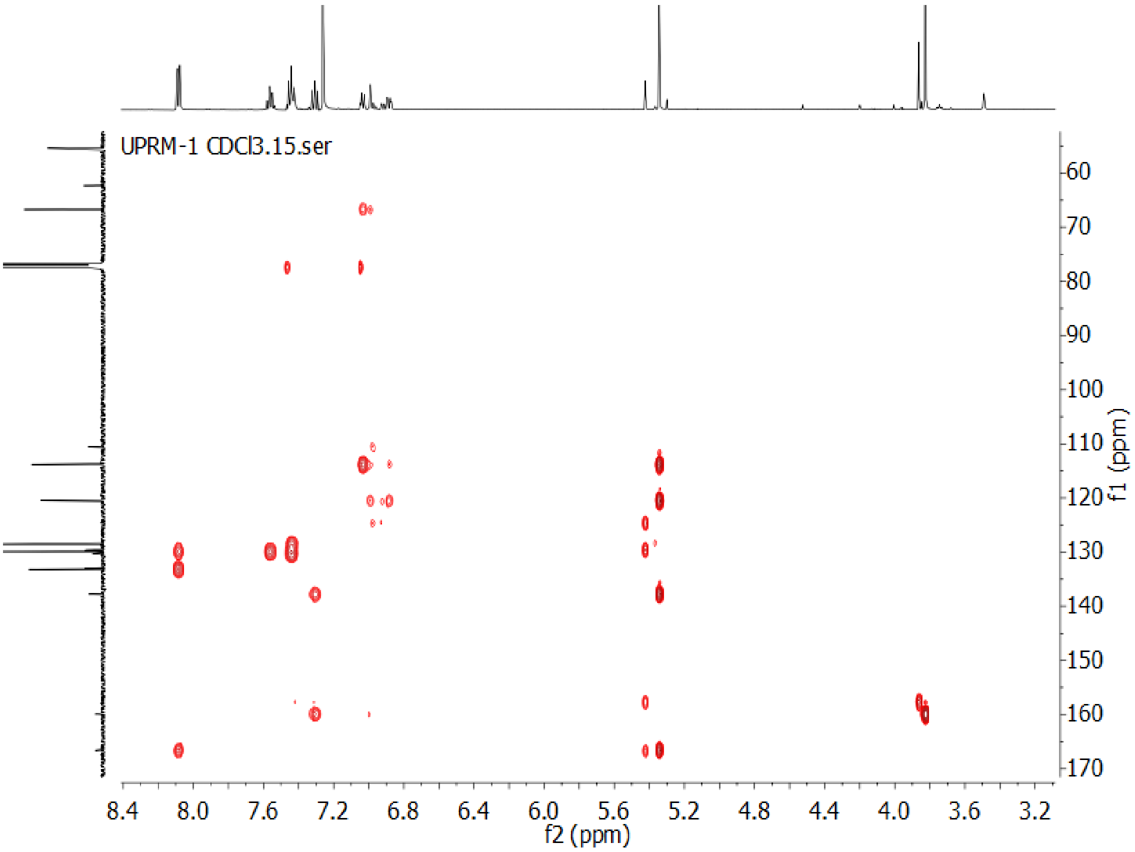

Figure S84. The HMBC spectrum of compound **11** (500/125 MHz, $CDCl_3$)

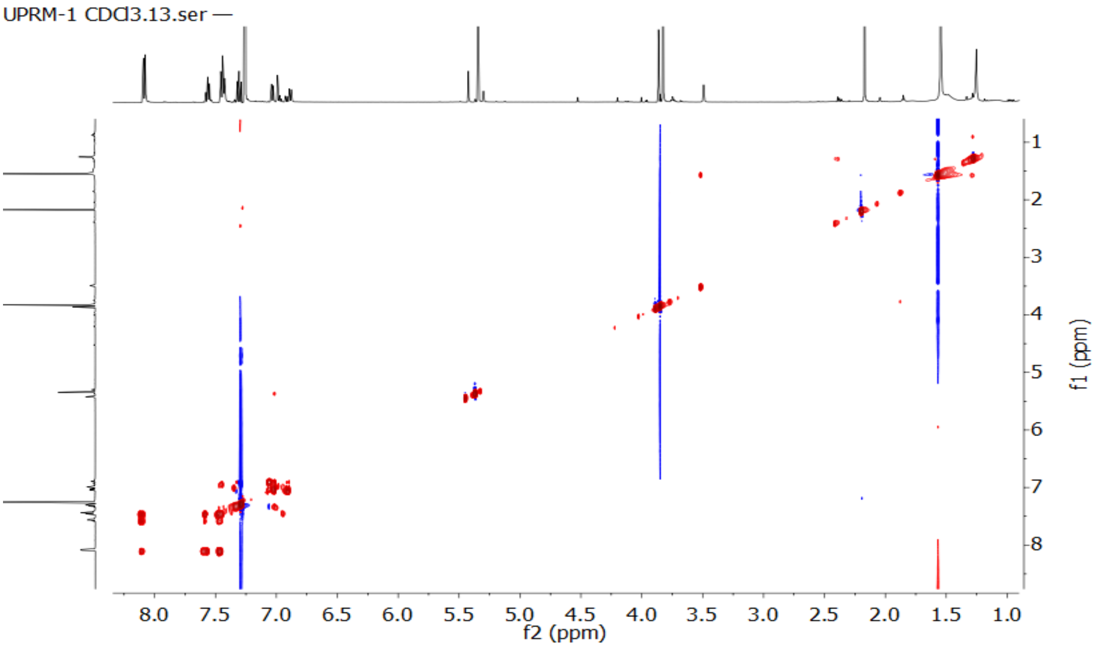

Figure S85. The TOCSY spectrum of compound **11** (500 MHz, $CDCl_3$)

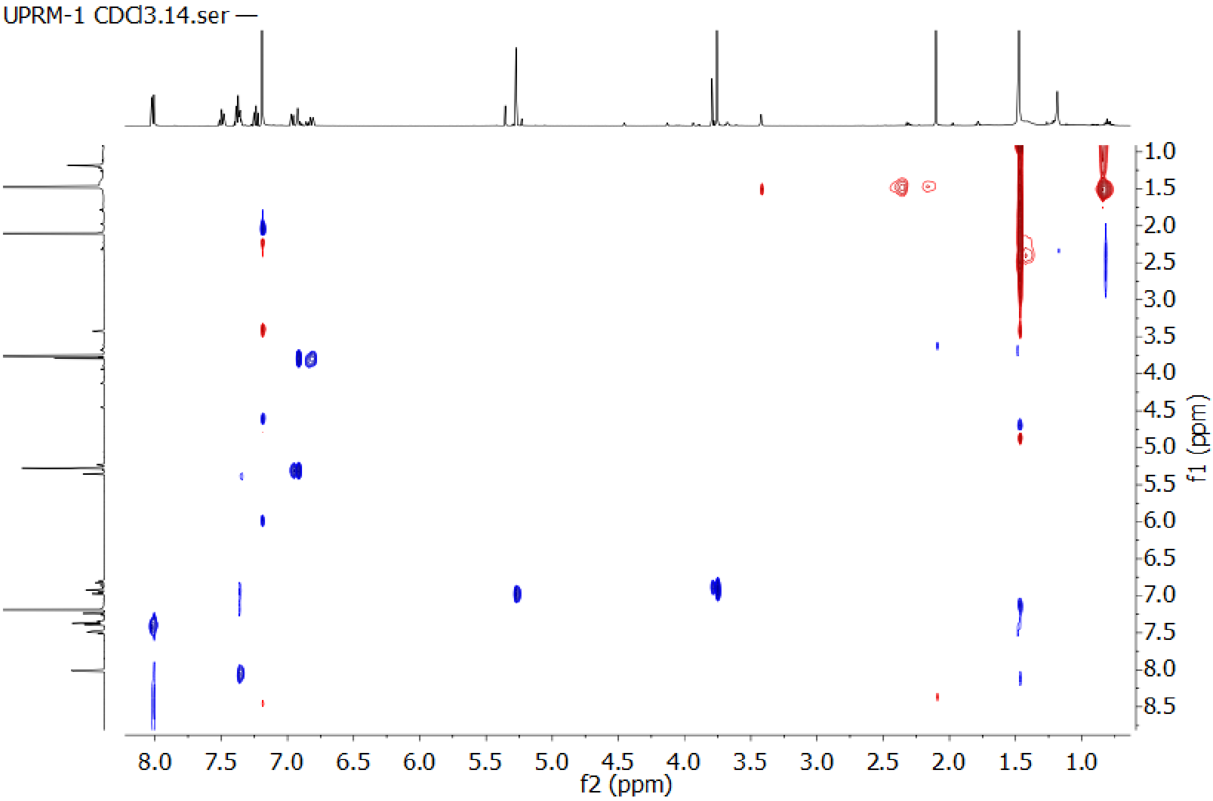

Figure S86. The NOESY spectrum of compound **11** (500 MHz, $CDCl_3$)

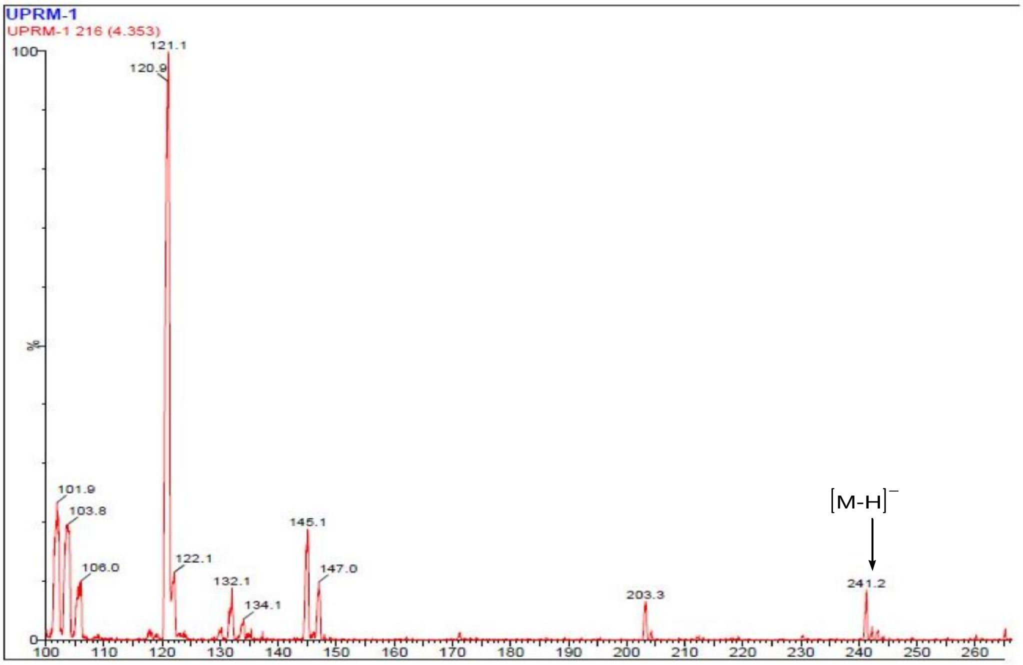

Figure S87. The LC-MS of compound **11**

13. Spectroscopic data for compound 12

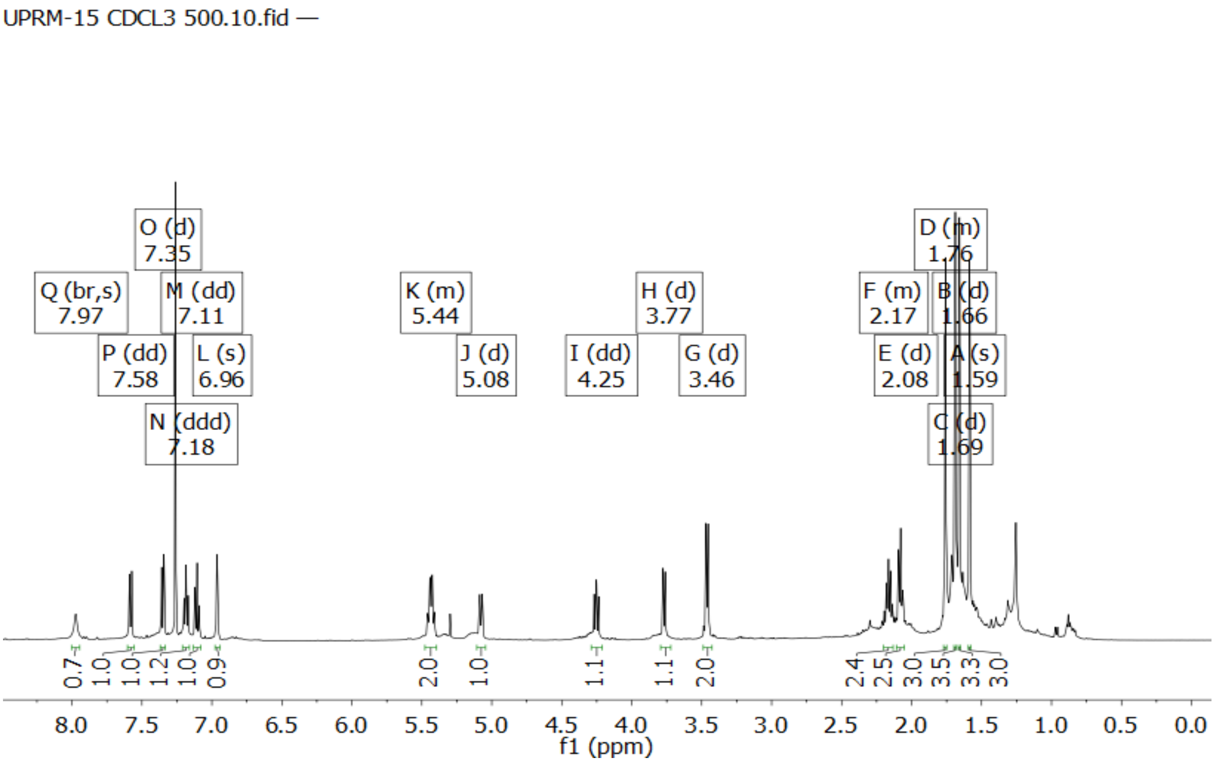

Figure S88. The ^{1}H NMR spectrum of compound **12** (600 MHz, $CDCl_3$)

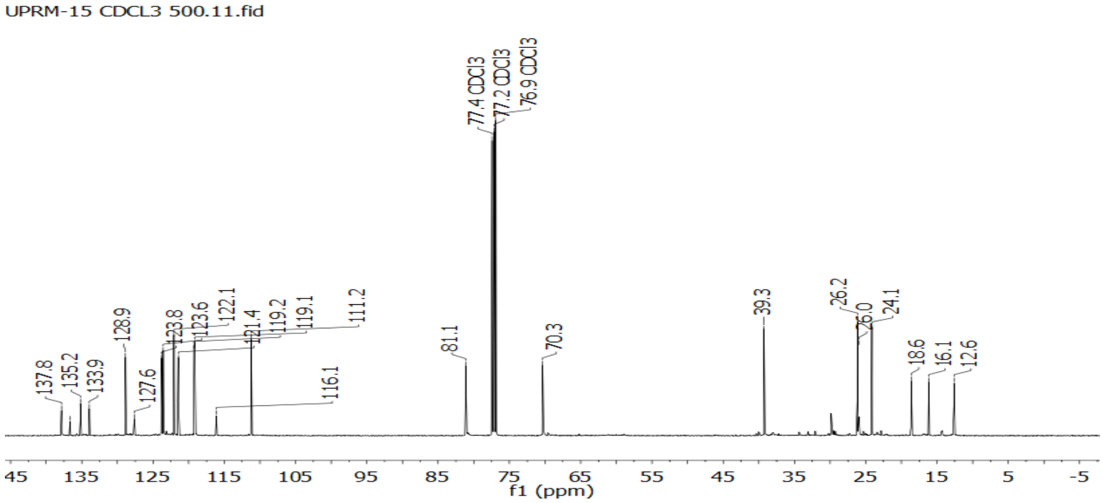

Figure S89. The ^{13}C NMR spectrum of compound **12** (125 MHz, $CDCl_3$)

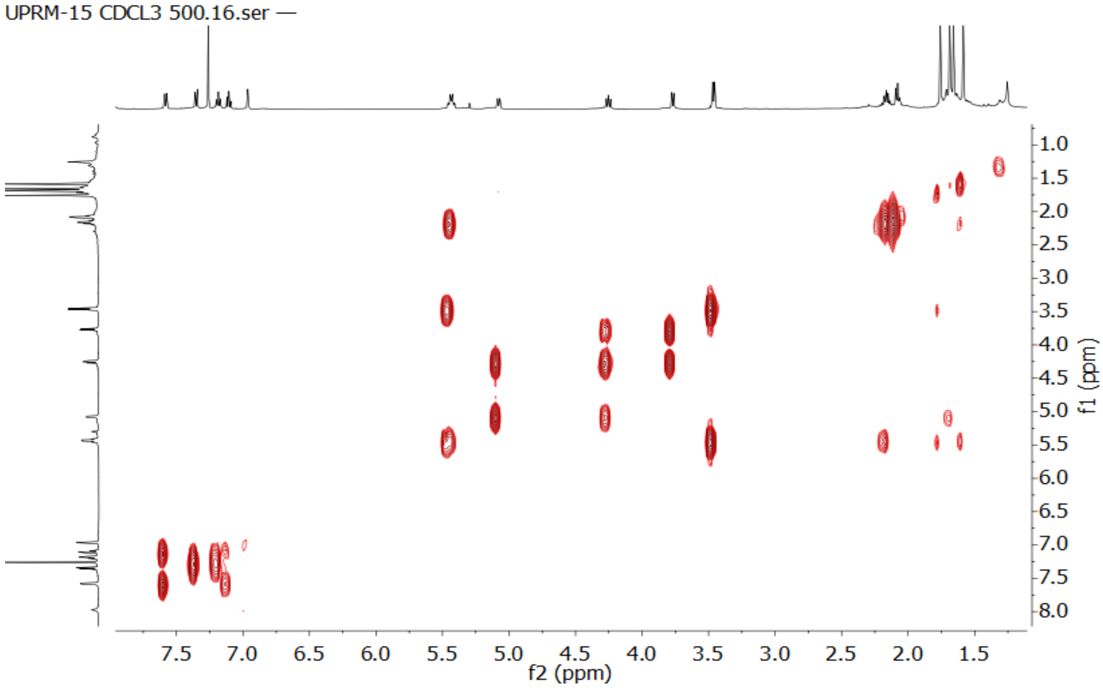

Figure S90. The COSY spectrum of compound **12** (500 MHz, $CDCl_3$)

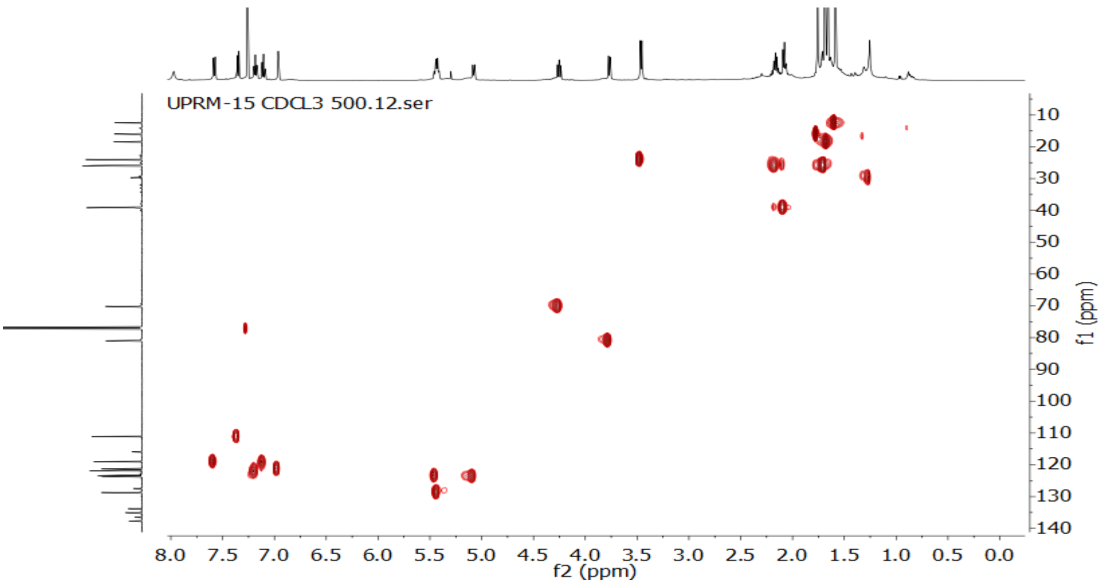

Figure S91. The HSQC spectrum of compound **12** (500/125 MHz, $CDCl_3$)

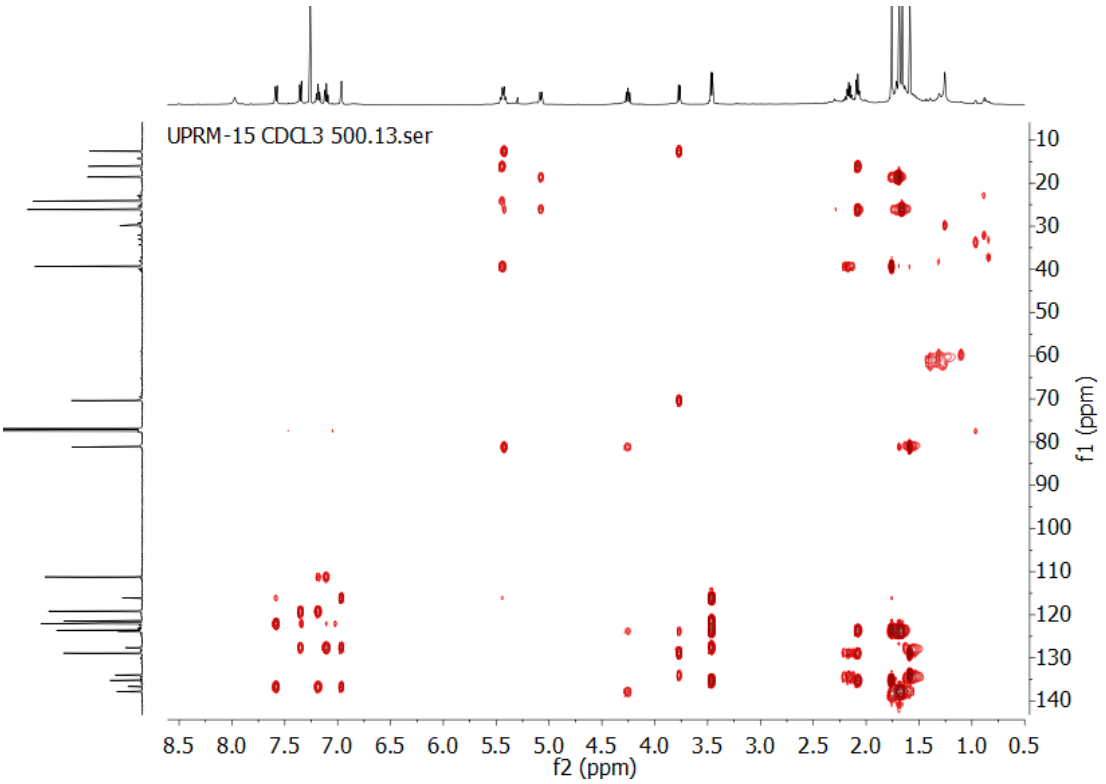

Figure S92. The HMBC spectrum of compound **12** (500/125 MHz, $CDCl_3$)

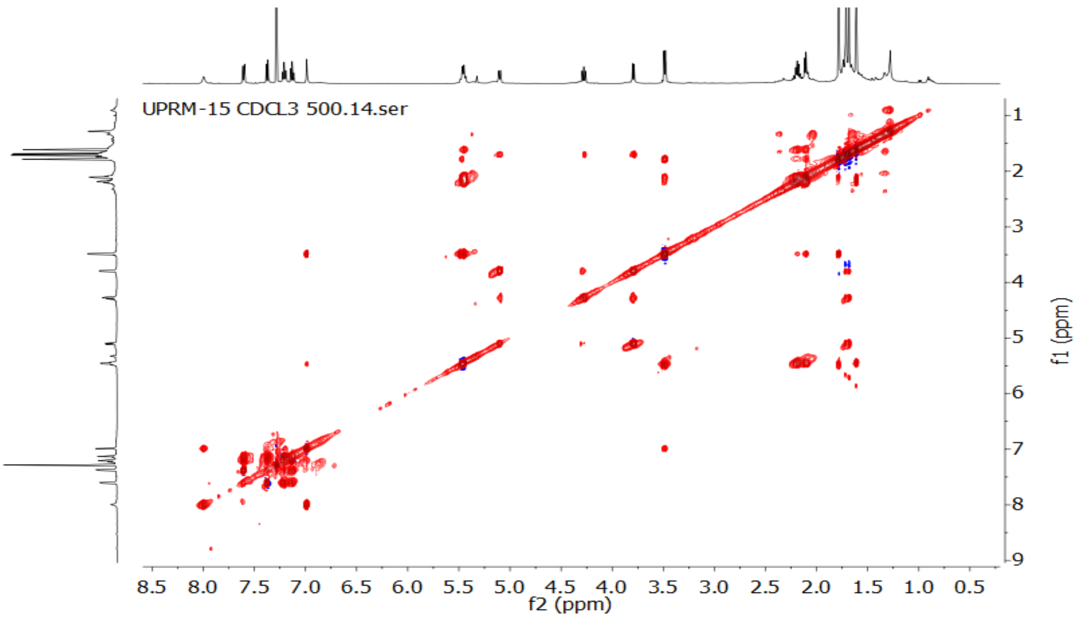

Figure S93. The TOCSY spectrum of compound **12** (500 MHz, $CDCl_3$)

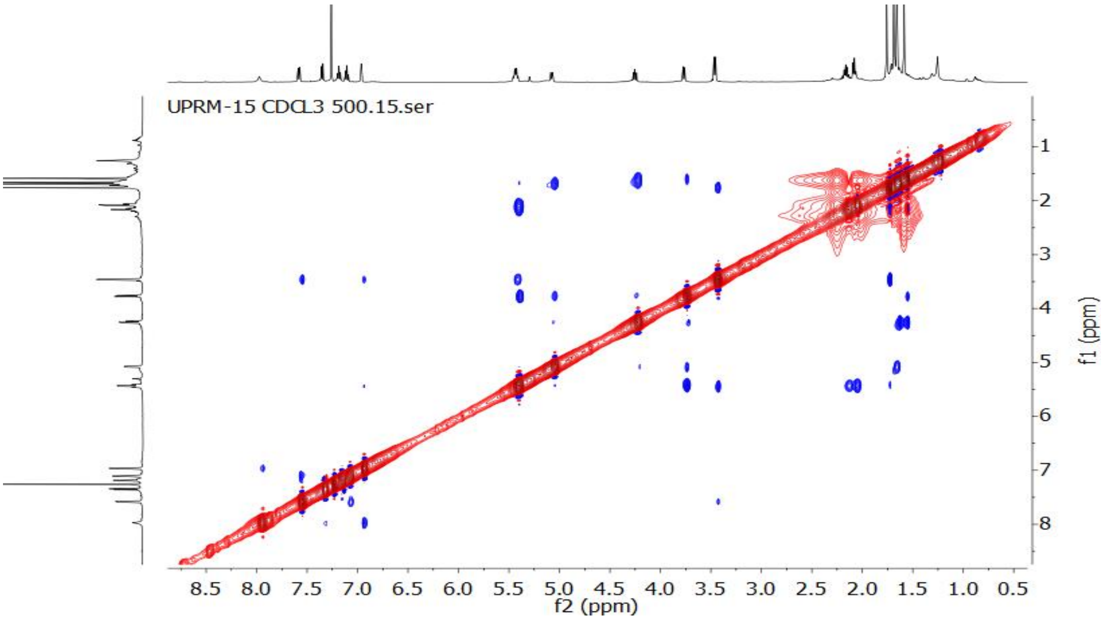

Figure S94. The NOESY spectrum of compound **12** (500 MHz, $CDCl_3$)

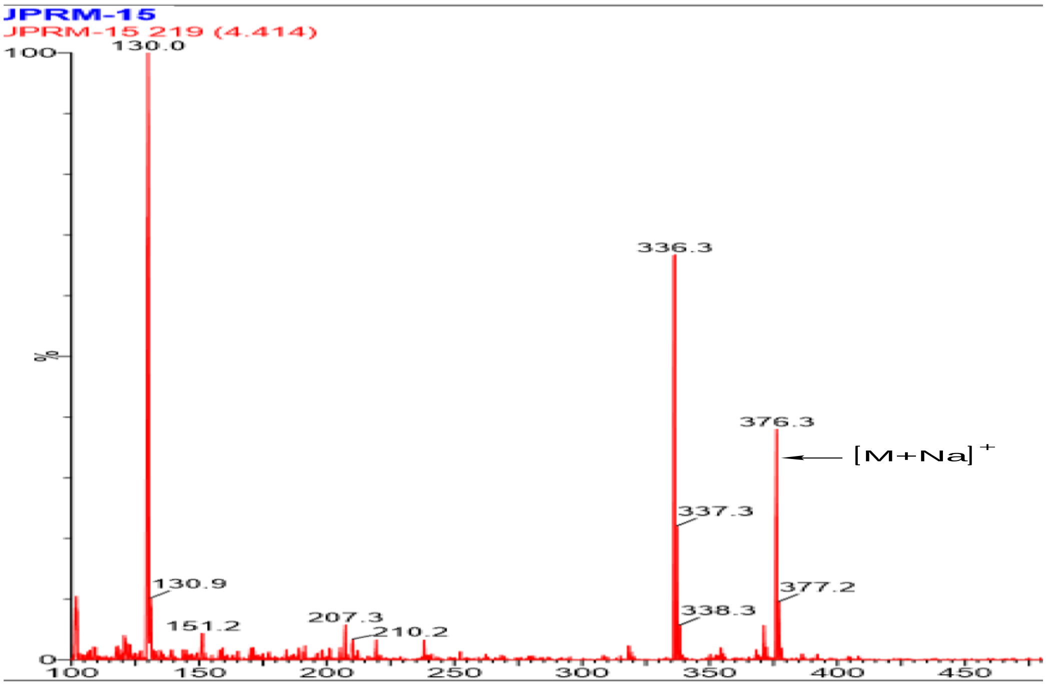

Figure S95. The LC-MS of compound **12**

14. Spectroscopic data for compound 13

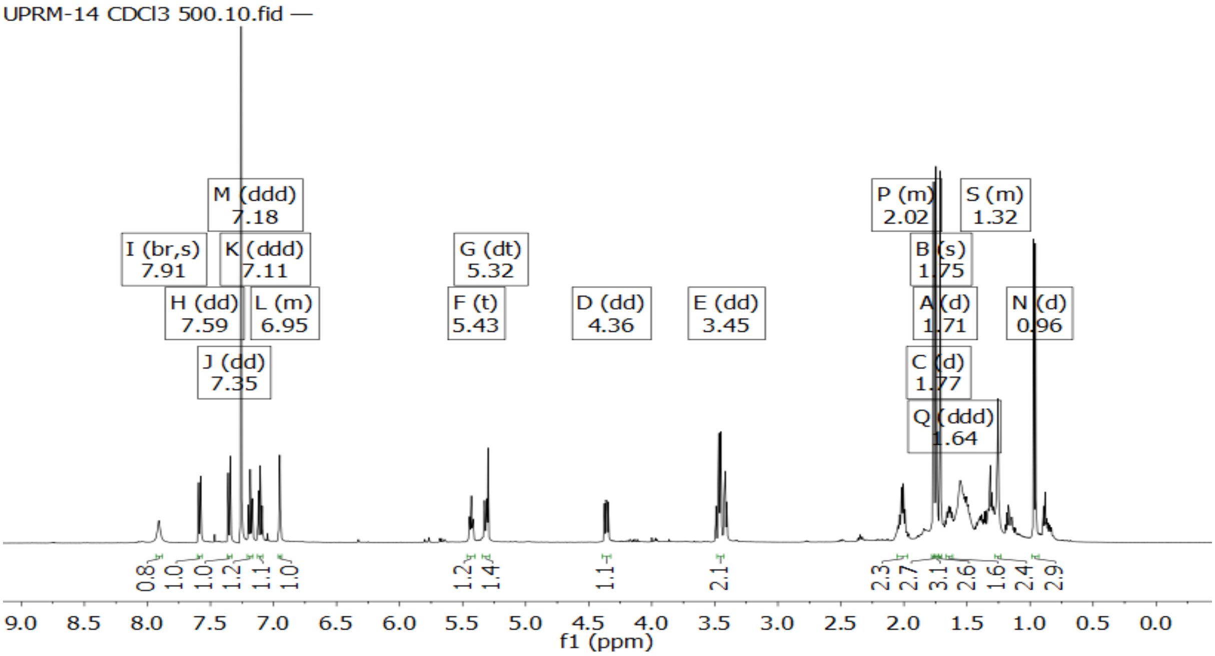

Figure S96. The ^{1}H NMR spectrum of compound **13** (500 MHz, $CDCl_3$)

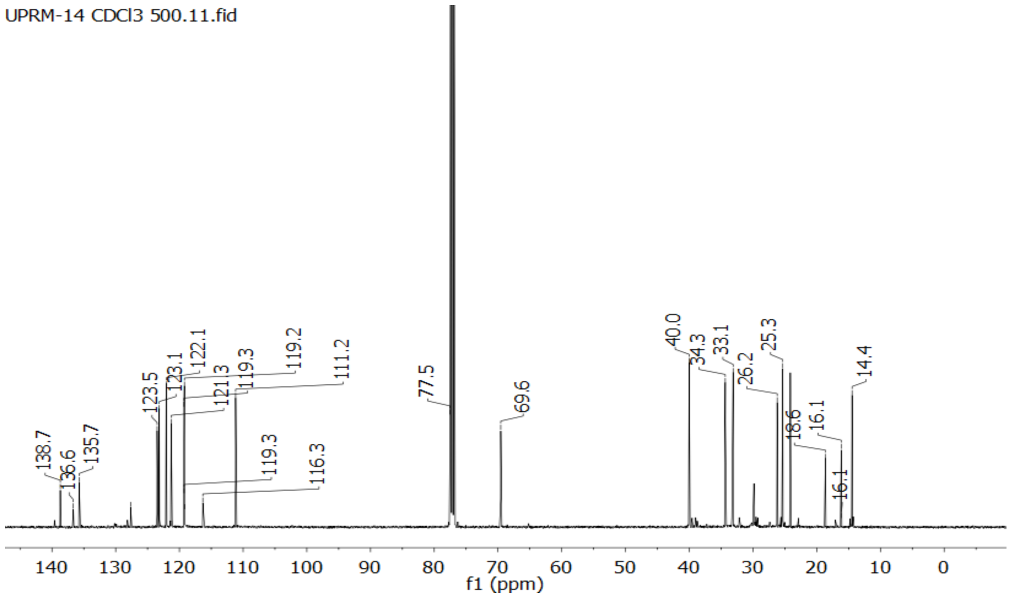

Figure S97. The ^{13}C NMR spectrum of compound **13** (125 MHz, $CDCl_3$)

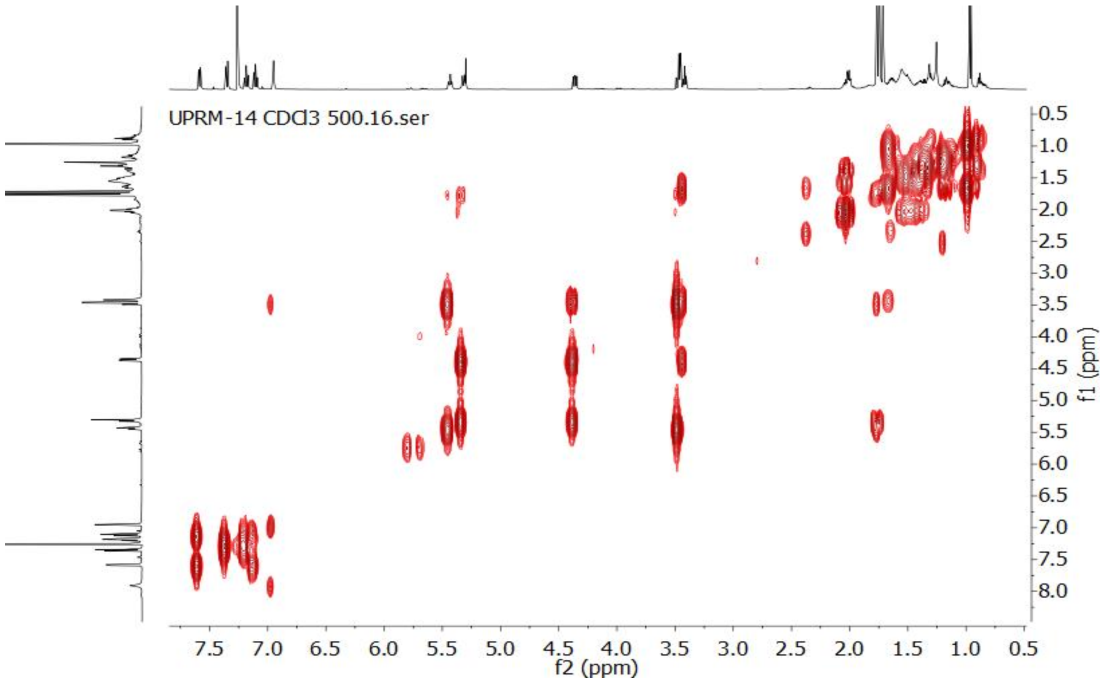

Figure S98. The COSY spectrum of compound **13** (500 MHz, $CDCl_3$)

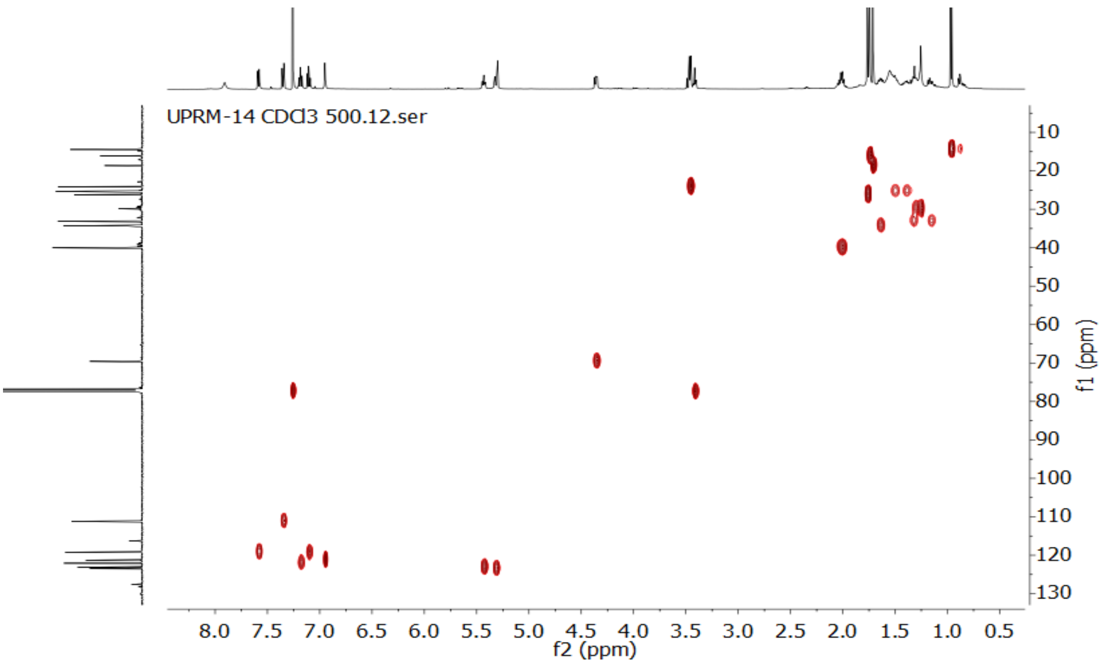

Figure S99. The HSQC spectrum of compound **13** (500/125 MHz, $CDCl_3$)

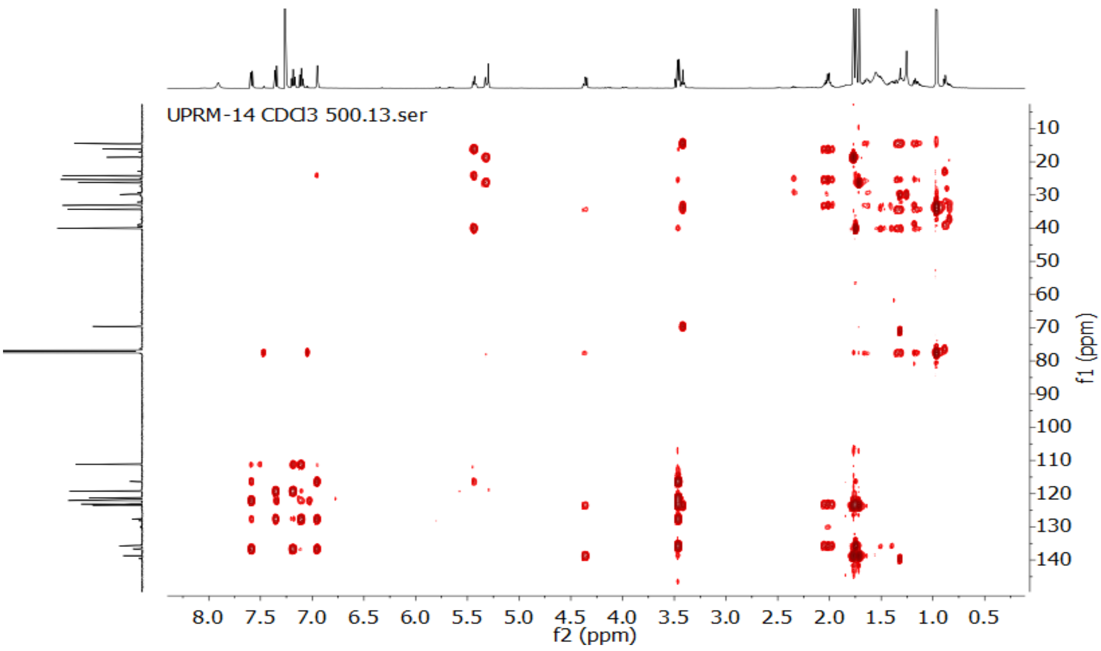

Figure S100. The HMBC spectrum of compound **13** (500/125 MHz, $CDCl_3$)

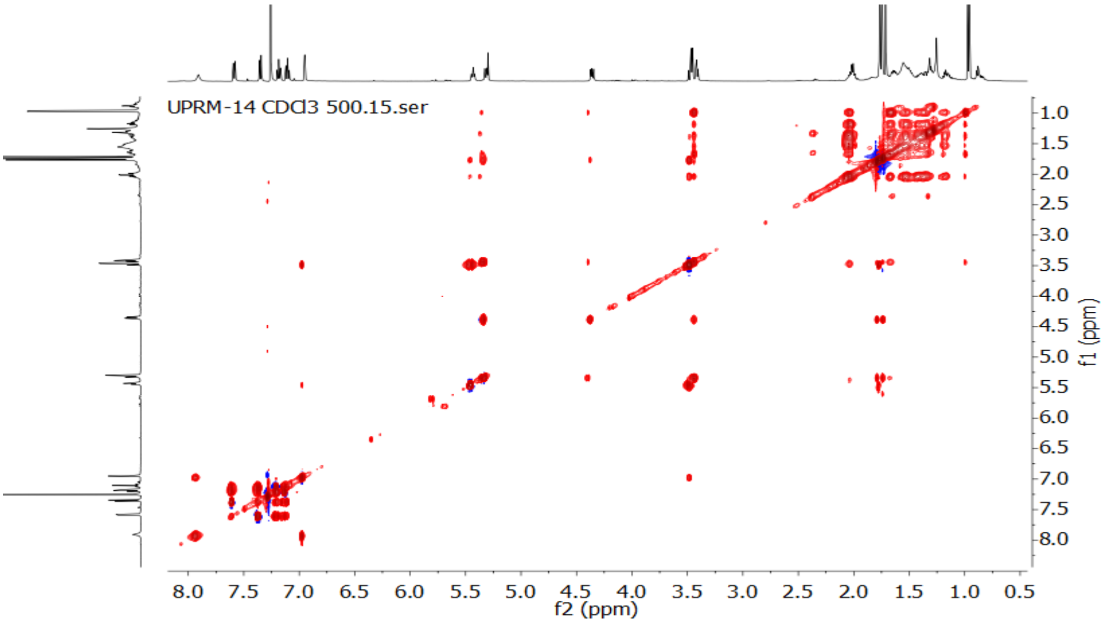

Figure S101. The TOCSY spectrum of compound **13** (500 MHz, $CDCl_3$)

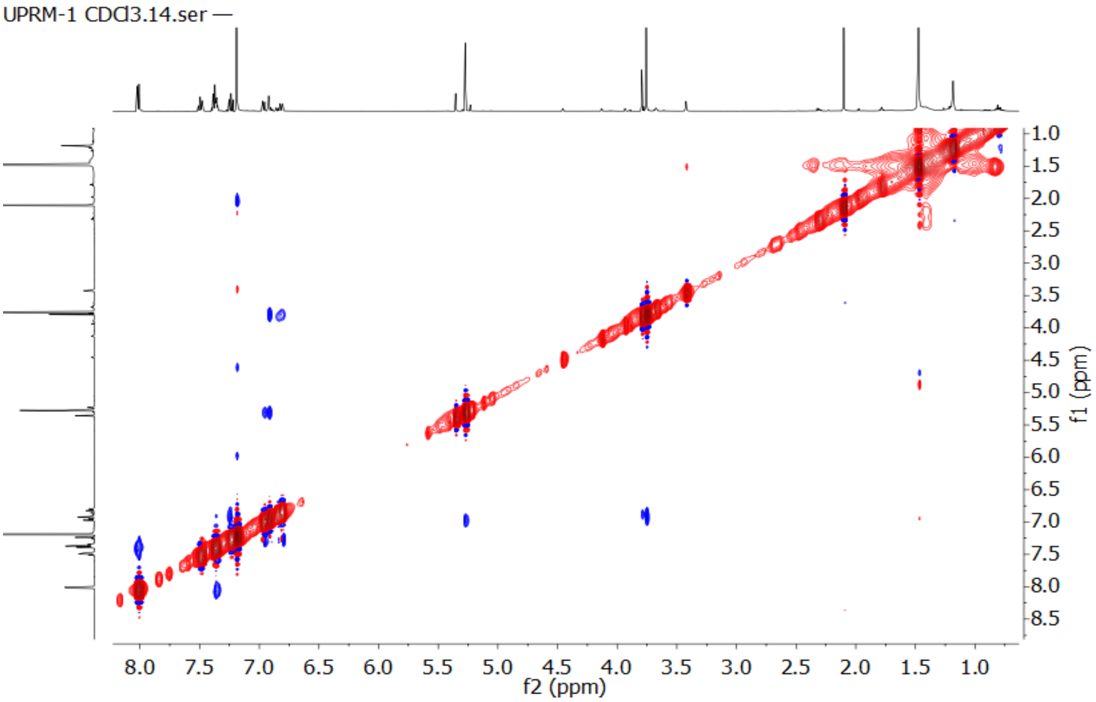

Figure S102. The NOESY spectrum of compound **13** (500 MHz, $CDCl_3$)

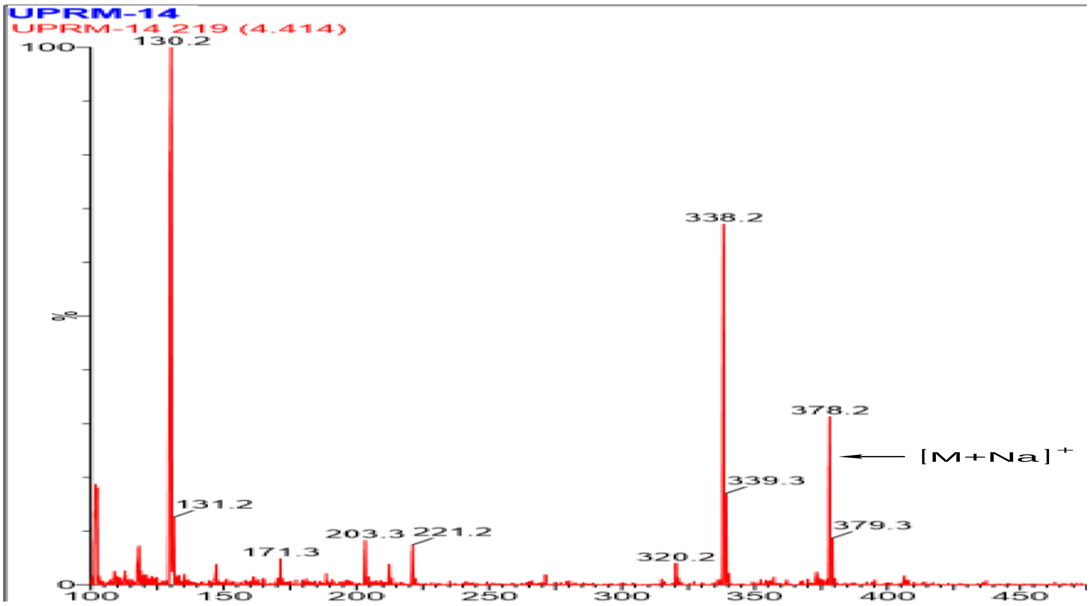

Figure S103. The LC-MS of compound **13**

15. Spectroscopic data for compound 14

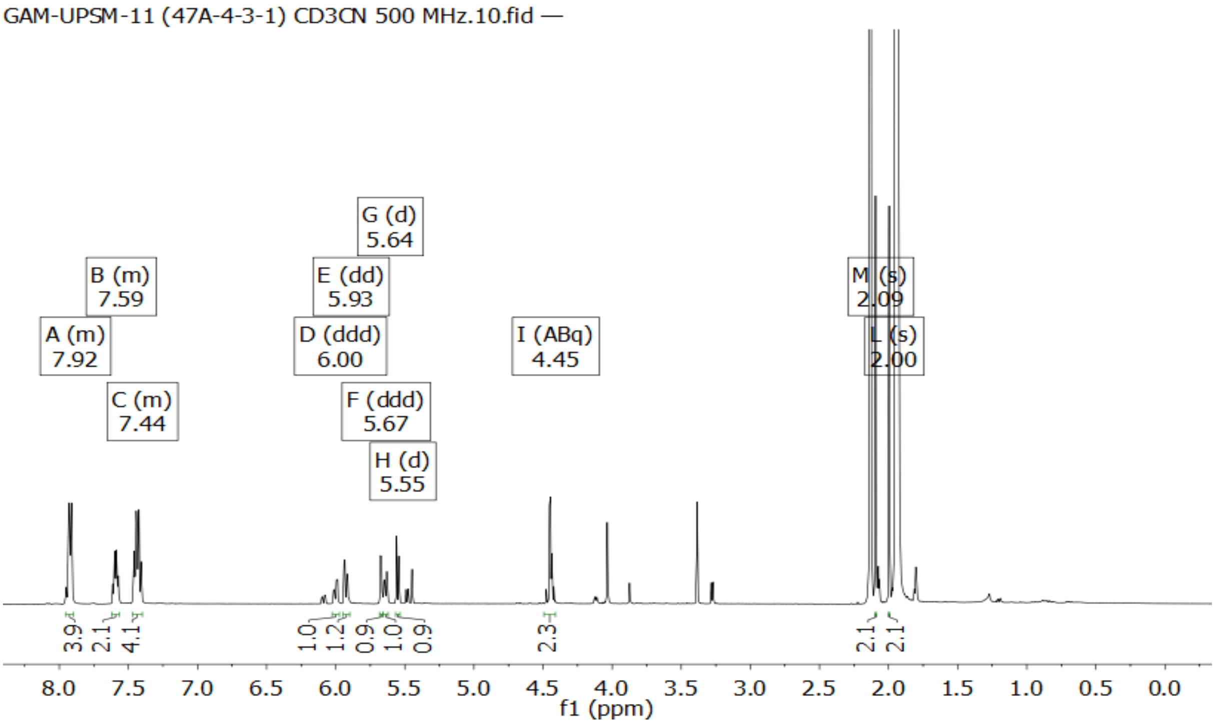

Figure S104. The ^{1}H NMR spectrum of compound **14** (500 MHz, CD_3CN)

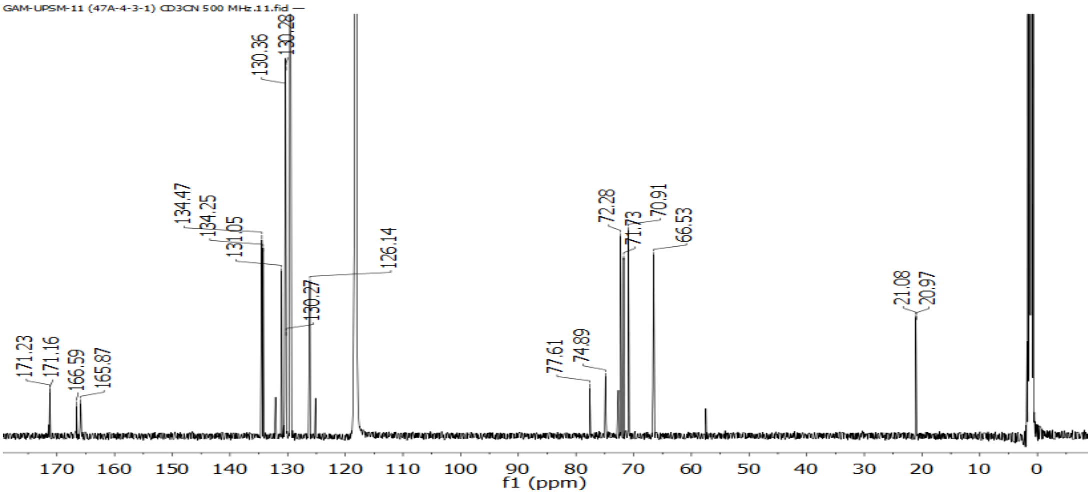

Figure S105. The ^{13}C NMR spectrum of compound **14** (125 MHz, CD_3CN)

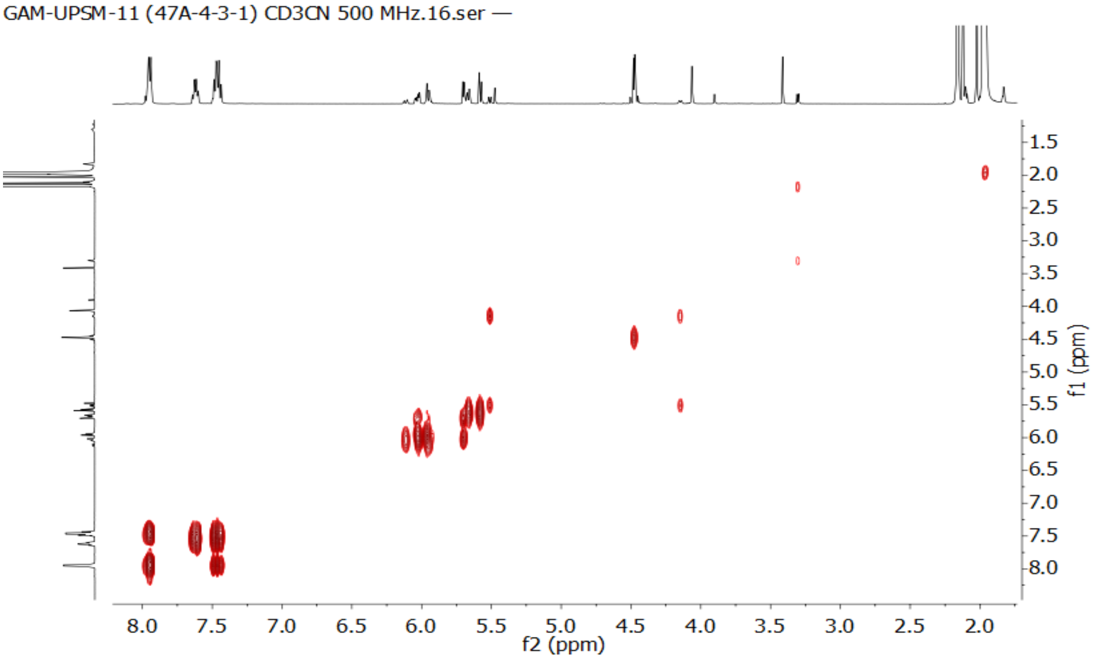

Figure S106. The COSY spectrum of compound **14** (500 MHz, CD_3CN)

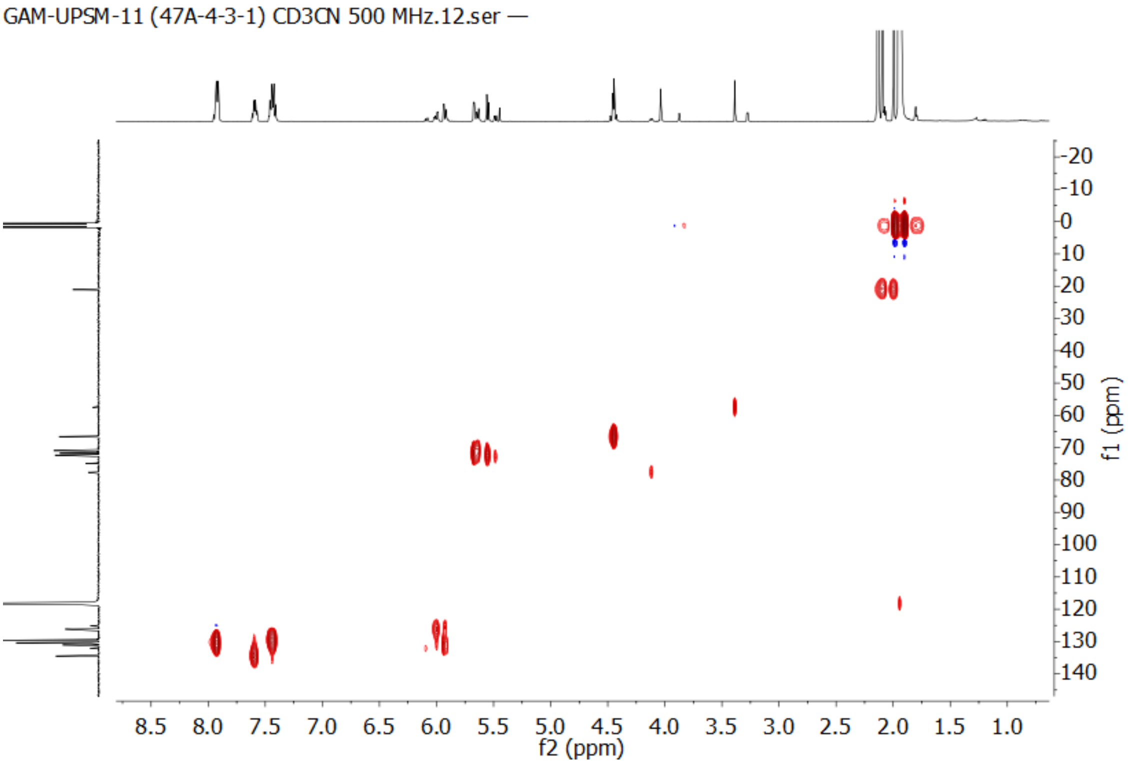

Figure S107. The HSQC spectrum of compound **14** (500/125 MHz, CD_3CN)

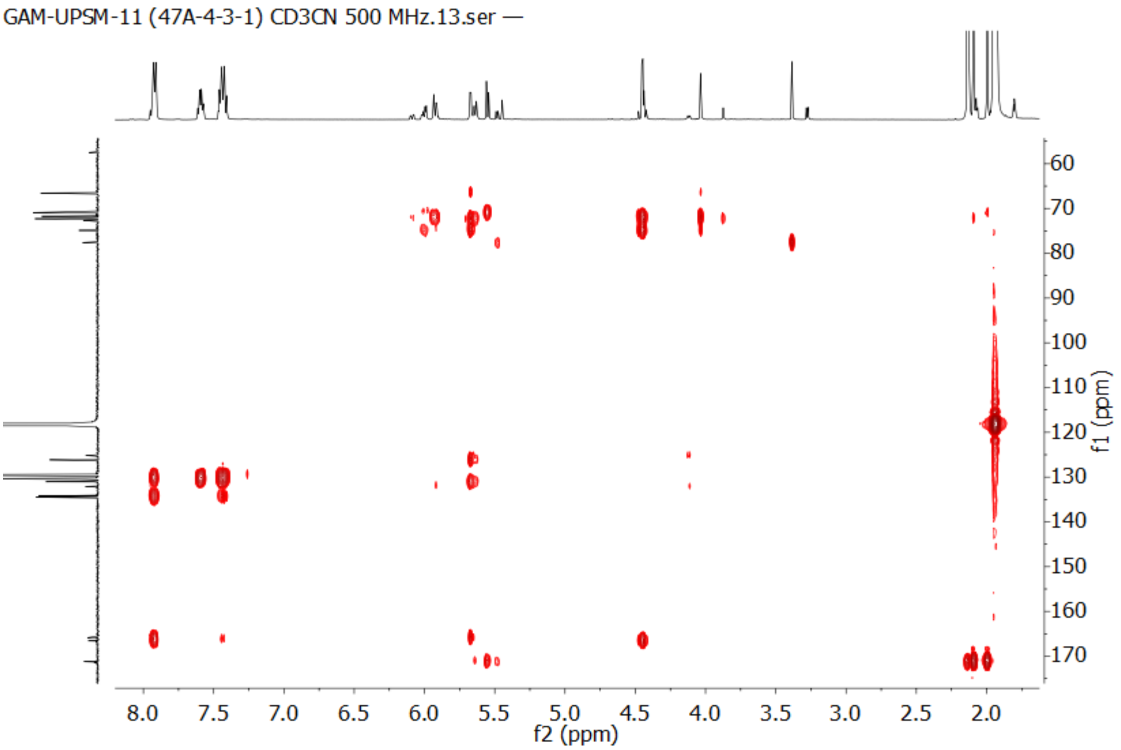

Figure S108. The HMBC spectrum of compound **14** (500/125 MHz, CD_3CN)

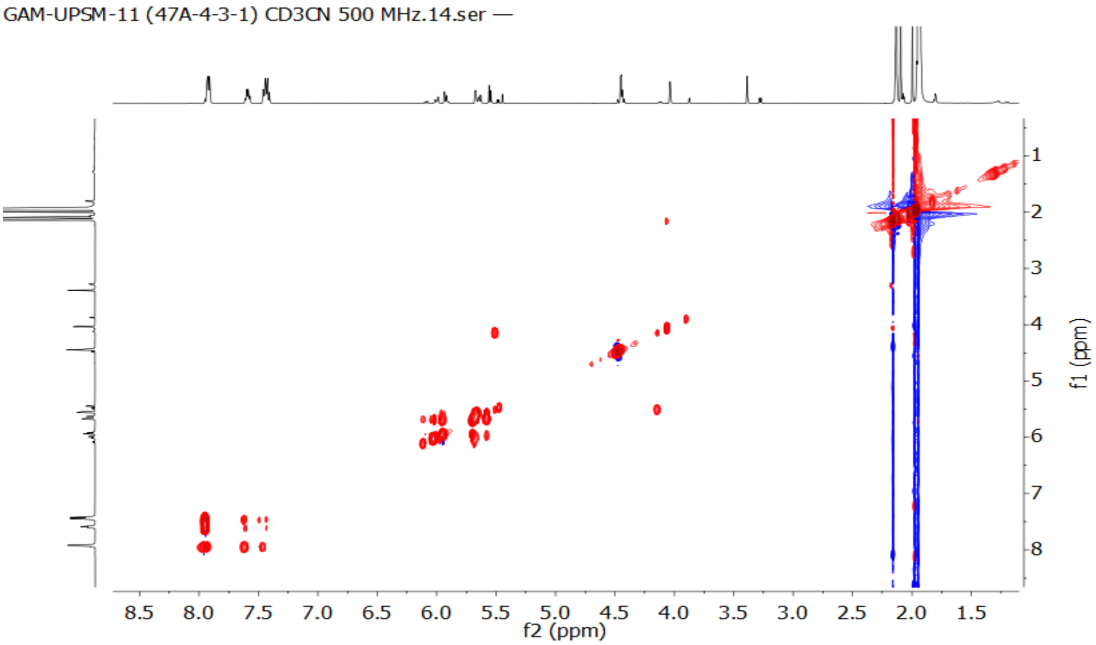

Figure S109. The TOCSY spectrum of compound **14** (500 MHz, CD_3CN)

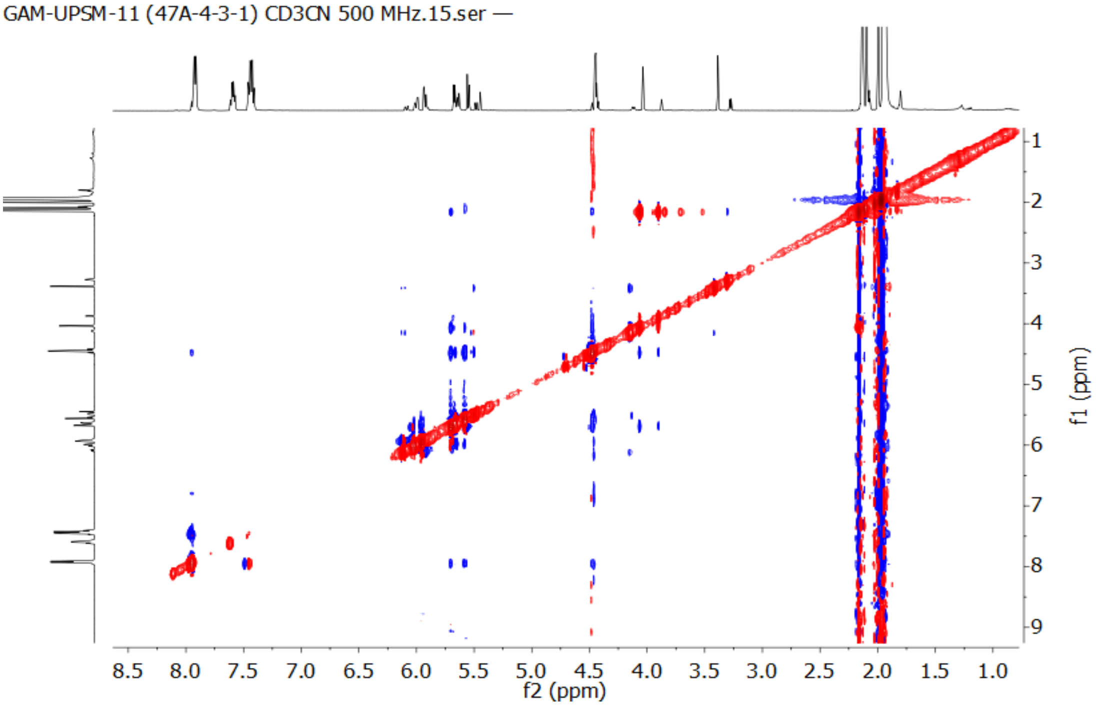

Figure S110. The NOESY spectrum of compound **14** (500 MHz, CD_3CN)

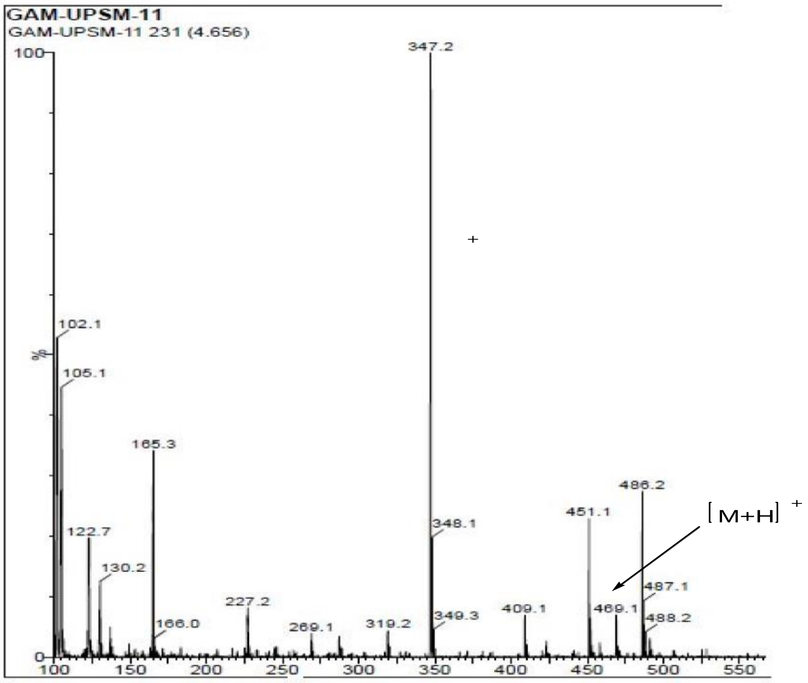

Figure S111. The LC-MS of compound **14**

16. Spectroscopic data for compound 15

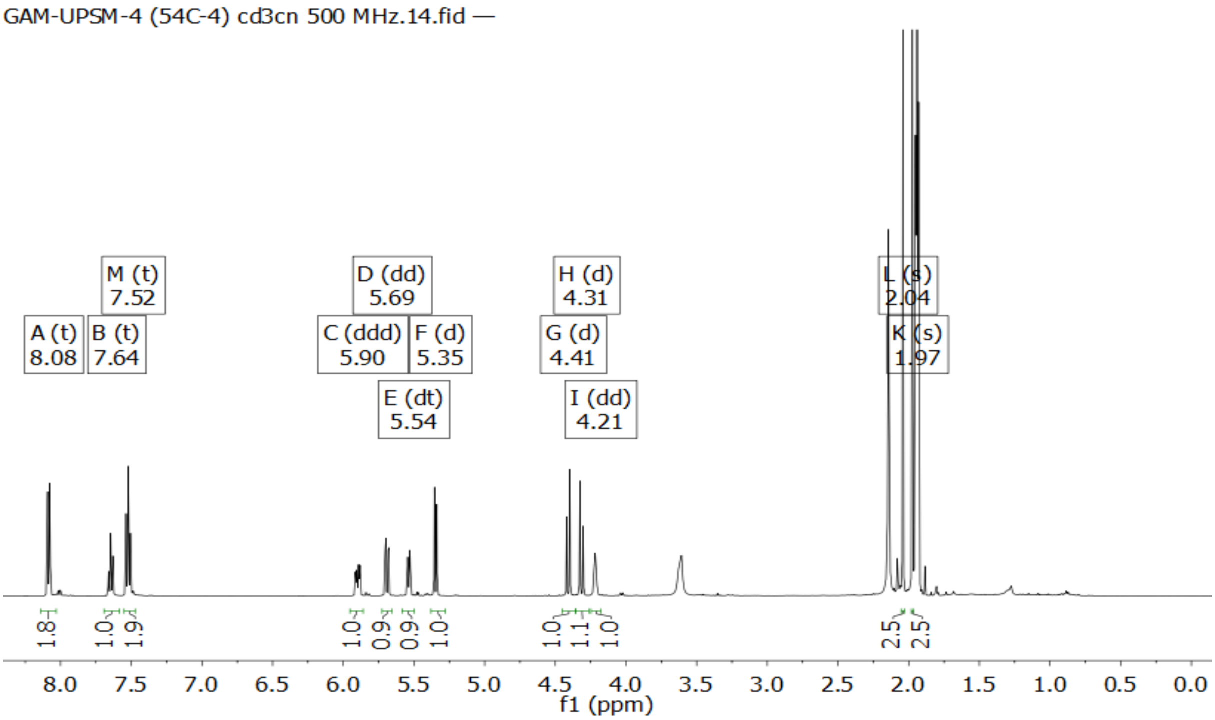

Figure S112. The ^{1}H NMR spectrum of compound **15** (500 MHz, CD_3CN)

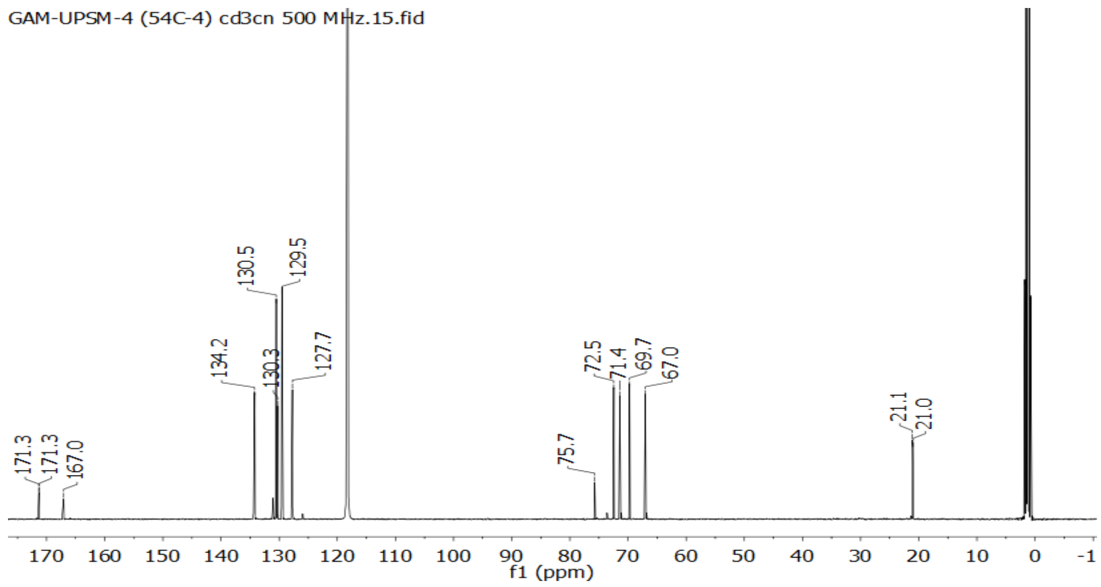

Figure S113. The ^{13}C NMR spectrum of compound **15** (125 MHz, CD_3CN)

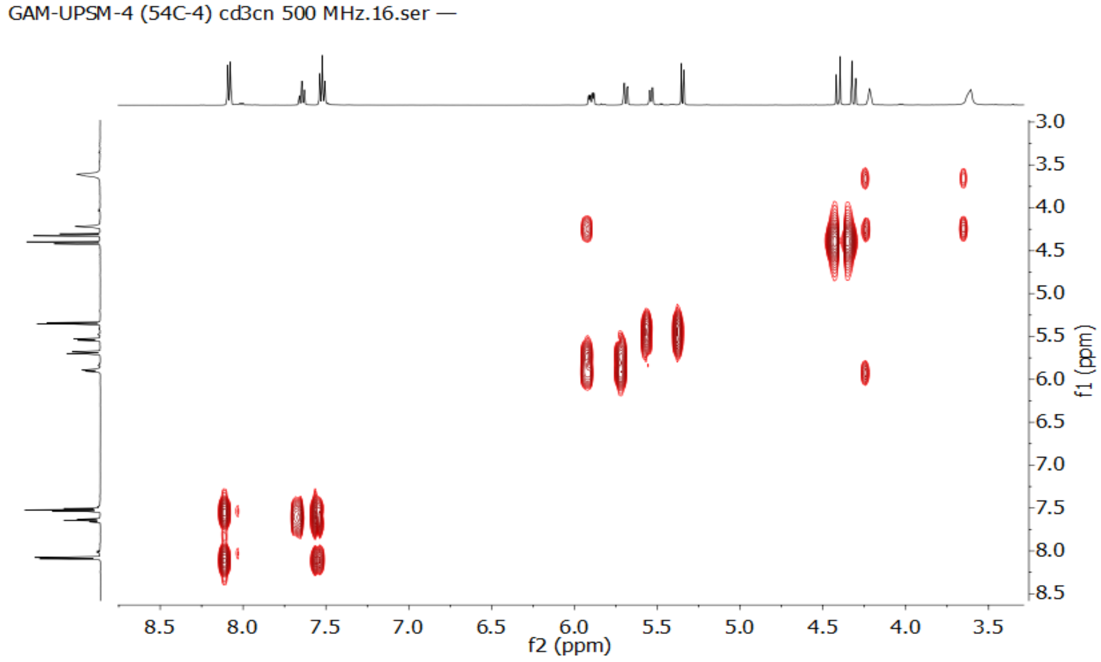

Figure S114. The COSY spectrum of compound **15** (500 MHz, CD_3CN)

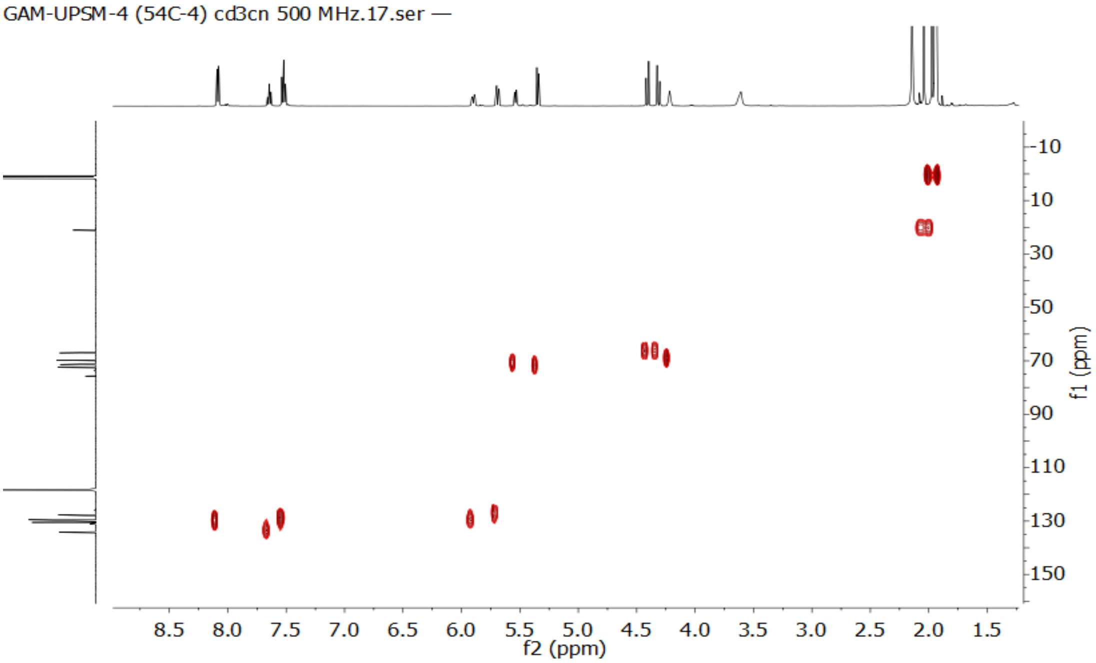

Figure S115. The HSQC spectrum of compound **15** (500/125 MHz, CD_3CN)

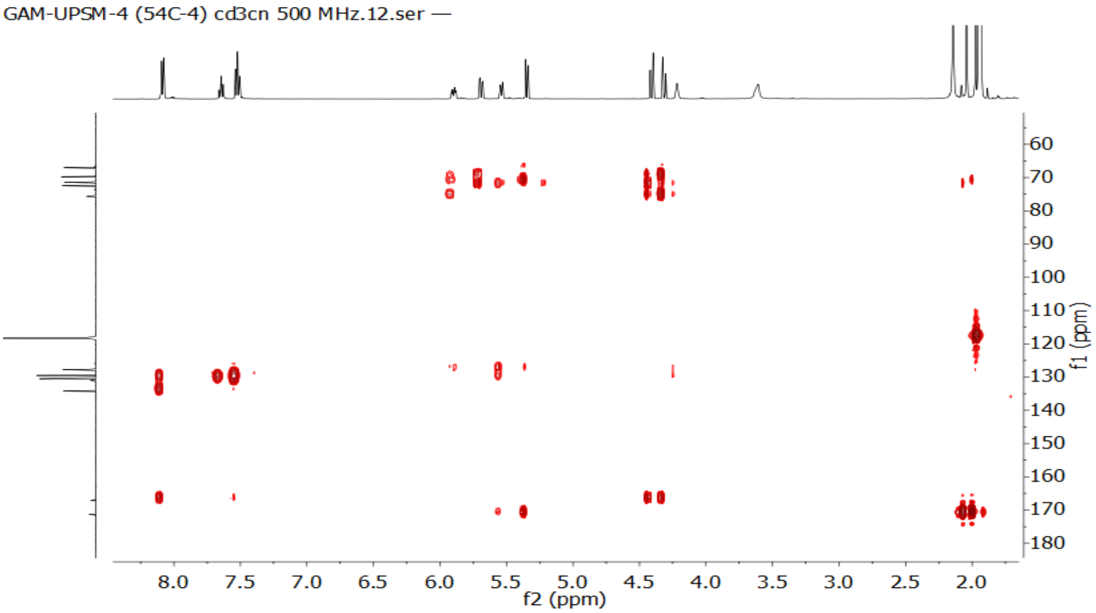

Figure S116. The HMBC spectrum of compound **15** (500/125 MHz, CD_3CN)

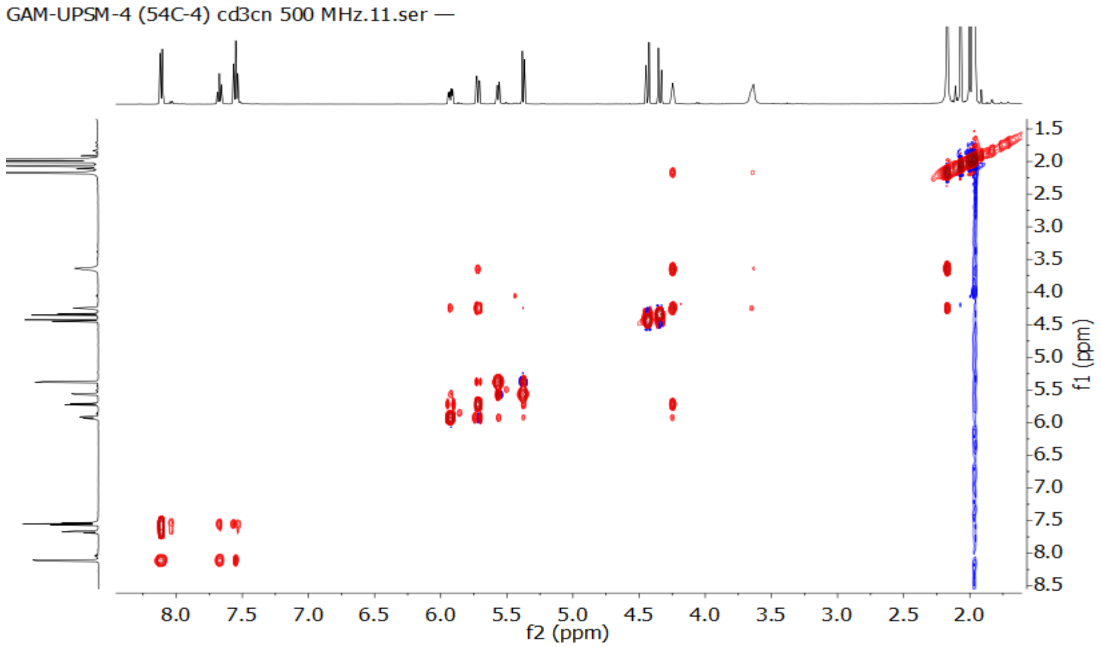

Figure S117. The TOCSY spectrum of compound **15** (500 MHz, CD_3CN)

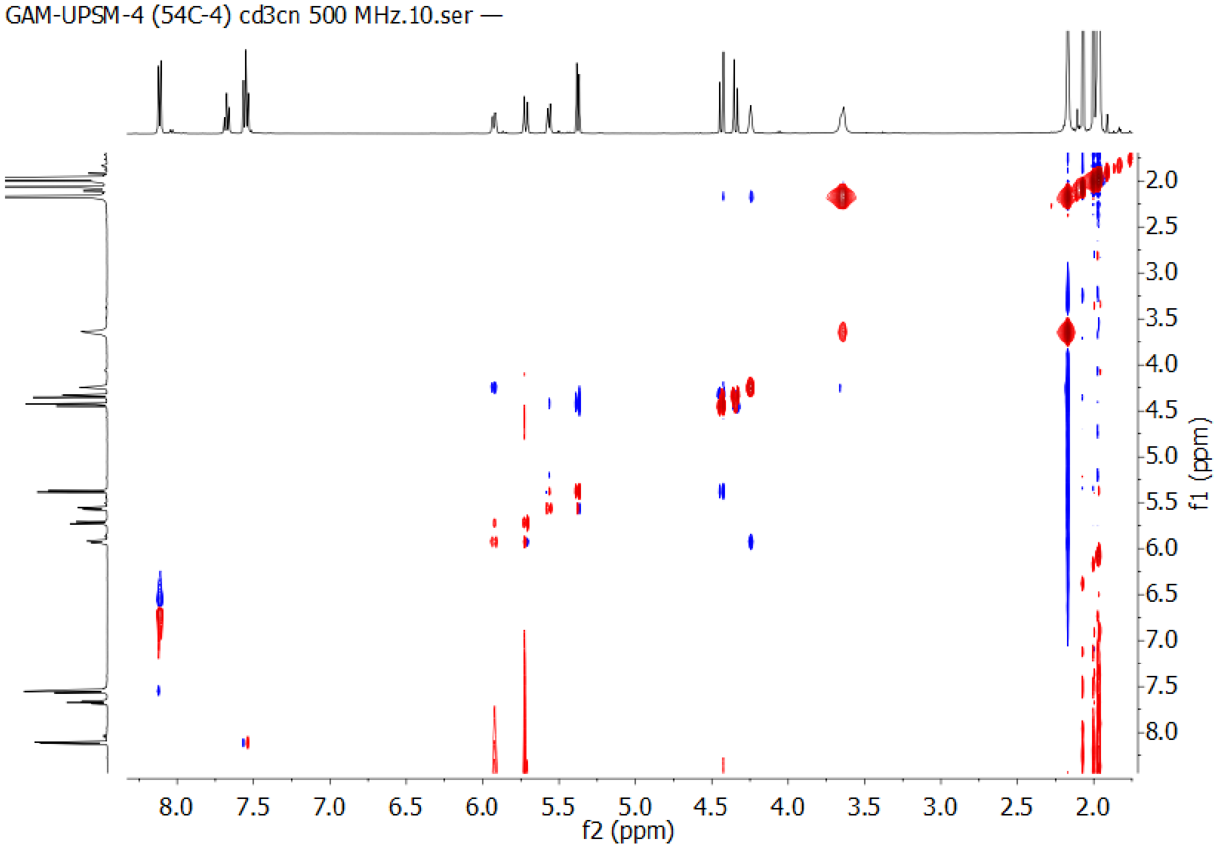

Figure S118. The NOESY spectrum compound **15** (500 MHz, CD_3CN)

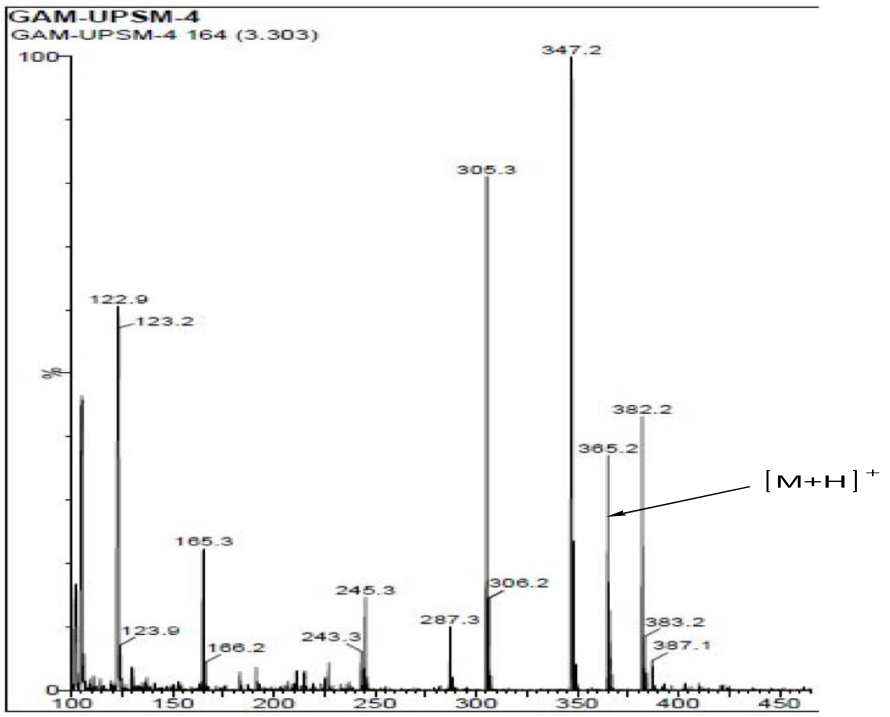

Figure S119. The LC-MS (ESI) of compound **15**

17. Spectroscopic data for compound 16

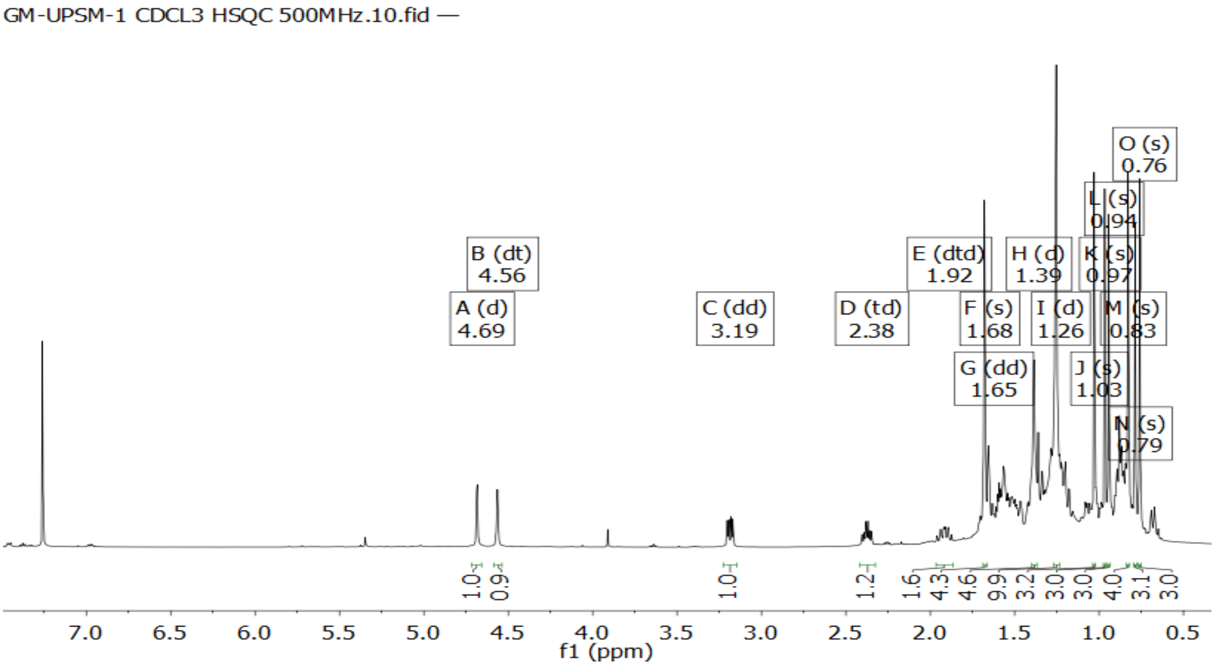

Figure S120. The ^{1}H NMR spectrum of compound **16** (500 MHz, $CDCl_3$)

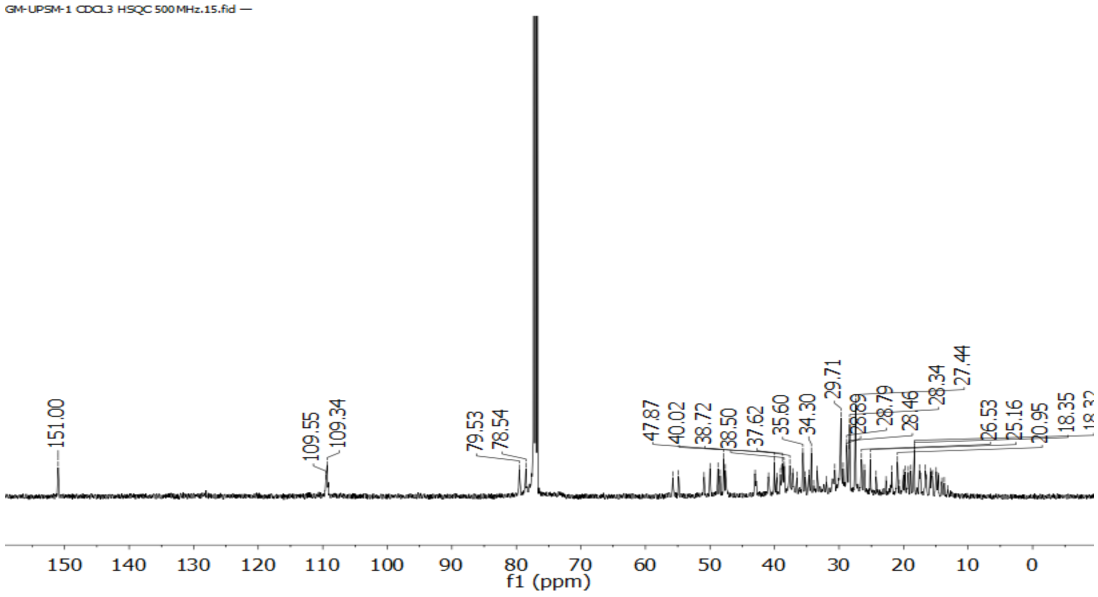

Figure S121. The ^{13}C NMR spectrum of compound **16** (125 MHz, $CDCl_3$)

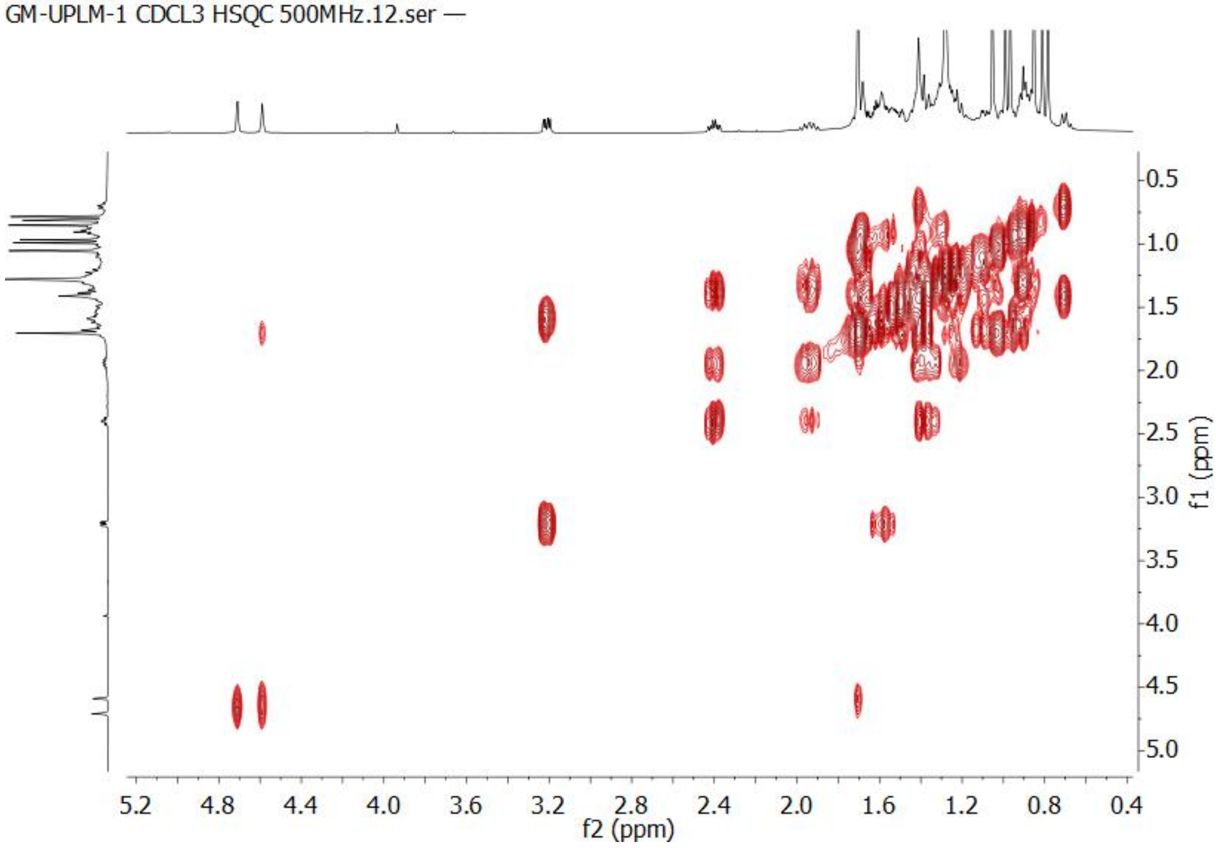

Figure S122. The COSY spectrum of compound **16** (500 MHz, $CDCl_3$)

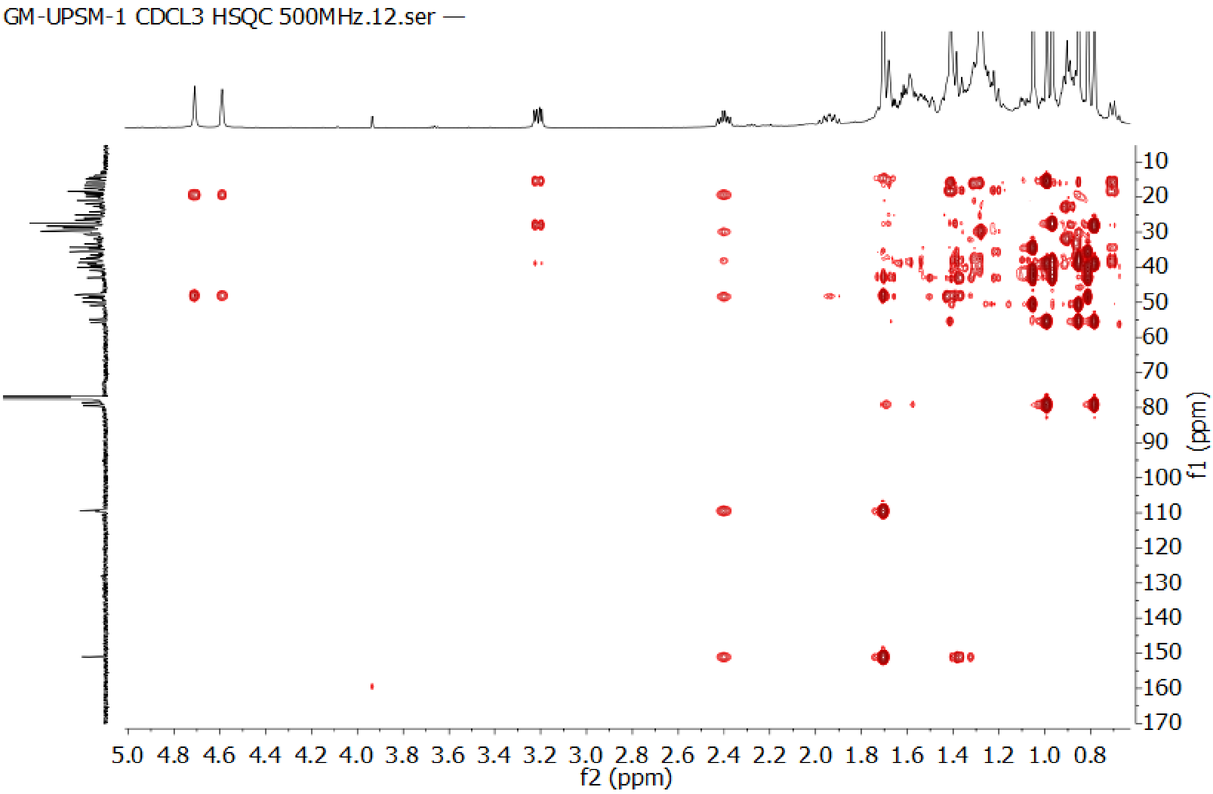

Figure S123. The HMBC spectrum of compound **16** (500/125 MHz, $CDCl_3$)

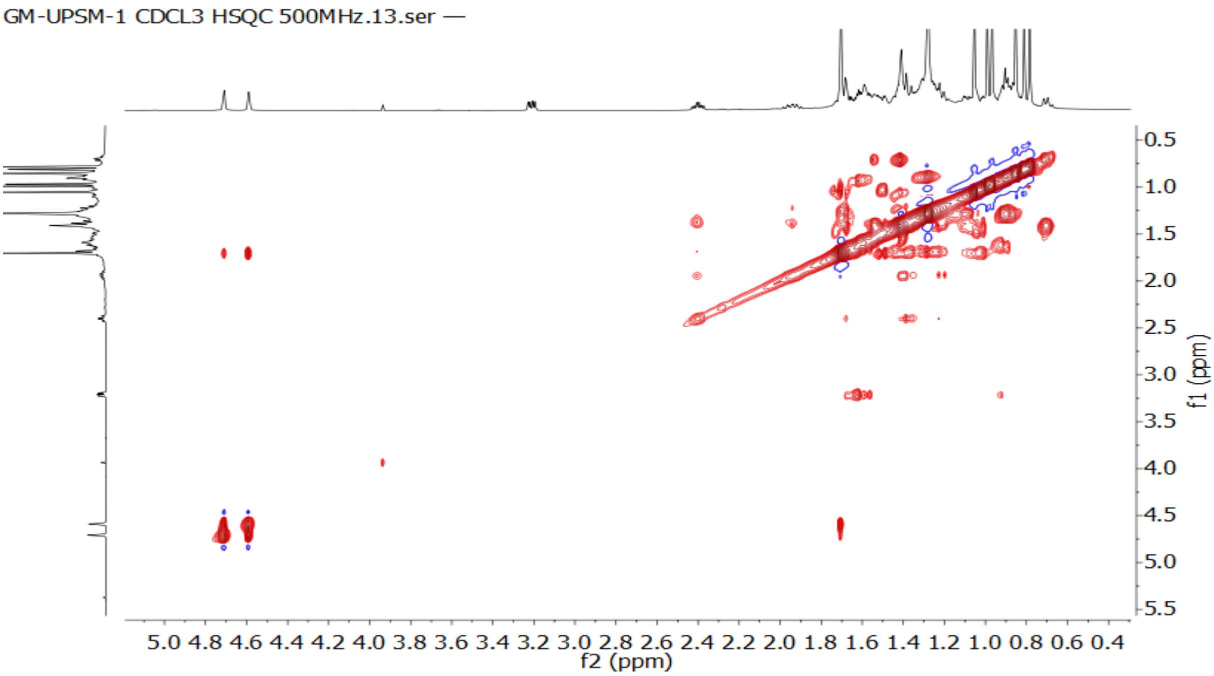

Figure S124. The TOCSY spectrum of compound **16** (500 MHz, $CDCl_3$)

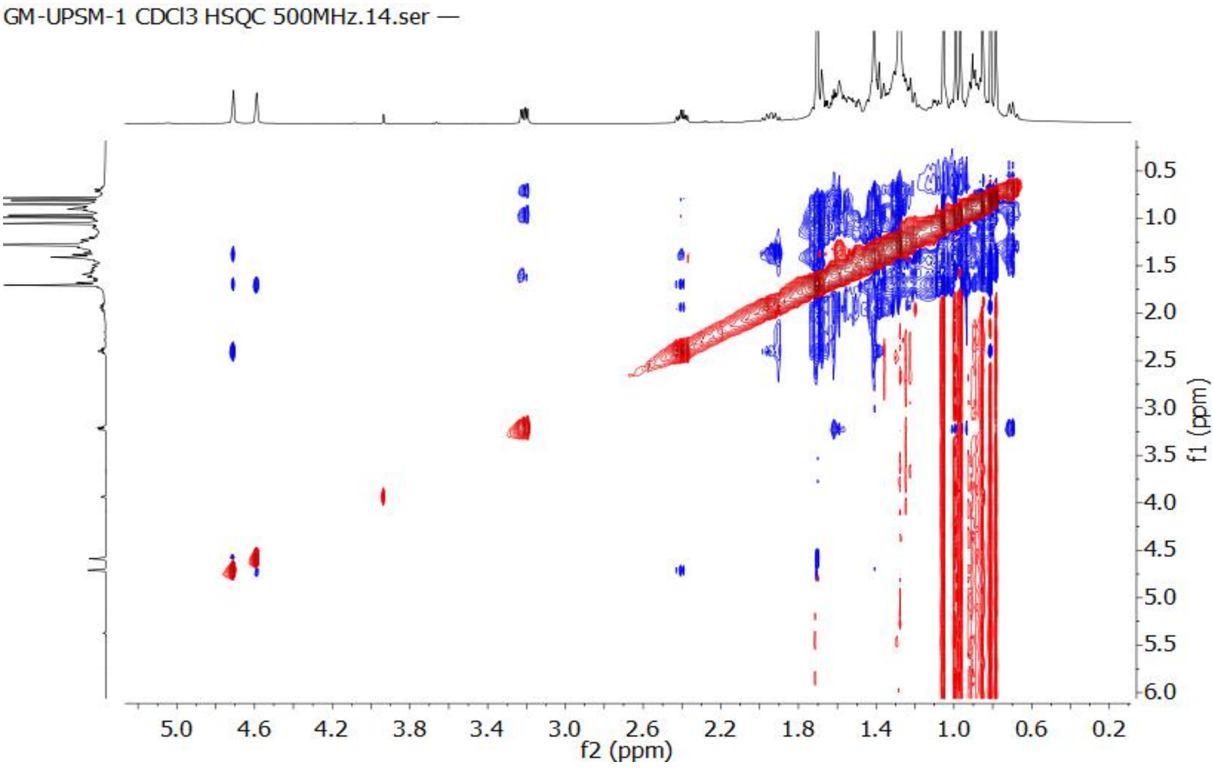

Figure S125: The NOESY spectrum of compound **16** (500 MHz, $CDCl_3$)

18. Spectroscopic data for compound 17

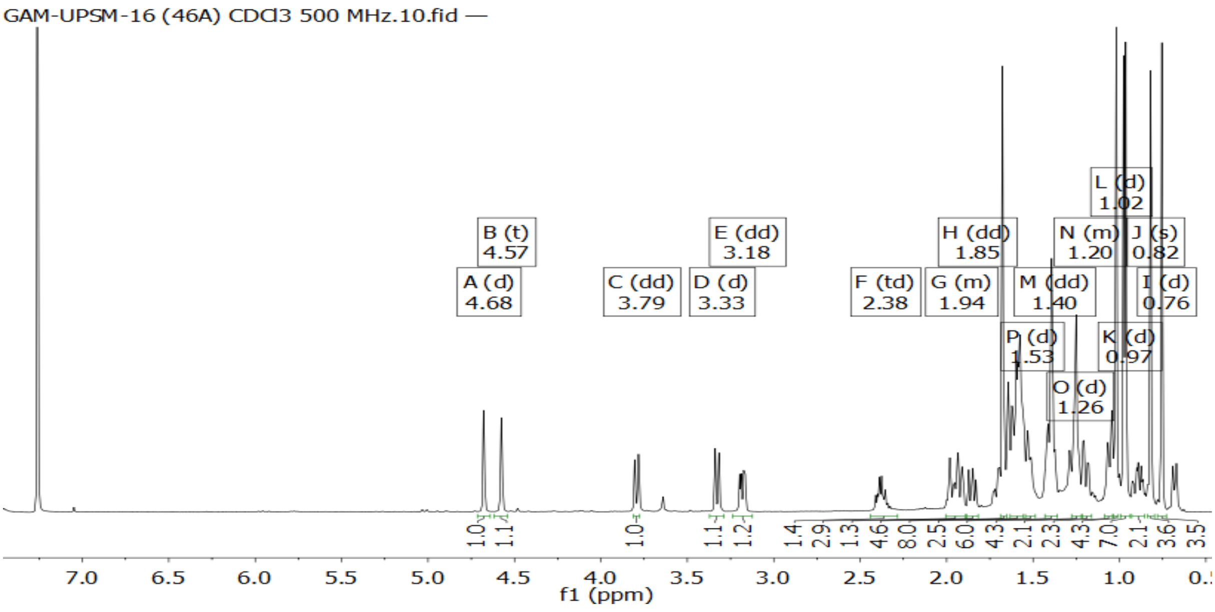

Figure S126. The ^{1}H NMR spectrum of compound **17** (500 MHz, $CDCl_3$)

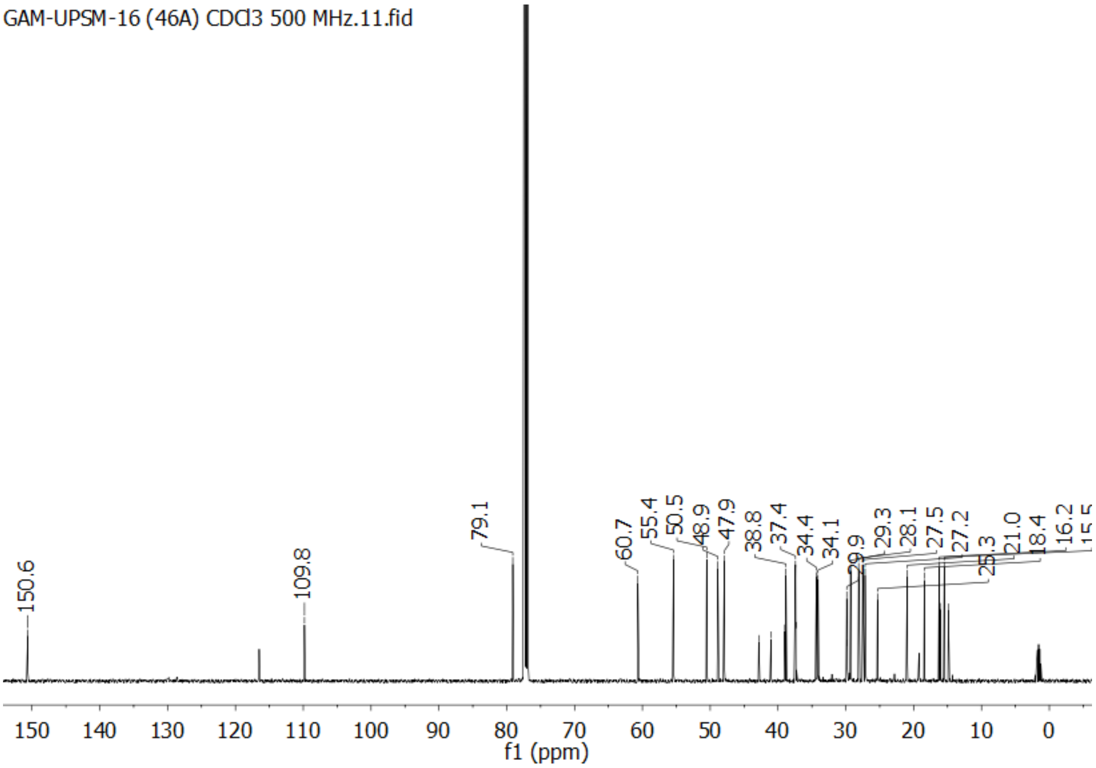

Figure S127. The ^{13}C NMR spectrum of compound **17** (125 MHz, $CDCl_3$)

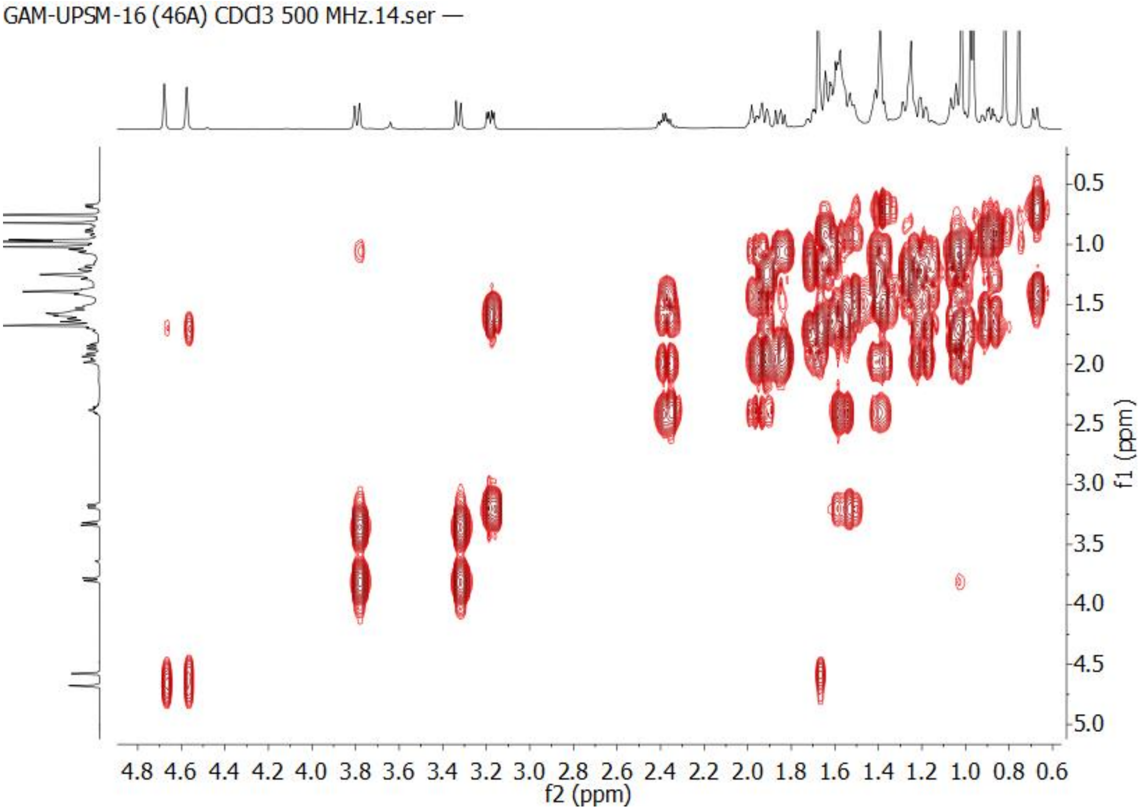

Figure S128. The COSY Spectrum of compound **17** (500 MHz, $CDCl_3$)

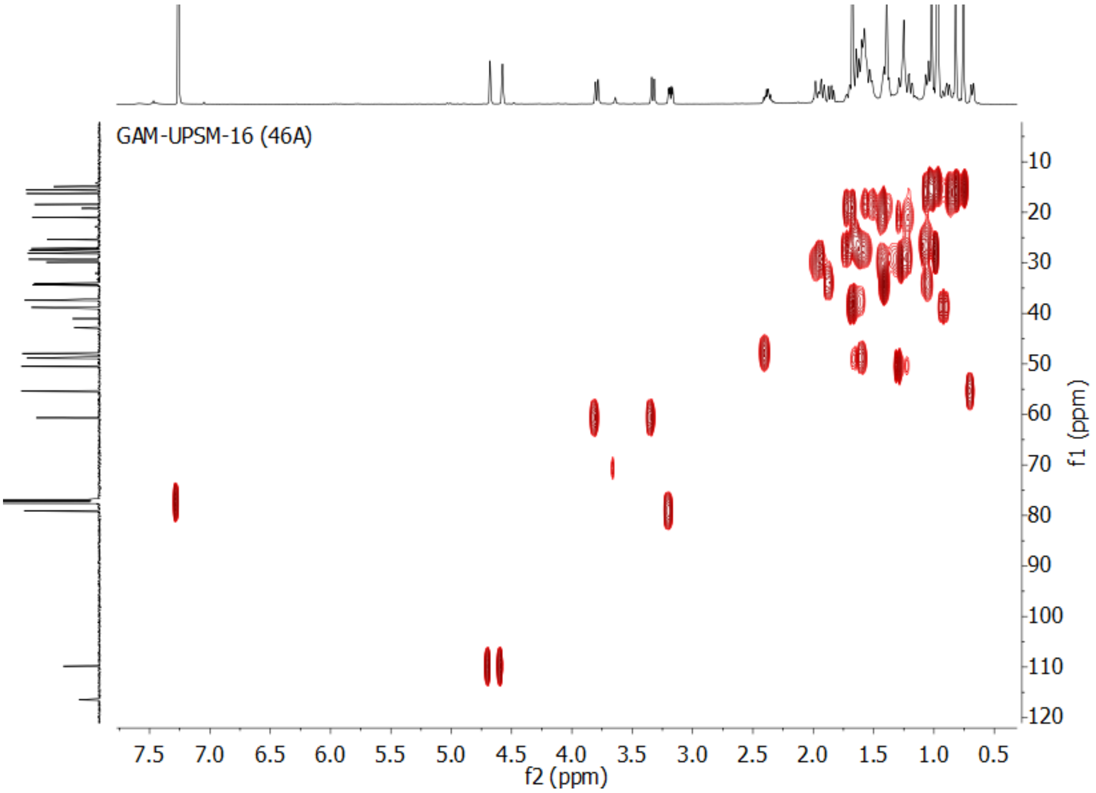

Figure S129. The HSQC spectrum of compound **17** (500/125 MHz, $CDCl_3$)

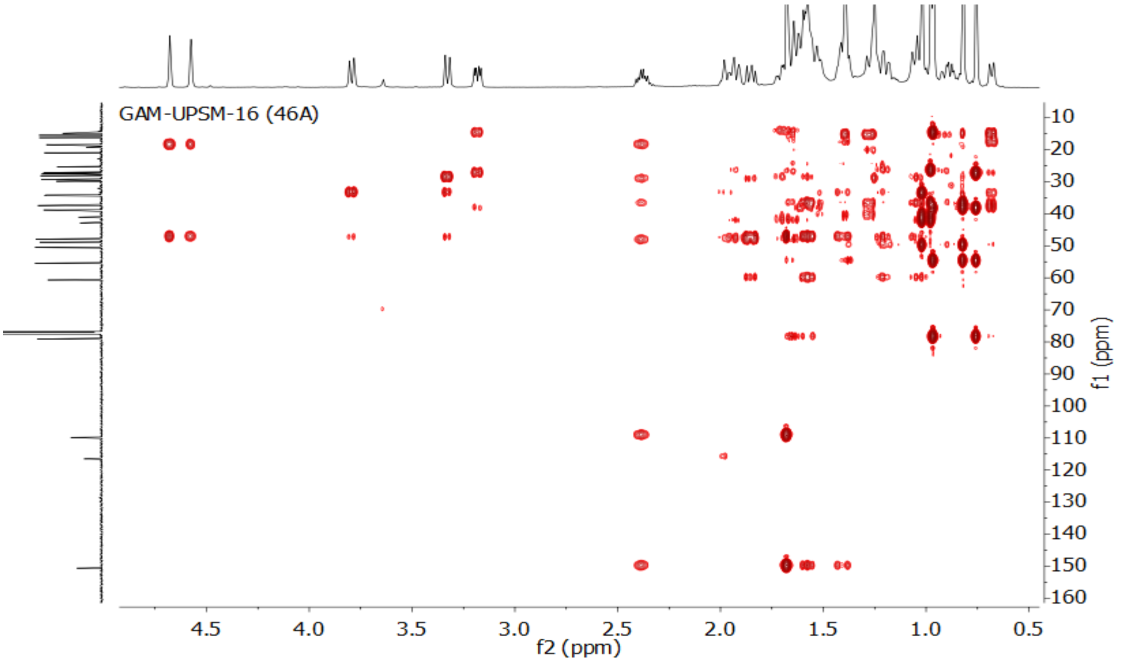

Figure S130. The HMBC spectrum of compound **17** (500/125 MHz, $CDCl_3$)

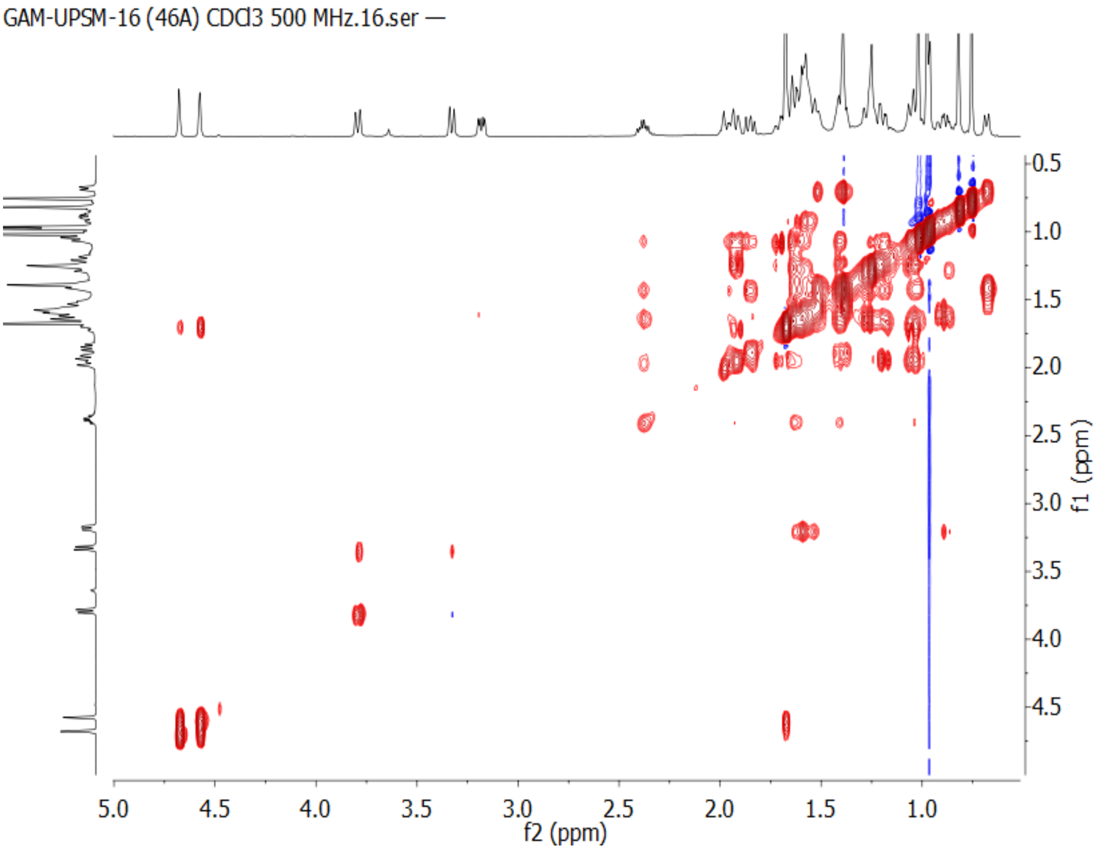

Figure S131. The TOCSY spectrum of compound **17** (500 MHz, $CDCl_3$)

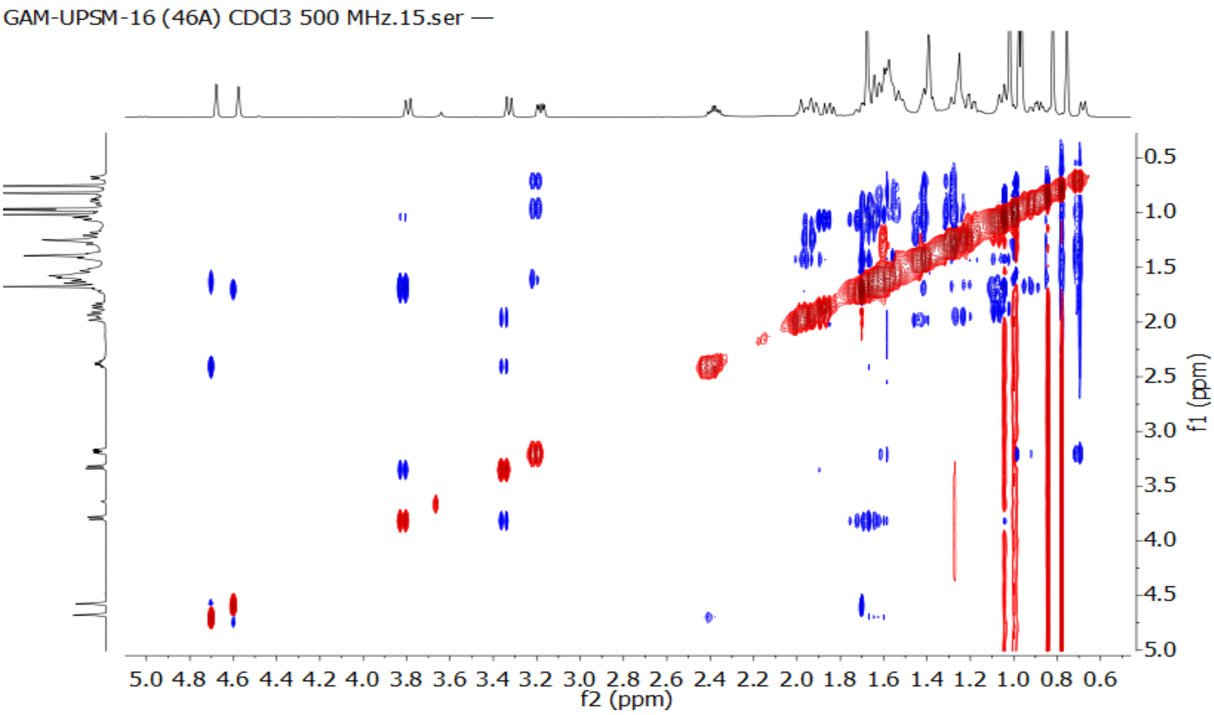

Figure S132. The NOESY spectrum of compound **17** (500 MHz, $CDCl_3$)

19. Spectroscopic data for compound 18 and 19

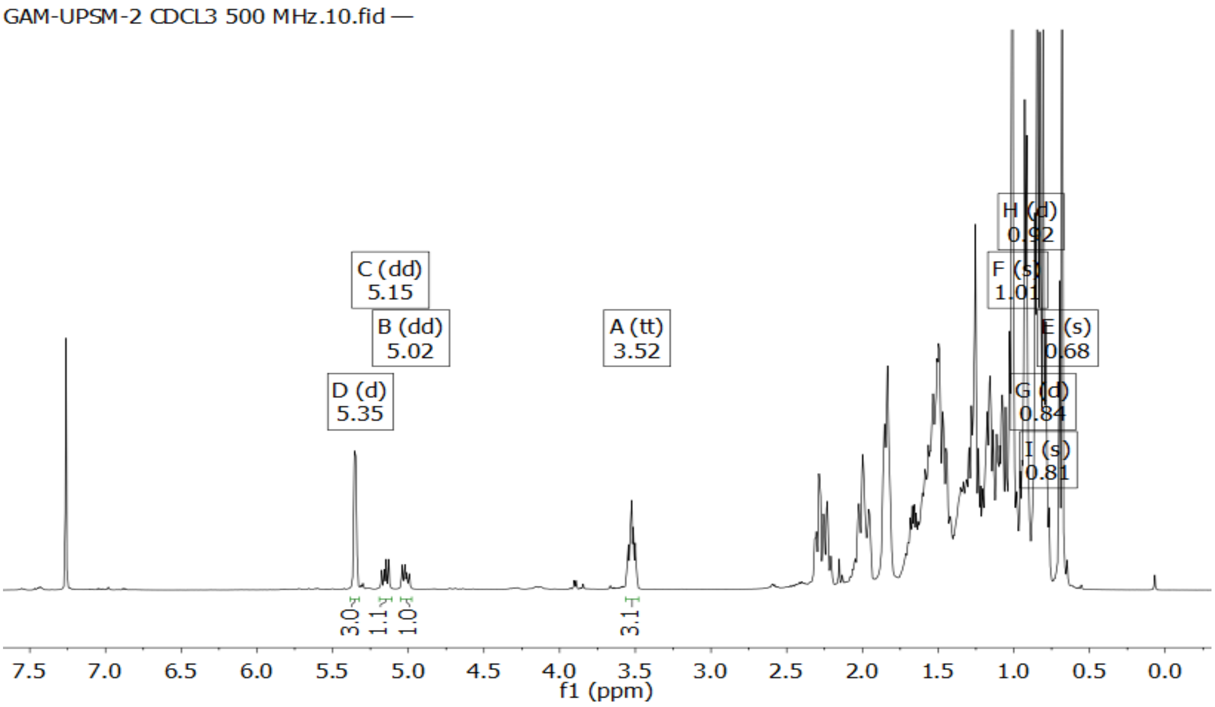

Figure S133. The ^{1}H NMR spectrum of a mixture of compound **18** and **19** (500 MHz, $CDCl_3$)

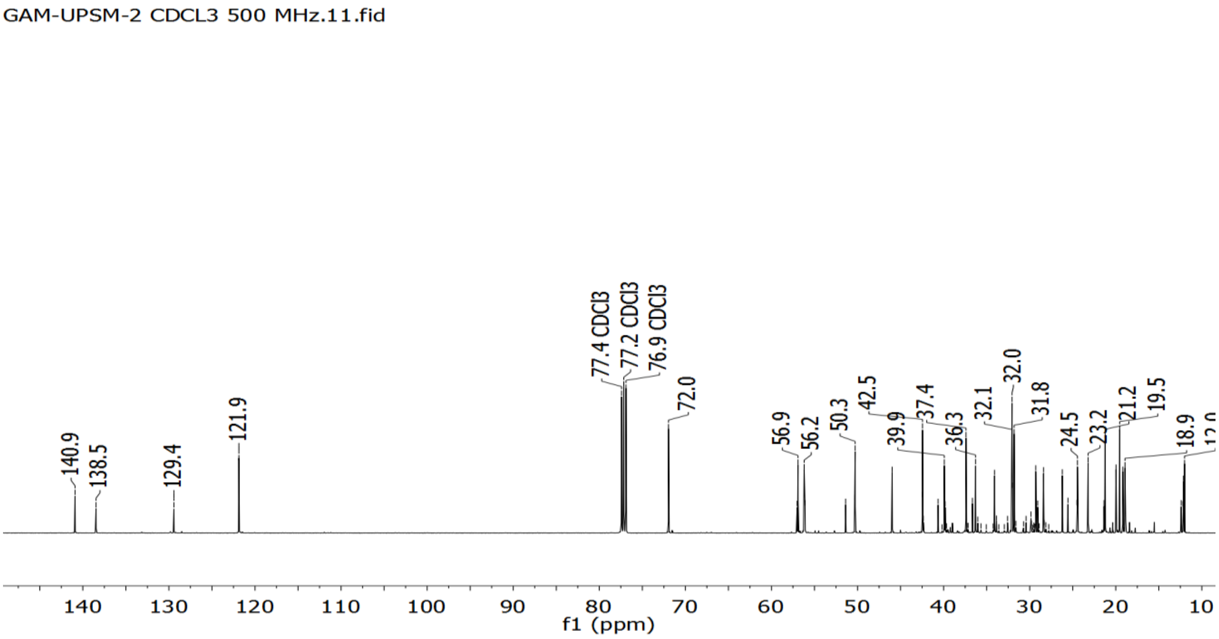

Figure S134. The ^{13}C NMR spectrum of a mixture of compound **18** and **19** (125 MHz, $CDCl_3$)

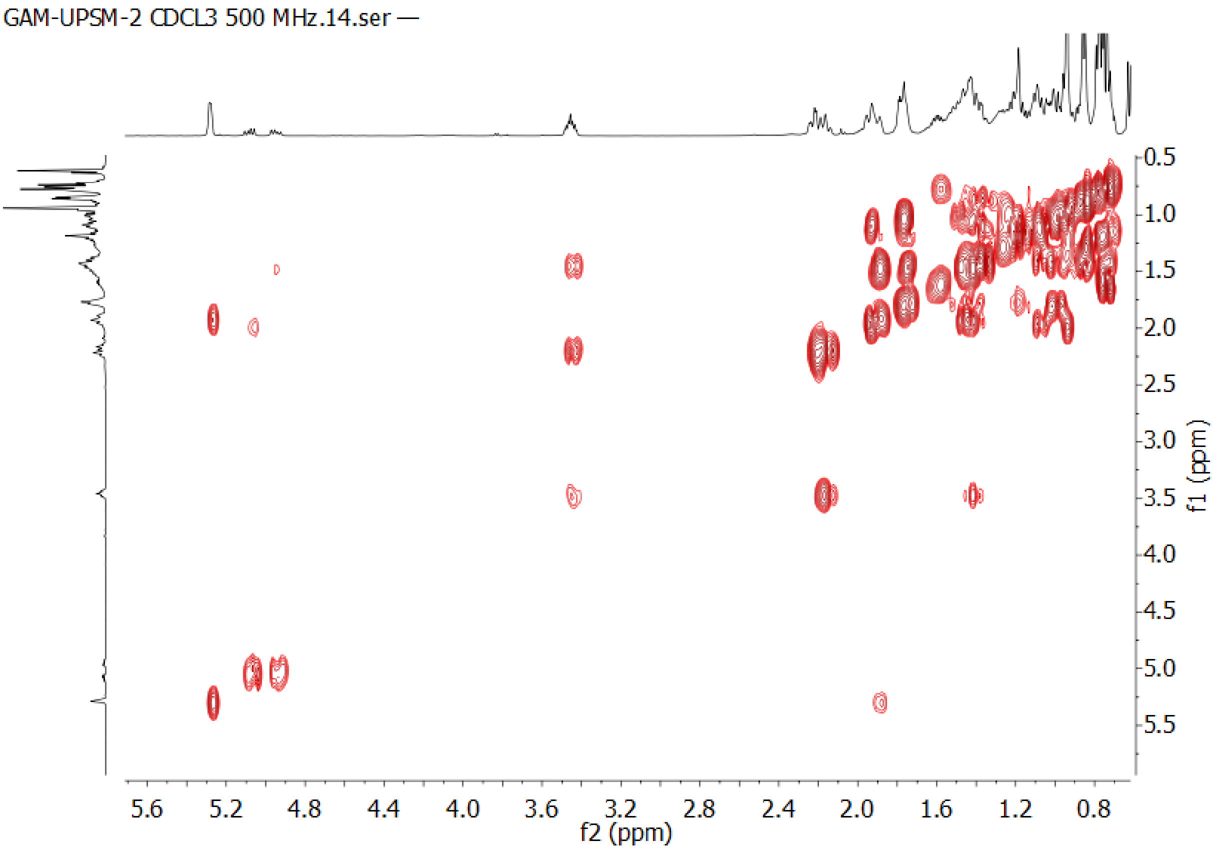

Figure S135. The COSY spectrum of a mixture of compound **18** and **19** (500 MHz, $CDCl_3$)

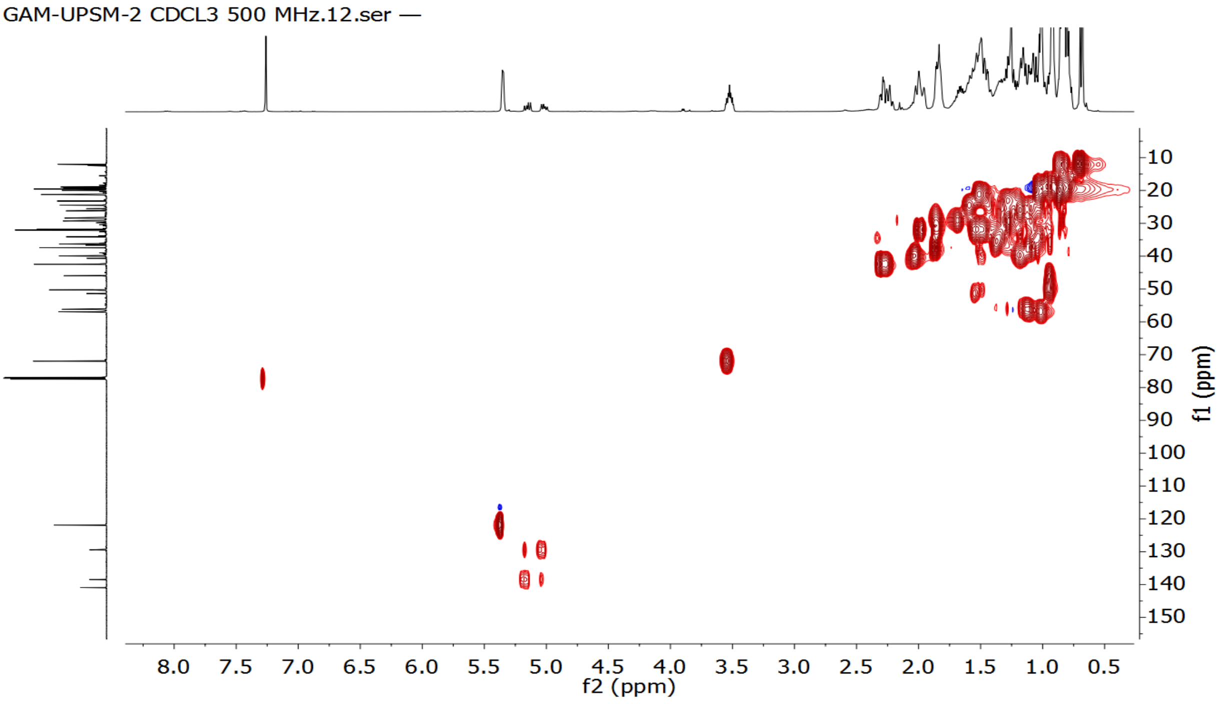

Figure S136. The HSQC spectrum of a mixture of compound **18** and **19** (500/125 MHz, $CDCl_3$)

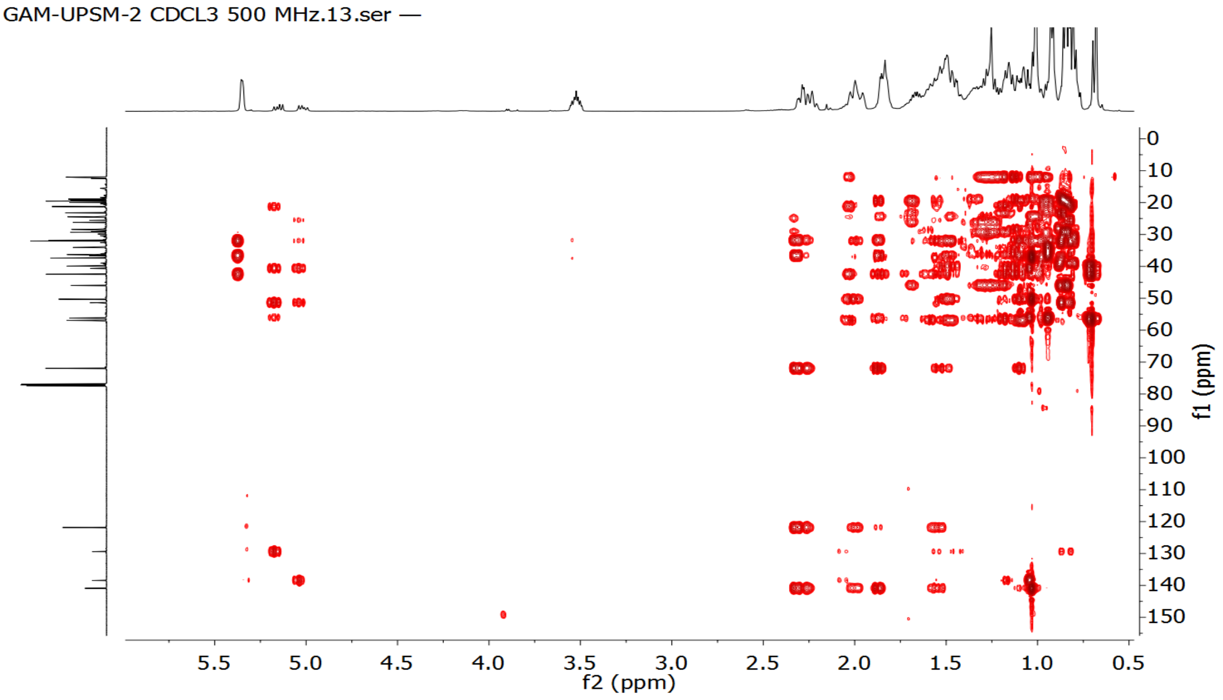

Figure S137. The HMBC spectrum of a mixture of compound **18** and **19** (500/125 MHz, $CDCl_3$)

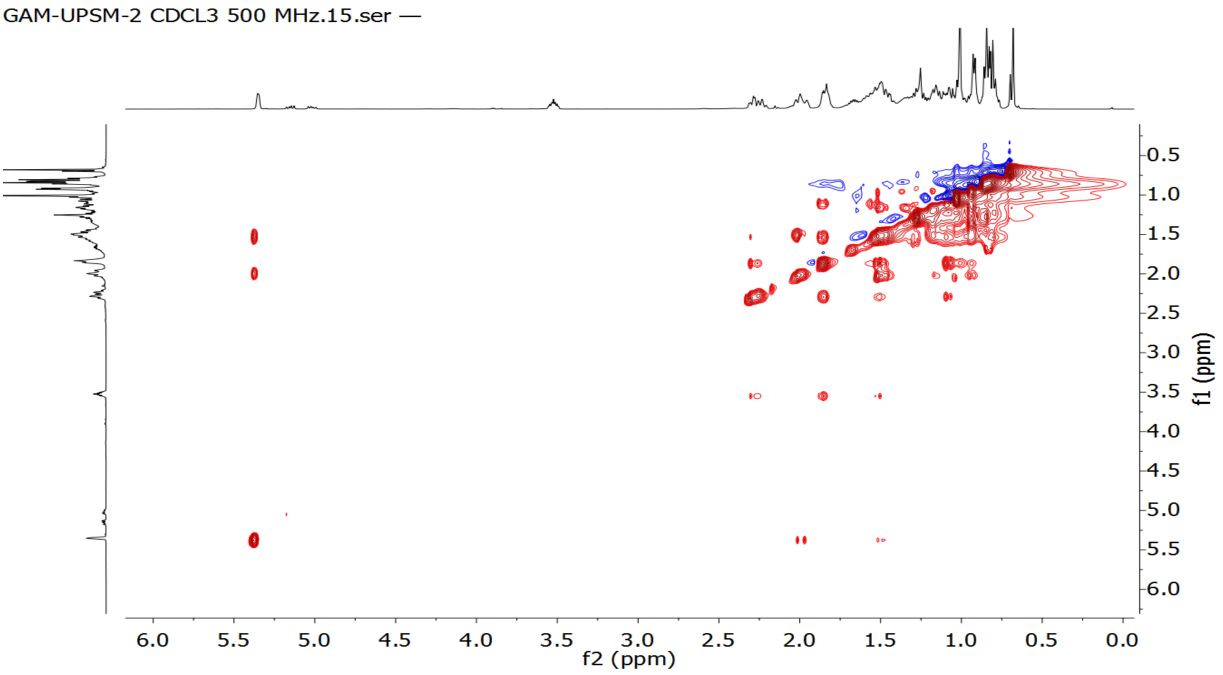

Figure S138. The TOCSY spectrum of a mixture of compound **18** and **19** (500 MHz, $CDCl_3$)

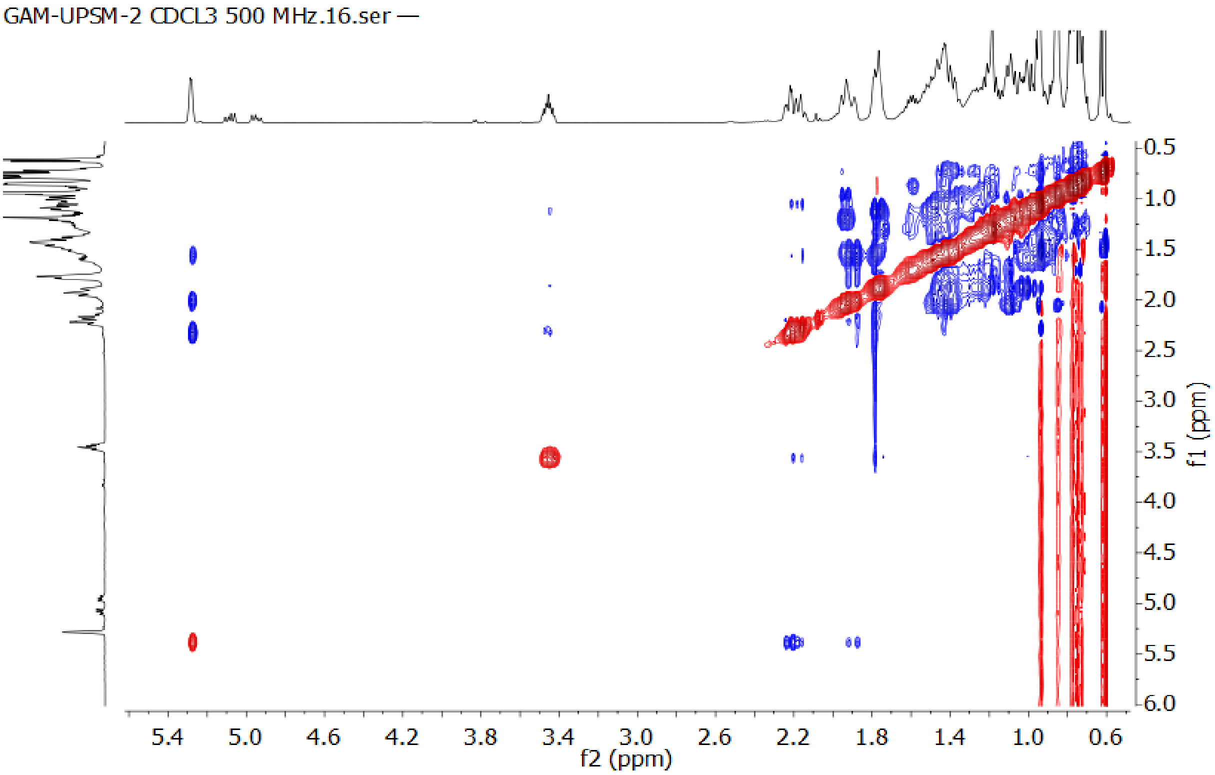

Figure S139. The NOESY spectrum of a mixture of compound **18** and **19** (500 MHz, $CDCl_3$)

20. Spectroscopic data for compound 20 and 21

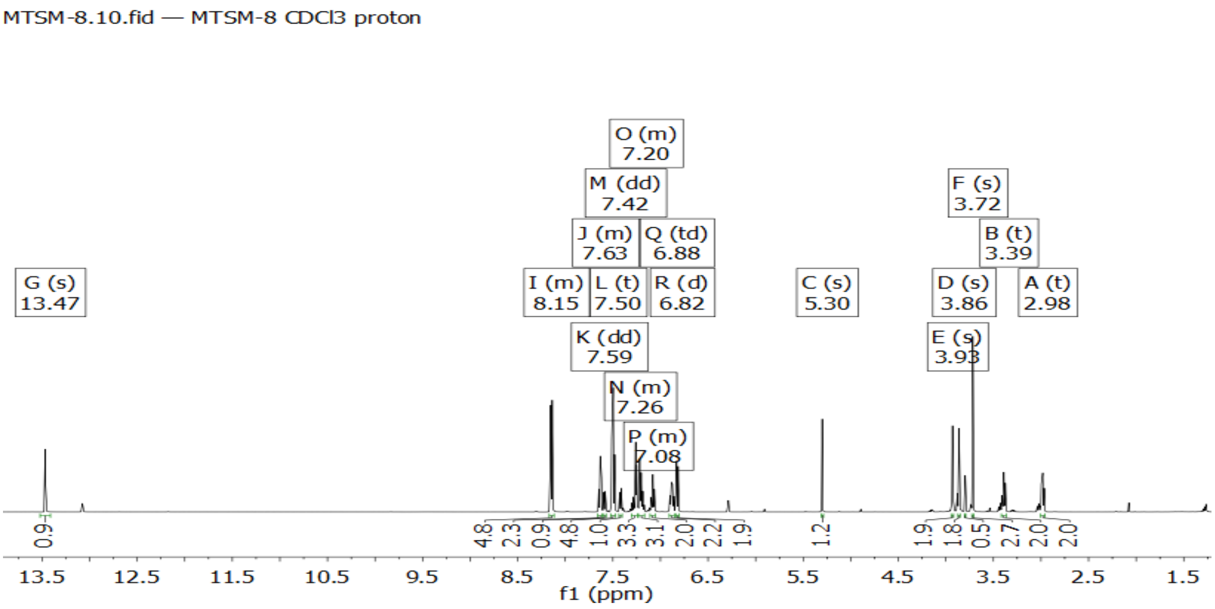

Figure S140. The ^{1}H NMR spectrum of a mixture of compound **20** and **21** (500 MHz, $CDCl_3$)

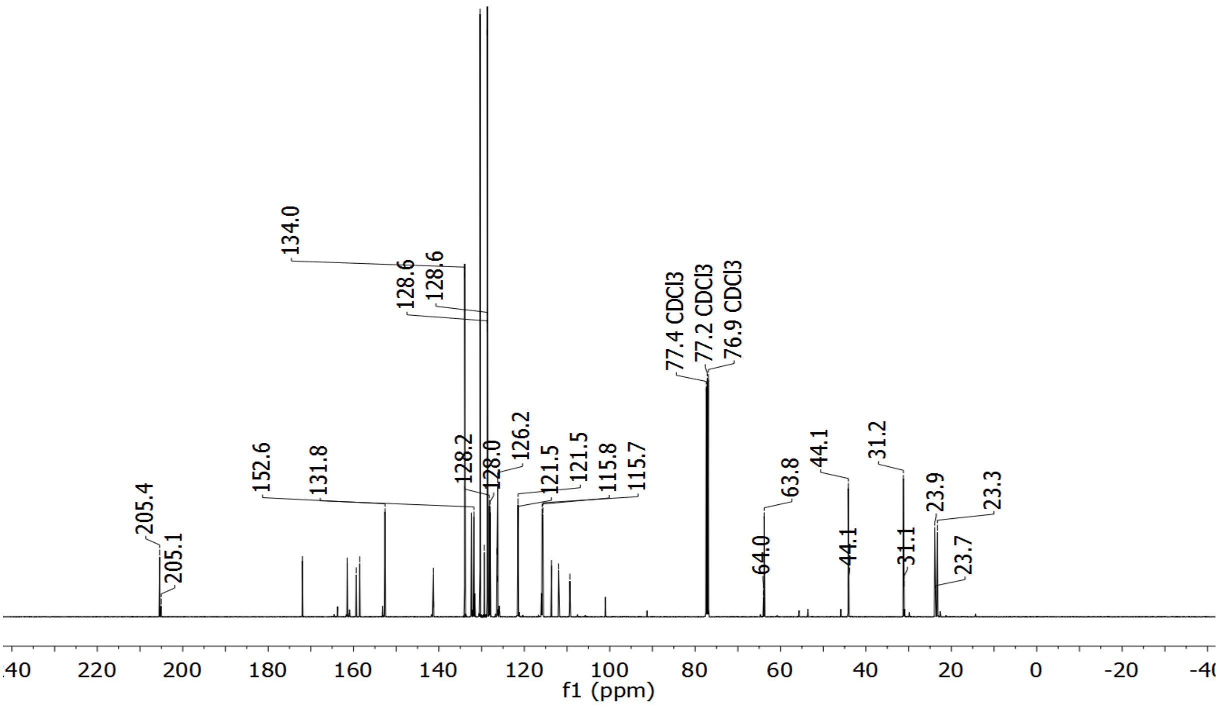

Figure S141. The ^{13}C NMR spectrum of a mixture of compound **20** and **21** (125 MHz, $CDCl_3$)

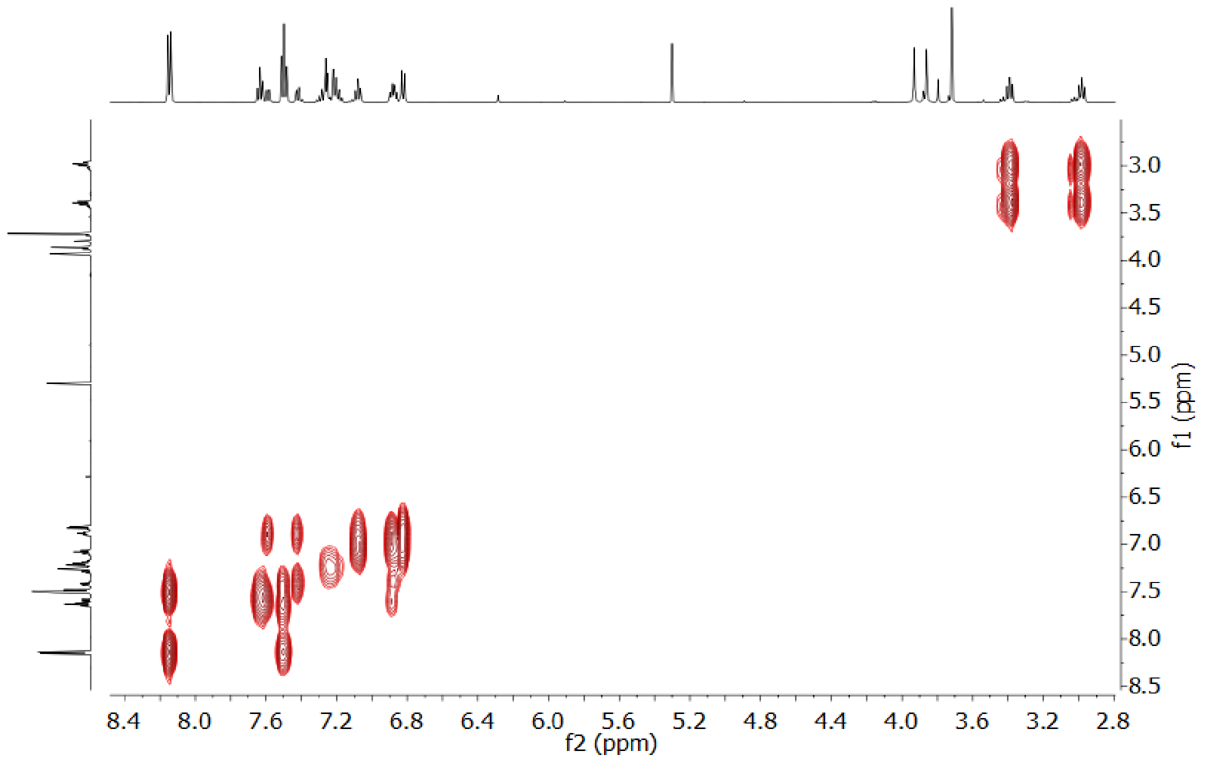

Figure S142. The COSY spectrum of a mixture of compound **20** and **21** (500 MHz, $CDCl_3$)

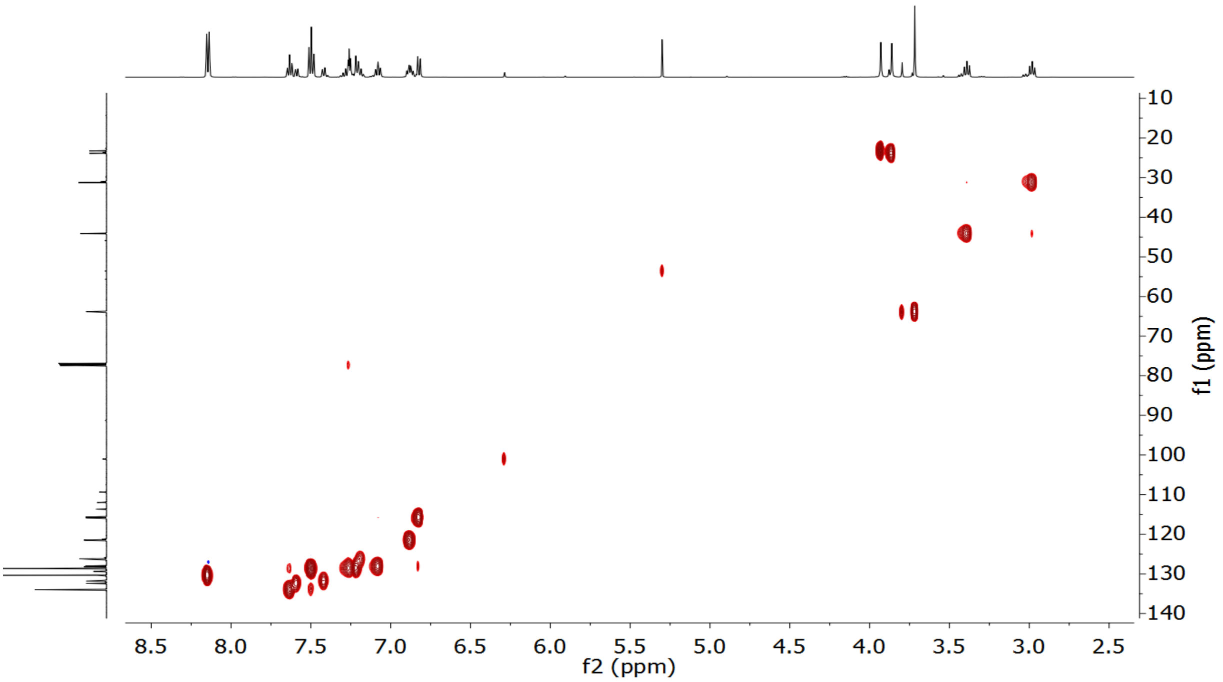

Figure S143 The HSQC spectrum of a mixture of compound **20** and **21** (500/125 MHz, $CDCl_3$)

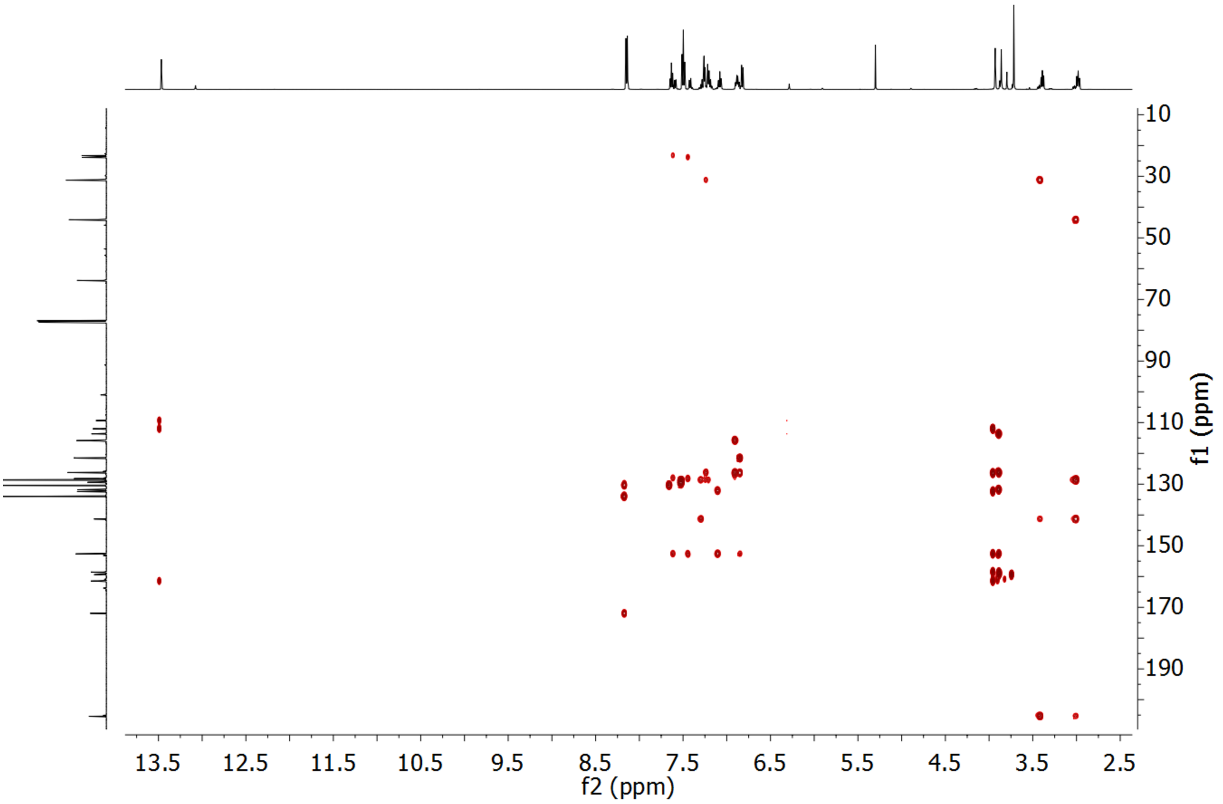

Figure S144. The HMBC spectrum of a mixture of compound **20** and **21** (500/125 MHz, $CDCl_3$)

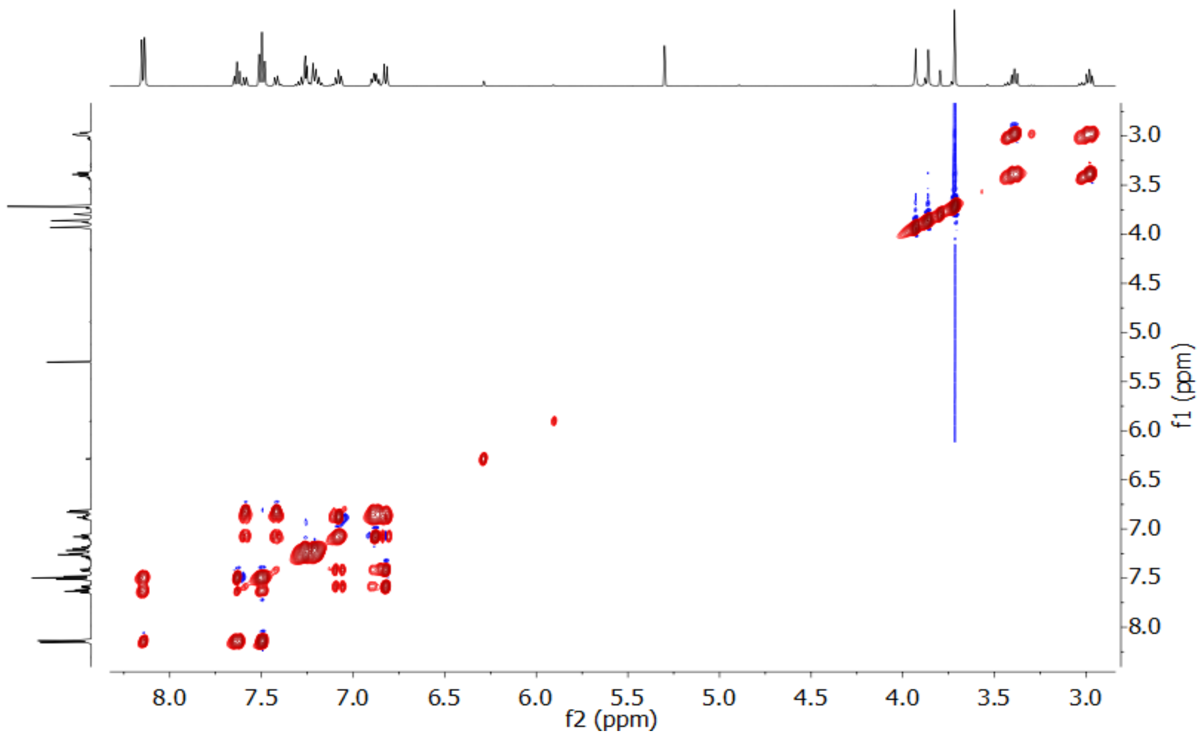

Figure S145. The TOCSY spectrum of a mixture of compound **20** and **21** (500 MHz, $CDCl_3$)

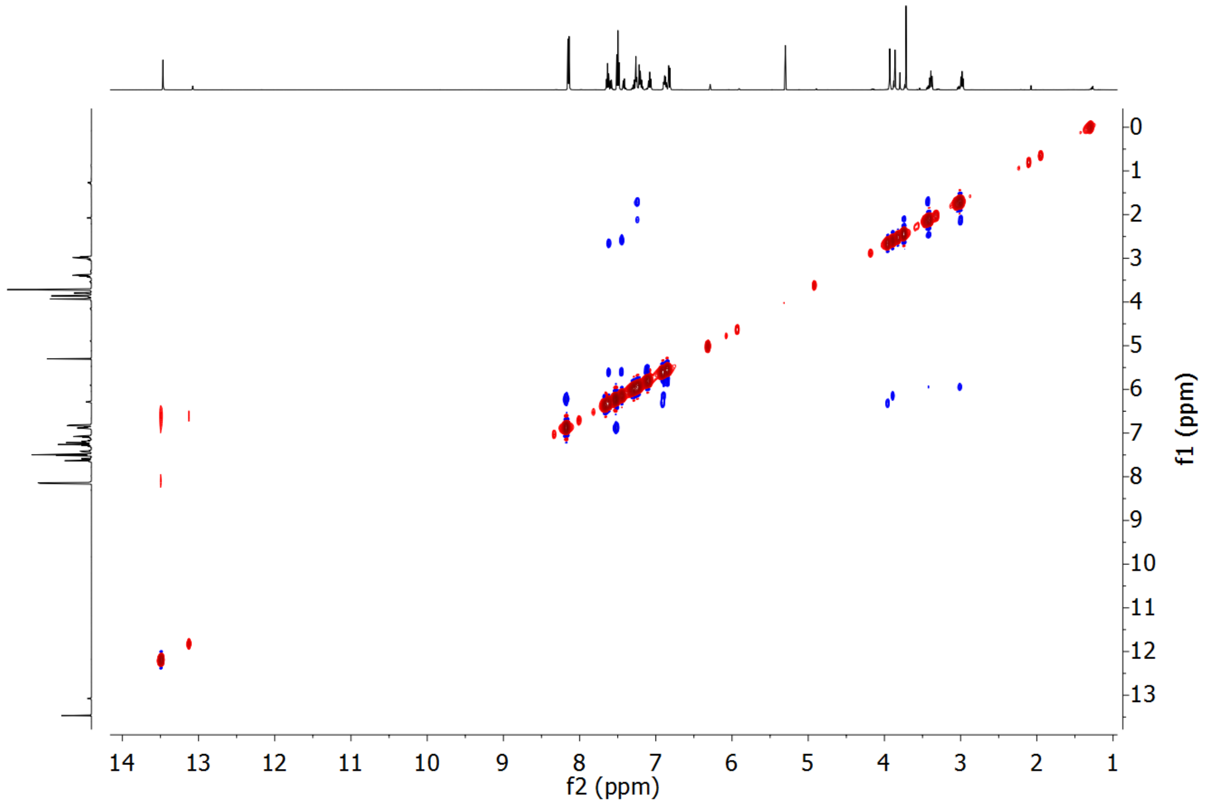

Figure S146. The NOESY spectrum of a mixture of compound **20** and **21** (500 MHz, $CDCl_3$)

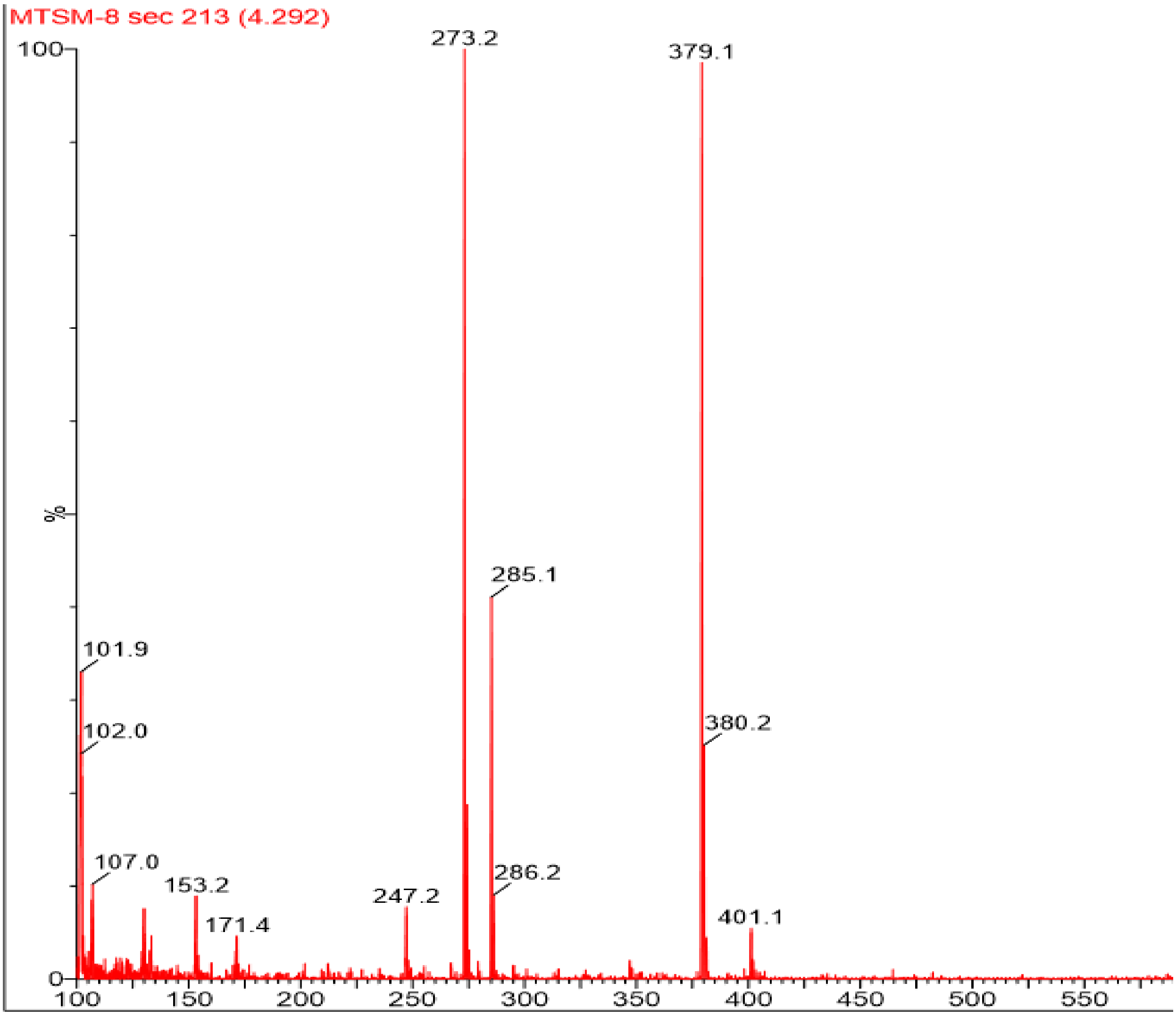

Figure S147. The LC-MS of a mixture of compound **20** and **21**

21. Other Spectroscopic and Physical Data of known Compounds (6-21)

*6-methoxyzeylenol (**6**)*: $[\alpha]_D^{24}$ + 38 (c 0.2, CH_3OH); UV (CH_2Cl_2) λ_{max} (log ε) 274 (2.85), 229 (4.02), 208 (4.25) nm; IR ν_{max} 3675, 2987, 2901, 2326, 1740, 1615, 1393, 1250, 1056, 892; LC-MS *m/z* 399.1 $[M+H]^+$

*Zeylenol (**7**)*: $[\alpha]_D^{24}$ + 64 (c 0.2, MeOH); UV (CH_2Cl_2) λ_{max} (log ε) 273 (3.26), 229 (4.25), 202 (4.12) nm; IR ν_{max} 3449, 1702, 1599, 1448, 1451, 1314, 1276, 1175, 1117, 952; LC-MS *m/z* 385.1 $[M+H]^+$

*Cleistenediol C (**8**)*: $[\alpha]_D^{24}$ +30 (c 0.2, MeOH); UV (MeOH) λ_{max} (log ε) 273 (3.43), 228 (4.27), 207 (4.24) nm; IR ν_{max} 3442, 2936, 1717, 1602, 1584, 1451, 1372, 1316, 1225, 1178, 1118, 10743, 1024, 961, 902, 749, 711 cm^{-1}; HRESIMS *m/z* observed 365.1291 $[M+H]^+$ calcd, 365.1236:, LC-MS *m/z* 365.1 $[M+H]^+$

*Cleistenediol F (**9**)*: $[\alpha]_D^{24}$ + 148 (c 0.2, MeOH); UV (MeOH) λ_{max} (log ε) 274 (3.44), 229 (4.39), 209 (4.35), nm; IR ν_{max} 3458, 3064, 2971, 2930, 2253, 1723, 1601, 1584, 1492, 1451, 1373, 1316, 1265, 1240, 1178, 1110, 1096, 1069, 1052, 967, 906, 731, 711; cm^{-1}; HRESIMS *m/z* observed 469.1493 $[M+H]^+$ calcd, 469.1498; LC-MS *m/z* 469.1 $[M+H]^+$

*Cherrevenol I (**10**)*: $[\alpha]_D^{24}$ +60 (c 0.3, MeOH); UV (CH_2Cl_2) λ_{max} (log ε) 274 (2.08), 232 (3.17) nm; IR ν_{max} 3522, 3456, 1692, 1601, 1584, 1493, 1420, 1370, 1279, 1255, 1119, 1093, 1082, 1029, 1001, 950, 863, 789, 703; LC-MS *m/z* 403.1 $[M+H]^+$, 405.1 $[M+2]^+$

*3-Methoxybenzylbenzoate (**11**)*: UV (CH_2Cl_2) λ_{max} (log ε) 272 (2.39), 223 (2.76) nm; IR ν_{max} 2524, 2444, 2158, 1974, 1713, 1360, 1272, 1221, 734 cm^{-1}; LC-MS *m/z* 241.2 $[M+H]^+$

*5-Hydroxy-7,4'-dimethoxyflavone (**12**)*: UV (MeOH) λ_{max} (log ε) 318 (1.8), 258 (2.16), 231 (2.12) nm; IR ν_{max} 3430, 2252, 2126, 1662, 1422, 1051, 1023, 1003, 822, 759 cm^{-1}; LC-MS *m/z* 299.2 $[M+H]^+$

*3,4-Dimethoxybenzyl alcohol (**13**)*: UV (CD_2Cl_2) λ_{max} (log ε) 278 (3.24), 232 (3.22), 203 (3.53) nm; IR ν_{max} 3310, 2944, 2832, 1449, 1418, 1113, 1022 cm^{-1}; LC-MS *m/z* 151.2 $[M-H_2O]^+$

*Zeyenyl-2,6-diacetate (**14**)*: $[\alpha]_D^{24}$ + 103 (c 0.3, CH_2Cl_2); UV (CH_2Cl_2) λ_{max} (log ε) 274 (2.96), 230 (3.96), 201 (3.99) nm; IR ν_{max} 3314, 2944, 2832, 2054, 1977, 1903, 1715, 1449, 1337, 1119, 1022; LC-MS *m/z* 469.1 $[M+H]^+$

*Benzoic acid 2,3-diacetoxy-1,6-dihydroxy-cyclohex-4-enylmethyl ester (**15**)*: $[\alpha]_D^{24}$ - 28 (c, 1.3, CH_2Cl_2); UV (CH_2Cl_2) λ_{max} (log ε) 273 (2.71), 229 (3.74), 201 (3.77) nm; LC-MS *m/z* 365.2 $[M+H]^+$

*Lupeol (**16**):* $[\alpha]_D^{24}$ +136 (c 0.2, CH_2Cl_2); UV (CH_2Cl_2) λ_{max} (log ε) 202 (3.56) nm; IR ν_{max} 3053, 2898, 2978, 2884, 1679, 2310, 1601, 1420, 1267, 890

*Betulin (**17**):* UV (CH_2Cl_2) λ_{max} (log ε) 330 (2.59) nm; IR ν_{max} 3502, 3412, 2988, 2970, 2309, 1760.

*Mixture of Stigmasterol (**18**) and β-sitosterol (**19**)*: IR ν_{max} 3412, 2933, 2957, 2866, 1667, 1463, 1377, 1367, 1332, 1051, 1022, 925.

*Mixture of Uvaretin (**20**) and Isouvaretin (**21**)*: $[\alpha]_D^{24}$ + 43 (c 0.3, CH_2Cl_2); UV (CH_2Cl_2) λ_{max} (log ε) 281 (3.54), 230 (4.13), 220 (2.95) nm; IR ν_{max} 3211, 1691, 1603, 1490, 1452, 1417, 1367, 1318, 1287, 1230, 1177, 754, 711; LC-MS *m/z* 377.2 $[M-H]^-$.

22. X-ray crystallography of compound 6

6-methoxyzeylenol (**6**): 0.32 x 0.28 x 0.16 mm^3, $C_{22}H_{22}O_7$, M = 398.39, orthorhombic, Temperature = 150(2) K, orthorhombic space group $P2_12_12_1$, a = 5.8321(6) Å, b = 14.2977(15) Å, c = 22.749(2) Å, α = 90°, β = 90°, γ = 90°, V = 1897.0(3) Å, Z = 4, ρ = 1.395 Mg/m^3, $\mu(MoK_\alpha)$ = 0.104 mm^{-1} , F(000) = 840, 17903 reflections (θ_{max} = 25.237°), Max. & min. transmission = 0.7457 and 0.6813, Refinement method = Full-matrix least-squares on F^2, Absolute structure parameter = 0.2(8), Independent reflections 3405 [R(int) = 0.0635], Completeness = 99.9 %. Final R indices (I > 2σ(I)): R_1 = 0.0477, wR_2 = 0.1084, R indices (all data): R_1 = 0.0601, wR_2 = 0.1160. GOF = 1.077 for 3405 data, 266 parameters and 0 restraints, largest diff. peak and hole 0.258 and -0.242 e.Å^{-3}. CCDC-2105244 contains the supplementary data for this structure. An ORTEP image (50% probability ellipsoids) of the molecule of 6-methoxyzeylenol (**6**) is shown below.

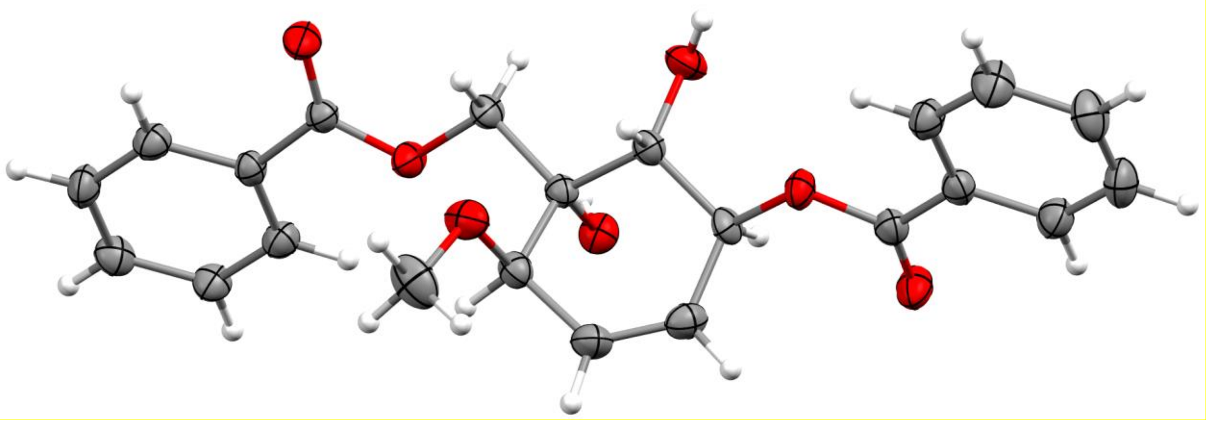

23. Antibacterial and cytotoxicity data

Table S1. Antibacterial activity of compounds **5**, **12**, **13** and **20&21** against Gram-positive *Bacillus subtilis* and of compound **20&21** against Gram-positive *Staphylococcus epidermidis*, and Gram-negative *Escherichia coli* and *Pectobacterium carotovorum,* showing the EC_{50}, EC_{90} and MIC values (in µM) determined for each compound from *Uvaria pandensis.* Compounds **2**, **4**, **11**, and **17** were not evaluated due to limited amount of available sample.

	B. subtilis				*S. epidermidis*	*E. coli*	*P. carotovorum*
**Compound** (µM)	**5**	**12**	**13**	**20&21**	**20&21**	**20&21**	**20&21**
**EC_{50}**	>4037.7	9.8	1154.1	8.7	7.9	1130.8	263.1
SD	N/A	52.6	762.8	0.8	2.3	164.9	6.5
SE range	N/A	38.5-99.1	636.1-1516.9	8.7-9.5	6.3-8.4	382.2-572.7	250.6-277.0
**EC_{90}**	>4037.7	147.2	1671.6	15.6	18.11	1958.6	664.3
SD	N/A	104.7	1181.1	3.4	1.5	171.2	1640.5
SE range	N/A	64.6-185.5	979.2-2343.1	12.1-15.8	11.6-18.5	1719.1-1916.6	646.3-672.7
**MIC**	>4037.7	>3188.9	>7042.2	118.7	47.55	2114.8	1441.0

Figure S148. Antibacterial activity dose-response curves of Compounds **5**, **12** and **13** against Gram-positive *Bacillus subtilis.* EC_{50}, EC_{90} and MIC values (in µM) determined for each compound can be found on Table 1.

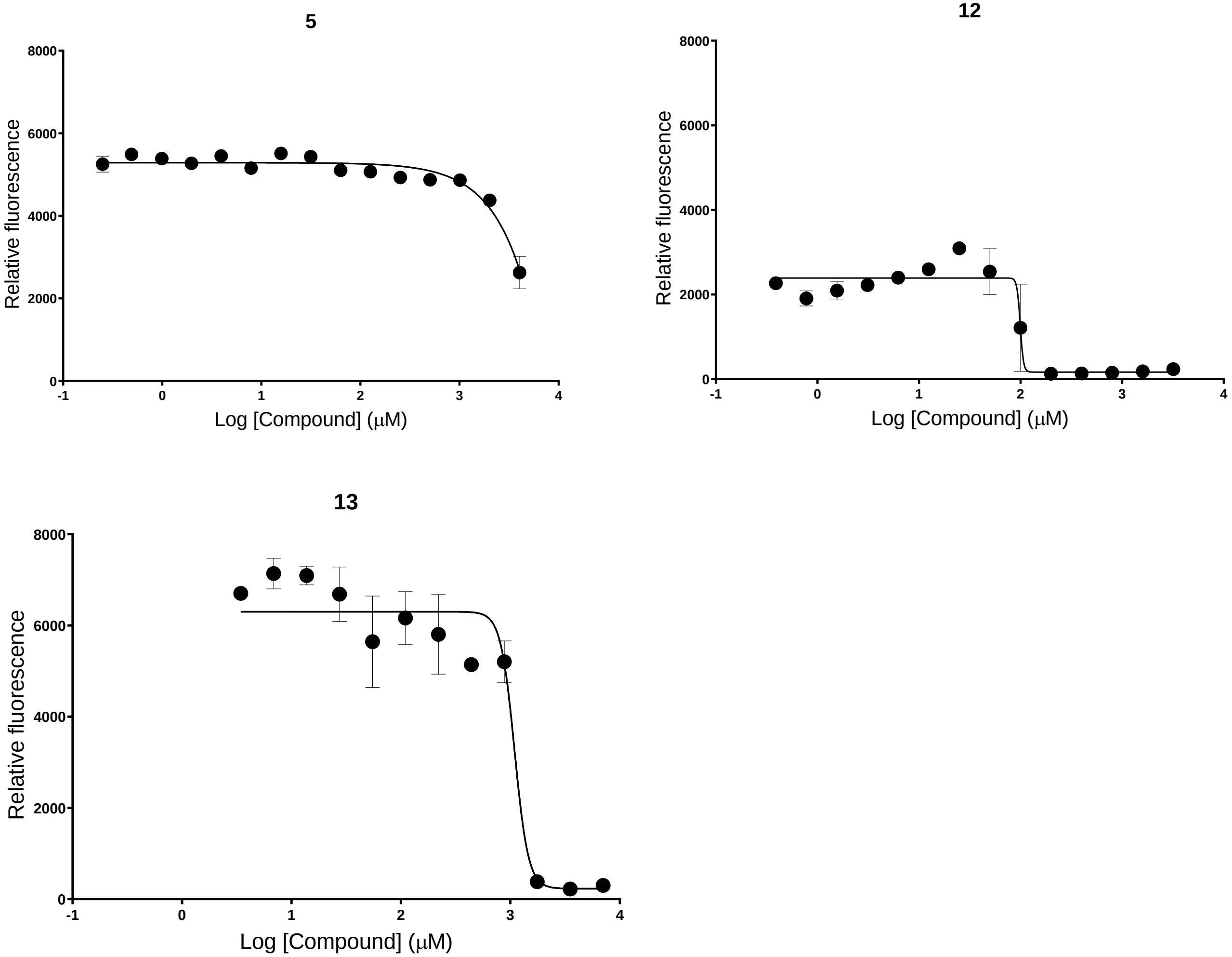

Figure S149. Antibacterial activity dose-response curves for compound **20&21** against Gram-positive *Bacillus subtilis and Staphylococcus epidermidis*, and *G*ram-negative *Escherichia coli* and *Pectobacterium carotovorum*. EC_{50}, EC_{90} and MIC values determined for this compound are summarized in Table 1.

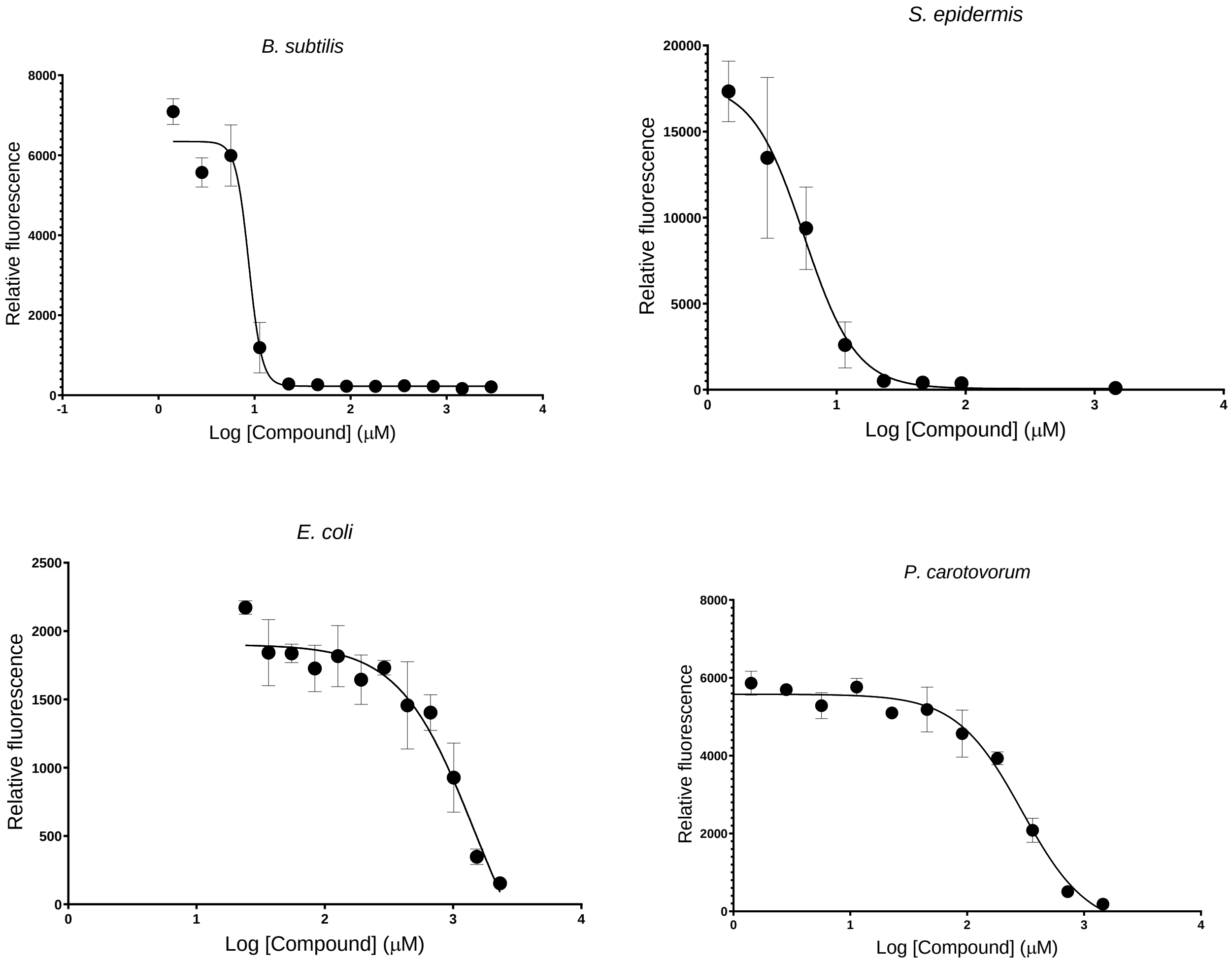

Figure S150. Cytotoxicity dose-response curves. Compound **5** showed EC_{50} = 365.0 µM (SD = 38.6; SE range of 350.8 to 395.4), compound **12** presented an EC_{50} = 117.1 µM (SD = 29.0; SE range of 82.4 to 115.9), compound **13** presented an EC_{50} >563.4 µM, and the compound **20** and **21** a EC_{50} = 138 µM (SD = 28.0; SE range of 95.0 to 127.1).

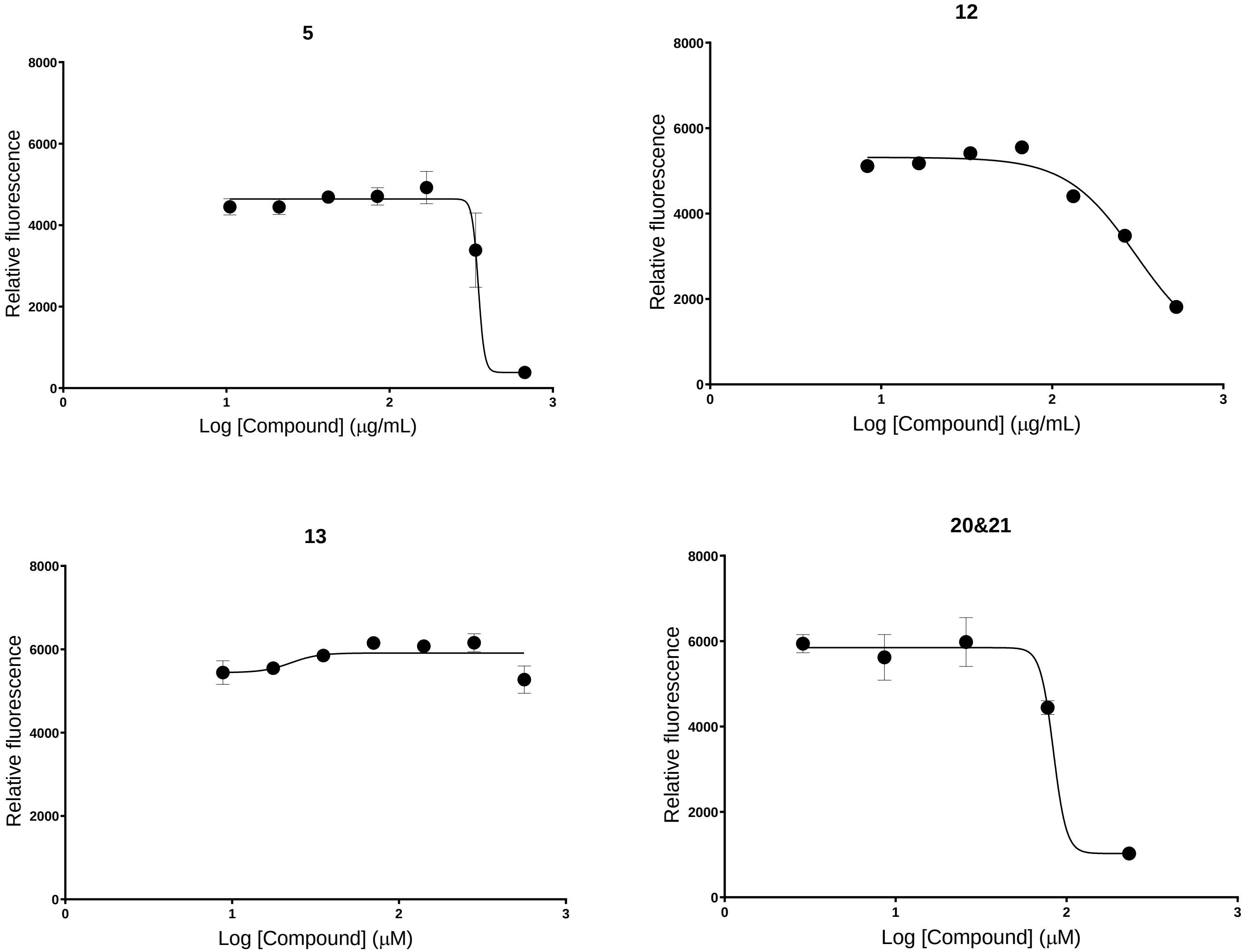

Figure S151. Measurements of the antibacterial activity of Ampicillin, as a positive control antibiotic tested against Gram-negative *Escherichia coli* and Gram-positive *Bacillus subtilis* following the same assays conditions as for the isolated natural compounds (see experimental section). Ampicillin showed an EC_{50} = 11.5 µM (SD = 0.721; SE range of 11.065 to 11.898 µM) and an EC_{90} = 89.2 µM (SD = 14.807; SE range of 81.493 to 98.591 µM) against *E. coli* and

an EC_{50} = 17.2 µM (SD = 8.910; SE range of 13.490 to 23.778 µM) and an EC_{90} = 160.4 µM (SD = 42.786; SE range of 97.127 to 146.531 µM) against *B. subtilis*, as previously reported (European Committee on Antimicrobial Susceptibility Testing. Data from the EUCAST MIC distribution website, last accessed November 2020". http://www.eucast.org).

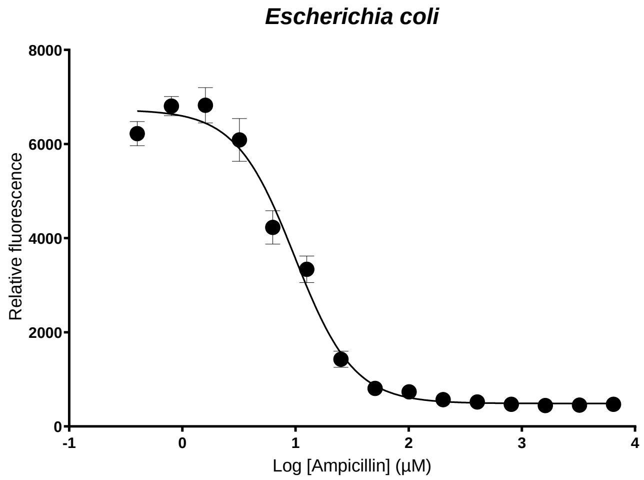

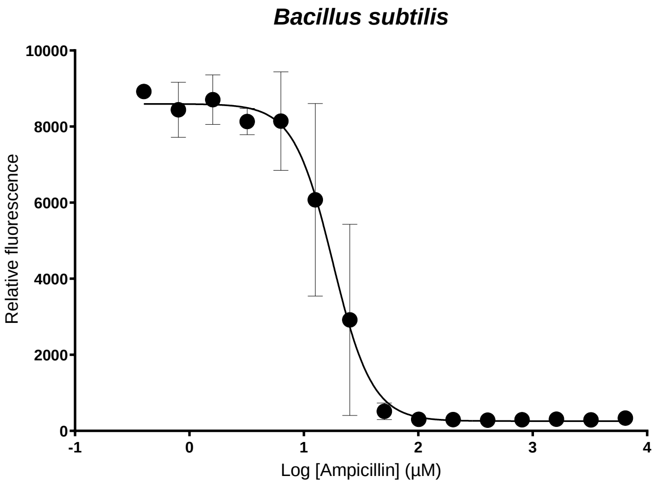

Supplement: Supplementary file 1 — np1c00811_si_001.pdf [file np1c00811_si_001.pdf]
